# Evaluating the Importance of Conformers for Understanding the Vacuum-Ultraviolet Spectra of Oxiranes: Experiment and Theory

**DOI:** 10.1021/acs.jpca.4c04391

**Published:** 2024-12-06

**Authors:** Ian T. Beck, Erica C. Mitchell, Annabelle Webb Hill, Justin M. Turney, Brandon Rotavera, Henry F. Schaefer

**Affiliations:** † Department of Chemistry, University of Georgia, 302 East Campus Road, Athens, Georgia 30602, United States; ‡ Center for Computational Quantum Chemistry, University of Georgia, 1004 Cedar Street, Athens, Georgia 30602, United States; § College of Engineering, University of Georgia, 597 D.W. Brooks Drive, Athens, Georgia 30602, United States

## Abstract

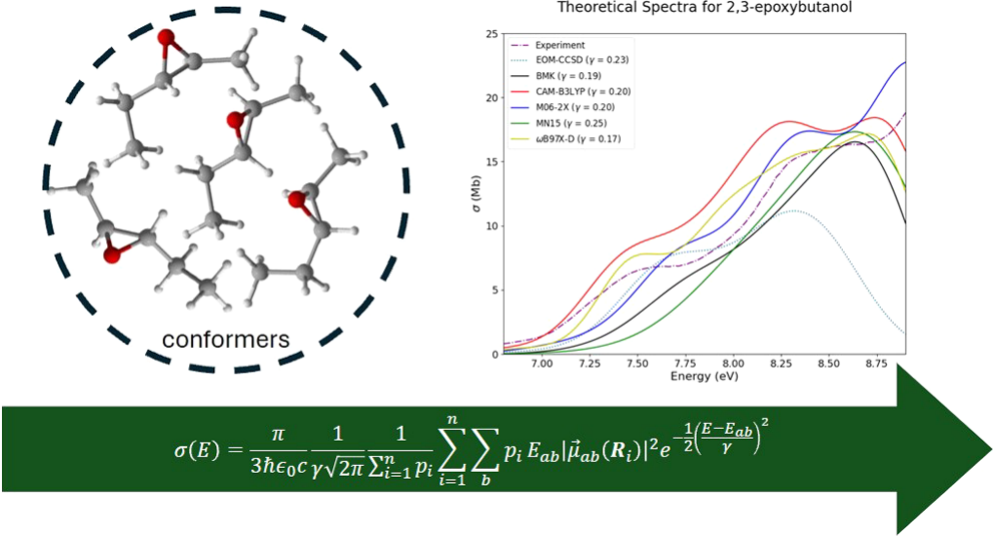

Vacuum-ultraviolet (VUV) absorption spectroscopy enables electronic transitions that offer the unambiguous identification of molecules. As target molecules become more complex, multifunctional species present a great challenge to both experimental and computational spectroscopy. This research reports both experimental and theoretical studies of oxiranes. Computationally, the nuclear ensemble approach has been used to accurately predict experimental spectra for a variety of molecules. However, this approach incurs great computational cost, as ensembles generally consist of thousands of geometries. The present study aims to drastically reduce the ensemble by evaluating the significance of the conformers to the predicted spectra. This approach was applied to 11 substituted oxiranes using the Conformer Rotamer Ensemble Sampling Tool (CREST) of Grimme to generate an ensemble of unique conformers determined by their Boltzmann populations. Five TD-DFT functionals (BMK, CAM-B3LYP, M06-2X, MN15, ωB97X-D) and EOM-CCSD were used to simulate the spectrum of each substituted oxirane ensemble. Computed spectra were then compared to the experiment using both qualitative and quantitative metrics. Based on these metrics, it was observed that certain conformers may not be necessary to characterize this set of oxiranes despite the temperature (323 K) of the experiment. A single conformer can then be used with TD-DFT and EOM-CCSD to replicate the experimental spectra of these medium-sized combustion species.

## 1 Introduction

Vacuum-ultraviolet (VUV) absorption spectroscopy is an important technique that plays vital roles in both catalysis^1−3^ and combustion^4−6^ characterization. In combustion, identification of intermediary species is requisite for the investigation of chemical kinetics models.^7−9^ VUV absorption spectroscopy provides a useful medium by which to study and identify these species. The VUV region (100–200 nm, 6.2–12.4 eV) enables valence and Rydberg electronic transitions that allow for the identification of stereoisomers more easily than when compared to other forms of absorption spectroscopy.^10,11^ Experimental absorption spectra, however, may not always be feasible to measure due to some chemical species being unstable or having too short a lifetime to measure. In instances where experimental spectra are unavailable, theoretically predicted spectra are essential to filling the gap. In addition, theoretical spectra can be utilized in conjunction with experimental data to help elucidate the vibronic features and electronic characteristics of absorption bands.^12−16^ There are several methods that are commonly used to theoretically predict VUV absorption spectra.^17^ These methods entail a mix of computational cost, accuracy, and complexity, all of which must be taken into account when choosing which method is best suited to a particular study. An ideal method is computationally inexpensive, highly accurate, and simple enough that it can be easily implemented.

Determining the photoabsorption cross section, σ(*E*), is one way that theoretical spectra can be evaluated. There are two main classes of methods that are used to determine σ(*E*): (a) time-dependent methods and (b) time-independent methods. Time-dependent methods are often complex and computationally expensive, so time-independent methods are more commonly used. The simplest time-independent method utilized is the vertical approximation, which produces a “stick spectrum”. This method is used to compute the vertical transitions from Franck–Condon factors, which are then plotted with Gaussian or Lorentzian broadening to mimic the spectral line shape.^14,18^ The artificial broadening brought about by these functions is useful for comparison to experimental spectra but gives no actual insight into the broadening of the spectra. The main advantage of stick spectra is that they are simple and easy to implement, and they can often give a good approximation to experimental spectra.^14,19,20^

One popular method to increase the accuracy of stick spectra is a time-independent method called the nuclear ensemble approach (NEA), or sometimes the nuclear ensemble method. The NEA is based on the idea that absorption spectra can be computed from a distribution of geometries to represent the ground-state nuclear density.^21−24^ Electronic transition properties are computed for each geometry in the ensemble, and the corollary is that the resulting spectrum may have a higher resolution than methods that compute only a single vertical transition. The NEA is a post-Condon approximation, meaning it can account for non-Condon effects that arise from the dependence of the transition dipole moment on nuclear coordinates. In its formulation for the absorption cross section, the NEA accounts for this dependence on nuclear geometry, eq 1. The guiding equation for the NEA is given as^21^
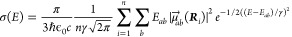
1where *E*_*ab*_ and μ⃗_*ab*_ are the excitation energy and corresponding transition dipole moment, respectively, from initial state *a* to final state *b*. The sums run over *b* final states for *n* geometries in the ensemble, and γ is the bandwidth parameter that determines how broad the spectrum is. A formal derivation of the NEA was done in 2012;^21^ however, the principle behind the NEA has been the subject of theoretical studies since the 1980s when it was known as the reflection principle.^25,26^

A problem often cited with the NEA is sampling; we want to sample an ensemble in a way that captures accuracy but is still small enough that it does not involve great computational cost. The most common sampling approaches for the NEA are Wigner sampling and molecular dynamics (MD) simulations.^27−31^ Both of these sampling methods use initial positions and momenta to produce distributions of geometries.^32,33^ Both sampling methods have been shown to be reliable in producing a complete ensemble.^30,32−36^ However, there is a distinct disadvantage to both of these methods, as they typically produce an ensemble with over thousands of geometries.^17,32^ A large ensemble limits the quality of the electronic structure method that can be used to compute the electronic transition energies and transition dipole moments. Previous studies have examined other methods of sampling in an effort to reduce the size of the ensemble produced by either Wigner or MD simulations. These studies include various machine learning (ML) models such as kernel-ridge-regression models,^34^ and Gaussian mixture modeling.^31^

In the NEA, the total number of geometries needed to represent the ground-state nuclear density is dependent on the number of conformers identified. As defined by Grimme and co-workers, conformers are stereoisomers described by distinct potential energy minima, while rotamers are degenerate potential energy minima produced from rotation around a bond that interchange nuclei of the same element.^37^ Conformers provide insight into the state in which a molecule is likely to be, in particular when considering Boltzmann populations at a given temperature. As a result, conformers are often utilized as a starting point when generating a Wigner distribution, which spawns about 500 geometries per conformer.^17,38,39^ The Conformer Rotamer Ensemble Sampling Tool (CREST) is a program that generates a conformer-rotamer ensemble (CRE) and was developed by Grimme and co-workers.^37^ CREST implements a method called iMTD-GC that utilizes metadynamics (MTD) sampling to search the conformational space of a molecule and a semiexperimental method called GFN2-xTB for optimizing structures that have been determined from the MTD simulation. This method has been shown to produce reliable CREs in the areas of metabolomics,^40^ quantum chemistry automation,^41^ predicting NMR spectra,^42^ and others.^43−45^ With its use of metadynamics, root-mean-squared deviations, and energy screening, CREST produces about  conformers per input molecule.

This study seeks to utilize CREST as a starting point for a reduced ensemble to generate theoretical spectra. This study is not the first to examine the use of a small-sized ensemble for computing theoretical spectra. In 2021, Sršeň and Slavíček examined a representative sampling method to select a subset of nuclear configurations to be used for the NEA in a way that still represents the entire density of the sample.^24^ They found that one geometry chosen with their representative method still performed better than one geometry selected at random when compared to experiment. Since CREST samples the conformational space with a basis in energetics and root-mean-squared deviations (RMSDs) of atomic positions, this guarantees that no two conformers will (a) be identical or (b) be in the same conformational well on the potential energy surface (PES) of the molecule. Both of these approaches ensure that local minima, which could be populated at experimental temperatures, are represented in addition to the global minimum of the PES. Due to the reliability and sampling methods of CREST, we suggest that it can be used to produce a sufficient ensemble of conformers to be utilized for the NEA.

In this work, CREST is used to generate conformers for 11 oxirane species, which are important metastable intermediates in QOOH combustion reaction pathways.^4,46^ The theoretical spectrum of each oxirane is predicted using all of its conformers with their respective Boltzmann weights and then compared to the spectrum of its single lowest energy conformer. Each spectrum is predicted using the NEA with vertical excitation energies and transition dipole moments computed using five TD-DFT functionals and EOM-CCSD. The TD-DFT functionals utilized the heavily augmented d-aug-cc-pVTZ basis set while EOM-CCSD used the slightly smaller d-jul-cc-pVTZ basis to capture the high-energy valence electron transitions (e.g., σ → σ* and n → σ*) and Rydberg transitions characteristic to VUV spectroscopy. All predicted spectra were scrutinized using quantitative metrics and directly compared to high-resolution gas-phase VUV absorption spectroscopy experiments. Overall, this study aims to (a) assess the importance of conformational sampling when simulating VUV spectra, (b) test whether a conformer-only ensemble is sufficient to reproduce experimental spectra, and (c) accurately predict molecular spectra to within ±5 Mb using density functional and wave function methods.

## 2 Methods

### 2.1 Computational Method

Conformational sampling was done using CREST to generate conformer-rotamer ensembles with the semiempirical method, GFN2-xTB.^37^ However, only the conformers were used to produce our ensemble since the rotamers did not provide unique geometries. All of the default values for energy thresholds were used, while the temperature was set at 323 K for direct comparison to the experimental spectra. The conformers produced from CREST were then further optimized with density functional theory (DFT) and density-fitted coupled-cluster singles, doubles, and perturbative triples (DF-CCSD(T)) methods. Optimizations used the minimally augmented jun-cc-pVTZ basis set.^47^ Optimizations were done in embarrassingly parallel fashion utilizing Psi4 1.6 single-point energies and an in-house interface to Psi4’s finite difference driver.^48^ To ensure that an ensemble of unique geometries was obtained, the root-mean-squared deviations of atomic coordinates (RMSDs) of each conformer were compared to each other with duplicates being removed. When RMSDs were compared between structures, the threshold was set to 0.125, which is the default set by CREST. This means if any two structures have an RMSD comparison less than this value, they will be deemed similar, and the one with the higher energy will be thrown out. Following optimizations, ensembles were returned to CREST to generate Boltzmann populations and reduce the ensemble a final time with the CREGEN standalone method.

**Figure 1:**
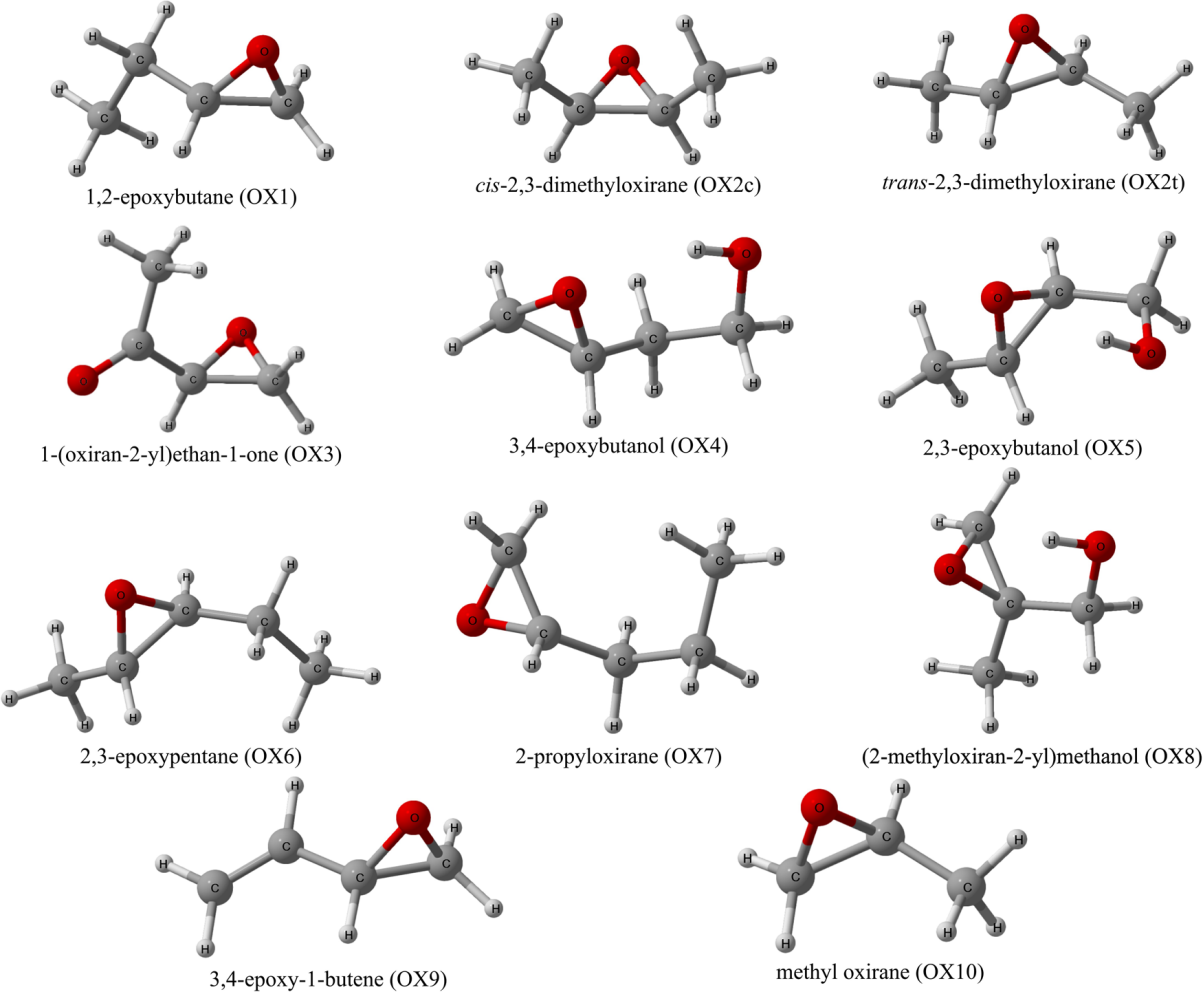
Ground-state structures of the substituted oxirane species from the initial GFN2-xTB optimizations obtained from CREST.

The QChem 5.0 program was used to calculate excited-state properties using equation-of-motion coupled-cluster singles and doubles (EOM-CCSD) and time-dependent density functional theory (TD-DFT) methods, respectively.^49−51^ While TD-DFT used the large, doubly augmented d-aug-cc-pVTZ basis set, the costly EOM-CCSD computations necessitated the use of the smaller d-jul-cc-pVTZ basis set, which removes diffuse functions from the hydrogens.^52,53^ TD-DFT also utilized the SG-3 grid (99 radial points and 590 angular points) in order to properly characterize Rydberg transitions. TD-DFT functionals have been chosen based on a study by Bralick and co-workers that examined the reliability of TD-DFT functionals for reproducing theoretical VUV absorption spectra.^54^ The study examined 10 functionals, and we have chosen to use the best four functionals they found, M06-2X, BMK, CAM-B3LYP, and ωB97X-D; additionally, we use MN15.^55−59^ Enough excitations were computed that theoretical spectra would span the onset energy plus approximately 1 eV, in accordance with the experimental spectra. After all excited-state calculations were complete, a modified version of Psi4’s spectrum.py was used to produce the spectra using eq 2. This equation is similar to eq 1, but instead uses a weighted average of Boltzmann populations, *p*_*i*_, for each conformer. The broadening factor in this equation, γ, was set such that the shape of the absorption cross section best matched the experiment. The substituted oxiranes that have been selected for study, Figure 1, have been experimentally characterized via high-resolution gas-phase vacuum-ultraviolet absorption spectroscopy (Section 2.2).^60^
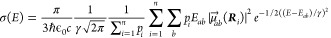
2

To assess how well our theoretical methods are able to reproduce spectra, quantitative comparisons have been made. Quantitative measurements include the relative integral change (RIC), eq 3, given as
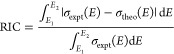
3where the definite integrals span the selected energy region (*E*_1_ to *E*_2_), and σ(*E*) is the absorption cross section for the experimental (expt) or theoretical (theo) spectrum. This metric measures the difference in the areas under the absorption cross section in the selected region. Values for RIC typically range from 0 to 1 with a value of 0 corresponding to a perfect match, and values greater than 1 correspond to significant differences in area. Additionally, the mean signed error (MSE), eq 4, is used to measure the average difference between experiment and theory. This is given as
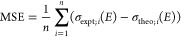
4where *n* is the number of points computed for a theoretical spectrum, and again σ(*E*) is the absorption cross section. The final quantitative metric is the mean absolute error (MAE), given with eq 5 as
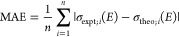
5While MSE yields a signed value, MAE gives an absolute measurement of the average error.

### 2.2 Experimental Section

Differential absorption spectroscopy experiments were employed to here measure the absorption cross section of gas-phase methyl oxirane in the same manner as in Doner et al.,^61^ in which measures are taken to control purity, contamination of the flow cell with ambient air and signal saturation. The temperature and pressure of the flow cell were 50 °C and 800 Torr, respectively. Liquid-phase methyl oxirane was purified via the freeze–pump–thaw method to remove dissolved gases and volatile impurities prior to manometric preparation of binary mixtures with He, which is used for dilution. Absorption measurements were conducted in the gas phase using a temperature- and pressure-controlled flow cell with a path length of 10 cm. White light is produced from a deuterium (D_2_) lamp and directed through the flow cell using high-reflectivity mirrors positioned at the inlet and exit. The latter mirror directs unabsorbed white light onto a diffraction grating, which provides high-resolution separation (<4 meV) onto a CCD detector that produces output voltages corresponding to wavelength. The output signal is then processed via an A/D converter and sent to a data-acquisition system for postprocessing. Prior to each absorption measurement, a reference scan (to measure background absorbance) and a dark scan (with the light source, a D_2_ lamp, blocked) were performed sequentially using a He flow for 1 min to enable background subtraction and proper determination of *I*_0_. Time-averaged absorption signals were then measured. To quantify absorption cross sections as a function of photon energy, σ(*E*) (cm^2^), the Beer–Lambert law was utilized. Units for absorption cross sections are reported in megabarns (Mb); 1 Mb = 10^–18^ cm^2^.

## 3 Results and Discussion

Eleven molecular systems have been closely examined in order to (a) assess the importance of conformational sampling when generating VUV spectra and (b) test the reproducibility of the experimental spectra with a conformer ensemble from CREST. Oxiranes were chosen as the group of molecules for their applicability to combustion chemical kinetics models, which rely on calculated concentrations from absorption spectroscopy.^10^ Spectra for all systems were produced with the five TD-DFT functionals mentioned above (BMK, CAM-B3LYP, M06-2X, MN15, and ωB97X-D) as well as with EOM-CCSD. This section is presented with careful examinations of TD-DFT and EOM-CCSD methods, compared to experimental spectra in order to assess performance. Quantitatively, the relative integral change (RIC), mean signed error (MSE), and mean absolute error (MAE) will be assessed and compared among methods. These quantitative metrics are measured only within the bounds of the presented spectra, up to approximately 1 eV past the experimental onset energy. Geometric parameters for all oxiranes presented are available in the Supporting Information.

After structure optimizations with the DFT and coupled cluster methods, all substituted oxirane systems examined present less than 25 conformers in the final ensemble. Three of the oxiranes examined resulted in only one conformer. To assess the importance of conformational sampling, we present only the spectra for oxiranes with more than one conformer. These are then compared to their spectra generated with only the lowest energy conformational isomer. Comparisons between these sets of spectra give insight into the importance of conformers for simulating VUV spectra for these substituted oxirane systems.

It should be noted that regardless of conformational sampling, red- and blue-shifting commonly occur with theoretical spectra when compared to experiment. This arises from the amount of exact Hartree–Fock exchange included in the TD-DFT functional.^62^ Functionals that include less exchange tend to underestimate vertical excitations, and ones that include more exchange tend to overestimate vertical excitations.^63,64^ The other main factor that affects the spectral shape is the lack of vibrational contributions, brought about by the vertical approximation.^21^ While methods exist to include vibrational coupling to electronic transitions,^65,66^ they are often too computationally expensive for the accuracy gain,^67^ especially when using heavily augmented basis sets for systems with greater than 5–6 nonhydrogen atoms, such as the ones in this study.

### 3.1 Ensemble Size Comparisons

As stated earlier, eight substituted oxiranes are presented in this section, with analyses comparing spectra generated with multiple conformers to spectra obtained with only the lowest energy conformer in the ensemble. In some instances, γ values have been adjusted between multiconformer spectra and single-conformer spectra. The value of γ within the NEA adjusts the height and broadening of the Gaussian functions that are overlaid and only serves to align the theoretical spectra better with experiment. Quantitative metrics (RIC, MSE, and MAE) as well as values for γ are presented in Table 1 for all examined oxiranes whose spectra were produced with multiple conformers. Corresponding values for spectra that are generated with only the lowest energy conformer are presented in Table 2.

**Table 1:** Theoretical Bandwidth Parameters (γ) in eV, Relative Integral Change (RIC) in Arbitrary Units, Mean Signed Error (MSE) in Mb, and Mean Absolute Error (MAE) in Mb for Each Substituted Oxirane Whose Spectra Were Generated with Multiple Conformers

		EOM-CCSD	BMK	CAM-B3LYP	M06-2X	MN15	ωB97X-D
OX1	γ	0.10	0.09	0.12	0.14	0.15	0.09
	RIC	0.540	0.518	0.407	0.442	0.512	0.498
	MSE	–5.265	–5.179	–2.958	–4.163	–5.669	–3.940
	MAE	5.456	5.685	4.329	4.741	5.669	5.454
OX3	γ	×	0.24	0.22	0.26	0.25	0.24
	RIC	×	1.045	0.903	0.893	1.572	0.974
	MSE	×	4.075	4.220	3.761	6.538	4.460
	MAE	×	4.940	4.328	4.180	7.460	4.610
OX4	γ	×	0.28	0.30	0.32	0.30	0.32
	RIC	×	0.556	1.602	1.044	0.558	1.213
	MSE	×	0.597	2.865	1.719	0.257	2.115
	MAE	×	0.993	2.885	1.807	1.416	2.155
OX5	γ	0.23	0.19	0.20	0.20	0.25	0.17
	RIC	0.225	0.265	0.319	0.194	0.311	0.160
	MSE	–1.353	–1.974	2.141	0.029	–2.359	0.666
	MAE	1.585	1.974	2.208	1.412	2.363	1.166
OX6	γ	0.18	0.15	0.16	0.22	0.15	0.18
	RIC	0.511	0.546	0.339	0.413	0.531	0.419
	MSE	–5.608	–6.494	–3.380	–4.234	–7.466	–4.544
	MAE	5.608	6.494	3.629	4.492	7.466	4.544
OX7	γ	0.21	0.20	0.25	0.28	0.23	0.23
	RIC	0.455	0.475	0.238	0.295	0.474	0.343
	MSE	–4.190	–4.551	–2.090	–2.678	–5.467	–3.183
	MAE	4.190	4.551	2.157	2.804	5.467	3.183
OX8	γ	0.21	0.13	0.20	0.18	0.30	0.13
	RIC	0.394	0.370	0.230	0.224	0.372	0.273
	MSE	–3.899	–3.899	–1.264	–2.320	–3.743	–2.336
	MAE	3.952	3.899	2.350	2.382	3.949	2.844
OX9	γ	0.50	0.47	0.50	0.45	0.46	0.50
	RIC	0.375	0.106	0.205	0.123	0.174	0.167
	MSE	–0.595	3.081	5.504	3.282	3.443	4.551
	MAE	12.052	3.227	5.504	3.580	4.851	4.714

**Table 2:** Theoretical Bandwidth Parameters (γ) in eV, Relative Integral Change (RIC) in Arbitrary Units, Mean Signed Error (MSE) in Mb, Mean Absolute Error (MAE) in Mb for Each Substituted Oxirane Whose Spectra Were Generated with One Conformer

		EOM-CCSD	BMK	CAM-B3LYP	M06-2X	MN15	ωB97X-D
OX1	γ	0.10	0.11	0.15	0.14	0.15	0.12
	RIC	0.559	0.484	0.351	0.427	0.538	0.406
	MSE	–6.107	–4.875	–2.591	–3.835	–5.887	–3.606
	MAE	6.251	5.294	3.743	4.645	5.887	4.373
OX2c	γ	0.13	0.13	0.13	0.13	0.15	0.12
	RIC	0.463	0.397	0.309	0.347	0.434	0.317
	MSE	–4.189	–3.787	–0.877	–1.660	–4.225	–2.137
	MAE	4.725	3.898	2.732	3.349	4.357	3.034
OX2t	γ	0.11	0.10	0.09	0.23	0.13	0.09
	RIC	0.436	0.536	0.340	0.404	0.583	0.379
	MSE	–4.455	–5.177	–2.295	–3.964	–5.793	–3.586
	MAE	4.559	5.177	3.213	4.023	5.793	3.799
OX3	γ	×	0.24	0.22	0.26	0.25	0.24
	RIC	×	1.048	0.911	0.892	1.639	0.976
	MSE	×	3.940	4.123	3.691	6.603	4.556
	MAE	×	4.859	4.341	4.146	7.660	4.726
OX4	γ	×	0.30	0.34	0.32	0.30	0.36
	RIC	×	0.525	1.867	1.002	0.563	1.262
	MSE	×	0.670	3.343	1.586	0.996	3.174
	MAE	×	1.005	3.376	1.708	2.269	3.174
OX5	γ	0.18	0.15	0.19	0.20	0.20	0.16
	RIC	0.230	0.300	0.283	0.160	0.325	0.157
	MSE	–1.293	–2.219	1.890	–0.106	–2.769	0.480
	MAE	1.674	2.219	1.996	1.177	2.776	1.150
OX6	γ	0.15	0.12	0.14	0.20	0.13	0.15
	RIC	0.564	0.546	0.362	0.393	0.496	0.474
	MSE	–6.316	–6.652	–3.869	–4.276	–7.102	–5.060
	MAE	6.316	6.652	3.869	4.304	7.102	5.060
OX7	γ	0.18	0.18	0.2	0.27	0.2	0.23
	RIC	0.453	0.456	0.271	0.292	0.371	0.339
	MSE	–4.413	–5.062	–2.054	–2.452	–4.740	–3.107
	MAE	4.413	5.062	2.436	2.944	4.740	3.107
OX8	γ	0.22	0.13	0.21	0.18	0.3	0.14
	RIC	0.369	0.348	0.230	0.227	0.336	0.268
	MSE	–4.232	–4.237	–1.257	–2.316	–4.199	–2.273
	MAE	4.288	4.237	2.373	2.415	4.532	2.789
OX9	γ	0.50	0.47	0.50	0.45	0.46	0.50
	RIC	0.408	0.193	0.274	0.237	0.252	0.242
	MSE	4.258	5.770	7.470	6.354	6.784	6.911
	MAE	12.889	5.845	7.470	6.354	7.297	6.911
OX10	γ	0.14	0.11	0.15	0.10	0.15	0.13
	RIC	0.420	0.460	0.420	0.482	0.358	0.460
	MSE	–4.342	–5.064	–2.771	–3.381	–4.569	–3.672
	MAE	4.476	5.254	4.167	5.012	4.819	4.749

**Figure 2:**
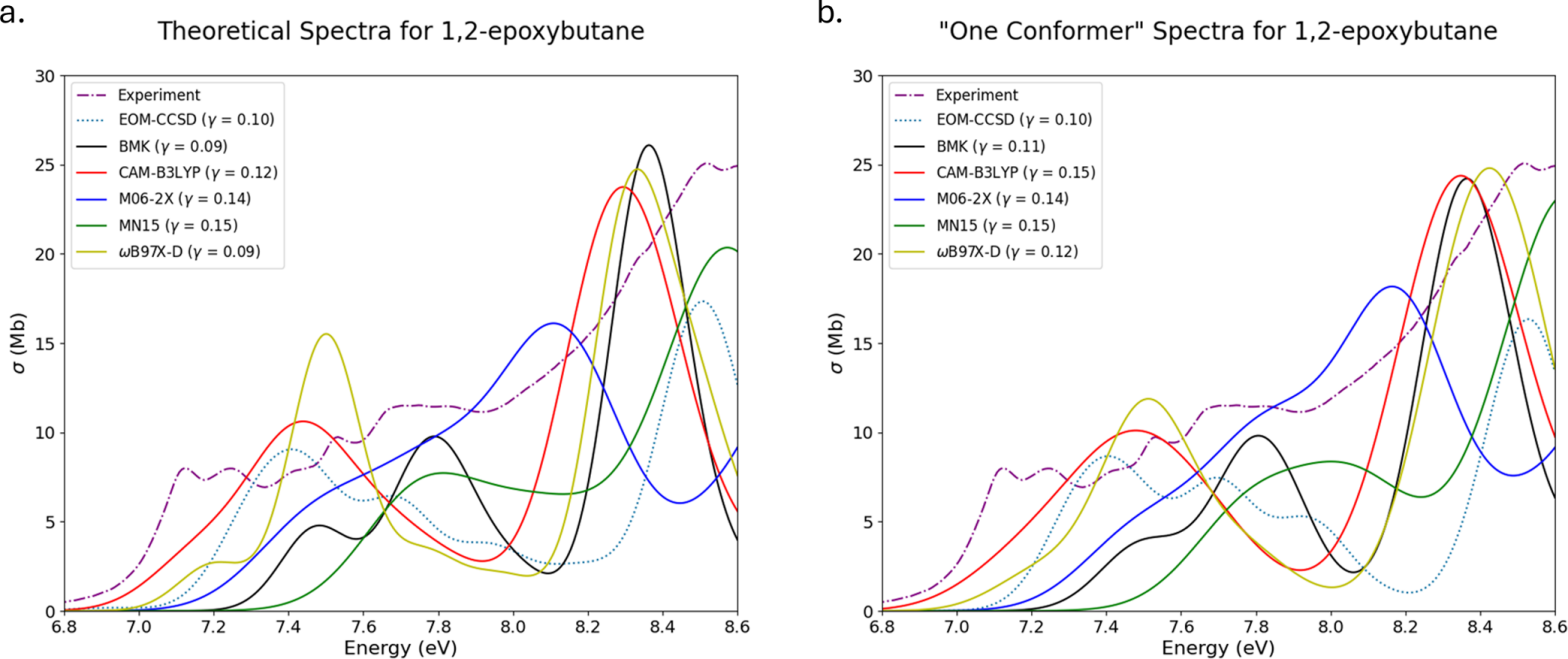
Experimental, EOM-CCSD, and TD-DFT spectra for 1,2-epoxybutane (OX1) overlaid together for comparison. (a) Computed from ensembles with multiple conformers. (b) Computed from ensembles with only one conformer.

The spectra for 1,2-epoxybutane (OX1) are presented in Figure 2a,b for multi- and single-conformer spectra, respectively. There are subtle differences between these sets of theoretical spectra; however, both sets are largely representative of the features on the experimental spectrum. The local maximum peak in the experimental spectrum, around 8.5 eV, is accounted for with all theoretical methods presented. In the single-conformer spectra; however, several of the methods are able to predict the energy of the feature slightly closer to experiment, e.g., ωB97X-D predicting 8.3 eV for multiconformer and 8.4 eV for single conformer. These differences arise from the averaging with Boltzmann weights for multiple conformers. The lowest energy conformers that were used for constructing the single-conformer spectra for OX1 had Boltzmann populations ranging from 33 to 43%, depending on the method. The exact Boltzmann populations for each structure are presented in the Supporting Information. In the multiconformer spectra, these conformers are weighted with other conformers that may contribute less to the overall population and cause a shift from the experimental spectrum. The lowest energy structures used in one-conformer spectra are not necessarily the conformers with the greatest Boltzmann population. However, these comparisons still show the importance of selecting a conformer that is representative of the entire system.

**Figure 3:**
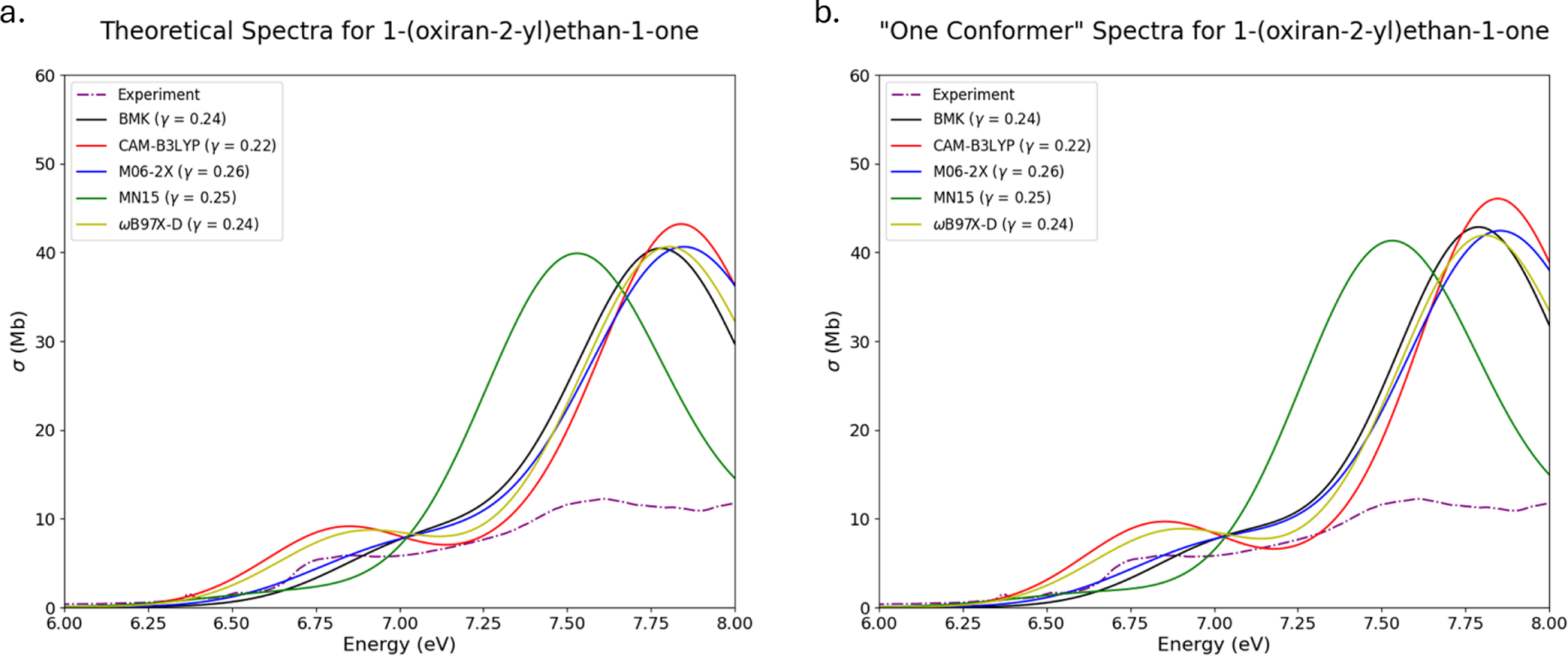
Experimental, EOM-CCSD, and TD-DFT spectra for 1-(oxiran-2-yl)ethan-1-one (OX3) overlaid together for comparison. (a) Computed from ensembles with multiple conformers. (b) Computed from ensembles with only one conformer.

OX3 is the next oxirane under examination, theoretical spectra for multi- and single-conformer spectra are presented in Figure 3a,b, respectively. For both sets of theoretical spectra, the absorption cross section is notably higher in intensity than the experimental spectrum. This is likely a result of the vertical approximation;^68,69^ however, both experimental features are represented by most of the theoretical methods employed. The differences between these sets of spectra are sparse due to the lowest energy conformer possessing a Boltzmann population of at least 90% across the methods employed. The large Boltzmann population heavily weighs the multiconformer spectra to the lowest energy conformer that produces the single-conformer spectra, resulting in two sets of spectra that are very similar.

**Figure 4:**
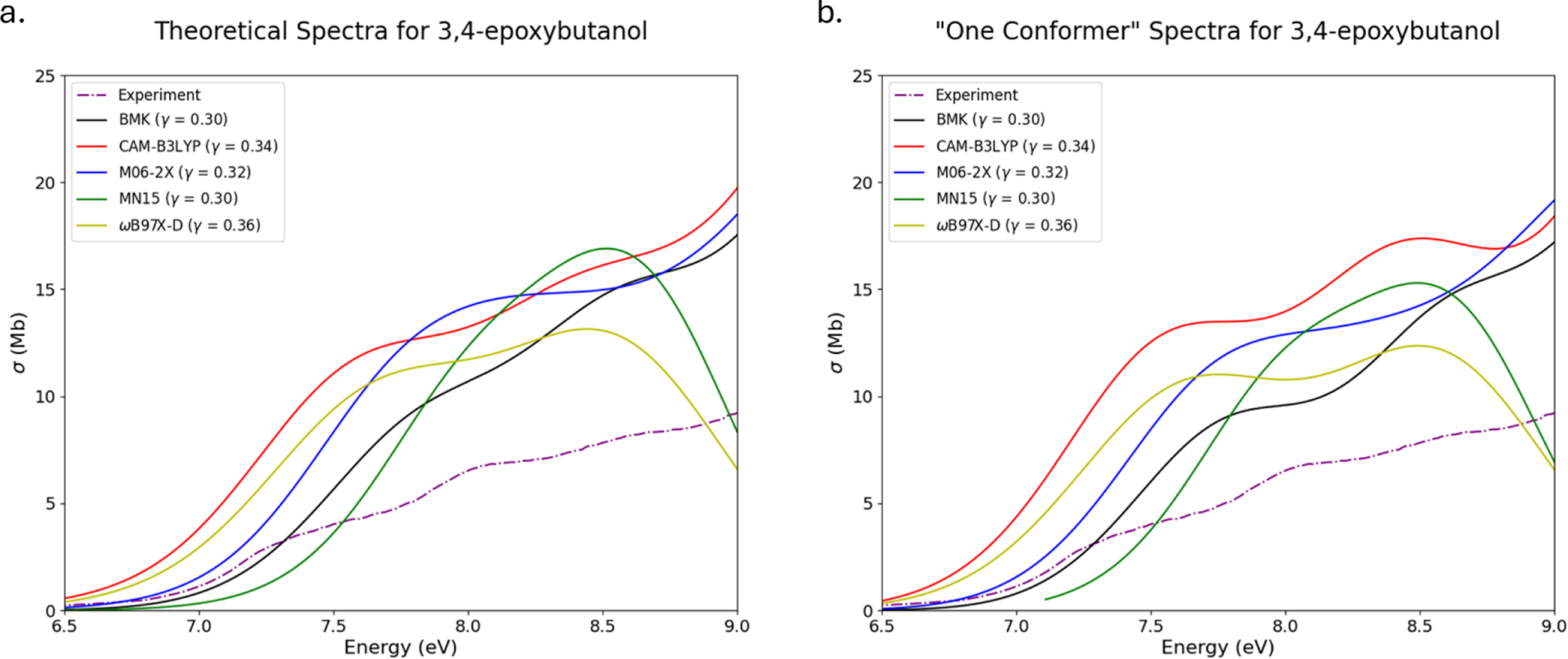
Experimental, EOM-CCSD, and TD-DFT spectra for 3,4-epoxybutanol (OX4) overlaid together for comparison. (a) Computed from ensembles with multiple conformers. (b) Computed from ensembles with only one conformer.

The spectra for 3,4-epoxybutanol (OX4) are presented in Figure 4a,b for multi- and single-conformer spectra, respectively. OX4 was the substituted oxirane with the largest final ensemble of 22 conformers. This is unsurprising given the structure of OX4 (cf. Figure 1); with an ethanol substituent on this oxirane, there are a multitude of conformational isomers that may present as local minima on OX4’s potential energy surface. The experimental spectrum for OX4 is largely lacking in experimental features in the selected region. The primary experimental features for this substituted oxirane are present as small shoulders as the absorption cross section progresses toward higher energies. Both sets of theoretical spectra vastly overestimate the absorption cross sections when compared to experiment. There are shoulders present in most of the theoretical spectra that are comparative to experiment, in particular with the single-conformer spectra the features are slightly more pronounced than in the multiconformer spectra. Boltzmann populations of the lowest energy conformers in the multiconformer spectra range from 30 to 49%, while the majority of the remaining conformers present Boltzmann populations <5%. This results in theoretical spectra that are too averaged and dissimilar to experiment when compared to spectra generated with the lowest energy, and, in this case, highest Boltzmann population.

**Figure 5:**
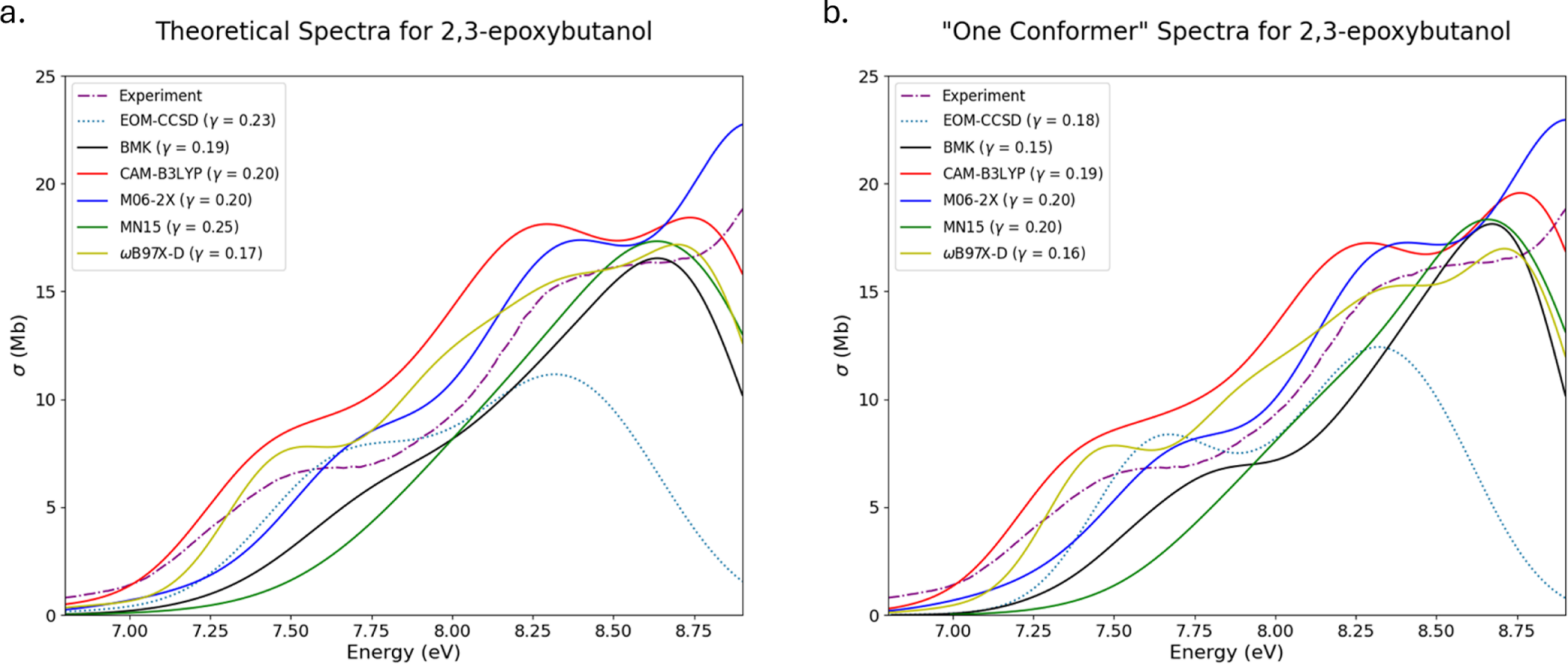
Experimental, EOM-CCSD, and TD-DFT spectra for 3,4-epoxybutanol (OX5) overlaid together for comparison. (a) Computed from ensembles with multiple conformers. (b) Computed from ensembles with only one conformer.

Theoretical and experimental spectra for 2,3-epoxybutanol (OX5) are presented in Figure 5a,b for spectra generated with multiple-conformer ensembles and single-conformer ensembles, respectively. The final ensemble used to generate the multiconformer spectra consisted of 7 oxiranes for each method; however, the lowest energy conformers present Boltzmann populations ranging from 65 to 87% (average 74%). Since the lowest energy structure used for constructing the single-conformer spectra has Boltzmann populations that are so large, there are no significant differences in peak locations. However, there are differences in feature height ratios and convolutions. For example, comparing the TD-BMK spectra reveals that the lowest energy conformer predicts an onset shoulder but becomes convoluted when averaged with other conformers which removes the prominence of the shoulder. Another small example lies in the height differences toward the end of the TD-CAM-B3LYP spectra. In the multiconformer TD-CAM-B3LYP spectrum, the last two peaks are almost the same height in absorption cross section, but in the single-conformer spectrum, the ratio of the heights of the last peak to the second to last peak is larger, as is present in the experimental spectrum. In both of these examples, the lowest energy conformer predicts spectral features that align better with the experimental spectrum than the multiconformer spectra. These features in the theoretical spectra are able to be slightly modified to align better with experiment by adjusting the value of γ, but these adjustments are not able to change peak height ratios or the presence of spectral features. The idiosyncrasies of these features in the single-conformer spectra are suppressed in the multiconformer spectra when other conformers are utilized that are not primary contributors to the overall population of conformational isomers.

**Figure 6:**
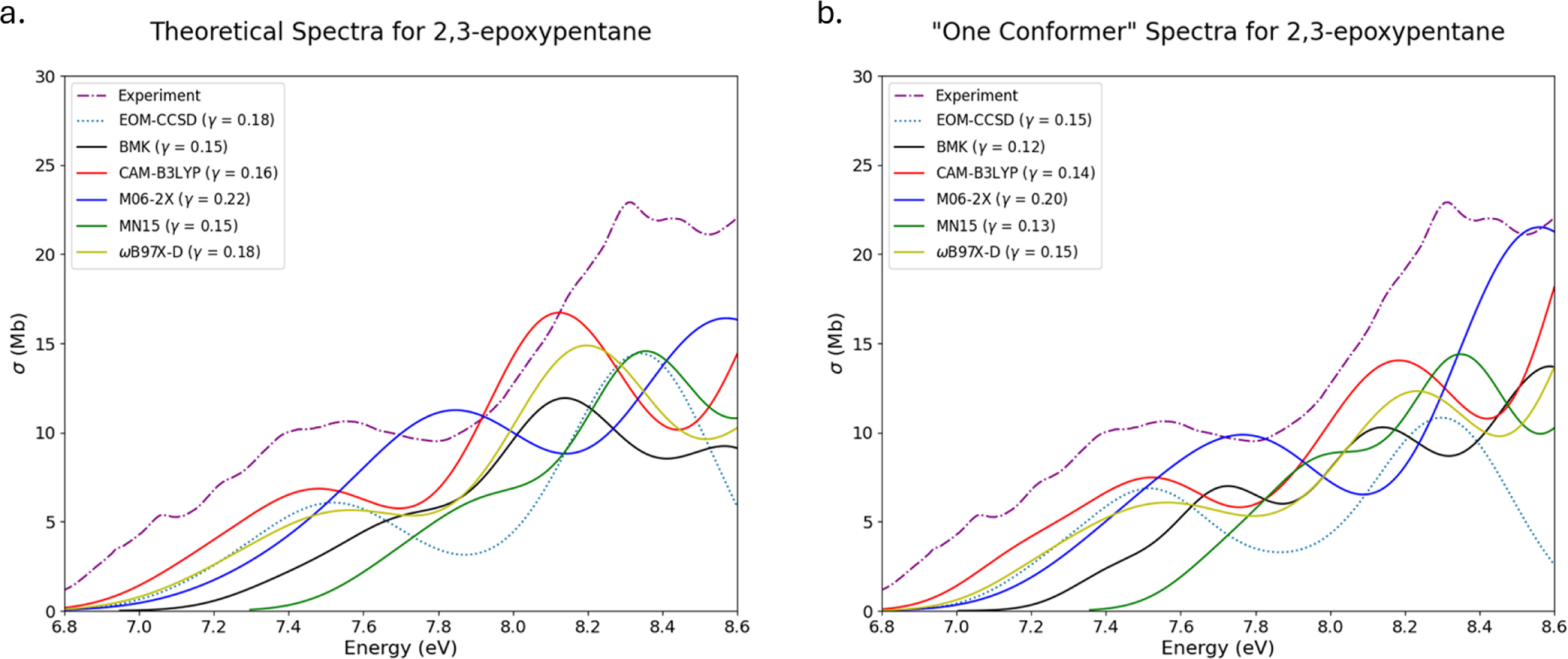
Experimental, EOM-CCSD, and TD-DFT spectra for 2,3-epoxypentane (OX6) overlaid together for comparison. (a) Computed from ensembles with multiple conformers. (b) Computed from ensembles with only one conformer.

Multi- and single-conformer spectra for 2,3-epoxypentane (OX6) are presented in Figure 6a,b. All examined methods for generating excitation properties for theoretical spectra resulted in final ensembles composed of three conformers each. The selected region of the experimental spectrum of OX6 can be described by two primary features: one feature just after the onset near 7.5 eV, and another, the local maximum, around 8.3 eV. All of the methods utilized seemingly capture both of these experimental features in both sets of spectra, except for the MN15 functional, which does not represent the onset feature well. The most evident difference between these two sets of spectra is present in the TD-M06-2X spectra. In the spectrum produced by the single-conformer ensemble, the height of both features is represented very well, although there is a noticeable energy shift from the experiment. However, the Boltzmann population of the lowest energy conformer that was used to produce this spectrum is the lowest of the three conformers used in the multiconformer counterpart. A quite unexpected result; typically, it would make sense that the conformer with the largest Boltzmann population would produce the best single-conformer spectrum, being the most representative of a system at a given temperature. This is striking, as the lowest energy conformer has a Boltzmann population of 24%, versus the other two conformers that present with 35 and 41% populations. The difference in the Boltzmann populations of the lowest energy conformer and the other two conformers is large, yet the produced single-conformer spectrum aligns very well with the experiment. This shows that when selecting a conformer for generating spectra with minimal ensembles, the Boltzmann populations are not the only factor that should be taken into account; relative energies of conformers should be considered too.

**Figure 7:**
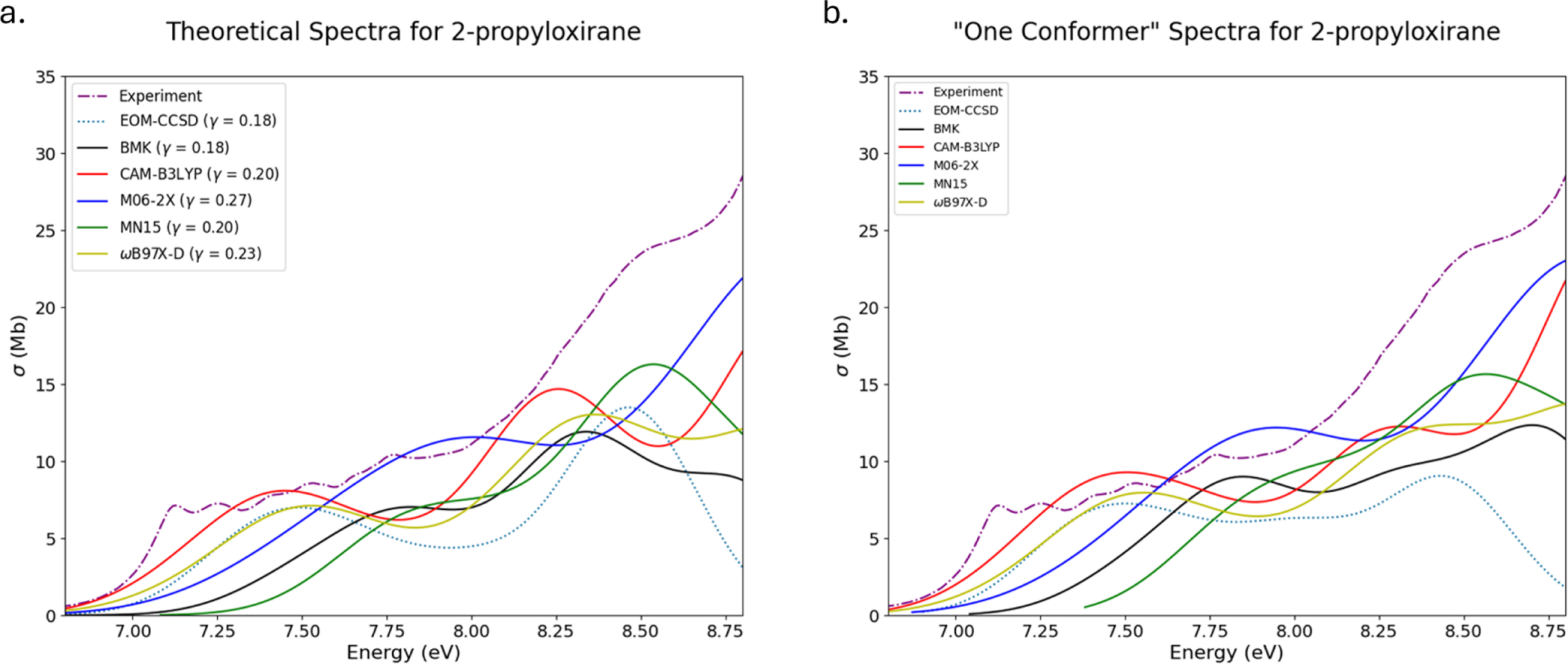
Experimental, EOM-CCSD, and TD-DFT spectra for 2-propyloxirane (OX7) overlaid together for comparison. (a) Computed from ensembles with multiple conformers. (b) Computed from ensembles with only one conformer.

Theoretical spectra for 2-propyloxirane (OX7) are presented in Figure 7a,b for multi- and single-conformer spectra, respectively. The experimental spectrum for OX7 begins with an onset described by some vibrational progressions before leading to a steady climb toward a peak outside of the selected region. Within the computational picture, the early onset region is represented by a broad peak and a shoulder on the climb toward the end of the selected region is represented similarly. Each ensemble in the multiconformer spectra contains 10 conformers, save for the TD-ωB97X-D ensemble which contains 9. The Boltzmann populations of the lowest energy conformers range from 19 to 34%. Each of these conformers has the highest Boltzmann populations for their respective method, which is the reason the differences between multi- and single-conformer spectra are small. The small differences in these spectra make it difficult to determine which set better represents the experimental spectrum by visual inspection. Turning to quantitative reasoning, the RIC values decrease for all but the CAM-B3LYP functional on going from multi- to single-conformer spectra. Additionally, MSE values improve from multi- to single-conformer spectra (except in EOM-CCSD and BMK). Values for MAE do not generally improve; however, this metric is not as useful as a differential since it does not take into account the sign for errors.

**Figure 8:**
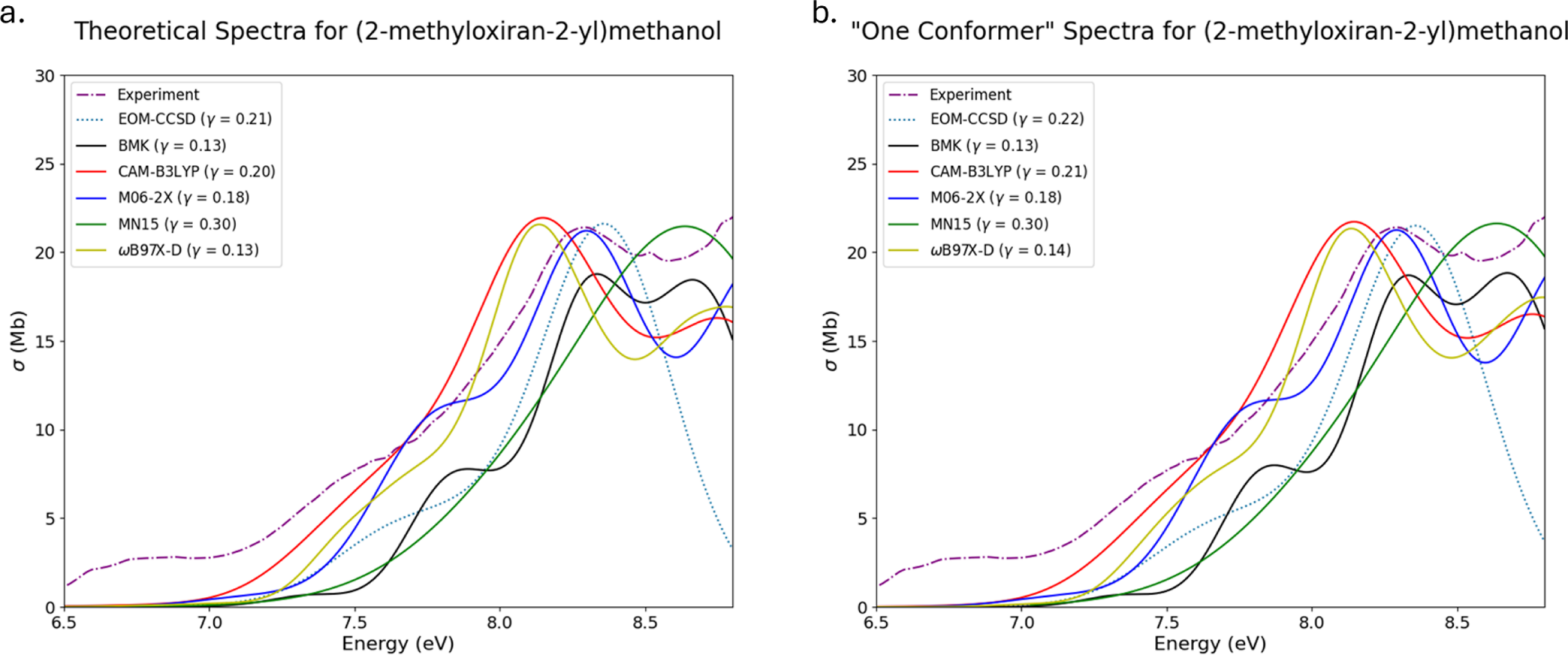
Experimental, EOM-CCSD, and TD-DFT spectra for (2-methyloxiran-2-yl)methanol (OX8) overlaid together for comparison. (a) Computed from ensembles with multiple conformers. (b) Computed from ensembles with only one conformer.

The differences in multiconformer spectra and single-conformer spectra in Figure 8a,b for (2-methyloxiran-2-yl)methanol (OX8) are minimal. The paucity of variability is a result of the large Boltzmann populations of the lowest energy conformers for all methods examined. Each ensemble used to compute multiconformer spectra contained seven conformational isomers, with the lowest energy isomer comprising at least 86% of the total Boltzmann population. In the case of this substituted oxirane, choosing a conformer for building spectra is easy; the lowest energy conformer constitutes the largest population by a significant margin, which makes this conformer wholistically representative of the system. In instances in which one conformer constitutes a large portion of the conformational population at a given temperature, especially when the conformer is the lowest relative energy, it is vital to include this conformer when computing theoretical spectra.

**Figure 9:**
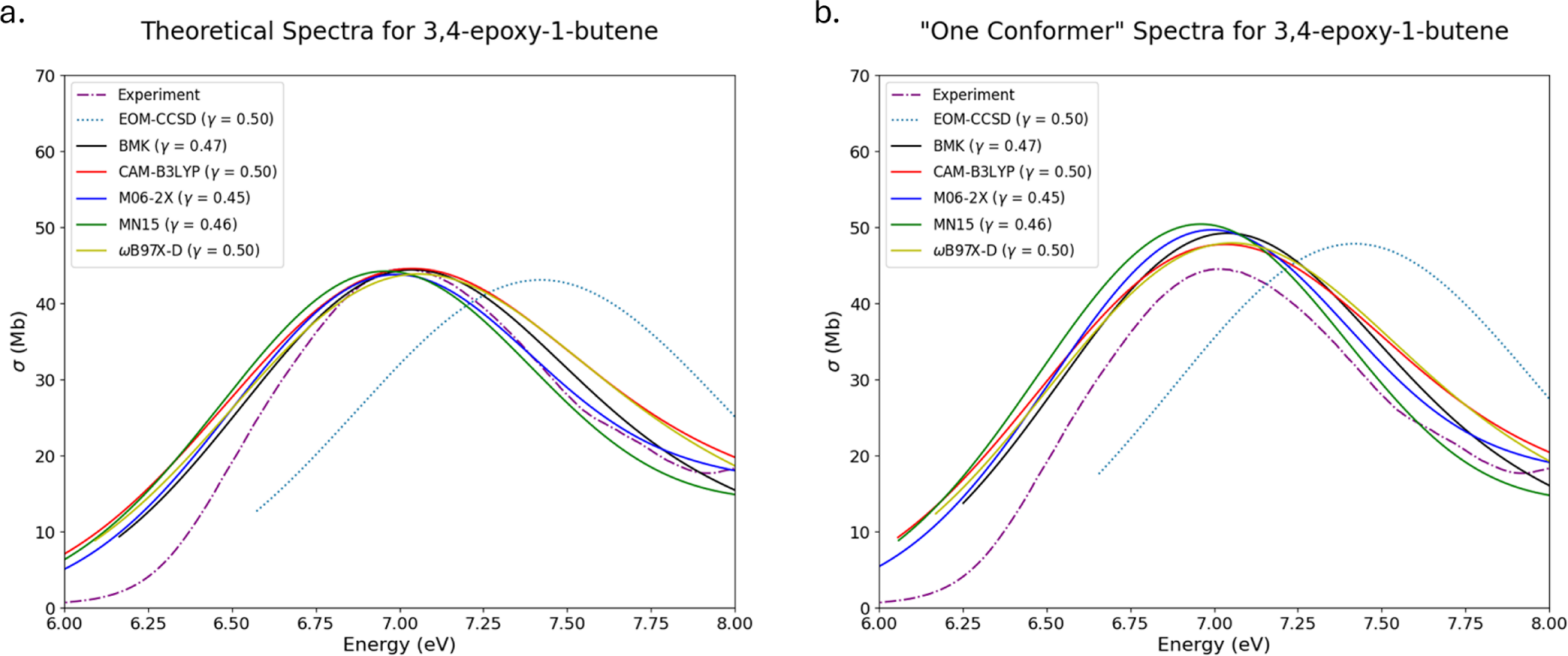
Experimental, EOM-CCSD, and TD-DFT spectra for 3,4-epoxy-1-butene (OX9) overlaid together for comparison. (a) Computed from ensembles with multiple conformers. (b) Computed from ensembles with only one conformer.

**Figure 10:**
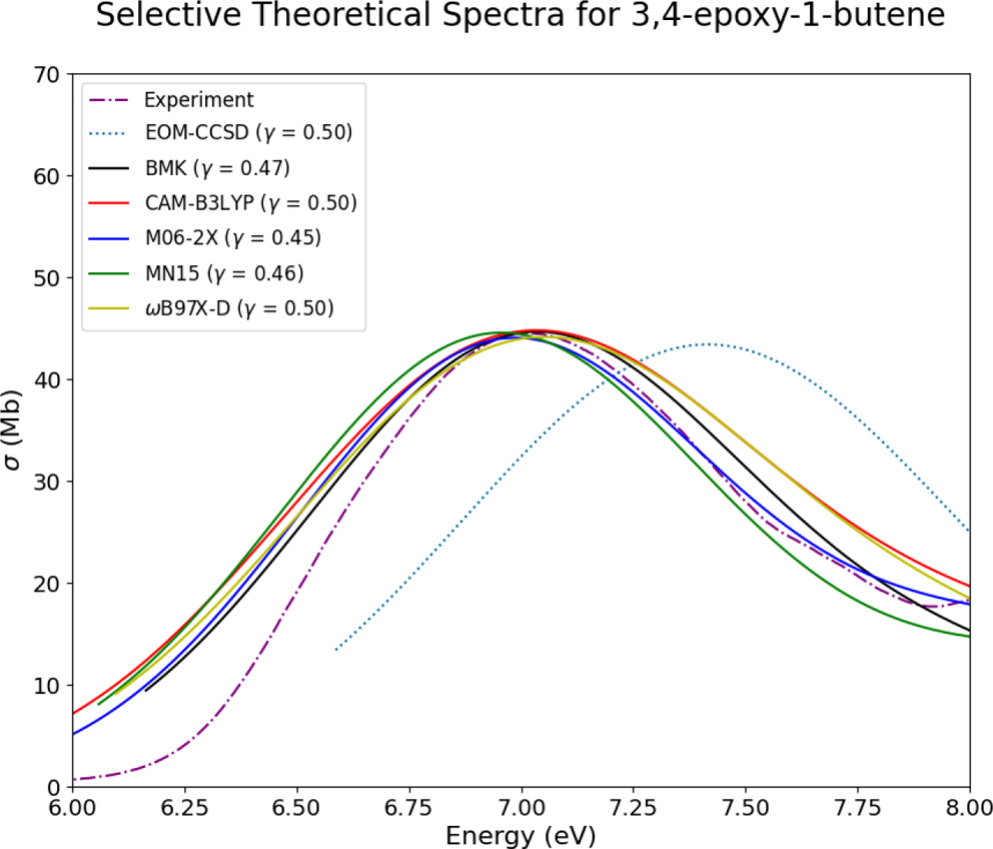
Experimental, EOM-CCSD, and TD-DFT spectra for 3,4-epoxy-1-butene (OX9) overlaid together for comparison, computed from conformers with Boltzmann populations greater than 20%.

Theoretical spectra for 3,4-epoxy-1-butene are shown in Figure 9a for multiconformer spectra, and in Figure 9b for single-conformer spectra. The multiconformer spectra are each represented by three conformers, with the lowest energy conformer consisting of 61–76% of the Boltzmann populations. The case of OX9 is one where reducing the ensemble size to one causes a decrease in the spectral quality. It is clear that this substituted oxirane system can be represented well with a reduced ensemble size; however, there is a limit to which it can be reduced. If instead of reducing the ensemble completely, spectra are generated only from conformers with Boltzmann populations of at least 20%, then the results in this case align much better with experiment than spectra generated only from the lowest energy conformer. This can be seen in Figure 10, where conformers with a Boltzmann population of less than 20% are left out, and the spectra that are produced again align well with experiment.

### 3.2 Single-Conformer Ensembles

**Figure 11:**
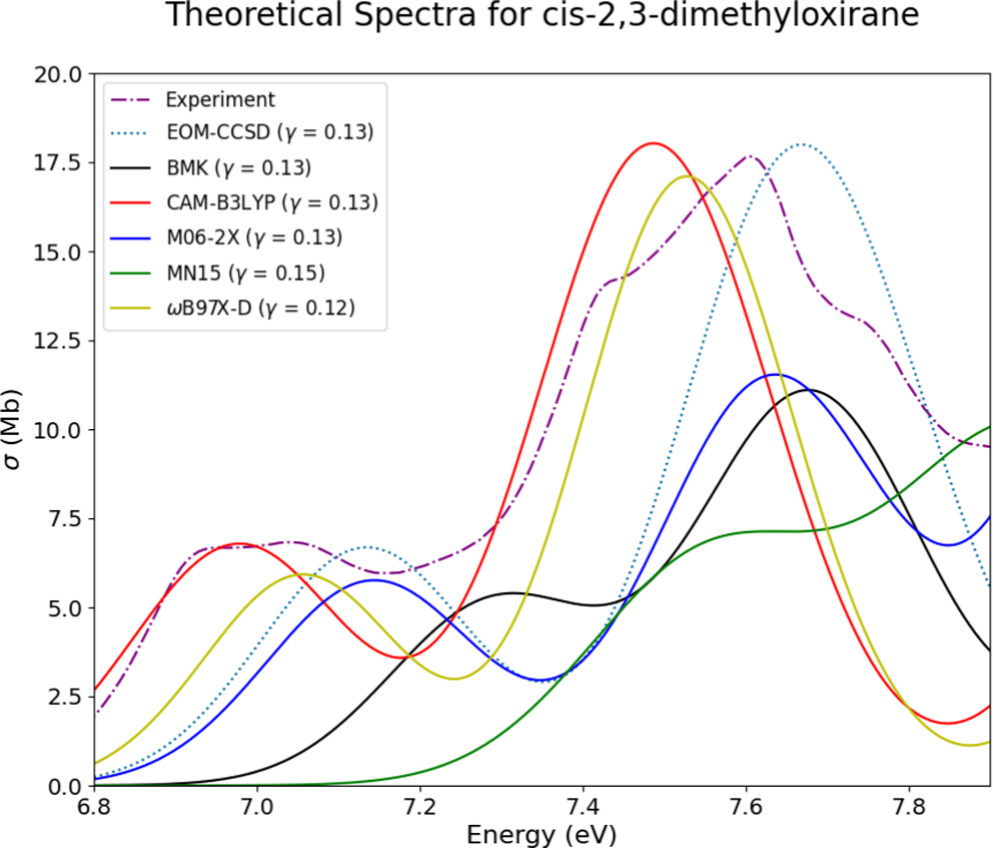
Experimental, EOM-CCSD, and TD-DFT spectra for *cis*-2,3-dimethyloxirane (OX2c) are overlaid together for comparison.

Three of the 11 selected oxiranes present with one conformer in the final ensemble after the second ensemble reduction via CREST. The first of these is *cis*-2,3-dimethyloxirane (OX2c), and spectra for this system are shown in Figure 11. The location of the locally maximum peak is mimicked well by all of the computed spectra, save for the TD-MN15 spectrum. Additionally, the height of this peak is well matched by the EOM-CCSD, TD-CAM-B3LYP, and TD-ωB97X-D methods. The other feature near the onset of the experimental spectrum is also well represented by these methods. Both of these DFT functionals present with a slight red shift compared to experiment, and the EOM-CCSD method presents with a slight blue shift. Overall, this system is well represented, with only one conformer.

**Figure 12:**
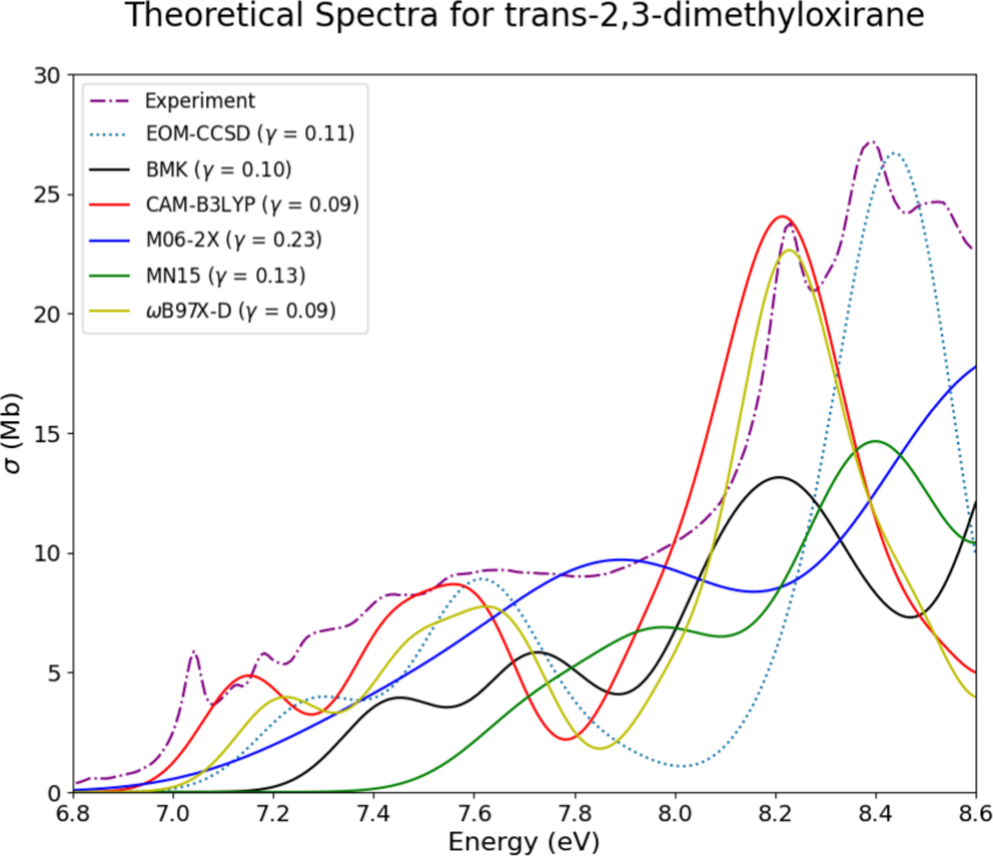
Experimental, EOM-CCSD, and TD-DFT spectra for *trans*-2,3-dimethyloxirane (OX2t) overlaid together for comparison.

The next system whose theoretical spectra are represented with only one conformer is *trans*-2,3-dimethyloxirane (OX2t). The theoretical spectra for this system, Figure 12, parallel their *cis* counterparts in regard to matching experimental features, in particular with the EOM-CCSD, TD-CAM-B3LYP, and TD-ωB97X-D methods. These three methods are able to mimic the general shape of the experimental spectrum and also present low RIC values and MSE values of less than ±5 Mb; see Table 2. Again, for the three aforementioned theoretical methods, the experimental spectrum is well mimicked.

**Figure 13:**
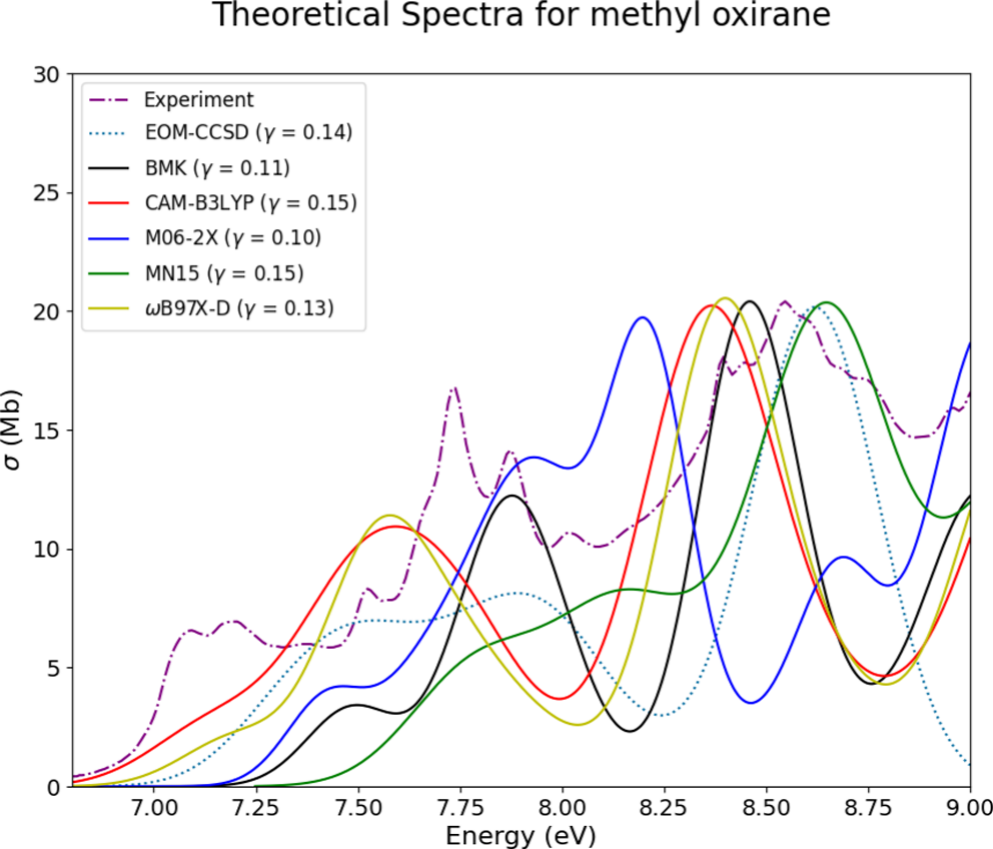
Experimental, EOM-CCSD, and TD-DFT spectra for methyl oxirane (OX10) are overlaid together for comparison.

The last oxirane with one conformer present in the final ensemble is methyl oxirane (OX10). The experimental spectrum for OX10, shown in Figure 13, is littered with a number of vibrational progressions that the vertical approximation does not capture. However, most of the theoretical methods employed can still encapsulate the general shape and location of experimental features. The locally maximum peak for the experimental spectrum is located at 8.5 eV and each of the theoretical methods matches the height of this peak as well as the location to within ca. 0.25 eV, save for TD-M06-2X. The other primary features on the experimental spectrum include a set of peaks around 7.7 eV, which are again represented by most computational methods employed, and the onset of the spectrum from 7 to 7.5 eV, which are not as well represented by theory. The vibrational progressions throughout the experimental spectrum for this molecule result in difficulties in reproducing experiments; however, the general shape is still shown in most of the theoretical methods employed with relatively low RIC values and MSE values at or below a magnitude of 5 Mb.

### 3.3 Effects of Conformational Sampling

When sampling the conformational space, it is vital to consider the Boltzmann populations of the conformational isomers. In cases where one conformational isomer is represented with a Boltzmann population of more than ca. 70%, that isomer is likely sufficient to construct theoretical spectra. This can be seen in examples such as OX8 and OX5 where the one-conformer spectra are very similar to the multiconformer spectra and their respective experimental spectra. In other instances, such as OX1 and OX6, the lowest energy conformer did not represent the majority of the Boltzmann population; however, the single-conformer spectra were arguably a better representation to experiment.

Another important consideration when examining the conformational space is the energy ordering of the conformers. For OX9, reducing the conformer-only ensemble to one is too great a reduction, and the quality of the spectra is decreased when compared to the set of multiconformer spectra. However, it was shown that the ensemble could still be reduced by limiting which conformers were utilized in generating the spectra. Limiting conformers in the multiconformer spectra to be at least 20% of the Boltzmann population resulted in theoretical spectra that were still representative of experiment. The exact population cutoff is not inflexible and should be tweaked on a case-by-case basis.

In several examples (OX2c, OX2t, and OX10) one conformer dominated the entire population and was still able to produce spectra that were likened to the experiment. In each of these examples, Section 3.2, initially, multiple conformers were obtained before the geometry optimizations. Loosening the parameters for the conformer-rotamer ensemble generation (e.g., RMSD) would allow for theoretical spectra composed of more than one conformer, if desired. Although this work focused on only the conformational space of the target molecules, it would be of interest to apply conformational selectivity to the NEA to possibly limit the ensemble size. In this way, the feasibility of high-level *ab initio* computations may be lost; however, the inclusion of non-Condon effects could better represent experiment.

## 4 Conclusions

In this research, we evaluated the importance of conformers in reproducing the experimental spectra. The use of conformational sampling with CREST resulted in, at most, 22 geometries for the oxiranes studied. Each oxirane utilized TD-DFT (functionals BMK, CAM-B3LYP, M06-2X, MN15, and ωB97X-D) and EOM-CCSD to compute the transition properties; OX3 and OX4 did not have EOM-CCSD computations. To assess the validity of this approach, quantitative metrics were explored in addition to a general qualitative examination of spectral shape, with all metrics comparing directly against our high-resolution gas-phase VUV absorption spectroscopy experiments.

In general, qualitative assessment across all methods and oxiranes gives satisfactory agreement with experiment, with some exceptions, particularly molecules with larger conformational spaces. TD-DFT methods exhibited red- and blue-shifting of the spectra compared to experiment, with the direction of the shift depending on the amount of exact Hartree–Fock exchange included in the TD-DFT functional. This is a known side effect of using vertical excitation energies.^70^ Quantitative metrics of the relative integral change (RIC), mean signed error (MSE), and mean absolute error (MAE) were examined. The general trend was MSE and MAE remaining largely unchanged between the number of conformers included, with a small exception for OX9, which peaked these metrics as a result of overreducing the ensemble.

The importance of conformational sampling is determined to be linked to both the Boltzmann population at a given temperature and the energy ordering of the conformers. We stress that conformers represented by large Boltzmann weights are often sufficient to construct theoretical absorption spectra that are representative of the experiment. In instances in which the Boltzmann population at a given temperature is not represented by one conformer, it is important to consider the low energy conformers. We encourage the use of conformer selectivity by analyzing the Boltzmann weights and relative energies of the conformers. By doing more selective conformational sampling, the total number of geometries in the nuclear ensemble approach (NEA) could possibly be reduced without a loss in accuracy.

From a careful analysis of both the qualitative and quantitative metrics alongside visual inspection of the theoretical spectra, we determined that conformational selectivity is important for matching theory to experiment. In particular, a Boltzmann weight threshold of 20% and a relative energy threshold of under 6 kcal mol^–1^ are suggested to reduce the number of conformers. By using a conformer-only ensemble, we were able to afford a high-level electronic structure method (EOM-CCSD) and the inclusion of heavily augmented basis sets (d-aug-cc-pVTZ and d-jul-cc-pVTZ) to properly characterize the high-energy and Rydberg excitations present in VUV spectroscopy. Better agreement might be possible by utilizing NEA along with our conformational selectivity parameters.

With this work, we propose two selection parameters based on the Boltzmann population and energy ordering to reduce the number of conformers per molecule. We hope this can increase the affordability of high-accuracy wave function methods when applied to the NEA. The combination of conformational selectivity and the nuclear ensemble approach could then possibly predict a priori spectra for multifunctional molecules with no experimental quantitative spectroscopy measurements.

## Supporting Information Available

Geometries and computed energies of all structures, data from experimental structures as well as data from the best functional for each substituted oxirane, and experimental spectrum of methyl oxirane which was previously unpublished (PDF)

## Dedication

Special Issue in honor of Professor Rodney J. Bartlett.
