# Supplementary material for: Evaluating the Importance of Conformers for Understanding the Vacuum-Ultraviolet Spectra of Oxiranes: Experiment and Theory
Source: J Phys Chem A. 2024 Dec 6;128(50):10906–20. doi: 10.1021/acs.jpca.4c04391 (PMC11664588; doi:10.1021/acs.jpca.4c04391)
Supplement: Supplementary file 1 — jp4c04391_si_001.pdf [file jp4c04391_si_001.pdf]

Supplementary Information: Evaluating the Importance of Conformers for Understanding the Vacuum Ultraviolet Spectra of Oxiranes: Experiment and Theory

Ian T. Beck, Erica C. Mitchell, Annabelle Webb Hill, Justin M. Turney, Brandon Rotavera, and Henry F. Schaefer III

Contents

1 Boltzmann Populations **S4**

2 1,2-epoxybutane (OX1) **S7**
2.1 BMK S7
2.2 CAM-B3LYP S10
2.3 M06-2X S14
2.4 MN15 . . . . S17
2.5 ωB97X-D S21
2.6 EOM-CCSD S24

3 *cis*-2,3-dimethyloxirane (OX2c) **S28**
3.1 BMK S28
3.2 CAM-B3LYP S29
3.3 M06-2X S30
3.4 MN15 . . . . S31
3.5 ωB97X-D S32
3.6 EOM-CCSD S33

4 *trans*-2,3-dimethyloxirane (OX2t) **S34**
4.1 BMK S34
4.2 CAM-B3LYP S35

4.3 M06-2X S36
4.4 MN15 . . . . S37
4.5 ωB97X-D S38
4.6 EOM-CCSD S39

5 1-(oxiran-2-yl)ethan-1-one (OX3) S40
5.1 BMK S40
5.2 CAM-B3LYP S42
5.3 M06-2X S44
5.4 MN15 . . . . S46
5.5 ωB97X-D S48

6 3,4-epoxybutanol (OX4) S50
6.1 BMK S50
6.2 CAM-B3LYP S62
6.3 M06-2X S74
6.4 MN15 . . . . S86
6.5 ωB97X-D S98

7 2,3-epoxybutanol (OX5) S110
7.1 BMK S110
7.2 CAM-B3LYP S114
7.3 M06-2X S118
7.4 MN15 . . . . S122
7.5 ωB97X-D S126
7.6 EOM-CCSD S130

8 2,3-epoxypentane (OX6) S134
8.1 BMK S134
8.2 CAM-B3LYP S138
8.3 M06-2X S142
8.4 MN15 . . . . S146
8.5 ωB97X-D S150
8.6 EOM-CCSD S154

9 2-propyloxirane (OX7) S158
9.1 BMK S158
9.2 CAM-B3LYP S164

9.3 M06-2X S170
9.4 MN15 . . . . S176
9.5 ωB97X-D S182
9.6 EOM-CCSD S188

10 (2-methyloxiran-2-yl)methanol (OX8) S194
10.1 BMK S194
10.2 CAM-B3LYP S198
10.3 M06-2X S202
10.4 MN15 . . . . S206
10.5 ωB97X-D S210
10.6 EOM-CCSD S214

11 3,4-epoxy-1-butene (OX9) S218
11.1 BMK S218
11.2 CAM-B3LYP S220
11.3 M06-2X S221
11.4 MN15 . . . . S223
11.5 ωB97X-D S224
11.6 EOM-CCSD S226

12 methyl oxirane (OX10) S227
12.1 BMK S227
12.2 CAM-B3LYP S228
12.3 M06-2X S229
12.4 MN15 . . . . S230
12.5 ωB97X-D S231
12.6 EOM-CCSD S232

Geometries for all structures are given in Cartesian coordinates with distances in angstroms and angles in degrees. Energies are given in Eh.

1 Boltzmann Populations

		EOM-CCSD	BMK	CAM-B3LYP	M06-2X	MN15	ωB97X-D
OX1	STRUC1	0.4282879	x	x	0.4041539	x	0.4609903
	STRUC2	x	x	x	0.3679207	0.3302285	0.3698343
	STRUC3	x	0.1778802	x	0.2279254	x	0.1691754
	STRUC4	x	0.4350013	0.4535468	x	0.3555507	x
	STRUC5	x	x	x	x	x	x
	STRUC6	0.1580774	x	0.1160139	x	0.3142208	x
	STRUC7	0.4136347	0.3871186	0.4304393	x	x	x
	Energy Ordering	7, 1, 6	7, 4, 3	7, 4, 6	2, 1, 3	2, 6, 4	2, 1, 3
OX2c	STRUC1	x	x	1	1	x	1
	STRUC2	1	1	x	x	1	x
OX2t	STRUC1	1	1	x	1	1	1
	STRUC2	x	x	1	x	x	x
OX3	STRUC1	x	0.9022138	0.9007618	0.929969	0.9538664	0.948286
	STRUC2	x	0.0945079	x	0.0557459	0.0332434	0.0382792
	STRUC3	x	0.0032782	0.005577	0.0031906	0.0026815	0.003451
	STRUC4	x	x	0.0936613	0.0110945	0.0102088	0.0099838
	Energy Ordering	x	1, 2, 3	1, 4, 3	1, 2, 4, 3	1, 2, 4, 3	1, 2, 4, 3

		EOM-CCSD	BMK	CAM-B3LYP	M06-2X	MN15	ωB97X-D
OX4	STRUC1	x	0.3251049	0.3046153	0.2996454	0.2567064	0.2481568
	STRUC2	x	0.3625592	0.283052	0.3888359	0.4862693	0.3707953
	STRUC3	x	0.0142946	0.0191725	0.0131866	0.0098799	0.0204232
	STRUC4	x	0.0104091	0.0145497	0.01113	0.0096053	0.0182649
	STRUC5	x	0.0175057	0.0164602	0.0225234	0.0178656	0.0298994
	STRUC6	x	0.030284	0.0382989	0.0315237	0.0243267	0.0404315
	STRUC7	x	0.018901	0.0290302	0.0154257	0.0105345	0.0245859
	STRUC8	x	0.0219687	0.030391	0.0203305	0.0156896	0.0274564
	STRUC9	x	x	0.0193872	x	x	x
	STRUC10	x	0.0122143	0.0187446	0.0100784	0.0069573	0.016576
	STRUC12	x	0.0052933	0.0064677	0.0054802	0.0043181	0.0089575
	STRUC13	x	0.0135491		0.011321	0.0077214	0.0209710
	STRUC14	x	0.0167955	0.0124746	0.0230325	0.0247626	0.0164953
	STRUC15	x	0.0043407	0.0037631	0.0047407	0.0051765	0.0054571
	STRUC16	x	0.0616851	0.0904326	0.0548083	0.0420083	0.0571445
	STRUC17	x	0.0207049	0.0292589	0.018927	0.0140053	0.0230052
	STRUC18	x	x	x	x	x	x
	STRUC19	x	0.0231483	0.041337	0.0189624	0.0126705	0.0265485
	STRUC20	x	0.0026893	0.0023518	0.002882	0.0031243	0.0035324
	STRUC21	x	0.0216259	0.0190508	0.0280886	0.0295062	0.0194192
	STRUC22	x	0.0073399	0.012086	0.0061037	0.004006	0.0098414
	STRUC23	x	0.0038059	0.0023525	0.0068392	0.0086465	0.0053007
	STRUC24	x	0.0047184	0.0047838	0.0050334	0.0054292	0.0051871
	STRUC25	x	0.0010626	0.0019398	0.0011011	0.0007903	0.0015506
	Energy Ordering	x	2, 1, 16, 6, 19, 8, 21, 17, 7, 5, 14, 3, 10, 4, 22, 13, 12, 24, 15, 23, 20, 25	1, 2, 16, 19, 6, 8, 17, 7, 3, 21, 10, 5, 4, 14, 22, 9, 12, 24, 15, 23, 20, 25	2, 1, 16, 6, 21, 14, 5, 8, 19, 17, 7, 3, 4, 10, 23, 22, 13, 12, 24, 15, 20, 25	2, 1, 16, 21, 14, 6, 5, 8, 17, 19, 7, 3, 4, 23, 10, 24, 15, 12, 22, 13, 20, 25	2, 1, 16, 21, 14, 6, 5, 8, 17, 19, 7, 3, 4, 23, 10, 24, 15, 12, 22, 13, 20, 25

		EOM-CCSD	BMK	CAM-B3LYP	M06-2X	MN15	ωB97X-D
OX5	STRUC1	0.7197701	0.763779	0.6477807	0.7873999	0.8665876	0.6699192
	STRUC2	0.2157721	0.1859403	0.2675543	0.1704762	0.1094399	0.2483102
	STRUC3	0.0212022	0.0176584	0.0242253	0.015012	0.0080001	0.0284347
	STRUC4	0.0190472	0.0136576	x	0.0109634	x	0.0229958
	STRUC5	x	x	0.0243056	x	0.0063041	x
	STRUC6	0.0131182	0.0099032	0.0184033	0.0081628	0.004764	0.014255
	STRUC7	0.0043471	0.0035802	0.006456	0.0032064	0.0019266	0.0068401
	STRUC9	0.0067431	0.0054812	0.0112746	0.0047793	0.0029778	0.0092449
	Energy Ordering	1, 2, 3, 6, 4, 9, 7	1, 2, 3, 6, 4, 9, 7	1, 2, 3, 6, 5, 9, 7	1, 2, 3, 6, 4, 9, 7	1, 2, 3, 6, 5, 9, 7	1, 2, 3, 6, 4, 9, 7
OX6	STRUC1	0.3973239	x	0.3585461	0.3466259	0.2739058	0.4293835
	STRUC2	0.2843268	0.2723557	0.5134268	0.2410593	0.2006297	0.2453227
	STRUC3	x	0.3391101	x	x	x	x
	STRUC4	x	0.3885342	x	x	x	x
	STRUC5	x	x	x	0.4123148	x	0.3252937
	STRUC6	0.3183492	x	0.1280271	x	0.5254645	x
	Energy Ordering	2, 1, 6	2, 4, 3	2, 1, 6	2, 1, 5	2, 6, 1	2, 1, 5
OX7	STRUC1	0.1117603	0.1013308	0.0796256	0.1322471	0.1444887	0.1460118
	STRUC2	0.1696706	0.1857952	0.21826	0.1510404	0.1257607	0.1791558
	STRUC3	0.2685287	0.2756248	0.3456822	0.2280971	0.1912598	0.2377267
	STRUC4	0.1496965	0.129505	0.1098064	0.1525971	0.1422077	0.1183379
	STRUC5	0.0608178	0.0611749	0.0527956	0.0596352	0.0587884	0.0709805
	STRUC6	0.119713	0.1099002	0.1017859	0.1058238	0.1045899	0.1127116
	STRUC7	0.0816577	0.0970963	0.0695651	0.1105598	0.148017	0.086358
	STRUC8	0.0112732	0.0115485	0.006825	0.0141484	0.0208622	x
	STRUC10	0.0106861	0.010065	0.0066851	0.0166428	0.0196672	0.0281285
	STRUC11	0.0161961	0.0179591	0.0089693	0.0292083	0.0443584	0.0205891
	Energy Ordering	3, 2, 4, 6, 1, 7, 5, 11, 8, 10	3, 2, 4, 6, 1, 7, 5, 11, 8, 10	3, 2, 4, 6, 1, 7, 5, 11, 8, 10	3, 4, 2, 1, 7, 6, 5, 11, 10, 8	3, 7, 1, 4, 2, 6, 5, 11, 8, 10	3, 2, 1, 4, 6, 7, 5, 11, 10
OX8	STRUC1	x	x	x	x	x	0.8671587
	STRUC2	0.090168	0.0721685	0.0924894	0.0713516	0.0463253	0.0993881
	STRUC3	0.0110444	0.0086406	0.0116361	0.0082292	0.0044149	0.0147328
	STRUC5	0.0043663	0.0029609	0.004806	0.0028549	0.0017788	0.0057559
	STRUC6	0.007496	0.0051614	0.0092106	0.0046855	0.0027614	0.0070502
	STRUC8	0.0016741	0.0012772	0.0018852	0.0013897	0.0009226	0.0026799
	STRUC9	0.0030898	0.002332	0.0039754	0.0022453	0.0014334	0.0032344
	STRUC10	0.8821614	0.9074594	0.8759973	0.9092438	0.9423636	x
	Energy Ordering	10, 2, 3, 6, 5, 9, 8	10, 2, 3, 6, 5, 9, 8	10, 2, 3, 6, 5, 9, 8	10, 2, 3, 6, 5, 9, 8	10, 2, 3, 6, 5, 9, 8	1, 2, 3, 6, 5, 9, 8
OX9	STRUC1	0.6727635	0.6784073	0.7643452	0.6132077	0.6237162	0.7138
	STRUC2	0.305732	0.3107034	0.2222664	0.3738066	0.3611589	0.2688265
	STRUC3	0.0215045	0.0108893	0.0133884	0.0129857	0.0151249	0.0173734
	Energy Ordering	1, 2, 3	1, 2, 3	1, 2, 3	1, 2, 3	1, 2, 3	1, 2, 3
OX10	STRUC1	x	1	1	1	x	x
	STRUC2	1	x	x	x	1	1

2 1,2-epoxybutane (OX1)

2.1 BMK

OX1 1,2-epoxybutane BMK STRUC1

C	-2.1552260254	-0.3640494508	0.0624018715
C	-0.9542436994	0.5326754897	-0.2785194723
C	1.2799494601	-0.8241018346	-0.1757107284
O	1.5355082230	0.5685734413	-0.0370421446
H	0.2249330219	0.0748103827	1.5378392715
H	-3.0544459924	-0.0234090708	-0.4520583362
H	-1.9644566855	-1.3977780713	-0.2332001528
H	-2.3586351938	-0.3533680140	1.1355835938
C	0.2926592275	0.0858814854	0.4514184448
H	-0.7526426445	0.5177619284	-1.3522321557
H	-1.1706615638	1.5692458586	-0.0054794682
H	1.1103236103	-1.1594595787	-1.1946357311
H	1.8950967396	-1.4694146792	0.4425393964

Energy of Optimized Geometry: -232.350425328180

OX1 1,2-epoxybutane BMK STRUC2

C	-2.0331064092	0.5474239164	0.1354031888
C	-0.9912840653	-0.4283818856	-0.4353550228
C	1.6216030232	-0.5457057982	-0.2511720956
O	1.1331892993	0.7210608722	0.1747679279
H	0.1892041355	-0.6935807452	1.4179028138
H	-1.6362537615	1.5638448728	0.1397945826
H	-2.9472086994	0.5374831937	-0.4597585258
H	-2.2951206664	0.2802742877	1.1617109071
C	0.2863589705	-0.4055743972	0.3725577351
H	-1.3882977658	-1.4477416806	-0.4381278492
H	-0.7540957795	-0.1641241110	-1.4687871925
H	1.6881743832	-0.6735793167	-1.3272834126
H	2.4521842987	-0.9370185516	0.3270119215

Energy of Optimized Geometry: -232.350721125818

OX1 1,2-epoxybutane BMK STRUC3

C	-1.7538328301	-0.3143292723	-0.5214095868
C	-1.0388987307	0.5429509380	0.5314911381
C	1.1595497242	-0.8792966082	0.1199037544
O	1.1817701138	0.3352557150	-0.6211121194
H	0.9601325665	0.9010750467	1.3904408235
H	-1.7458460016	-1.3705472119	-0.2472589879
H	-2.7948184440	-0.0046068412	-0.6193112349
H	-1.2718653066	-0.2052961577	-1.4942600919
C	0.4629420194	0.3357493701	0.6051640242
H	-1.2113996110	1.6029414482	0.3209526552
H	-1.4545095366	0.3513520258	1.5250420137
H	0.5889646071	-1.6812482999	-0.3354404891
H	2.1076374542	-1.1646566561	0.5640382464

Energy of Optimized Geometry: -232.349924752853

OX1 1,2-epoxybutane BMK STRUC4

C	-2.1768302498	0.0010867431	0.2110683494
C	-0.8785361327	0.6316462393	-0.3177587557
C	1.1251683604	-1.0359018107	-0.0982937920
O	1.5983917748	0.3023398812	-0.1918112005
H	0.3359779705	0.2652460130	1.4957422152
H	-2.3038763014	0.2039772042	1.2768548446
H	-3.0458839257	0.4018374934	-0.3118887625
H	-2.1663999353	-1.0822095110	0.0740347437
C	0.3317808148	0.1033195223	0.4192547026
H	-0.7542454536	0.4251635481	-1.3834472101
H	-0.9152778307	1.7184634755	-0.2021007611
H	0.8390978321	-1.4881708919	-1.0433738240
H	1.6750501075	-1.6724063305	0.5870795149

Energy of Optimized Geometry: -232.350425401661

OX1 1,2-epoxybutane BMK STRUC5

C	-2.1265213068	-0.5103380326	0.0091708054
C	-0.9857173245	0.4856008683	-0.2542111334
C	1.3343518888	-0.7228545147	-0.2136088926
O	1.4940818281	0.6708645594	0.0211252166
H	0.2063684173	-0.0147748547	1.5422662711
H	-1.8642454829	-1.5062934255	-0.3538583574
H	-2.3391187652	-0.5871833848	1.0778796040
H	-3.0422412414	-0.1954937676	-0.4925358766
C	0.2823336549	0.0740720733	0.4599621693
H	-0.7744548590	0.5579690060	-1.3237512700
H	-1.2737224257	1.4843539376	0.0856030698
H	1.1964872516	-0.9998726114	-1.2546921643
H	1.9860586675	-1.3663297305	0.3681831506

Energy of Optimized Geometry: -232.350425327372

OX1 1,2-epoxybutane BMK STRUC6

C	-1.7358378009	-0.1545252044	-0.6396881070
C	-0.9660074056	0.8037424479	0.2790604264
C	0.9751389196	-0.9939463904	0.4389243144
O	1.2666473921	-0.0029348847	-0.5388492772
H	0.9924586097	1.0410657400	1.2644531696
H	-1.1682017496	-0.3481622112	-1.5512551852
H	-1.9333687765	-1.1072149040	-0.1454888907
H	-2.6960481328	0.2791720059	-0.9209680359
C	0.4660282794	0.3919818977	0.5680831296
H	-0.9337413646	1.7997835379	-0.1731501654
H	-1.4845436323	0.9129615876	1.2360194737
H	0.3102335538	-1.7832830303	0.1056061023
H	1.8212752576	-1.3103743844	1.0403794864

Energy of Optimized Geometry: -232.349924703956

OX1 1,2-epoxybutane BMK STRUC7

C	-2.0186543965	0.5734284486	0.2219693524
C	-1.0203008913	-0.4685558559	-0.3082504164
C	1.5991710868	-0.5826645782	-0.3053335284
O	1.1424080688	0.7284991198	0.0048003294
H	0.2950595266	-0.5252309372	1.4708322987
H	-2.2034707516	0.4275099376	1.2887707596
H	-1.6240479528	1.5808973223	0.0802815341
H	-2.9743963890	0.5016213080	-0.2990593805
C	0.3139346465	-0.3616859574	0.3947113083
H	-1.4146538809	-1.4787216941	-0.1641828829
H	-0.8611904079	-0.3264202580	-1.3799779954
H	1.5861756187	-0.8355753176	-1.3611763138
H	2.4709769292	-0.9103700002	0.2514552032

Energy of Optimized Geometry: -232.350721279305

2.2 CAM-B3LYP

OX1 1,2-epoxybutane CAM-B3LYP STRUC1

C	-2.1576610378	-0.2971700705	0.0598239592
C	-0.9325961507	0.5373946737	-0.2897283276
C	1.2487484378	-0.8539504007	-0.1475241781
O	1.5480069511	0.5337603913	-0.0432645885
H	0.2184607818	0.0908878970	1.5276743948
H	-3.0398103017	0.0644142871	-0.4658557494
H	-2.0128333876	-1.3432959746	-0.2102856181
H	-2.3715362335	-0.2576735108	1.1283858547
C	0.2926814512	0.0766602485	0.4431594135
H	-0.7318990341	0.4970447403	-1.3611077317
H	-1.1129221048	1.5858667232	-0.0436216142
H	1.0815360004	-1.2117851847	-1.1574659996
H	1.8426319211	-1.5018762428	0.4862707073

Energy of Optimized Geometry: -232.391301873584

OX1 1,2-epoxybutane CAM-B3LYP STRUC2

C	-2.0306951094	0.5387700366	0.1166703057
C	-0.9805759945	-0.4145288265	-0.4377632930
C	1.6017177294	-0.5596116022	-0.2312019674
O	1.1431290124	0.7236177983	0.1800556623
H	0.1724662023	-0.6784493657	1.4133729647
H	-1.6548327688	1.5609514880	0.1264390397
H	-2.9381108239	0.5178869832	-0.4850490431
H	-2.3033965025	0.2727321205	1.1385514985
C	0.2832573400	-0.3969039797	0.3691445894
H	-1.3689961694	-1.4358731482	-0.4515592486
H	-0.7386658151	-0.1499633443	-1.4681555172
H	1.6810941031	-0.6960290571	-1.3038286965
H	2.4187687305	-0.9658468888	0.3533550019

Energy of Optimized Geometry: -232.391663650016

OX1 1,2-epoxybutane CAM-B3LYP STRUC3

C	-1.7464338844	-0.3305882942	-0.5277621846
C	-1.0420501453	0.5020507236	0.5317377422
C	1.1688544712	-0.8492979922	0.1184741773
O	1.1805137271	0.3706117217	-0.6153726417
H	0.9257972034	0.8880397453	1.4037864614
H	-1.7111449122	-1.3941241234	-0.2942278150
H	-2.7950986760	-0.0469177615	-0.5992018107
H	-1.2908001756	-0.1811149596	-1.5053143197
C	0.4506469362	0.3242278392	0.6057668484
H	-1.2345102384	1.5625248036	0.3525938476
H	-1.4524912274	0.2831519112	1.5203355926
H	0.6262808598	-1.6594694595	-0.3521680514
H	2.1152334578	-1.1236205413	0.5698563808

Energy of Optimized Geometry: -232.390320778071

OX1 1,2-epoxybutane CAM-B3LYP STRUC4

C	-2.1657185095	0.2370599547	0.0575780292
C	-0.7778475377	0.7217853847	-0.3402171595
C	1.0064888006	-1.1377808240	-0.0695982656
O	1.6305359654	0.1412742324	-0.0645159231
H	0.2430164527	0.1485197727	1.5188164512
H	-2.3560663513	0.4088523814	1.1174902399
H	-2.9381689668	0.7594391658	-0.5043428820
H	-2.2798386388	-0.8306335612	-0.1303857668
C	0.3051033025	0.0361880541	0.4392778408
H	-0.6008601220	0.5528623465	-1.4031814172
H	-0.6975779216	1.7983234462	-0.1761212578
H	0.7522658968	-1.5196445596	-1.0522433536
H	1.4310747998	-1.8601743787	0.6175068380

Energy of Optimized Geometry: -232.391301990207

OX1 1,2-epoxybutane CAM-B3LYP STRUC5

C	-2.1411633864	-0.3978027802	0.0829717462
C	-0.9632526732	0.5324931314	-0.1747819616
C	1.2756484354	-0.7648064770	-0.3163575732
O	1.5230400143	0.6025357712	-0.0099413498
H	0.2723995064	-0.1334347509	1.5153341254
H	-1.9666042161	-1.3841591281	-0.3470904514
H	-2.3156622078	-0.5285873146	1.1514032325
H	-3.0558777542	-0.0003245839	-0.3535886357
C	0.3059148775	0.0207941204	0.4397150793
H	-0.8027261793	0.6628692167	-1.2457493912
H	-1.1739377807	1.5230179039	0.2336632873
H	1.0849771304	-0.9725791718	-1.3635412810
H	1.9180279984	-1.4743893791	0.1917173649

Energy of Optimized Geometry: -232.391301925340

OX1 1,2-epoxybutane CAM-B3LYP STRUC6

C	-1.7240070675	-0.1017320108	-0.6744417888
C	-0.9555299190	0.7906089502	0.2872637430
C	0.9498659052	-1.0078165665	0.4292937345
O	1.2804137667	0.0010938439	-0.5188556772
H	0.9664932237	0.9792309441	1.3127710867
H	-1.1639791329	-0.2526454451	-1.5959434828
H	-1.9331851447	-1.0782699983	-0.2390666623
H	-2.6795620906	0.3515551478	-0.9327944460
C	0.4542806117	0.3562348078	0.5847695737
H	-0.9049971304	1.8052729954	-0.1148233996
H	-1.4871752708	0.8687418347	1.2385481822
H	0.2922425041	-1.7842469095	0.0590917854
H	1.7749232190	-1.3510659918	1.0426176342

Energy of Optimized Geometry: -232.390320786897

OX1 1,2-epoxybutane CAM-B3LYP STRUC7

C	-2.0547407761	0.4361633361	0.1288886831
C	-0.9682484481	-0.5254209053	-0.3332442980
C	1.6237543997	-0.4973785634	-0.2197200352
O	1.1030361203	0.8038405014	0.0273476207
H	0.2625412667	-0.4600082642	1.4852200976
H	-2.2743822396	0.3017631486	1.1886718415
H	-1.7423681372	1.4690261693	-0.0194621435
H	-2.9798540590	0.2801655997	-0.4241518250
C	0.3194545350	-0.3240716917	0.4080704041
H	-1.2937188137	-1.5585815705	-0.1891962802
H	-0.7791611618	-0.3951190466	-1.3997560250
H	1.6740504900	-0.7802896321	-1.2651593906
H	2.4836678955	-0.7708464487	0.3803788619

Energy of Optimized Geometry: -232.391663681071

2.3 M06-2X

OX1 1,2-epoxybutane M06-2X STRUC1

C	-2.1324741766	-0.3698355991	0.0384322817
C	-0.9459124369	0.5340756964	-0.2772447592
C	1.2561206195	-0.8204738528	-0.1601687525
O	1.5315412049	0.5658332143	-0.0316298643
H	0.2173201498	0.0850362432	1.5392465735
H	-3.0293227060	-0.0384779856	-0.4821535661
H	-1.9266660333	-1.3978740555	-0.2613827941
H	-2.3490243007	-0.3718472957	1.1074147116
C	0.2887072866	0.0914776658	0.4545384057
H	-0.7299350719	0.5309723047	-1.3469459052
H	-1.1726134258	1.5656213898	-0.0001748935
H	1.0804395138	-1.1600366833	-1.1751431893
H	1.8629523470	-1.4691236351	0.4596181886

Energy of Optimized Geometry: -232.405559453892

OX1 1,2-epoxybutane M06-2X STRUC2

C	-2.0216117486	0.5151654138	0.1234050015
C	-0.9753157120	-0.4409831104	-0.4394156025
C	1.6140035824	-0.5183507314	-0.2425133454
O	1.1116892306	0.7379527846	0.1850886154
H	0.1915507723	-0.7007853879	1.4107537890
H	-1.6353157226	1.5338977970	0.1322484078
H	-2.9330018539	0.4994418591	-0.4723247247
H	-2.2842157377	0.2454243739	1.1471411293
C	0.2893390653	-0.4050391059	0.3692074691
H	-1.3594147158	-1.4633903698	-0.4494734673
H	-0.7335643117	-0.1725866377	-1.4693607936
H	1.6852912854	-0.6409018972	-1.3173601164
H	2.4464824793	-0.9015773259	0.3350439966

Energy of Optimized Geometry: -232.405878392861

OX1 1,2-epoxybutane M06-2X STRUC3

C	-1.7266645020	-0.3522693804	-0.5183659656
C	-1.0429114849	0.5023376323	0.5400631652
C	1.1614094371	-0.8456579784	0.0909584867
O	1.1666827326	0.3877436154	-0.6108808093
H	0.9372718028	0.8711636799	1.4170069653
H	-1.7083819483	-1.4082899291	-0.2501569213
H	-2.7686996954	-0.0587696046	-0.6328141628
H	-1.2331292783	-0.2336456926	-1.4828306333
C	0.4521104836	0.3257344365	0.6127590632
H	-1.2363922641	1.5595548453	0.3431213004
H	-1.4575986398	0.2913865740	1.5280702198
H	0.6037909616	-1.6399842305	-0.3897089179
H	2.1119989828	-1.1313846218	0.5250447515

Energy of Optimized Geometry: -232.405388001850

OX1 1,2-epoxybutane M06-2X STRUC4

C	-2.1625617826	0.0792355124	0.0706820287
C	-0.8244799320	0.6661060043	-0.3649946245
C	1.0627080365	-1.0715719024	-0.0208010639
O	1.6131255150	0.2333087816	-0.1001754282
H	0.2609049971	0.2686431787	1.5111259662
H	-2.3532569852	0.2814411510	1.1254091595
H	-2.9844524096	0.5035933142	-0.5034464108
H	-2.1747346412	-1.0022585337	-0.0682891187
C	0.3099428484	0.0980324150	0.4387385715
H	-0.6353331511	0.4587870298	-1.4195382139
H	-0.8327406505	1.7517686729	-0.2494543404
H	0.8018851891	-1.5182094165	-0.9741970933
H	1.5385301507	-1.7294309265	0.6962661471

Energy of Optimized Geometry: -232.405558987879

OX1 1,2-epoxybutane M06-2X STRUC5

C	-2.1383895720	-0.3333379166	0.0693405287
C	-0.9426285528	0.5808722360	-0.1741661765
C	1.2327470288	-0.8154823433	-0.3074133415
O	1.5414794979	0.5383247430	-0.0152173780
H	0.2699163133	-0.1153274084	1.5283247246
H	-1.9667824536	-1.3192689862	-0.3638488811
H	-2.3186063081	-0.4655501457	1.1368455705
H	-3.0444527968	0.0774423149	-0.3728487737
C	0.3056789454	0.0276048351	0.4512516569
H	-0.7632415594	0.7081185102	-1.2430403879
H	-1.1359294636	1.5735498946	0.2374629901
H	1.0165914658	-1.0212654120	-1.3501441362
H	1.8454870143	-1.5475293115	0.2042425766

Energy of Optimized Geometry: -232.405559382950

OX1 1,2-epoxybutane M06-2X STRUC6

C	-1.7214984196	-0.1543946819	-0.6206984553
C	-0.9595477791	0.7874912970	0.3014934221
C	0.9643791352	-0.9917281001	0.3969283665
O	1.2594224461	0.0266363535	-0.5454946066
H	0.9890529112	0.9958928780	1.2946750274
H	-1.1480595714	-0.3490782001	-1.5268142184
H	-1.9292296011	-1.1067684606	-0.1335897302
H	-2.6752653599	0.2829778483	-0.9107361109
C	0.4631706123	0.3736627865	0.5763610276
H	-0.9286213754	1.7883530137	-0.1359071014
H	-1.4755187793	0.8847662559	1.2591029986
H	0.2968127974	-1.7653013100	0.0375027348
H	1.8080483198	-1.3325554174	0.9850912316

Energy of Optimized Geometry: -232.405387809683

OX1 1,2-epoxybutane M06-2X STRUC7

C	-2.0089868945	0.4775798151	0.3212765779
C	-1.0053735892	-0.4815054345	-0.3104322451
C	1.5912554545	-0.4945493699	-0.3952029865
O	1.1053178533	0.7690805697	0.0305284205
H	0.3617023068	-0.6318026892	1.4101349432
H	-2.1526302157	0.2496791646	1.3781357140
H	-1.6502426123	1.5033825507	0.2439018567
H	-2.9786029103	0.4158104619	-0.1703662677
C	0.3382675625	-0.3815646141	0.3524531006
H	-1.3616681054	-1.5110977808	-0.2336523722
H	-0.8837701397	-0.2560879417	-1.3713829601
H	1.5487866602	-0.6642207622	-1.4651342877
H	2.4912024433	-0.8330489601	0.1037491415

Energy of Optimized Geometry: -232.405878336220

2.4 MN15

OX1 1,2-epoxybutane MN15 STRUC1

C	-2.1270436143	-0.3560496713	0.0427814608
C	-0.9403258151	0.5459715406	-0.2721422308
C	1.2390040241	-0.8299192391	-0.1662144255
O	1.5353744929	0.5492198110	-0.0374829268
H	0.2223350672	0.0873837007	1.5432916801
H	-3.0260345482	-0.0253237972	-0.4744003260
H	-1.9222690296	-1.3841198270	-0.2584643507
H	-2.3410705645	-0.3604819101	1.1124595941
C	0.2913701228	0.0946350696	0.4581379754
H	-0.7192385098	0.5406876537	-1.3413908348
H	-1.1641787370	1.5785482971	0.0029688600
H	1.0438591171	-1.1667807862	-1.1792879651
H	1.8398293269	-1.4928948116	0.4447802896

Energy of Optimized Geometry: -232.227168789808

OX1 1,2-epoxybutane MN15 STRUC2

C	-2.0144784603	0.5116572279	0.1236380702
C	-0.9713409734	-0.4442225517	-0.4416344099
C	1.6115867137	-0.5076967891	-0.2482312139
O	1.1022455382	0.7385276679	0.1914240753
H	0.1932519298	-0.7168231623	1.4095929289
H	-1.6209318000	1.5275378007	0.1439640380
H	-2.9245923848	0.5091586365	-0.4741975130
H	-2.2823969294	0.2348296469	1.1443332852
C	0.2901866614	-0.4098797401	0.3709433552
H	-1.3570768261	-1.4659768114	-0.4622463006
H	-0.7203784646	-0.1692307515	-1.4682956934
H	1.6748552545	-0.6261832709	-1.3247058616
H	2.4513833556	-0.8917887135	0.3187316247

Energy of Optimized Geometry: -232.227508452586

OX1 1,2-epoxybutane MN15 STRUC3

C	-1.7098811893	-0.3320146005	-0.5519296267
C	-1.0457205462	0.5181455995	0.5209582655
C	1.1416498821	-0.8596380100	0.1149588904
O	1.1775882383	0.3681943938	-0.5902463169
H	0.9233745990	0.8664223076	1.4327469696
H	-1.7184995854	-1.3865606400	-0.2768022937
H	-2.7424400943	-0.0233530898	-0.7052931295
H	-1.1811295375	-0.2294442553	-1.4995521091
C	0.4452385268	0.3249903053	0.6213460604
H	-1.2254376977	1.5769707740	0.3195112492
H	-1.4842133046	0.3132419990	1.5000950465
H	0.5753473353	-1.6480750977	-0.3667374434
H	2.0794877497	-1.1629855113	0.5653828740

Energy of Optimized Geometry: -232.227457530995

OX1 1,2-epoxybutane MN15 STRUC4

C	-2.1548693271	0.0349941355	0.0949508907
C	-0.8383426247	0.6467315794	-0.3670570913
C	1.0720945308	-1.0500522315	-0.0139969258
O	1.6055958828	0.2572557198	-0.1258339116
H	0.2743285991	0.3108310666	1.5055848193
H	-2.3382361823	0.2524115519	1.1481290642
H	-2.9960937485	0.4242816919	-0.4758979309
H	-2.1396615023	-1.0493641717	-0.0217029588
C	0.3152778605	0.1193641191	0.4359695555
H	-0.6496755399	0.4185129044	-1.4180423578
H	-0.8723618538	1.7342867092	-0.2772597566
H	0.7951213909	-1.5168250292	-0.9538446793
H	1.5650635672	-1.6926170659	0.7056753951

Energy of Optimized Geometry: -232.227168821675

OX1 1,2-epoxybutane MN15 STRUC5

C	-2.0981851454	-0.3765168469	0.3337510378
C	-0.9744747713	0.5329965832	-0.1468140761
C	1.2153040564	-0.8185189137	-0.3262809646
O	1.5095154198	0.5647313164	-0.2493505179
H	0.4279875991	0.1030283807	1.4982913951
H	-1.9253956933	-1.4057593986	0.0168504672
H	-2.1659146290	-0.3718928919	1.4225033473
H	-3.0621637696	-0.0593496260	-0.0602555414
C	0.3496796086	0.1012251519	0.4137336302
H	-0.9000908873	0.5188419022	-1.2360793523
H	-1.1699668472	1.5662110707	0.1468589083
H	0.8888645171	-1.1671268794	-1.3008820060
H	1.9012174099	-1.4691318383	0.2029761660

Energy of Optimized Geometry: -232.227168806871

OX1 1,2-epoxybutane MN15 STRUC6

C	-1.6994167326	-0.0982535081	-0.6636464458
C	-0.9493990029	0.7968409552	0.3116705322
C	0.9288845171	-1.0239849063	0.3795151328
O	1.2658174597	0.0189507055	-0.5173485949
H	0.9816832299	0.9289296133	1.3527205351
H	-1.0973139281	-0.2784360824	-1.5542648693
H	-1.9437318673	-1.0608834703	-0.2146496249
H	-2.6333586752	0.3665368165	-0.9747138899
C	0.4589188681	0.3454566812	0.6000928703
H	-0.8921666527	1.8130614502	-0.0859356885
H	-1.4871312670	0.8679250246	1.2597494657
H	0.2465791306	-1.7668812669	-0.0167261506
H	1.7506563873	-1.4098552533	0.9714100873

Energy of Optimized Geometry: -232.227457536719

OX1 1,2-epoxybutane MN15 STRUC7

C	-2.0002402815	0.5314347274	0.2258157129
C	-1.0018589671	-0.4945894063	-0.2961579606
C	1.5885451549	-0.5417210301	-0.3155744500
O	1.1087238200	0.7529196079	-0.0001725507
H	0.3168298690	-0.5280939514	1.4694381613
H	-2.1788508929	0.3914357413	1.2928788011
H	-1.6134842753	1.5396092662	0.0799063856
H	-2.9575893930	0.4547620278	-0.2872664538
C	0.3234345012	-0.3593641526	0.3952684826
H	-1.3808714972	-1.5086758152	-0.1507342817
H	-0.8401611312	-0.3569042277	-1.3673558343
H	1.5616974664	-0.7995689225	-1.3689537549
H	2.4760454830	-0.8515887038	0.2234753810

Energy of Optimized Geometry: -232.227508402452

2.5 ωB97X-D

OX1 1,2-epoxybutane ωB97X-D STRUC1

C	-2.1354426994	-0.3991334195	0.0395587488
C	-0.9562460687	0.5141183706	-0.2774391817
C	1.2758001417	-0.7981728156	-0.1600056518
O	1.5259049055	0.5933055516	-0.0280787834
H	0.2128646139	0.0786765732	1.5341804851
H	-3.0349668902	-0.0701280933	-0.4790668258
H	-1.9278866317	-1.4260071065	-0.2642283300
H	-2.3527164834	-0.4060940296	1.1088484122
C	0.2892284685	0.0893359678	0.4488935842
H	-0.7508092319	0.5176842350	-1.3493849597
H	-1.1936573558	1.5427595620	0.0027242632
H	1.1172947658	-1.1428690416	-1.1771672186
H	1.8903400004	-1.4393189558	0.4623872833

Energy of Optimized Geometry: -232.436857760107

OX1 1,2-epoxybutane ωB97X-D STRUC2

C	-2.0299327130	0.5446430653	0.1311139382
C	-0.9852996597	-0.4089742585	-0.4385112915
C	1.6008725074	-0.5587778987	-0.2448844116
O	1.1462634965	0.7158765475	0.1836584599
H	0.1771928432	-0.6957252761	1.4058351262
H	-1.6507669438	1.5663237283	0.1411938029
H	-2.9431265682	0.5266624020	-0.4628368846
H	-2.2915600906	0.2731440568	1.1551785857
C	0.2847122743	-0.4004652640	0.3641221384
H	-1.3764464628	-1.4294093569	-0.4557795891
H	-0.7500184798	-0.1368564178	-1.4691812309
H	1.6737692254	-0.6837243996	-1.3205720219
H	2.4194433051	-0.9757362799	0.3317536152

Energy of Optimized Geometry: -232.437047325923

OX1 1,2-epoxybutane ωB97X-D STRUC3

C	-1.7613601653	-0.3564670649	-0.4658195817
C	-1.0304734051	0.5044674077	0.5551920122
C	1.1775654831	-0.8384498899	0.0877485006
O	1.1612632960	0.3731760527	-0.6523311488
H	0.9620946631	0.9094153454	1.3671668764
H	-1.7124169754	-1.4143856149	-0.2066984399
H	-2.8133606493	-0.0784670697	-0.5140763400
H	-1.3323621793	-0.2277309945	-1.4593248239
C	0.4677037962	0.3367442299	0.5862394201
H	-1.2356663110	1.5601143923	0.3599698028
H	-1.4078193546	0.3014070286	1.5605052329
H	0.6235444631	-1.6559608720	-0.3596156166
H	2.1378397177	-1.1054583634	0.5158410992

Energy of Optimized Geometry: -232.436246283314

OX1 1,2-epoxybutane ωB97X-D STRUC4

C	-2.1601660646	0.2283494994	0.0665594525
C	-0.7771679031	0.7407615621	-0.3202260967
C	0.9946816316	-1.1373991145	-0.0915472861
O	1.6304697028	0.1315509914	-0.0728380286
H	0.2583448520	0.1346400267	1.5234145682
H	-2.3483540533	0.3666487613	1.1324547504
H	-2.9398277887	0.7584259691	-0.4789505092
H	-2.2589999302	-0.8355404784	-0.1531582508
C	0.3118145124	0.0381922555	0.4410219452
H	-0.6014526349	0.6018649135	-1.3884565107
H	-0.7071969184	1.8131275674	-0.1249263714
H	0.7236728173	-1.5021788812	-1.0775648921
H	1.4151828490	-1.8757692018	0.5824099682

Energy of Optimized Geometry: -232.436857650306

OX1 1,2-epoxybutane ωB97X-D STRUC5

C	-2.1423050818	-0.3627583659	0.0371472025
C	-0.9507807825	0.5610257662	-0.1914261648
C	1.2567045898	-0.7946312016	-0.2843272749
O	1.5365891320	0.5651770959	0.0113952458
H	0.2441802583	-0.1149433858	1.5271124365
H	-1.9624614219	-1.3477873961	-0.3956386769
H	-2.3381724671	-0.4979565553	1.1020222058
H	-3.0449920145	0.0443064057	-0.4166347418
C	0.2995012031	0.0278047128	0.4499215718
H	-0.7666219471	0.6925229650	-1.2591537603
H	-1.1612372730	1.5516059058	0.2177127892
H	1.0717119190	-1.0095658670	-1.3323222177
H	1.8701760641	-1.5182170584	0.2413213922

Energy of Optimized Geometry: -232.436857639898

OX1 1,2-epoxybutane ωB97X-D STRUC6

C	-1.7247898407	-0.0680964566	-0.6834801400
C	-0.9512090983	0.7907729155	0.3072499270
C	0.9385279834	-1.0311695652	0.3907318420
O	1.2875345304	0.0123513277	-0.5058885334
H	0.9643562569	0.9175298325	1.3601046759
H	-1.1583622591	-0.2026735474	-1.6047631291
H	-1.9472738016	-1.0530395058	-0.2721677151
H	-2.6730819556	0.4040760035	-0.9367008156
C	0.4547381314	0.3311907016	0.5993968566
H	-0.8874684658	1.8158634905	-0.0665060459
H	-1.4866663076	0.8459356760	1.2582761647
H	0.2747982934	-1.7845482153	-0.0185062162
H	1.7528708876	-1.4094246466	0.9994971237

Energy of Optimized Geometry: -232.436246185086

OX1 1,2-epoxybutane ωB97X-D STRUC7

C	-2.0566434654	0.4233696393	0.1629416033
C	-0.9691567459	-0.5004209220	-0.3749261249
C	1.6258582585	-0.4727777352	-0.2605179734
O	1.1023860063	0.7980214382	0.0939418526
H	0.2641044001	-0.5807753752	1.4437684222
H	-2.2697741832	0.2090274713	1.2115272515
H	-1.7449475040	1.4651315598	0.0899403729
H	-2.9838211061	0.3058712023	-0.3970235269
C	0.3210067486	-0.3546204719	0.3809940158
H	-1.2929621438	-1.5422048336	-0.3084435246
H	-0.7829965228	-0.2879965603	-1.4294110691
H	1.6746537986	-0.6697766718	-1.3267721256
H	2.4867739975	-0.7953309976	0.3150646113

Energy of Optimized Geometry: -232.437047304843

2.6 EOM-CCSD

OX1 1,2-epoxybutane EOM-CCSD STRUC1

C	-2.1398138827	-0.4053304178	0.0408751919
C	-0.9579746565	0.5136911807	-0.2797101783
C	1.2756048253	-0.8043096560	-0.1631024235
O	1.5335814737	0.6062458827	-0.0295294231
H	0.2143865605	0.0774314118	1.5414976178
H	-3.0415706667	-0.0800840520	-0.4808694748
H	-1.9266619618	-1.4343538584	-0.2593999941
H	-2.3562100614	-0.4070272764	1.1124736826
C	0.2853840600	0.0870430106	0.4553876526
H	-0.7432630093	0.5095071463	-1.3519710058
H	-1.1970512019	1.5452221606	-0.0036057133
H	1.1126731040	-1.1405598240	-1.1827083352
H	1.8970565594	-1.4415597793	0.4568137118

Energy of Optimized Geometry: -232.037430762393

OX1 1,2-epoxybutane EOM-CCSD STRUC2

C	-2.0265555140	0.5244014253	0.1605783361
C	-0.9869736048	-0.4291564126	-0.4333675736
C	1.6099824928	-0.5362053381	-0.2742883406
O	1.1285732567	0.7426073921	0.1802084061
H	0.2071810652	-0.7212248013	1.4019120874
H	-1.6393325199	1.5452314232	0.1779131923
H	-2.9482803557	0.5167658695	-0.4243371399
H	-2.2762300073	0.2391249263	1.1861232811
C	0.2921180502	-0.4151046025	0.3607479602
H	-1.3772016829	-1.4523506959	-0.4537455343
H	-0.7574363414	-0.1445757906	-1.4642121510
H	1.6652055227	-0.6425401544	-1.3532064708
H	2.4484073982	-0.9331305469	0.2881093961

Energy of Optimized Geometry: -232.037810400789

OX1 1,2-epoxybutane EOM-CCSD STRUC3

C	-1.7479613501	-0.3371323670	-0.4924114437
C	-1.0336263054	0.5190929231	0.5529303107
C	1.1610927002	-0.8613750130	0.1040431779
O	1.1677059611	0.3646910082	-0.6513817139
H	0.9689151332	0.9009824182	1.3822201348
H	-1.7100335199	-1.3978258771	-0.2342920040
H	-2.7989939108	-0.0516318690	-0.5628022556
H	-1.2917490938	-0.2046263482	-1.4753393560
C	0.4653090097	0.3333124182	0.6028138543
H	-1.2263520353	1.5789175782	0.3557700822
H	-1.4318659198	0.3116946846	1.5518719251
H	0.5928638987	-1.6667847015	-0.3475701183
H	2.1194756160	-1.1376591530	0.5310287190

Energy of Optimized Geometry: -232.036825181701

OX1 1,2-epoxybutane EOM-CCSD STRUC4

C	-2.1607086694	-0.0995402253	0.2570370835
C	-0.9122401358	0.5650280256	-0.3293075366
C	1.1540072627	-0.9827094662	-0.0559449865
O	1.5853847086	0.3768791062	-0.2546060633
H	0.3553762476	0.3745522244	1.4702492251
H	-2.2816863919	0.1554488810	1.3132117510
H	-3.0603947068	0.2240709548	-0.2692800752
H	-2.0959043282	-1.1878575984	0.1805351667
C	0.3321443714	0.1433007915	0.4068841704
H	-0.7936514126	0.3043615431	-1.3846980152
H	-1.0045080852	1.6540762967	-0.2754067161
H	0.8647977678	-1.5035673848	-0.9638405924
H	1.7484871732	-1.5502180329	0.6519514066

Energy of Optimized Geometry: -232.037430762096

OX1 1,2-epoxybutane EOM-CCSD STRUC5

C	-2.1548111560	-0.3103383845	0.0730108342
C	-0.9400824941	0.5928101063	-0.1569493439
C	1.2261455092	-0.8264319740	-0.3380833253
O	1.5604150870	0.5342007234	-0.0049749166
H	0.2741079597	-0.1683737348	1.5246858294
H	-2.0055047611	-1.2900766658	-0.3877575846
H	-2.3318616771	-0.4656635729	1.1405698087
H	-3.0571906077	0.1308134518	-0.3539143670
C	0.3058564951	0.0036239453	0.4503615525
H	-0.7651036466	0.7425884956	-1.2259720926
H	-1.1159801392	1.5798384405	0.2815498728
H	1.0092229664	-0.9955314740	-1.3885888671
H	1.8364731890	-1.5780562422	0.1509467751

Energy of Optimized Geometry: -232.037430762046

OX1 1,2-epoxybutane EOM-CCSD STRUC6

C	-1.7508921718	-0.2525731897	-0.5311173365
C	-0.9866247437	0.7454093340	0.3383998192
C	1.0395362554	-0.9327498495	0.3876497229
O	1.2333779404	0.0692978503	-0.6283459340
H	1.0061794693	1.0939107759	1.2050111060
H	-1.2357818384	-0.4088970574	-1.4808841876
H	-1.8564782580	-1.2186710035	-0.0324807973
H	-2.7541133027	0.1204837807	-0.7444448499
C	0.4738093278	0.4148824103	0.5425596201
H	-1.0373898846	1.7420429878	-0.1125851050
H	-1.4543450610	0.8270362479	1.3253496377
H	0.3982184173	-1.7559071186	0.0932860936
H	1.9351058369	-1.2017624438	0.9378652325

Energy of Optimized Geometry: -232.036825181704

OX1 1,2-epoxybutane EOM-CCSD STRUC7

C	-2.0200535347	0.5285681322	0.2182286557
C	-1.0061165682	-0.4965048887	-0.2955235850
C	1.5971250764	-0.5602401265	-0.3001661075
O	1.1251237259	0.7691174956	-0.0108567989
H	0.3123899599	-0.5000270945	1.4767883643
H	-2.1959651034	0.3996571321	1.2896295996
H	-1.6500960826	1.5429655618	0.0555679248
H	-2.9784067490	0.4263367351	-0.2944762961
C	0.3225653924	-0.3539043827	0.3981355678
H	-1.3792342226	-1.5137273258	-0.1353361902
H	-0.8506168296	-0.3691960482	-1.3706631898
H	1.5822413706	-0.8277577305	-1.3521318894
H	2.4778210899	-0.8619135041	0.2567588548

Energy of Optimized Geometry: -232.037810401283

3 *cis*-2,3-dimethyloxirane (OX2c)

3.1 BMK

OX2c *cis*-2,3-dimethyloxirane BMK STRUC1

H	-1.2238440950	0.8476653164	1.2338863843
C	0.7428972380	0.2792478267	0.4402024567
H	-1.9008783907	-1.5206151065	0.6135007804
H	-2.5042439102	-0.3743942055	-0.5905031629
H	-1.0731319990	-1.3459307038	-0.9473394075
O	-0.0000253998	1.0981406651	-0.4596816290
C	-0.7428933061	0.2792212584	0.4402304574
C	-1.6002700889	-0.8101320879	-0.1598248762
C	1.6002920708	-0.8100790005	-0.1598712561
H	2.5039957892	-0.3742494424	-0.5910240949
H	1.9013658745	-1.5202612286	0.6135482925
H	1.0729728947	-1.3462445306	-0.9470170904
H	1.2238583980	0.8477207856	1.2338325352

Energy of Optimized Geometry: -232.35610057256

OX2c *cis*-2,3-dimethyloxirane BMK STRUC2

H	-1.1728593690	0.8815960629	1.2596544647
C	0.7575434052	0.3498242304	0.3577324762
H	-1.7646050044	-1.5578379583	0.8751767830
H	-2.5159043223	-0.5426328254	-0.3626062535
H	-1.0643134128	-1.4694116895	-0.7536557335
O	-0.0953930893	1.0563200908	-0.5394655925
C	-0.7221313588	0.2763335682	0.4756056789
C	-1.5605077126	-0.8981536296	0.0288741386
C	1.6261284662	-0.7400456183	-0.2249466814
H	2.0229831262	-1.3715280030	0.5730946824
H	1.0715626864	-1.3616196554	-0.9257177983
H	2.4695244021	-0.2956348979	-0.7575043403
H	1.2645892052	1.0027067296	1.0654561574

Energy of Optimized Geometry: -232.356100665565

3.2 CAM-B3LYP

OX2c *cis*-2,3-dimethyloxirane CAM-B3LYP STRUC1

H	-1.2118178803	0.8361179413	1.2325314599
C	0.7320206350	0.2722675241	0.4366919062
H	-1.8875399825	-1.5152872957	0.6173867549
H	-2.4944687404	-0.3696263391	-0.5763509491
H	-1.0755705054	-1.3438354127	-0.9454150949
O	-0.0001112057	1.1002695737	-0.4651666720
C	-0.7319499308	0.2721470631	0.4368362370
C	-1.5887551148	-0.8046746483	-0.1540965834
C	1.5888161660	-0.8044743712	-0.1544251688
H	2.4947426126	-0.3694302474	-0.5761886736
H	1.8871887386	-1.5154485150	0.6168838098
H	1.0757388144	-1.3432183074	-0.9461019466
H	1.2119230691	0.8362620760	1.2323529838

Energy of Optimized Geometry: -232.397306743133

OX2c *cis*-2,3-dimethyloxirane CAM-B3LYP STRUC2

H	-1.1615167978	0.9392363169	1.2064495261
C	0.7476311443	0.3432364861	0.3520351048
H	-1.7893638636	-1.4853526345	0.9145523303
H	-2.5094857244	-0.5080376968	-0.3629720342
H	-1.0818610014	-1.4786783988	-0.7072294976
O	-0.0704579633	1.0372596798	-0.5881117569
C	-0.7123477766	0.3000529884	0.4506720487
C	-1.5643519571	-0.8636058584	0.0472113982
C	1.6045770553	-0.7698721241	-0.1669843315
H	1.9777874722	-1.3758205074	0.6594489823
H	1.0627318093	-1.4132476390	-0.8545701369
H	2.4650206129	-0.3610150201	-0.6965272487
H	1.2558401331	1.0108566697	1.0430276803

Energy of Optimized Geometry: -232.397306702128

3.3 M06-2X

OX2c *cis*-2,3-dimethyloxirane M06-2X STRUC1

H	-1.2142783338	0.8498556947	1.2273150894
C	0.7344553577	0.2798496978	0.4363742644
H	-1.8573411895	-1.5311658906	0.6167988326
H	-2.5001297477	-0.3844577468	-0.5604113030
H	-1.0658990382	-1.3277456375	-0.9550799960
O	-0.0000189585	1.0949703841	-0.4677090317
C	-0.7344552088	0.2798319632	0.4363920753
C	-1.5827490442	-0.8085945149	-0.1522600568
C	1.5827661651	-0.8085584352	-0.1522820889
H	2.4998442912	-0.3843080619	-0.5609969021
H	1.8579107284	-1.5307724533	0.6169144713
H	1.0657005041	-1.3281539185	-0.9546776185
H	1.2142880424	0.8499028951	1.2272710356

Energy of Optimized Geometry: -232.410968906207

OX2c *cis*-2,3-dimethyloxirane M06-2X STRUC2

H	-1.1641712282	0.9381278800	1.2116923141
C	0.7496496524	0.3502231040	0.3512823559
H	-1.7504164112	-1.5145340875	0.9060919111
H	-2.5133057433	-0.5262480369	-0.3409294291
H	-1.0712015619	-1.4600765450	-0.7292054354
O	-0.0756516690	1.0362728944	-0.5810910317
C	-0.7148562176	0.3013742876	0.4544723491
C	-1.5551575453	-0.8725384844	0.0465790880
C	1.6010725251	-0.7670371781	-0.1755284468
H	1.9616714175	-1.3866561918	0.6459415527
H	1.0525232566	-1.3931217755	-0.8742875004
H	2.4673503147	-0.3593117839	-0.6965323017
H	1.2572155760	1.0191057157	1.0409599409

Energy of Optimized Geometry: -232.410968693513

3.4 MN15

OX2c *cis*-2,3-dimethyloxirane MN15 STRUC1

H	-1.2149569537	0.8515388371	1.2273760859
C	0.7342703041	0.2825248576	0.4357663636
H	-1.8491911112	-1.5355498166	0.6161326663
H	-2.4981268756	-0.3918359000	-0.5595708032
H	-1.0568605335	-1.3241325609	-0.9544836612
O	-0.0000187098	1.0932391754	-0.4688776495
C	-0.7342706259	0.2825059922	0.4357865802
C	-1.5781220969	-0.8095711239	-0.1509463524
C	1.5781391337	-0.8095364506	-0.1509660210
H	2.4978349442	-0.3916844638	-0.5601666742
H	1.8497717296	-1.5351461149	0.6162617446
H	1.0566579641	-1.3245614129	-0.9540651392
H	1.2149687516	0.8515833554	1.2273316525

Energy of Optimized Geometry: -232.233214906609

OX2c *cis*-2,3-dimethyloxirane MN15 STRUC2

H	-1.1857476624	0.9353800985	1.1946771908
C	0.7433752162	0.3517851322	0.3643228391
H	-1.7560452408	-1.5220066019	0.8708627649
H	-2.5050631666	-0.5331043781	-0.3828705278
H	-1.0500357493	-1.4538732472	-0.7525628348
O	-0.0657301394	1.0366437875	-0.5794399046
C	-0.7222504505	0.3024410162	0.4423441783
C	-1.5507321108	-0.8735624148	0.0185802085
C	1.5988234692	-0.7674990791	-0.1498018347
H	1.9367877256	-1.3981297906	0.6730568500
H	1.0585802276	-1.3823026418	-0.8655301813
H	2.4799803438	-0.3652263884	-0.6493775936
H	1.2405840010	1.0170531649	1.0654635491

Energy of Optimized Geometry: -232.233215138855

3.5 ωB97X-D

OX2c *cis*-2,3-dimethyloxirane ωB97X-D STRUC1

H	-1.2130008113	0.8393380012	1.2311330265
C	0.7323161822	0.2758748145	0.4344347329
H	-1.8687052273	-1.5242952755	0.6204088431
H	-2.5036676818	-0.3780392453	-0.5611177541
H	-1.0796991593	-1.3384398541	-0.9538693825
O	-0.0000710785	1.1001380757	-0.4650029855
C	-0.7322624444	0.2757952893	0.4345499615
C	-1.5884464832	-0.8074290421	-0.1527225199
C	1.5884712986	-0.8072952181	-0.1529231777
H	2.5036936794	-0.3778920026	-0.5612641374
H	1.8687675344	-1.5242167054	0.6201544811
H	1.0797564150	-1.3382418756	-0.9541377263
H	1.2130479991	0.8394010697	1.2310465481

Energy of Optimized Geometry: -232.442572592147

OX2c *cis*-2,3-dimethyloxirane ωB97X-D STRUC2

H	-1.1633457292	0.9313398793	1.2130437493
C	0.7474612703	0.3458766279	0.3507053799
H	-1.7660579157	-1.5034851652	0.9098273915
H	-2.5165006587	-0.5179401693	-0.3465120181
H	-1.0843611647	-1.4721920783	-0.7240702656
O	-0.0735551777	1.0409738999	-0.5805004319
C	-0.7129387176	0.2986402972	0.4514293174
C	-1.5618337432	-0.8699884833	0.0453615438
C	1.6058204157	-0.7677118090	-0.1733858601
H	1.9652687947	-1.3846292303	0.6514727733
H	1.0671816572	-1.4009978202	-0.8744019799
H	2.4740354233	-0.3568170021	-0.6893192167
H	1.2563577461	1.0093745828	1.0464058591

Energy of Optimized Geometry: -232.442572447258

3.6 EOM-CCSD

OX2c *cis*-2,3-dimethyloxirane EOM-CCSD STRUC1

H	-1.2182821149	0.8460131358	1.2346717030
C	0.7363830129	0.2756138970	0.4430393566
H	-1.8763311078	-1.5307307699	0.6178133605
H	-2.4986412191	-0.3817313029	-0.5748493587
H	-1.0631329390	-1.3404204374	-0.9494379692
O	-0.0000080223	1.1086497064	-0.4749557720
C	-0.7363788040	0.2756093166	0.4430524975
C	-1.5872243741	-0.8123414550	-0.1540603807
C	1.5872276131	-0.8123326857	-0.1540785477
H	2.4984082097	-0.3816519043	-0.5753077686
H	1.8767560485	-1.5304427524	0.6178964947
H	1.0629639523	-1.3407530912	-0.9491177410
H	1.2182978090	0.8460380434	1.2346375754

Energy of Optimized Geometry: -232.043000296984

OX2c *cis*-2,3-dimethyloxirane EOM-CCSD STRUC2

H	-1.1677115698	0.9355121582	1.2188602631
C	0.7521288159	0.3473105039	0.3580423980
H	-1.7672396154	-1.5153032937	0.9091924523
H	-2.5133224079	-0.5263774941	-0.3538579748
H	-1.0676216181	-1.4719742856	-0.7225080727
O	-0.0768748296	1.0489353279	-0.5903333111
C	-0.7161655153	0.2978591966	0.4613907560
C	-1.5592870465	-0.8771949316	0.0460400521
C	1.6055644668	-0.7706020985	-0.1767822106
H	1.9746921290	-1.3892025998	0.6457348557
H	1.0520335099	-1.4006627280	-0.8716166425
H	2.4684799112	-0.3585673668	-0.7047764977
H	1.2615228180	1.0173226100	1.0478639581

Energy of Optimized Geometry: -232.043000297684

4 *trans*-2,3-dimethyloxirane (OX2t)

4.1 BMK

OX2t *trans*-2,3-dimethyloxirane BMK STRUC1

C	-1.9842878645	0.4278480493	0.1119740788
C	-0.6043073508	0.1379673777	-0.4280495831
H	2.2417552423	1.4778353987	0.0421993836
H	2.7290543792	-0.1858264416	0.3990575515
C	0.6043128853	0.1379551431	0.4280419989
H	-2.7290519290	-0.1857699749	-0.3989900824
H	-2.0269175386	0.2053760616	1.1788391055
H	-2.2417162610	1.4779203421	-0.0423452967
H	2.0269505720	0.2051446523	-1.1788289612
H	-0.4547898913	0.3431724639	-1.4862707762
O	-0.0000255678	-1.0808487766	0.0000222297
C	1.9843095963	0.4277464956	-0.1119887513
H	0.4547965499	0.3432220816	1.4862512805

Energy of Optimized Geometry: -232.357816033772

OX2t *trans*-2,3-dimethyloxirane BMK STRUC2

C	-1.9791293805	0.3835394154	0.2637849252
C	-0.6191295172	0.2165239488	-0.3705960765
H	1.9838612415	0.4501516364	-1.1846079841
H	2.2425202007	1.4544968692	0.2580688916
C	0.6192720001	0.0540296594	0.4254930472
H	-1.9834451230	-0.0439169968	1.2671874902
H	-2.2433096944	1.4413470189	0.3272989328
H	-2.7407391850	-0.1243047639	-0.3315742362
H	2.7412877903	-0.2437757495	0.2578621450
H	-0.5072031753	0.6288228631	-1.3714734847
O	-0.0002954847	-1.0591702247	-0.2146203331
C	1.9793113882	0.4547256229	-0.0939880707
H	0.5078538469	0.0444655444	1.5080277947

Energy of Optimized Geometry: -232.357815930241

4.2 CAM-B3LYP

OX2t *trans*-2,3-dimethyloxirane CAM-B3LYP STRUC1

C	-1.9658004576	0.4318793917	0.1047114253
C	-0.5956879958	0.1373617781	-0.4214632690
H	2.2189755186	1.4808315371	0.0520715015
H	2.7114140249	-0.1754162113	0.4080443529
C	0.5957800819	0.1373522598	0.4215713236
H	-2.7111423537	-0.1756486518	-0.4084150612
H	-2.0226171871	0.2141396711	1.1697535846
H	-2.2191492023	1.4807971127	-0.0527067544
H	2.0222749761	0.2138430870	-1.1699889437
H	-0.4464652425	0.3427025322	-1.4782542889
O	-0.0000384764	-1.0887464263	0.0001924081
C	1.9657608688	0.4318347490	-0.1049786810
H	0.4466950381	0.3428775004	1.4783375337

Energy of Optimized Geometry: -232.399322531089

OX2t *trans*-2,3-dimethyloxirane CAM-B3LYP STRUC2

C	-1.9398013192	0.4970314964	0.2281340606
C	-0.5954438327	0.2352934265	-0.3759717179
H	2.0100407635	0.3193067934	-1.1677389589
H	2.2905860610	1.3461265690	0.2478085971
C	0.6092019167	0.0348485379	0.4232725324
H	-1.9888798268	0.1022044154	1.2413936073
H	-2.1440755018	1.5677871177	0.2590455730
H	-2.7232472214	0.0215912950	-0.3619556937
H	2.7061033850	-0.3673734227	0.3020413820
H	-0.4549614605	0.6134636371	-1.3852012176
O	-0.0540811276	-1.0711070334	-0.1868348966
C	1.9825990762	0.3527226836	-0.0800318914
H	0.4893398306	0.0617279632	1.5031822508

Energy of Optimized Geometry: -232.399322559256

4.3 M06-2X

OX2t *trans*-2,3-dimethyloxirane M06-2X STRUC1

C	-1.9672779329	0.4274758192	0.1084535846
C	-0.5963760967	0.1379408661	-0.4253786148
H	2.2223461808	1.4771256699	0.0386920095
H	2.7129192536	-0.1799527236	0.4044653911
C	0.5963712543	0.1380143932	0.4253540863
H	-2.7129102565	-0.1801068110	-0.4043703475
H	-2.0113473475	0.1994834705	1.1724884684
H	-2.2223943206	1.4770719132	-0.0390008032
H	2.0113760820	0.1992412306	-1.1724962318
H	-0.4417888358	0.3456776341	-1.4809228132
O	0.0000089891	-1.0806093732	0.0000809139
C	1.9672731267	0.4274826653	-0.1085132964
H	0.4417714650	0.3459183434	1.4808631991

Energy of Optimized Geometry: -232.412684072195

OX2t *trans*-2,3-dimethyloxirane M06-2X STRUC2

C	-1.9520537539	0.4400756424	0.2474123041
C	-0.6013832438	0.2372267400	-0.3709919152
H	1.9909856595	0.3936711429	-1.1591005545
H	2.2639324815	1.3849511557	0.2855002828
C	0.6090752746	0.0328085875	0.4285399261
H	-1.9794050786	-0.0010017490	1.2428297313
H	-2.1825112079	1.5027853363	0.3254010507
H	-2.7244606718	-0.0326803680	-0.3592358617
H	2.7145192944	-0.3230351465	0.2850485209
H	-0.4638422792	0.6570313793	-1.3640405387
O	-0.0300895326	-1.0564407327	-0.2243549032
C	1.9758457716	0.3955470580	-0.0701169175
H	0.4834493491	0.0198785980	1.5080323918

Energy of Optimized Geometry: -232.412684012781

4.4 MN15

OX2t *trans*-2,3-dimethyloxirane MN15 STRUC1

C	-1.9628454175	0.4272882896	0.1103966274
C	-0.5943970470	0.1368866337	-0.4274267851
H	2.2182918999	1.4781305558	0.0274847850
H	2.7136592776	-0.1751984937	0.4005160062
C	0.5943932759	0.1369475287	0.4273999652
H	-2.7136545155	-0.1753111908	-0.4004122010
H	-1.9995558467	0.1926415060	1.1736985540
H	-2.2183178319	1.4781061659	-0.0277613438
H	1.9995981974	0.1923946641	-1.1736956367
H	-0.4368926551	0.3493610679	-1.4821034718
O	-0.0000002438	-1.0790508672	0.0000695049
C	1.9628495392	0.4272735589	-0.1104430589
H	0.4368711684	0.3495780451	1.4820424073

Energy of Optimized Geometry: -232.234745533999

OX2t *trans*-2,3-dimethyloxirane MN15 STRUC2

C	-1.9240653217	0.5434931413	0.2237234228
C	-0.5922656928	0.2306214511	-0.3882846131
H	1.9820917234	0.2030794735	-1.2015058654
H	2.3186197213	1.3069561159	0.1445603849
C	0.6119841214	0.0401976522	0.4225408803
H	-1.9542931591	0.1786053011	1.2498457127
H	-2.1061904810	1.6183232661	0.2263208230
H	-2.7273067942	0.0637472139	-0.3351589721
H	2.7021507845	-0.4104101189	0.2912378567
H	-0.4430381559	0.5642672257	-1.4123475785
O	-0.0776825679	-1.0677987018	-0.1352744214
C	1.9856018590	0.3022647459	-0.1165420163
H	0.4933561281	0.1272808639	1.4998767216

Energy of Optimized Geometry: -232.234745529586

4.5 ωB97X-D

OX2t *trans*-2,3-dimethyloxirane ωB97X-D STRUC1

C	-1.9758987667	0.3851358721	0.1319905569
C	-0.6051766555	0.1168670169	-0.4140863720
H	2.1758415095	1.5400352391	0.0119995264
H	2.7244120549	-0.0995127396	0.3714104843
C	0.5984448621	0.1507776284	0.4127732570
H	-2.7159759621	-0.2391821606	-0.3694094466
H	-2.0127967046	0.1704889866	1.1993708258
H	-2.2501072025	1.4291084276	-0.0257981639
H	2.0024513989	0.2596386994	-1.2012260736
H	-0.4764661981	0.3225258628	-1.4744710729
O	0.0271416413	-1.0873177407	0.0049886490
C	1.9542844776	0.4822009680	-0.1359112359
H	0.4593955086	0.3593891707	1.4712683285

Energy of Optimized Geometry: -232.444122188286

OX2t *trans*-2,3-dimethyloxirane ωB97X-D STRUC2

C	-1.9540466470	0.4405881213	0.2399906726
C	-0.6012301067	0.2280101036	-0.3712680537
H	2.0056689658	0.4108088193	-1.1531024112
H	2.2487873682	1.4010325762	0.2964127774
C	0.6075016051	0.0339312381	0.4254255310
H	-1.9946022845	0.0141706182	1.2416684147
H	-2.1796785224	1.5060594416	0.3027954770
H	-2.7261733840	-0.0339102687	-0.3663857547
H	2.7183144146	-0.3011257561	0.2988889283
H	-0.4692353137	0.6430022130	-1.3681075271
O	-0.0256672560	-1.0646638115	-0.2203265769
C	1.9745751467	0.4087264081	-0.0641725893
H	0.4851737484	0.0254163714	1.5062931578

Energy of Optimized Geometry: -232.444122102408

4.6 EOM-CCSD

OX2t *trans*-2,3-dimethyloxirane EOM-CCSD STRUC1

C	-1.9728497403	0.4321239117	0.1045848179
C	-0.5965176437	0.1432893089	-0.4282343666
H	2.2296731325	1.4834965458	0.0491576795
H	2.7173364743	-0.1805378504	0.4086570761
C	0.5965143698	0.1433895451	0.4282013711
H	-2.7173258510	-0.1807105673	-0.4085737088
H	-2.0185041642	0.2091797769	1.1719046631
H	-2.2297195942	1.4834328904	-0.0495345126
H	2.0185202217	0.2089033628	-1.1719354786
H	-0.4405291295	0.3473856641	-1.4857733101
O	0.0000088523	-1.0977992310	0.0001164590
C	1.9728441754	0.4321337666	-0.1046731479
H	0.4405136582	0.3477165098	1.4856939041

Energy of Optimized Geometry: -232.044810362931

OX2t *trans*-2,3-dimethyloxirane EOM-CCSD STRUC2

C	-1.9610783392	0.4358681779	0.2324416304
C	-0.6015048587	0.2345938376	-0.3780119660
H	1.9996104439	0.4015290092	-1.1540316158
H	2.2605229870	1.4076414151	0.2881617661
C	0.6074710590	0.0464540312	0.4342593164
H	-1.9909062101	0.0083056695	1.2360637793
H	-2.1989731000	1.5010784507	0.2934217903
H	-2.7278037348	-0.0517242372	-0.3737996206
H	2.7203467397	-0.3024501523	0.3031013868
H	-0.4615956947	0.6400313878	-1.3782233458
O	-0.0228162741	-1.0762378512	-0.2153038902
C	1.9790349916	0.4114155817	-0.0629563810
H	0.4760640880	0.0414127651	1.5145754451

Energy of Optimized Geometry: -232.044810362869

5 1-(oxiran-2-yl)ethan-1-one (OX3)

5.1 BMK

(OX3) 1-(oxiran-2-yl)ethan-1-one BMK STRUC1

C	-1.0288865187	-1.4217487081	-0.0967875300
C	-0.8729034322	0.0793119741	0.0423824033
O	1.5563344080	-0.2491229227	0.5832289203
H	1.3940681500	-0.5529068803	-1.5054318897
C	0.5250541342	0.6362514579	0.1759096089
H	-2.0722216522	-1.6849870244	0.0602862153
H	-0.7374013936	-1.7243082859	-1.1072191522
H	-0.3776182942	-1.9458746440	0.6041662951
H	2.4034824063	0.8999850847	-0.9833184811
O	-1.8038279663	0.8415758985	0.0368137138
H	0.5670959137	1.6382325203	0.5900469404
C	1.6076683572	0.1991833491	-0.7524585881

Energy of Optimized Geometry: -306.366788785920

(OX3) 1-(oxiran-2-yl)ethan-1-one BMK STRUC2

C	-1.666412974385	-1.305733205983	0.586423262054
C	-0.792285186869	-0.234532222420	-0.037678202045
O	1.559878977887	0.290114611735	-0.656120149154
H	0.880204990914	1.828785969895	0.613068030309
C	0.692990257131	-0.352245982856	0.249042982600
H	-1.396392884112	-2.283875388326	0.179609269382
H	-1.495729244153	-1.344675572293	1.665807235623
H	-2.713146218847	-1.093772051524	0.380837656265
H	2.306185130053	0.822642062168	1.250191263974
O	-1.226589512015	0.669464382764	-0.694815327655
H	1.045887153039	-1.303259063396	0.637631902295
C	1.436774422647	0.896859146248	0.605875472848

Energy of Optimized Geometry: -306.364202406619

(OX3) 1-(oxiran-2-yl)ethan-1-one BMK STRUC3

C	-1.475472757751	-1.353813837636	0.582690408981
C	-0.831756320546	-0.078616994945	0.076619910397
O	1.579084441742	-0.382276529386	-0.273086530200
H	1.102711107995	1.522479743967	-1.086292282606
C	0.583530014207	0.178507656941	0.582405498286
H	-2.439785091349	-1.501475496590	0.101789967225
H	-0.812513821266	-2.198240414287	0.375664274666
H	-1.603692799229	-1.297278961896	1.667339380288
H	2.322557019041	1.521414797459	0.304672347749
O	-1.362984843026	0.666175063046	-0.699200269631
H	0.759693014593	0.015033580291	1.642764392498
C	1.492014628694	1.038281457252	-0.198200425426

Energy of Optimized Geometry: -306.361444584029

(OX3) 1-(oxiran-2-yl)ethan-1-one BMK STRUC4

C	-1.642216165380	-1.337964458311	0.577894493132
C	-0.800569497783	-0.203133025001	0.024394519189
O	1.481095769976	0.365081172622	-0.789445689610
H	1.034648098949	1.663120344849	0.808800956374
C	0.700513327432	-0.409797425430	0.088455419791
H	-1.241697997229	-1.684596515507	1.533478361798
H	-2.672386631760	-1.008993354818	0.693158851281
H	-1.603664010501	-2.182043595307	-0.117270520431
H	2.476622813721	0.522208126289	1.071170206575
O	-1.269674933170	0.806702600630	-0.420357419496
H	1.051594503682	-1.424391459141	0.254339876554
C	1.540663750867	0.734587632095	0.565406633677

Energy of Optimized Geometry: -306.364151992837

5.2 CAM-B3LYP

(OX3) 1-(oxiran-2-yl)ethan-1-one CAM-B3LYP STRUC1

C	-1.046877693560	-1.404551141437	-0.033672166534
C	-0.858810321752	0.078929020430	0.084526177918
O	1.579799476687	-0.329710232518	0.455358008988
H	1.284779211723	-0.375619016615	-1.642635313520
C	0.540656877414	0.603703908018	0.197654452006
H	-2.086217391697	-1.618815970911	-0.261817937908
H	-0.393626158356	-1.832734147466	-0.792556706542
H	-0.770001950167	-1.874360927864	0.910756532971
H	2.332576791754	0.995913450092	-1.018289427594
O	-1.776944764332	0.860472232404	0.104748114104
H	0.615153678702	1.551870317407	0.716676964095
C	1.545278511454	0.279329178845	-0.819728972590

Energy of Optimized Geometry: -306.415007417181

(OX3) 1-(oxiran-2-yl)ethan-1-one CAM-B3LYP STRUC2

C	-1.657713908698	-1.302511770905	0.564557151221
C	-0.788789129461	-0.225494367378	-0.019473371434
O	1.561649141286	0.247068132293	-0.665974778744
H	0.890167839374	1.837243127194	0.543802019223
C	0.682923253059	-0.331951350568	0.278756424583
H	-1.427161277216	-2.256172336872	0.086119785282
H	-1.455416793352	-1.423573671053	1.629737170936
H	-2.703989455571	-1.060309603223	0.408371487720
H	2.291013198138	0.844453183715	1.230309028613
O	-1.223083351973	0.684501433850	-0.675320194188
H	1.026002769114	-1.267673124991	0.706907833056
C	1.428150330800	0.897597744143	0.577211048807

Energy of Optimized Geometry: -306.411977022370

(OX3) 1-(oxiran-2-yl)ethan-1-one CAM-B3LYP STRUC3

C	-1.4214109451	-1.3614454791	0.4962897849
C	-0.8339767683	-0.0482579783	0.0719945446
O	1.5799520266	-0.4518340502	-0.1827481119
H	1.1609489182	1.3671214874	-1.2045851673
C	0.5705953125	0.2223905422	0.5629373294
H	-2.4134172488	-1.4845132954	0.0740019014
H	-0.7694989263	-2.1715598400	0.1684355727
H	-1.4718823627	-1.4145610741	1.5848324729
H	2.3253929874	1.4962077297	0.2157636147
O	-1.4146512372	0.7395955665	-0.6287594785
H	0.7072328858	0.1861608391	1.6400128120
C	1.5039997052	0.9732723824	-0.2572207381

Energy of Optimized Geometry: -306.409798435692

(OX3) 1-(oxiran-2-yl)ethan-1-one CAM-B3LYP STRUC4

C	-1.633147274639	-1.336899700560	0.550761669261
C	-0.796261264462	-0.194620720015	0.049884305314
O	1.468223434672	0.331242179727	-0.824763651911
H	1.059851950456	1.665800164617	0.754504295000
C	0.693984479842	-0.399378314999	0.105801874452
H	-1.312411619764	-1.634550757682	1.550581758792
H	-2.680584822123	-1.052387892459	0.565211991295
H	-1.496198239200	-2.203528262103	-0.098385485420
H	2.481069637119	0.515073186798	1.026170135052
O	-1.265721781499	0.834709165549	-0.358415273115
H	1.037464085792	-1.411466255728	0.290266590513
C	1.542002347987	0.722899987590	0.527260912249

Energy of Optimized Geometry: -306.411981016424

5.3 M06-2X

(OX3) 1-(oxiran-2-yl)ethan-1-one M06-2X STRUC1

C	-1.0083715458	-1.4101107499	-0.1334043084
C	-0.8636413167	0.0772854574	0.0417539749
O	1.5482198453	-0.2260197926	0.5978901184
H	1.3783664753	-0.6058166996	-1.4719729810
C	0.5218463903	0.6423026193	0.1492192536
H	-2.0456679043	-1.6859571150	0.0285357811
H	-0.7241083561	-1.6862211588	-1.1505693058
H	-0.3481171947	-1.9458902972	0.5484835636
H	2.3856553647	0.8626847792	-1.0092187037
O	-1.8045839041	0.8290543460	0.0819464403
H	0.5618657448	1.6551955390	0.5264476977
C	1.5903400788	0.1750110822	-0.7520807179

Energy of Optimized Geometry: -306.428379170955

(OX3) 1-(oxiran-2-yl)ethan-1-one M06-2X STRUC2

C	-1.662104212454	-1.298286550517	0.564751405575
C	-0.787395944997	-0.225449132811	-0.024371731571
O	1.560560311880	0.244727509242	-0.657214789707
H	0.865196151531	1.829974759213	0.537363956355
C	0.684297800438	-0.342240089841	0.277396984299
H	-1.434764880100	-2.250835898821	0.081968873826
H	-1.447369293978	-1.418396779969	1.627834261913
H	-2.708383552169	-1.050479570384	0.416117428016
H	2.279172751948	0.855261069114	1.235337532786
O	-1.211876985027	0.688540029673	-0.680294528775
H	1.026670206844	-1.276063867864	0.709856313459
C	1.419654526976	0.900051670752	0.577957299520

Energy of Optimized Geometry: -306.425499995583

(OX3) 1-(oxiran-2-yl)ethan-1-one M06-2X STRUC3

C	-1.392946167878	-1.372759180823	0.446881283168
C	-0.837632920827	-0.026998325931	0.074503055215
O	1.562412602095	-0.481184981396	-0.143464376265
H	1.174280164344	1.282401693556	-1.261325200327
C	0.568867600269	0.243010373902	0.565857390395
H	-2.384332842312	-1.503224714729	0.024042207236
H	-0.715855508956	-2.146664933974	0.081953911992
H	-1.431939692735	-1.466378522186	1.533575970810
H	2.349664272836	1.466131351688	0.148538633313
O	-1.435478558293	0.780563394327	-0.586920037499
H	0.702237881107	0.258200946896	1.644042416452
C	1.518214869098	0.934872899344	-0.296023262148

Energy of Optimized Geometry: -306.422570036831

(OX3) 1-(oxiran-2-yl)ethan-1-one M06-2X STRUC4

C	-1.665889822190	-1.269473469856	0.618295603460
C	-0.769724051455	-0.261103424550	-0.061170922097
O	1.560377709974	0.356104972190	-0.628559206545
H	0.811162728940	1.789806317146	0.718044327306
C	0.703226058814	-0.354324689614	0.230134902586
H	-1.111735867774	-2.018721951962	1.178711337102
H	-2.337482009832	-0.738294431487	1.292087415228
H	-2.278985413488	-1.756210881582	-0.138782936904
H	2.250465869721	0.792433585687	1.321035965794
O	-1.205262423964	0.604417615852	-0.773027997386
H	1.077826364195	-1.311141912463	0.575524700367
C	1.392482952771	0.876903438588	0.665485364601

Energy of Optimized Geometry: -306.423846507879

5.4 MN15

(OX3) 1-(oxiran-2-yl)ethan-1-one MN15 STRUC1

C	-1.001103493551	-1.407515477671	-0.136527404986
C	-0.861870362643	0.079322996199	0.040761309565
O	1.544279774459	-0.227991756569	0.599377871422
H	1.369214336289	-0.610915263523	-1.470416867341
C	0.523802594666	0.642970812858	0.149591929177
H	-2.037020827456	-1.689023209571	0.024768431154
H	-0.716577129891	-1.680565695887	-1.154703643813
H	-0.337410590176	-1.941337903258	0.540968005087
H	2.384984881705	0.852284178505	-1.013588892480
O	-1.808691749963	0.831116666030	0.082800909194
H	0.562014976509	1.659348929836	0.523125240282
C	1.588709099187	0.167718545878	-0.748958415713

Energy of Optimized Geometry: -306.205050700534

(OX3) 1-(oxiran-2-yl)ethan-1-one MN15 STRUC2

C	-1.6610743999	-1.3390148298	0.4575187868
C	-0.7961862198	-0.1829119249	0.0499654041
O	1.5230164491	0.1088388493	-0.7098984601
H	0.9561772229	1.8538404234	0.3260582215
C	0.6793939715	-0.3194063019	0.3386188003
H	-1.3425858657	-2.2259157867	-0.0925309110
H	-1.5280550956	-1.5591159065	1.5173605292
H	-2.7015515343	-1.1254613212	0.2451057417
H	2.3781430411	0.9056502149	1.0470399240
O	-1.2190281060	0.8024887075	-0.5039543156
H	1.0034481224	-1.2141453086	0.8583599039
C	1.4783544000	0.9106443039	0.4434798066

Energy of Optimized Geometry: -306.201655780030

(OX3) 1-(oxiran-2-yl)ethan-1-one MN15 STRUC3

C	-1.3995648494	-1.3688319545	0.4583720847
C	-0.8335777819	-0.0310805271	0.0763969229
O	1.5591527412	-0.4736242253	-0.1523879725
H	1.1541584359	1.3055358439	-1.2425212288
C	0.5704961437	0.2366406255	0.5738410836
H	-2.3899562998	-1.4966370951	0.0309627081
H	-0.7273505685	-2.1524131867	0.1034987077
H	-1.4450112555	-1.4553416130	1.5454497780
H	2.3454363544	1.4704064650	0.1545763852
O	-1.4217762777	0.7720383686	-0.6072906812
H	0.7055701319	0.2415308295	1.6525704038
C	1.5106302815	0.9414515501	-0.2868021231

Energy of Optimized Geometry: -306.199043144529

(OX3) 1-(oxiran-2-yl)ethan-1-one MN15 STRUC4

C	-1.671313558216	-1.283362495301	0.593371422817
C	-0.771430075875	-0.251017252713	-0.038684230582
O	1.553798854049	0.318801401015	-0.651885789695
H	0.822425491499	1.790274341937	0.660311166792
C	0.703420515462	-0.361536293291	0.237183952267
H	-1.121964266122	-2.033846622304	1.157563648107
H	-2.377468197310	-0.779468886627	1.251690981853
H	-2.248430535079	-1.770572706051	-0.191311048527
H	2.268125950867	0.810682866141	1.276979105841
O	-1.202979364229	0.653550965920	-0.711552681371
H	1.071762990197	-1.313664664680	0.603398480544
C	1.404875430965	0.876724253780	0.625813284475

Energy of Optimized Geometry: -306.200403424573

5.5 ωB97X-D

(OX3) 1-(oxiran-2-yl)ethan-1-one ωB97X-D STRUC1

C	-1.049373564058	-1.407324519765	-0.030931739409
C	-0.860781922221	0.079773076686	0.086717472873
O	1.583181427992	-0.329810956242	0.449343049244
H	1.277005669132	-0.371736694378	-1.646554059847
C	0.543491013655	0.601048297279	0.203163554719
H	-2.087147191539	-1.622685753657	-0.267037399755
H	-0.389298664592	-1.835607754868	-0.785099337742
H	-0.778660879937	-1.874124230414	0.917500658867
H	2.327454273083	1.001065796732	-1.022620970603
O	-1.777104555498	0.863377889992	0.103611103007
H	0.621540051597	1.549795633270	0.722361495222
C	1.543605584161	0.280137318595	-0.821175740650

Energy of Optimized Geometry: -306.451362367824

(OX3) 1-(oxiran-2-yl)ethan-1-one ωB97X-D STRUC2

C	-1.655309625932	-1.302717254673	0.568375342155
C	-0.793133464446	-0.220660253841	-0.024884828118
O	1.566864102002	0.244778492851	-0.658130121174
H	0.899475464243	1.839643427737	0.547060707045
C	0.681187923537	-0.330535501787	0.277573629566
H	-1.421855037984	-2.254184699380	0.085481402916
H	-1.437143276812	-1.421877469292	1.631432025113
H	-2.705955532093	-1.068549677585	0.425787389686
H	2.289663022202	0.836287276627	1.242001039438
O	-1.229814242961	0.684321220671	-0.686055485278
H	1.017096924834	-1.268133848914	0.709979389214
C	1.432110727946	0.895750934034	0.580775045919

Energy of Optimized Geometry: -306.448074787246

(OX3) 1-(oxiran-2-yl)ethan-1-one ωB97X-D STRUC3

C	-1.399017458490	-1.374626160454	0.448169827097
C	-0.840074436997	-0.030789031911	0.072050451780
O	1.576950084511	-0.479087808345	-0.144657339074
H	1.189662060539	1.286571108830	-1.266129943525
C	0.571268167061	0.240274864081	0.554748378541
H	-2.393451729973	-1.502441499941	0.030480817195
H	-0.730859060895	-2.156543505860	0.083710911551
H	-1.436929037616	-1.467495955269	1.535697420867
H	2.343522253333	1.478738607587	0.157639533380
O	-1.445963665285	0.781841655609	-0.577307621200
H	0.697877467443	0.261152697688	1.635023191457
C	1.520960820860	0.937967771787	-0.295442374484

Energy of Optimized Geometry: -306.445610211073

(OX3) 1-(oxiran-2-yl)ethan-1-one ωB97X-D STRUC4

C	-1.661402349540	-1.269741617419	0.623352166763
C	-0.774236235644	-0.259265060981	-0.065635965746
O	1.570108492901	0.358808096529	-0.622379166647
H	0.831238415060	1.800004077650	0.726264932042
C	0.700548992945	-0.342520633259	0.232556060271
H	-1.101944038995	-2.029353409042	1.165451716307
H	-2.313114680132	-0.742293083673	1.320114023041
H	-2.297370131494	-1.745604678205	-0.121761102970
H	2.250392048491	0.783598167659	1.336467069850
O	-1.218643109168	0.591485860471	-0.790816216686
H	1.069339677331	-1.302256879846	0.580533545829
C	1.397757789732	0.876640325573	0.672868474337

Energy of Optimized Geometry: -306.446698290847

6 3,4-epoxybutanol (OX4)

6.1 BMK

OX4 3,4-epoxybutanol BMK STRUC1

C	-2.0568221883	-0.0406696209	0.2946686473
C	-0.8668884894	0.4785607149	-0.4137501255
H	0.2366588280	0.9041511647	1.3818376475
H	-2.0960624176	0.0312256641	1.3769099075
H	-3.0124938781	-0.0867274158	-0.2161911858
O	1.8148140127	-0.8431066888	0.2069495304
H	1.6893927026	0.5790073522	-1.2930107861
H	2.4814198532	1.0531334248	0.2135820656
C	0.3168688751	1.0866046291	0.3072642281
O	-1.0663130762	-0.9275086862	-0.2136485177
H	-0.9918773204	0.7769847742	-1.4521452725
C	1.6420541473	0.4906614893	-0.1983559260
H	0.3117900999	2.1676511909	0.1417085743
H	0.9894804248	-1.3186069501	0.0747571029

Energy of Optimized Geometry: -307.56582327610

OX4 3,4-epoxybutanol BMK STRUC10

C	-2.2748623872	0.2396990517	0.2871354135
C	-0.9621341680	0.4538653010	-0.3636658604
H	0.1946676167	-0.0091758788	1.3794020296
H	-2.3129339278	0.1187430673	1.3652304653
H	-3.1784100822	0.6118811605	-0.1841803084
O	2.6836731639	-0.1634758652	0.3791819284
H	1.2305539469	-1.2077756146	-0.5322372732
H	1.6048686645	0.3270970333	-1.3220203937
C	0.3273891391	0.5030408580	0.4238276365
O	-1.6171303965	-0.8085392087	-0.4129261055
H	-0.9571095753	0.9755703336	-1.3187899747
C	1.4765765549	-0.1616629072	-0.3476481094
H	0.5793395082	1.5490512567	0.6320780854
H	2.9750691230	0.7383125337	0.5202402904

Energy of Optimized Geometry: -307.562462172042

OX4 3,4-epoxybutanol BMK STRUC12

C	-1.7201129577	-0.2365464513	-0.8133087843
C	-0.7095459622	-0.2766856808	0.2732463439
H	0.6338387609	0.9105032975	1.4463526092
H	-1.6962199296	0.5865588774	-1.5215447729
H	-2.1777813308	-1.1551218610	-1.1653900423
O	2.2736430613	-0.7210681254	0.0563728550
H	1.3677623086	0.3418767619	-1.4746793968
H	2.3450698990	1.2501566425	-0.3183430947
C	0.3612131111	0.7853616896	0.3950716183
O	-2.0479130697	0.1288411650	0.5202827559
H	-0.4718713832	-1.2486972511	0.6997866024
C	1.6209894538	0.4400855890	-0.4119811989
H	-0.0490000526	1.7341276348	0.0379079567
H	1.7934891165	-1.5005562072	-0.2242624390

Energy of Optimized Geometry: -307.561605741775

OX4 3,4-epoxybutanol BMK STRUC13

C	-1.9279230452	0.2435188819	0.6785767336
C	-0.9241377168	0.3619161842	-0.4068561115
H	0.7106936547	-0.6403467218	-1.3711425585
H	-1.7167998641	-0.4086255326	1.5210557525
H	-2.6213070912	1.0562634335	0.8670212697
O	2.7503666513	-0.1622412058	0.2681618957
H	1.6077653570	1.4072293491	-0.2230732049
H	1.1987109318	0.6990327998	1.3402929780
C	0.3923462197	-0.3814108025	-0.3578763913
O	-2.0977866889	-0.4203021497	-0.5665109971
H	-0.9286686750	1.2789369132	-0.9923176044
C	1.4883085835	0.4613767075	0.3090845235
H	0.2461432169	-1.3155154930	0.1936415228
H	2.7129092354	-0.9925408978	0.7451641030

Energy of Optimized Geometry: -307.561868106640

OX4 3,4-epoxybutanol BMK STRUC14

C	-1.3665550899	-0.6857729815	0.5089051304
C	-1.0000968207	0.6719139759	0.0449522626
H	0.7435499108	1.7748495476	0.6165428054
H	-0.5806623229	-1.4077284686	0.6968124283
H	-2.2956090183	-0.8279398159	1.0511495636
O	1.7571439567	-0.6764877183	0.8191826792
H	1.0322907292	-0.7663432857	-1.0553185782
H	2.3518575993	0.3882872657	-0.8529791731
C	0.4272511891	1.1247000097	-0.2065503798
O	-1.5239202830	-0.2951887143	-0.8541508555
H	-1.6977393788	1.4762806734	0.2667869674
C	1.4444385237	-0.0092213528	-0.3864397932
H	0.4270992468	1.7410386767	-1.1119012944
H	2.2112143789	-0.0740309418	1.4098249214

Energy of Optimized Geometry: -307.562788400410

OX4 3,4-epoxybutanol BMK STRUC15

C	-1.8571570100	-0.2378527322	0.5593275138
C	-1.0737830347	0.6580718282	-0.3265016060
H	0.6776779117	1.7670842431	0.2027732986
H	-1.3495948669	-0.8441347702	1.3019800995
H	-2.8949189112	-0.0033453905	0.7723209099
O	2.5657388786	-0.1866127255	0.3761773344
H	0.9410608548	-0.2754906880	1.5405286568
H	0.8298749081	-1.2892029468	0.0949045648
C	0.4336478437	0.8100129952	-0.2656815805
O	-1.5818713398	-0.5863201112	-0.7916930241
H	-1.5992926727	1.5152840002	-0.7404244406
C	1.1651963335	-0.3177364301	0.4738682440
H	0.8052006703	0.8567556018	-1.2960516796
H	2.8363375729	-0.3248745404	-0.5325570172

Energy of Optimized Geometry: -307.561402523625

OX4 3,4-epoxybutanol BMK STRUC16

C	-2.1738826594	0.1915034143	-0.2537041380
C	-0.7222707902	-0.0132042448	-0.4519488479
H	-0.1379973904	1.4632263687	0.9780715975
H	-2.5126748378	1.0525591839	0.3146385493
H	-2.8798997772	-0.2103899392	-0.9728838281
O	2.1738585909	-0.3659543205	-0.5460243090
H	2.2274509626	0.8808169843	1.1094144301
H	1.2594285540	-0.5982168189	1.3035862569
C	0.3027012854	0.9342237969	0.1296909879
O	-1.4580974481	-0.7813439011	0.4980385231
H	-0.3992774742	-0.5821846009	-1.3184806823
C	1.5517817281	0.1867361324	0.5971620669
H	0.5924186368	1.6704950769	-0.6253161868
H	2.8938940020	-0.9379064108	-0.2798864808

Energy of Optimized Geometry: -307.564120872646

OX4 3,4-epoxybutanol BMK STRUC17

C	-1.7559927234	-0.1069951865	-0.8127121483
C	-0.6786204824	-0.3463449668	0.1763212899
H	0.6256624643	0.6568029885	1.5468795832
H	-1.8104768340	0.8565437642	-1.3114906299
H	-2.1993612945	-0.9426368946	-1.3439294515
O	2.1949195549	-0.6811281247	-0.0435011411
H	1.3445637949	0.6109569799	-1.4240542184
H	2.2999825220	1.3876116116	-0.1436353653
C	0.3490631166	0.7192444437	0.4909168645
O	-2.0131407836	-0.0744672834	0.5852974466
H	-0.3626904542	-1.3713445227	0.3432379839
C	1.6086787233	0.5656167837	-0.3599942997
H	-0.0931207971	1.7031443089	0.3161942917
H	2.9885052430	-0.8100097626	-0.5633783552

Energy of Optimized Geometry: -307.563002731912

OX4 3,4-epoxybutanol BMK STRUC18

C	-1.4301113334	-0.9165346638	0.2625202965
C	-0.7994236568	0.4168510719	0.1982475617
H	0.3350521732	1.6970097299	-1.0864665127
H	-1.0099565840	-1.7193903524	-0.3339960393
H	-1.9752039975	-1.2143040258	1.1518165328
O	1.8597886735	-0.7778493309	0.5170314073
H	2.5512703823	0.8647652649	-0.5422845769
H	1.7007553390	1.2388556038	0.9688032369
C	0.4076197243	0.6866380562	-0.6736276881
O	-2.0199615417	0.1484846190	-0.4789300611
H	-0.9101827489	1.0701605670	1.0620470890
C	1.7177382054	0.5648700104	0.1023428905
H	0.4111611769	-0.0173044066	-1.5091545547
H	2.6795823225	-0.8831629032	1.0004450758

Energy of Optimized Geometry: -307.562844545903

OX4 3,4-epoxybutanol BMK STRUC19

C	-2.2606756915	0.2471367927	0.3230642655
C	-0.9563755185	0.4681771630	-0.3407153798
H	0.2181281248	-0.0640388962	1.3694373084
H	-2.2825386757	0.0875841044	1.3965605540
H	-3.1665248960	0.6486976686	-0.1188079188
O	2.6474354350	-0.0918072026	0.3953387305
H	1.2043955935	-1.2158867690	-0.5890721774
H	1.5901290739	0.3502503311	-1.3327239789
C	0.3434839664	0.4767482192	0.4288049706
O	-1.6299142024	-0.7842556483	-0.4257175209
H	-0.9605609636	1.0239646605	-1.2763319635
C	1.4680288109	-0.1741691418	-0.3760542155
H	0.6253823681	1.5066179151	0.6653507248
H	3.3582943435	-0.5533137903	-0.0502045100

Energy of Optimized Geometry: -307.563116989246

OX4 3,4-epoxybutanol BMK STRUC2

C	-1.7092128136	-0.1298499417	0.5931555577
C	-0.9646556725	0.5460234880	-0.4936448926
H	0.2731717531	2.1840572345	0.0804807048
H	-1.2700158889	-0.2129546919	1.5811560403
H	-2.7927502110	-0.1634231651	0.5547563167
O	1.7556344343	-0.8406214066	0.0283628858
H	2.2666082515	0.9624859945	0.7506930556
H	0.9019007617	0.2271250496	1.5832113577
C	0.4079381873	1.1723199892	-0.3154582108
O	-1.0525011464	-0.8812562365	-0.4254208896
H	-1.5624814119	0.9667832910	-1.2979482991
C	1.3575612498	0.3796840202	0.5962010613
H	0.8810244511	1.2665169716	-1.2967367157
H	0.9591014129	-1.3378959274	-0.1816272685

Energy of Optimized Geometry: -307.565934959410

OX4 3,4-epoxybutanol BMK STRUC20

C	-1.8608632156	-0.2171170982	0.5725672461
C	-1.0779531000	0.6346383405	-0.3570843741
H	0.6483752545	1.7849440370	0.1325220624
H	-1.3546683242	-0.7795036335	1.3497525431
H	-2.9015673075	0.0217201242	0.7659786269
O	2.5641610463	-0.2145496326	0.3021424268
H	0.9150607098	-0.2319774120	1.5495783937
H	0.8663107874	-1.2707118342	0.1177500771
C	0.4284203064	0.8023686718	-0.2992907663
O	-1.5741843143	-0.6357737163	-0.7556238992
H	-1.6071657104	1.4644632429	-0.8195091500
C	1.1681807542	-0.2890691654	0.4846084336
H	0.8135972142	0.8074234987	-1.3234443283
H	2.8899353274	0.6157673975	0.6521884679

Energy of Optimized Geometry: -307.560912141950

OX4 3,4-epoxybutanol BMK STRUC21

C	-1.3842260724	-0.6698067750	0.5038914130
C	-0.9851886336	0.6837247813	0.0618708558
H	0.7904856735	1.7008161220	0.6910576993
H	-0.6138956753	-1.4092294049	0.6873563734
H	-2.3183531499	-0.8010505106	1.0402424329
O	1.7316507264	-0.6379174243	0.8362503027
H	1.0113389053	-0.7422230166	-1.1121762792
H	2.3557280975	0.3882896640	-0.8493685327
C	0.4522624479	1.1149431452	-0.1681464962
O	-1.5263528577	-0.2593802560	-0.8555173064
H	-1.6665480842	1.4995522639	0.2916933595
C	1.4425344881	-0.0228771375	-0.4067059999
H	0.4652563943	1.7764988471	-1.0397037648
H	2.3689541582	-1.3406508092	0.7049158194

Energy of Optimized Geometry: -307.563047308367

OX4 3,4-epoxybutanol BMK STRUC22

C	-1.9369627666	0.2239180872	0.6651251152
C	-0.9245703448	0.3368036360	-0.4131689775
H	0.7303498972	-0.6742252735	-1.3316565807
H	-1.7180636361	-0.4001888805	1.5265222166
H	-2.6521632158	1.0241077881	0.8241110232
O	2.6928543482	-0.2030959102	0.2811239611
H	1.5713754719	1.4446055665	-0.2866370504
H	1.1890497847	0.7832366556	1.3146027161
C	0.4060355485	-0.3767934298	-0.3309208072
O	-2.0775007596	-0.4768061823	-0.5632803250
H	-0.9459126862	1.2370370277	-1.0241909438
C	1.4792568801	0.5158146542	0.2909749843
H	0.2940170194	-1.2854056164	0.2647064192
H	3.3891699259	0.3296529349	0.6661979521

Energy of Optimized Geometry: -307.561940544264

OX4 3,4-epoxybutanol BMK STRUC23

C	-1.3799969824	-0.7393394133	0.4502742117
C	-0.9752582347	0.6494429215	0.0996228623
H	0.8014458397	1.7632770558	0.5406620607
H	-0.6450298843	-1.5389023714	0.4745794628
H	-2.2644904968	-0.8996327579	1.0584879404
O	1.8940128456	-0.5679999598	0.8463663674
H	1.0246960069	-0.8503953581	-1.0210714029
H	2.3443542544	0.3126193051	-0.8933569757
C	0.4409901704	1.0841446060	-0.2368203685
O	-1.6195395127	-0.2002592771	-0.8388426713
H	-1.6208287112	1.4430628848	0.4681024796
C	1.4501772264	-0.0584658401	-0.3929307534
H	0.3898080830	1.6543408637	-1.1697577435
H	1.1397681053	-0.8337372453	1.3737561282

Energy of Optimized Geometry: -307.561267857830

OX4 3,4-epoxybutanol BMK STRUC24

C	-1.8701053507	-0.2045468631	0.5490064218
C	-1.0543245400	0.6675529315	-0.3310196435
H	0.7026431088	1.7426914666	0.2374279411
H	-1.3866847462	-0.8163924435	1.3033124170
H	-2.9058152170	0.0528623434	0.7454017548
O	2.5425447921	-0.1667757648	0.2968274716
H	0.9012447815	-0.3273553289	1.5462374768
H	0.8214720526	-1.3054361022	0.0621475872
C	0.4532871834	0.7960603768	-0.2494332810
O	-1.5822976020	-0.5689146008	-0.7952038773
H	-1.5558749260	1.5324686549	-0.7581704675
C	1.1535947769	-0.3485088832	0.4795134010
H	0.8520864076	0.8452398846	-1.2670219290
H	3.0189262861	-0.8899913919	0.7052046440

Energy of Optimized Geometry: -307.561487968348

OX4 3,4-epoxybutanol BMK STRUC25

C	-2.1631082163	0.1789651142	0.1342826139
C	-0.7901455089	0.2868224582	-0.4138268643
H	0.2392788323	0.6246036159	1.4248499192
H	-2.3575317257	0.5399806505	1.1400968368
H	-3.0149669108	0.1617997200	-0.5377807021
O	1.8963510923	-0.8184105688	-0.0033729365
H	1.7950990902	0.8699485119	-1.1998583123
H	2.4748374895	1.1269902733	0.4188337029
C	0.3279948074	0.9318499761	0.3797708184
O	-1.3035316845	-0.9483232401	0.0556295340
H	-0.6843920249	0.3317113852	-1.4965268178
C	1.7165749510	0.5711600538	-0.1457498643
H	0.2164949492	2.0196258022	0.3326576672
H	2.7422504192	-1.0774480889	-0.3695843273

Energy of Optimized Geometry: -307.559961061107

OX4 3,4-epoxybutanol BMK STRUC3

C	-1.7651546460	-0.0860853606	-0.8524736488
C	-0.6900287068	-0.3555372949	0.1307434800
H	0.5455756193	0.6316136877	1.5748132367
H	-1.8347192605	0.9001954038	-1.3018810474
H	-2.1883000408	-0.9023059597	-1.4282562932
O	2.2762909990	-0.6485753309	-0.0788378948
H	1.3920436124	0.6025654886	-1.3715066309
H	2.2769541212	1.4245121958	-0.0832859420
C	0.3184099244	0.7081762867	0.5055199763
O	-2.0311031201	-0.1298274924	0.5443967387
H	-0.3574075970	-1.3831789754	0.2432304438
C	1.6162975471	0.5787540311	-0.3032789421
H	-0.1230235778	1.6946047099	0.3389990633
H	2.5947847081	-0.6791223933	0.8245985979

Energy of Optimized Geometry: -307.562623259960

OX4 3,4-epoxybutanol BMK STRUC4

C	-1.4826074222	-0.9021673189	0.1793574162
C	-0.7842849965	0.3966320592	0.2715097933
H	0.3422202486	1.7955798277	-0.8873889216
H	-1.1361337803	-1.6351203070	-0.5426560006
H	-2.0069379733	-1.2967962422	1.0429688520
O	1.9439679574	-0.8572323383	0.4493590714
H	2.5594817212	0.8692786695	-0.5177652309
H	1.7681238804	1.0459656813	1.0480046962
C	0.4010744827	0.7400087084	-0.6031618625
O	-2.0417760333	0.2803163752	-0.3821759328
H	-0.8307639753	0.9235111366	1.2226748676
C	1.7399006546	0.4947304406	0.1055680297
H	0.3474242460	0.1543207753	-1.5265803328
H	2.0081462729	-1.3831703355	-0.3491371954

Energy of Optimized Geometry: -307.562298367616

OX4 3,4-epoxybutanol BMK STRUC5

C	-1.4389035130	-0.9122267700	0.2443016115
C	-0.7895148116	0.4269682501	0.2442111431
H	0.3345206584	1.7853566102	-0.9633254164
H	-1.0584035173	-1.6779143020	-0.4250918328
H	-1.9596395295	-1.2628585518	1.1295402495
O	1.9603696523	-0.8358170599	0.4003369297
H	2.5536798510	0.8230131956	-0.5564883094
H	1.7671583131	1.1399751829	0.9906351900
C	0.3957239527	0.7409165035	-0.6427703775
O	-2.0323646004	0.2054966949	-0.3983961637
H	-0.8683892846	1.0197824827	1.1544745110
C	1.7279238610	0.5183445820	0.0863474318
H	0.3535541247	0.1111977264	-1.5345267548
H	1.2676159798	-1.1508266879	0.9825760079

Energy of Optimized Geometry: -307.562830821011

OX4 3,4-epoxybutanol BMK STRUC6

C	-2.1681927268	0.3050398673	-0.1241085325
C	-0.7386133843	0.1474808961	-0.4722749392
H	-0.0435285104	1.4608860218	1.0689547498
H	-2.4494878427	1.0496651531	0.6140884393
H	-2.9336248859	0.0350761993	-0.8441356625
O	2.1568617176	-0.5347117356	-0.5290244549
H	2.3223527910	0.7273221180	1.0310256751
H	1.2348379330	-0.6353051473	1.3248414493
C	0.3522544077	0.9697843657	0.1757229495
O	-1.4042216360	-0.7831736553	0.3810477491
H	-0.5089232729	-0.2547020316	-1.4561305928
C	1.5534635767	0.0995343841	0.5787217037
H	0.6841812149	1.7415009508	-0.5239904109
H	1.6690331210	-1.3289281119	-0.7481633507

Energy of Optimized Geometry: -307.563392203815

OX4 3,4-epoxybutanol BMK STRUC7

C	-2.2767833154	0.2615081017	0.2634685279
C	-0.9551874250	0.4538104455	-0.3752902669
H	0.1694921004	-0.0142295150	1.3764625201
H	-2.3281826564	0.1568484676	1.3427901896
H	-3.1712217544	0.6364745845	-0.2226435690
O	2.7006116872	-0.0976943963	0.3661212181
H	1.2160097888	-1.2007062704	-0.5774416801
H	1.6529764041	0.3587406517	-1.2814919560
C	0.3268575681	0.5005263622	0.4236386410
O	-1.6229543713	-0.8037236969	-0.4146716445
H	-0.9343263181	0.9623268088	-1.3371055057
C	1.4819450139	-0.1651064238	-0.3388545147
H	0.5927457562	1.5388579797	0.6400530537
H	2.6447224904	-0.6431165636	1.1518396688

Energy of Optimized Geometry: -307.562909366847

OX4 3,4-epoxybutanol BMK STRUC8

C	-2.1907217916	0.1524958022	-0.2440360955
C	-0.7366709376	-0.0501886360	-0.4296154294
H	-0.1544725026	1.4603401531	0.9622337753
H	-2.5371055434	1.0366373063	0.2828320702
H	-2.8922123770	-0.2886741606	-0.9443268904
O	2.2360991969	-0.4038152068	-0.5208084249
H	2.2267411285	0.9271001672	1.0686887907
H	1.3172649048	-0.5768812465	1.2569201750
C	0.2798996705	0.9304543245	0.1100473719
O	-1.4678032409	-0.7776962642	0.5530035174
H	-0.4077080420	-0.6582370548	-1.2675840377
C	1.5670351406	0.2196893598	0.5545200817
H	0.5191331895	1.6728880257	-0.6596059872
H	2.5997722125	0.2655535451	-1.1019548970

Energy of Optimized Geometry: -307.563063417756

OX4 3,4-epoxybutanol BMK STRUC9

C	-1.9379630070	0.1345148149	0.6931967973
C	-0.9296803166	0.3881759305	-0.3641983808
H	0.6963687888	-0.5111285223	-1.4232264422
H	-1.7196065781	-0.6030047628	1.4597096511
H	-2.6463655311	0.9102218985	0.9639027392
O	2.7304976595	-0.1983854776	0.3472925076
H	1.5817374795	1.4622059092	-0.1153463196
H	1.2149167794	0.6066091731	1.3832558745
C	0.3973156616	-0.3378259875	-0.3834122010
O	-2.0886141498	-0.3902254962	-0.6194806711
H	-0.9463132023	1.3639786401	-0.8453878928
C	1.4888235765	0.4661444685	0.3362135328
H	0.2842819805	-1.3135080007	0.0942225368
H	3.0344241785	-0.3251463691	-0.5526477964

Energy of Optimized Geometry: -307.561848701697

6.2 CAM-B3LYP

OX4 3,4-epoxybutanol CAM-B3LYP STRUC1

C	-2.0426902498	-0.0475268968	0.2856987685
C	-0.8686069839	0.4674679104	-0.4083534688
H	0.2215729909	0.8966585027	1.3802633235
H	-2.0882053954	0.0192907531	1.3663156268
H	-2.9977051826	-0.0894560047	-0.2234264132
O	1.8226750710	-0.8277950561	0.2073053861
H	1.6860571379	0.6090156439	-1.2789862182
H	2.4542989136	1.0829980114	0.2331578142
C	0.3017424027	1.0772204181	0.3074566524
O	-1.0575615481	-0.9419379300	-0.2217216077
H	-0.9929439073	0.7716018605	-1.4434565897
C	1.6263517315	0.5141760084	-0.1877780933
H	0.2827992451	2.1578620913	0.1494675892
H	0.9980502262	-1.3096744283	0.0808971695

Energy of Optimized Geometry: -307.618858229977

OX4 3,4-epoxybutanol CAM-B3LYP STRUC2

C	-1.6811541826	-0.1650597209	0.5838127879
C	-0.9680005160	0.5420458986	-0.4750115664
H	0.2492120194	2.1843156330	0.0776378167
H	-1.2302486130	-0.2799592915	1.5612136174
H	-2.7634711810	-0.2026422825	0.5644846303
O	1.7457663013	-0.8287188942	0.0536495684
H	2.2576761579	0.9978170015	0.7237072089
H	0.9155559174	0.2798166210	1.5944846301
C	0.3898360565	1.1709804317	-0.3077005722
O	-1.0401991801	-0.8885845494	-0.4659703928
H	-1.5790957761	0.9863759029	-1.2543333306
C	1.3486695362	0.4120130495	0.5967057360
H	0.8525640931	1.2674173045	-1.2915621930
H	0.9428880461	-1.3154310450	-0.1684141618

Energy of Optimized Geometry: -307.618783030993

OX4 3,4-epoxybutanol CAM-B3LYP STRUC3

C	-1.7787842872	-0.0585204577	-0.8070274056
C	-0.6958561104	-0.3382816390	0.1302010904
H	0.5725316391	0.6209285502	1.5436549877
H	-1.8487911371	0.9247532423	-1.2591778170
H	-2.2288968591	-0.8658697339	-1.3721989488
O	2.2699554274	-0.6859768006	-0.1146902076
H	1.3836604159	0.5739086488	-1.4001963291
H	2.2822473697	1.3886476899	-0.1255245344
C	0.3350427919	0.6953292514	0.4785062189
O	-2.0185502829	-0.0940653289	0.5951250525
H	-0.3772885318	-1.3693784425	0.2342713775
C	1.6105962498	0.5518012910	-0.3347773117
H	-0.0874061949	1.6893058518	0.3198105932
H	2.6126897334	-0.7071762774	0.7820105491

Energy of Optimized Geometry: -307.616025606669

OX4 3,4-epoxybutanol CAM-B3LYP STRUC4

C	-1.4621693796	-0.9210458406	0.2135636740
C	-0.7849938724	0.3659953994	0.2836577815
H	0.2717136135	1.7740853901	-0.9025767486
H	-1.1278069368	-1.6554780896	-0.5099967335
H	-1.9584116019	-1.3205192336	1.0897460728
O	1.9869478826	-0.8107709072	0.4139823604
H	2.5089847172	0.9331633006	-0.5803321633
H	1.7556177225	1.0929083992	0.9992700644
C	0.3678297867	0.7261252364	-0.6068286827
O	-2.0573402626	0.2469788320	-0.3421025019
H	-0.8149501614	0.8936491578	1.2334270528
C	1.7184274331	0.5367442870	0.0625240403
H	0.3128469416	0.1351507880	-1.5241157529
H	2.0850644183	-1.3330508963	-0.3855958673

Energy of Optimized Geometry: -307.615743008158

OX4 3,4-epoxybutanol CAM-B3LYP STRUC5

C	-1.4644164912	-0.8823229213	0.2298362244
C	-0.7832207340	0.4124524720	0.2506632733
H	0.3385546631	1.7587329937	-0.9456132394
H	-1.1135218202	-1.6508749125	-0.4491952647
H	-1.9894631697	-1.2349323154	1.1100880022
O	1.9799305409	-0.8615037317	0.3893633072
H	2.5421001917	0.8122204288	-0.5625654558
H	1.7755076203	1.1117747319	0.9909681469
C	0.3981126598	0.7159677702	-0.6243629684
O	-2.0374619911	0.2574219121	-0.3959674482
H	-0.8407825747	0.9907689021	1.1700902222
C	1.7249263127	0.4995099356	0.0839091147
H	0.3532271481	0.0946641898	-1.5191588858
H	1.3310138539	-1.1729550376	1.0240652283

Energy of Optimized Geometry: -307.615869377416

OX4 3,4-epoxybutanol CAM-B3LYP STRUC6

C	-2.1509981716	0.3398436395	-0.1363292537
C	-0.7418468849	0.1374843171	-0.4498854481
H	-0.0307510002	1.4124681302	1.0976702636
H	-2.4272794634	1.0983747904	0.5871593151
H	-2.9106423145	0.0926193118	-0.8683509438
O	2.1759059368	-0.5162114189	-0.5612469486
H	2.3130321774	0.7007731817	1.0412491756
H	1.2548714003	-0.6852782054	1.2903854580
C	0.3532071572	0.9333026745	0.1949684402
O	-1.4428112069	-0.7723712954	0.3988300284
H	-0.5072123406	-0.2782252917	-1.4249410399
C	1.5543099897	0.0783750326	0.5693195947
H	0.6738039764	1.7195407678	-0.4913595105
H	1.7315628236	-1.3388652554	-0.7744270033

Energy of Optimized Geometry: -307.616734327957

OX4 3,4-epoxybutanol CAM-B3LYP STRUC7

C	-2.2521724200	0.2927228742	0.2809963913
C	-0.9529261359	0.4491263238	-0.3614415925
H	0.1808325599	-0.0421727713	1.3630051084
H	-2.3001039707	0.1912415532	1.3592221265
H	-3.1414318205	0.6924393929	-0.1915169523
O	2.7019672423	-0.1034645381	0.3474524470
H	1.2167619879	-1.2029559539	-0.6004557111
H	1.6393764306	0.3599876992	-1.2905608113
C	0.3296437702	0.4779398383	0.4141380481
O	-1.6435320929	-0.7975893549	-0.4009816449
H	-0.9308763368	0.9573311148	-1.3217446060
C	1.4713533102	-0.1692737982	-0.3529005311
H	0.5964019778	1.5116413372	0.6432004313
H	2.6593009874	-0.6754381820	1.1171002361

Energy of Optimized Geometry: -307.616450525094

OX4 3,4-epoxybutanol CAM-B3LYP STRUC8

C	-2.1744908769	0.1308975846	-0.2408440712
C	-0.7408678209	-0.0718993544	-0.4097852938
H	-0.1599360926	1.4651093552	0.9342948480
H	-2.5285896426	1.0271303115	0.2565687562
H	-2.8718668434	-0.3244627516	-0.9339772387
O	2.2395182866	-0.3869839303	-0.5385535645
H	2.2140937105	0.9857385894	1.0172894568
H	1.3465768876	-0.5283812846	1.2569343461
C	0.2674675663	0.9188746529	0.0913039329
O	-1.4674241704	-0.7803642176	0.5920368944
H	-0.4093877964	-0.7006135058	-1.2289311654
C	1.5622046795	0.2546124636	0.5318518721
H	0.4814452285	1.6491364832	-0.6949093081
H	2.6010532185	0.2780131551	-1.1289659799

Energy of Optimized Geometry: -307.616497445574

OX4 3,4-epoxybutanol CAM-B3LYP STRUC9

C	-1.9409702495	0.1151469669	0.6625500111
C	-0.9266874352	0.3866560977	-0.3517042927
H	0.6989808755	-0.4886675713	-1.4014032708
H	-1.7394268164	-0.6299051052	1.4240014899
H	-2.6571743117	0.8815700837	0.9337026064
O	2.7254613203	-0.1956877923	0.3674447927
H	1.5770699556	1.4685023443	-0.0885296766
H	1.2084789191	0.6098798610	1.4015760078
C	0.3970396385	-0.3202003365	-0.3635956770
O	-2.0725927933	-0.3957137893	-0.6585461925
H	-0.9392116643	1.3688889989	-0.8164043830
C	1.4766785489	0.4721136110	0.3549557842
H	0.2894703534	-1.2992769095	0.1031829449
H	3.0349827052	-0.3087078893	-0.5344482498

Energy of Optimized Geometry: -307.615332515913

OX4 3,4-epoxybutanol CAM-B3LYP STRUC10

C	-2.2549124226	0.2492170202	0.2885412920
C	-0.9618315256	0.4402928406	-0.3576802577
H	0.1970685922	-0.0283713677	1.3669686165
H	-2.2954922087	0.1339538625	1.3655911119
H	-3.1545354853	0.6349594631	-0.1760805294
O	2.6834780963	-0.1459967886	0.3794744545
H	1.2440728300	-1.1996658931	-0.5439599933
H	1.5990399760	0.3396924137	-1.3208775325
C	0.3227396485	0.4868305759	0.4145380397
O	-1.6255597818	-0.8192242057	-0.4082366060
H	-0.9548543572	0.9590816156	-1.3126897634
C	1.4695605774	-0.1527818004	-0.3511516937
H	0.5647834623	1.5314018618	0.6335393692
H	2.9705931982	0.7603683363	0.5124803949

Energy of Optimized Geometry: -307.616002487571

OX4 3,4-epoxybutanol CAM-B3LYP STRUC12

C	-1.7462233972	-0.2204673413	-0.7782238140
C	-0.7105228928	-0.2762947822	0.2506067591
H	0.6328646128	0.9008873091	1.4068676005
H	-1.7527084297	0.6084093791	-1.4774365565
H	-2.2154426359	-1.1318632638	-1.1296362790
O	2.2931029721	-0.7130659321	0.0524282690
H	1.3666599991	0.3085894812	-1.4966915563
H	2.3213755429	1.2535810000	-0.3610776773
C	0.3602597497	0.7693546939	0.3584082203
O	-2.0359435688	0.1276752834	0.5694947891
H	-0.4579559032	-1.2539863477	0.6505998881
C	1.6110565099	0.4324026885	-0.4372834977
H	-0.0443514366	1.7192966998	0.0038477171
H	1.8481841154	-1.5085976244	-0.2454430870

Energy of Optimized Geometry: -307.614912606875

OX4 3,4-epoxybutanol CAM-B3LYP STRUC13

C	-1.9322357847	0.2368934443	0.6443391998
C	-0.9199461284	0.3562117606	-0.4010005341
H	0.7134903626	-0.6406869461	-1.3386729572
H	-1.7408033762	-0.4079658883	1.4949797735
H	-2.6318800003	1.0456425538	0.8184207157
O	2.7460353294	-0.1561754658	0.2905181290
H	1.6046718866	1.4092981747	-0.2198212328
H	1.1942309861	0.7248232613	1.3474247262
C	0.3918733767	-0.3690093804	-0.3319450015
O	-2.0826828407	-0.4370066945	-0.5987786757
H	-0.9172812637	1.2706622744	-0.9878409228
C	1.4770835807	0.4717490714	0.3205995352
H	0.2489385939	-1.2981388372	0.2246016829
H	2.7078385357	-0.9747092140	0.7909235089

Energy of Optimized Geometry: -307.615321570217

OX4 3,4-epoxybutanol CAM-B3LYP STRUC14

C	-1.3666165742	-0.6955841448	0.4771105240
C	-1.0051305765	0.6485849580	0.0408681597
H	0.6954565399	1.7575821732	0.6734156099
H	-0.5849189357	-1.4160906318	0.6755490965
H	-2.3016341654	-0.8506281809	1.0027038893
O	1.7565972473	-0.6764512952	0.8283622420
H	1.0785549697	-0.7111948556	-1.0670515422
H	2.3555212355	0.4663031342	-0.7923048287
C	0.4084989480	1.1226629968	-0.1698012324
O	-1.5055059871	-0.3046264517	-0.8882278190
H	-1.7115281440	1.4446671068	0.2583939630
C	1.4483096645	0.0328827755	-0.3641031410
H	0.4145268909	1.7671863745	-1.0530106708
H	2.2003060542	-0.0866836088	1.4420390917

Energy of Optimized Geometry: -307.615585402532

OX4 3,4-epoxybutanol CAM-B3LYP STRUC15

C	-1.8588896650	-0.2387873177	0.5369124237
C	-1.0730728510	0.6304478710	-0.3354229132
H	0.6446474101	1.7540156088	0.2109226677
H	-1.3691152311	-0.8357586576	1.2961251030
H	-2.8966229601	0.0011791284	0.7364212821
O	2.5692593395	-0.1309652397	0.4082985478
H	0.9229634098	-0.2888177121	1.5366047193
H	0.8742971733	-1.2851397356	0.0832163394
C	0.4197362615	0.7949300069	-0.2591747909
O	-1.5730358144	-0.6178870461	-0.8045451522
H	-1.5910391051	1.4822082610	-0.7660670332
C	1.1636003657	-0.3073019135	0.4751251011
H	0.7994032922	0.8564785340	-1.2835351856
H	2.8625331425	-0.2688755033	-0.4953511620

Energy of Optimized Geometry: -307.614357895667

OX4 3,4-epoxybutanol CAM-B3LYP STRUC16

C	-2.1573481418	0.1838802426	-0.2612049297
C	-0.7260541663	-0.0316290458	-0.4311955108
H	-0.1426206521	1.4415403662	0.9830581641
H	-2.5013335673	1.0545746043	0.2860121130
H	-2.8559017999	-0.2179239275	-0.9855270979
O	2.1843413782	-0.3398675176	-0.5682529423
H	2.2161652688	0.9130373326	1.0849507740
H	1.2935629110	-0.5859901639	1.2926419435
C	0.2920402861	0.9142527861	0.1328371757
O	-1.4726819478	-0.7872331107	0.5221677956
H	-0.3974467033	-0.6144535503	-1.2840807594
C	1.5514341682	0.2046112807	0.5828140115
H	0.5562103838	1.6564336538	-0.6234623918
H	2.9190553582	-0.8942651088	-0.2990306822

Energy of Optimized Geometry: -307.617614341947

OX4 3,4-epoxybutanol CAM-B3LYP STRUC17

C	-1.7444506099	-0.1197714289	-0.8127194229
C	-0.6819537061	-0.3467612754	0.1617498333
H	0.6015677642	0.6485918890	1.5360750713
H	-1.8066175200	0.8383042855	-1.3174269723
H	-2.1812002938	-0.9566536905	-1.3446482340
O	2.2102971338	-0.6651655094	-0.0297677981
H	1.3512161260	0.6176020662	-1.4155964042
H	2.2718952225	1.4070931126	-0.1253876729
C	0.3370169453	0.7090827617	0.4789084355
O	-2.0156885731	-0.0820212014	0.5833286669
H	-0.3635142079	-1.3688809499	0.3303393533
C	1.5967442602	0.5766062270	-0.3493348544
H	-0.1055582001	1.6915221944	0.3085638156
H	3.0094491449	-0.7727371310	-0.5491587035

Energy of Optimized Geometry: -307.616458562697

OX4 3,4-epoxybutanol CAM-B3LYP STRUC18

C	-1.4470723108	-0.9047091761	0.2351678132
C	-0.7949547781	0.3958153375	0.1946854113
H	0.3313148187	1.6701472698	-1.0776591758
H	-1.0475567451	-1.7095870399	-0.3696083128
H	-1.9972864171	-1.2071432127	1.1181159029
O	1.8753941892	-0.7796040295	0.5404430320
H	2.5394666935	0.8636055127	-0.5387705439
H	1.6935968814	1.2421184797	0.9675183575
C	0.4102493412	0.6643837324	-0.6588502021
O	-2.0204758436	0.1775321065	-0.4936126565
H	-0.8948524932	1.0346520440	1.0687731803
C	1.7105209537	0.5633019158	0.1078180368
H	0.4230437684	-0.0356764540	-1.4946760503
H	2.6986085027	-0.8613292046	1.0259314884

Energy of Optimized Geometry: -307.616415205311

OX4 3,4-epoxybutanol CAM-B3LYP STRUC19

C	-2.2414130698	0.2646450742	0.3176372363
C	-0.9539457034	0.4553741196	-0.3388789964
H	0.2156430899	-0.0797151498	1.3580905035
H	-2.2697664184	0.1149298309	1.3908182487
H	-3.1400688397	0.6812067594	-0.1215768994
O	2.6470889062	-0.0789238765	0.4018418002
H	1.2178260927	-1.2150794922	-0.5904862872
H	1.5865563466	0.3517222470	-1.3285503392
C	0.3386487438	0.4600787700	0.4190361023
O	-1.6407075917	-0.7915035492	-0.4224660226
H	-0.9512079756	1.0048810136	-1.2764969350
C	1.4622193833	-0.1718542168	-0.3743578449
H	0.6127639700	1.4885166408	0.6635562746
H	3.3602131833	-0.5371325176	-0.0470875482

Energy of Optimized Geometry: -307.616812513709

OX4 3,4-epoxybutanol CAM-B3LYP STRUC20

C	-1.8694588679	-0.1926876997	0.5390291458
C	-1.0656850363	0.6270917446	-0.3648704361
H	0.6407528902	1.7611784732	0.1657915623
H	-1.3943663437	-0.7576563220	1.3312987674
H	-2.9075958827	0.0649307289	0.7132360053
O	2.5660646210	-0.2027464454	0.3392133910
H	0.8877068381	-0.2779117235	1.5466386922
H	0.8880922583	-1.2801402086	0.0959879642
C	0.4279457998	0.7852857705	-0.2806533375
O	-1.5701203063	-0.6370312472	-0.7792349331
H	-1.5713980881	1.4616400419	-0.8417177737
C	1.1593835505	-0.3021342725	0.4874969082
H	0.8275190971	0.8123585033	-1.2965042408
H	2.8711272086	0.6183052423	0.7322013316

Energy of Optimized Geometry: -307.613876431830

OX4 3,4-epoxybutanol CAM-B3LYP STRUC21

C	-1.3937644533	-0.6709335220	0.4615014853
C	-0.9884807732	0.6667114378	0.0483285349
H	0.7415147788	1.6777562810	0.7607706155
H	-0.6335833204	-1.4118149063	0.6672490374
H	-2.3419771049	-0.8080146998	0.9680444998
O	1.7154763510	-0.6559837471	0.8574688745
H	1.0686324401	-0.6809526684	-1.1200430461
H	2.3714328381	0.4618604141	-0.7582777240
C	0.4394667520	1.1124942271	-0.1231064535
O	-1.4967351912	-0.2622859181	-0.9025144242
H	-1.6781783717	1.4779403172	0.2630302220
C	1.4520165316	0.0162672556	-0.3706006258
H	0.4703625833	1.8053197050	-0.9672924075
H	2.3736335937	-1.3381864511	0.7094512697

Energy of Optimized Geometry: -307.616019082960

OX4 3,4-epoxybutanol CAM-B3LYP STRUC22

C	-1.9392385521	0.2231580677	0.6385856011
C	-0.9206088386	0.3367177008	-0.4015358634
H	0.7218397949	-0.6793613655	-1.3004785534
H	-1.7412478198	-0.3976441219	1.5053221452
H	-2.6563001092	1.0217977983	0.7874157091
O	2.6893884184	-0.2099873440	0.2952974650
H	1.5765429675	1.4373886484	-0.2937702317
H	1.1945951757	0.8062281903	1.3140232723
C	0.4015948334	-0.3652229130	-0.3057102418
O	-2.0663044678	-0.4825984165	-0.5893932351
H	-0.9316345152	1.2367067650	-1.0106103597
C	1.4731134790	0.5196105870	0.2948976714
H	0.2905185243	-1.2664327848	0.2972721717
H	3.3867737580	0.3285430406	0.6745719432

Energy of Optimized Geometry: -307.615552994577

OX4 3,4-epoxybutanol CAM-B3LYP STRUC23

C	-1.3875325973	-0.7187883893	0.4460328714
C	-0.9751920501	0.6401315792	0.0923665670
H	0.7852777807	1.7435659857	0.5537504071
H	-0.6627442234	-1.5247732382	0.4844420448
H	-2.2722864287	-0.8699234212	1.0536182198
O	1.9108889314	-0.5632446619	0.8560397178
H	1.0388128737	-0.8516714691	-1.0107657084
H	2.3344502084	0.3278773774	-0.8853864440
C	0.4338218521	1.0665041415	-0.2269976275
O	-1.6229944315	-0.2020038568	-0.8545685715
H	-1.6143637671	1.4422162801	0.4492032393
C	1.4468492629	-0.0526780495	-0.3850619997
H	0.3918727374	1.6463407556	-1.1523192064
H	1.1696150122	-0.9034833829	1.3611685389

Energy of Optimized Geometry: -307.613876738180

OX4 3,4-epoxybutanol CAM-B3LYP STRUC24

C	-1.8886233181	-0.2566618327	0.4406004192
C	-1.0559486768	0.6078655664	-0.3917215931
H	0.6032986984	1.7503897268	0.2742692552
H	-1.4434002679	-0.8384327051	1.2383018349
H	-2.9391774702	-0.0242282939	0.5706164100
O	2.5316134442	-0.0367019722	0.4458185187
H	0.8219783228	-0.3140190054	1.5796304102
H	0.9001804163	-1.2735229382	0.0905530463
C	0.4256653133	0.7954187417	-0.2241513328
O	-1.5141610108	-0.6514161025	-0.8742153777
H	-1.5535678537	1.4483341008	-0.8661605021
C	1.1387905029	-0.3031848326	0.5325658413
H	0.8771893170	0.8739832614	-1.2151689554
H	3.0185972758	-0.7442384642	0.8726970072

Energy of Optimized Geometry: -307.614603705868

OX4 3,4-epoxybutanol CAM-B3LYP STRUC25

C	-2.1477321571	0.1977618640	0.1307406150
C	-0.7952339104	0.2913714536	-0.4085907243
H	0.2405055544	0.6141993585	1.4166861505
H	-2.3420748029	0.5533627213	1.1368543692
H	-3.0005452200	0.2015208216	-0.5381047215
O	1.9103574714	-0.8243115011	0.0110501346
H	1.7792275607	0.8503518677	-1.2044297543
H	2.4563696254	1.1375087774	0.4042569219
C	0.3256372773	0.9174993514	0.3725071953
O	-1.3136692881	-0.9491161472	0.0481171876
H	-0.6917584349	0.3436080721	-1.4896121515
C	1.7028307175	0.5677228337	-0.1482118152
H	0.2164085798	2.0044359202	0.3329320963
H	2.7607645455	-1.0677165486	-0.3599442647

Energy of Optimized Geometry: -307.613679152586

6.3 M06-2X

OX4 3,4-epoxybutanol M06-2X STRUC1

C	-2.0484106904	-0.0073084735	0.2908584357
C	-0.8555679523	0.4772709439	-0.4049820786
H	0.2428143542	0.8603724616	1.3878013594
H	-2.0924432907	0.0746215968	1.3704700509
H	-2.9997466499	-0.0364849391	-0.2251941470
O	1.8102144034	-0.8602211150	0.1798831404
H	1.6819194855	0.5965261864	-1.2882924085
H	2.4771722863	1.0387297009	0.2223894635
C	0.3227327264	1.0662613592	0.3186774922
O	-1.0779000428	-0.9191216591	-0.2013469463
H	-0.9670622512	0.7736120923	-1.4439875914
C	1.6358543476	0.4884387742	-0.1974093039
H	0.3156076353	2.1493730474	0.1795426110
H	0.9759935477	-1.3245998514	0.0528479602

Energy of Optimized Geometry: -307.631404226493

OX4 3,4-epoxybutanol M06-2X STRUC2

C	-1.7121319153	-0.0906949383	0.5814781787
C	-0.9525632638	0.5430398505	-0.4990091000
H	0.2753029560	2.1681210026	0.1005552408
H	-1.2904569570	-0.1452858592	1.5773194075
H	-2.7932173650	-0.1178443945	0.5256441687
O	1.7583517151	-0.8477651030	0.0034650138
H	2.2388477616	0.9400664629	0.7897023475
H	0.8663700161	0.1723002270	1.5707221870
C	0.4096327595	1.1618626953	-0.3040811396
O	-1.0463385922	-0.8766984156	-0.3981858750
H	-1.5323255001	0.9459542633	-1.3230543300
C	1.3375933360	0.3591030577	0.5993107723
H	0.8945659369	1.2658432290	-1.2765236484
H	0.9648953972	-1.3565395104	-0.1970527145

Energy of Optimized Geometry: -307.631671101130

OX4 3,4-epoxybutanol M06-2X STRUC3

C	-1.7418376218	-0.0699722211	-0.8257184737
C	-0.6869125887	-0.3444176188	0.1527325195
H	0.5822102006	0.6213470986	1.5701735899
H	-1.7882270508	0.9106742674	-1.2866765237
H	-2.1761013098	-0.8828102482	-1.3944639300
O	2.2448497795	-0.6843693236	-0.1225494234
H	1.3516163160	0.5950604770	-1.3859302493
H	2.2802178518	1.3905905816	-0.1154497206
C	0.3328582953	0.7003647210	0.5075848198
O	-2.0192241778	-0.0915244426	0.5657776407
H	-0.3716728988	-1.3747164301	0.2750241950
C	1.5987180715	0.5614754565	-0.3244692595
H	-0.1046928500	1.6890641012	0.3540242474
H	2.5655714916	-0.7256587120	0.7817477983

Energy of Optimized Geometry: -307.628205102024

OX4 3,4-epoxybutanol M06-2X STRUC4

C	-1.4244845187	-0.9112465519	0.2194416347
C	-0.7792193466	0.3984221064	0.2815870716
H	0.3045871539	1.8093059279	-0.8908181634
H	-1.0583260263	-1.6402728739	-0.4945965247
H	-1.9191727482	-1.3124802018	1.0951042344
O	1.9262293570	-0.8409639756	0.4118706618
H	2.5279180938	0.8881046896	-0.5641316208
H	1.7648871653	1.0661960879	1.0121850018
C	0.3773947902	0.7563502433	-0.6073404058
O	-2.0372735629	0.2348751674	-0.3511170048
H	-0.8274826581	0.9356138239	1.2251128230
C	1.7181845838	0.5177967911	0.0706452871
H	0.3097769620	0.1715504333	-1.5290745448
H	1.9475839813	-1.3638863115	-0.3933119675

Energy of Optimized Geometry: -307.628031434118

OX4 3,4-epoxybutanol M06-2X STRUC5

C	-1.4079550645	-0.9017050570	0.2423556246
C	-0.7799569404	0.4274739127	0.2479509073
H	0.3318144971	1.7949648783	-0.9464127938
H	-1.0190769966	-1.6570501471	-0.4316244172
H	-1.9175448979	-1.2651394482	1.1267764900
O	1.9301794939	-0.8497617089	0.3909875045
H	2.5429476566	0.8084374634	-0.5625863840
H	1.7672475573	1.1293134615	0.9858272455
C	0.3925952957	0.7495507852	-0.6352087343
O	-2.0221784622	0.2065833598	-0.3906553059
H	-0.8568387280	1.0119020355	1.1619900609
C	1.7150769107	0.5146724649	0.0797426114
H	0.3428183385	0.1295004262	-1.5317157952
H	1.2241244959	-1.1505221912	0.9683174477

Energy of Optimized Geometry: -307.628753435256

OX4 3,4-epoxybutanol M06-2X STRUC6

C	-2.1487827705	0.2636938919	-0.0748031787
C	-0.7405229318	0.1578148525	-0.4578589486
H	-0.0557609509	1.4545272478	1.0889016832
H	-2.4295606984	0.9778563284	0.6907220205
H	-2.9235034858	-0.0039779957	-0.7828253490
O	2.1142115266	-0.5123890330	-0.5720653362
H	2.3250899940	0.7564779573	0.9816364029
H	1.2510079689	-0.6048627636	1.3116242491
C	0.3370033235	0.9833069275	0.1853912400
O	-1.3502618206	-0.8176783985	0.3801093843
H	-0.5249731505	-0.2057210010	-1.4580935588
C	1.5429571204	0.1291642973	0.5565398756
H	0.6466765513	1.7704547741	-0.5044433618
H	1.6046886280	-1.3004559014	-0.7727718233

Energy of Optimized Geometry: -307.629097771538

OX4 3,4-epoxybutanol M06-2X STRUC7

C	-2.2568149522	0.2767339221	0.2474548502
C	-0.9475132842	0.4571663394	-0.3808910216
H	0.1573802223	-0.0040050726	1.3709455761
H	-2.3112866109	0.1831378472	1.3260226811
H	-3.1466635273	0.6520291905	-0.2427645962
O	2.6855878997	-0.1156605665	0.3843966829
H	1.1918841562	-1.2037476827	-0.5670749155
H	1.6486881588	0.3449082531	-1.2731561180
C	0.3232406744	0.5027719829	0.4166089823
O	-1.6121549389	-0.7980662581	-0.4167244997
H	-0.9208878183	0.9589615204	-1.3444045816
C	1.4653829192	-0.1715421985	-0.3305144783
H	0.5950021411	1.5384664294	0.6296034433
H	2.6062758656	-0.6505636862	1.1775835452

Energy of Optimized Geometry: -307.628365739973

OX4 3,4-epoxybutanol M06-2X STRUC8

C	-2.1732097806	0.1193863905	-0.2240566500
C	-0.7341203008	-0.0577041841	-0.4211732051
H	-0.1754521651	1.4573700589	0.9580865206
H	-2.5265690697	0.9986216256	0.3031465075
H	-2.8731374725	-0.3306557253	-0.9174229392
O	2.2182967387	-0.3769472675	-0.5432063711
H	2.2103582125	0.9604455818	1.0437708452
H	1.3190292635	-0.5489871305	1.2480122614
C	0.2634641318	0.9331214121	0.1065620231
O	-1.4358194191	-0.7978609295	0.5678821010
H	-0.4029546805	-0.6585487668	-1.2614193703
C	1.5513136034	0.2471787132	0.5414412972
H	0.4865770248	1.6779814999	-0.6633426474
H	2.5524996092	0.3007606268	-1.1354760925

Energy of Optimized Geometry: -307.628648518311

OX4 3,4-epoxybutanol M06-2X STRUC9

C	-1.9203619455	0.1236284476	0.6609302920
C	-0.9222793911	0.3871779950	-0.3792719276
H	0.7243547282	-0.4738759952	-1.4222999393
H	-1.6959065833	-0.6141205079	1.4233375650
H	-2.6327308745	0.8916550023	0.9356843486
O	2.7124600929	-0.1932473973	0.3905602353
H	1.5574814172	1.4704497015	-0.0544248693
H	1.1642434157	0.5774139185	1.4112490021
C	0.4011990546	-0.3234099310	-0.3878064408
O	-2.0698415316	-0.3964965258	-0.6500083987
H	-0.9413344607	1.3652054446	-0.8522266425
C	1.4605141097	0.4643253464	0.3684292310
H	0.2831829262	-1.3089543661	0.0646270894
H	3.0216302237	-0.3080496823	-0.5112526978

Energy of Optimized Geometry: -307.627322440187

OX4 3,4-epoxybutanol M06-2X STRUC10

C	-2.2553633233	0.2345036885	0.2853482914
C	-0.9604066078	0.4527650049	-0.3611195033
H	0.1896855527	-0.0053109315	1.3732504537
H	-2.2886725645	0.1170979572	1.3623708110
H	-3.1604122771	0.6029647344	-0.1818151276
O	2.6713260198	-0.1620351709	0.3817634994
H	1.2119691221	-1.1986994173	-0.5357461590
H	1.5912239170	0.3338709195	-1.3196073555
C	0.3217387768	0.5053689910	0.4182714398
O	-1.6016207540	-0.8128929918	-0.4122189619
H	-0.9593700439	0.9743282452	-1.3145672782
C	1.4591791459	-0.1549804107	-0.3478608410
H	0.5733650993	1.5496050240	0.6274908139
H	2.9497494108	0.7437535084	0.5358020344

Energy of Optimized Geometry: -307.627929776468

OX4 3,4-epoxybutanol M06-2X STRUC12

C	-1.6798700862	-0.2367291450	-0.7952054093
C	-0.6995393610	-0.2687440191	0.2956957396
H	0.6587227493	0.8982934845	1.4570509583
H	-1.6378048093	0.5778152682	-1.5101945041
H	-2.1325740704	-1.1553120723	-1.1479732745
O	2.2361912754	-0.7450787487	0.0117391524
H	1.3262218640	0.3770827999	-1.4765658497
H	2.3393539322	1.2364083301	-0.3178303129
C	0.3670659380	0.7837545433	0.4116436771
O	-2.0357214363	0.1429781423	0.5233413077
H	-0.4696055699	-1.2354713172	0.7342254081
C	1.5973345005	0.4460895350	-0.4178878777
H	-0.0507611529	1.7345157452	0.0736000039
H	1.7262864016	-1.5025190612	-0.2825029372

Energy of Optimized Geometry: -307.627305762027

OX4 3,4-epoxybutanol M06-2X STRUC13

C	-1.8990846254	0.2463337722	0.6753791546
C	-0.9216839993	0.3593931162	-0.4107905932
H	0.7130484110	-0.6243342899	-1.3767683445
H	-1.6714965310	-0.3981771609	1.5174956289
H	-2.5896156303	1.0574258371	0.8706044727
O	2.7377086723	-0.1614536829	0.2716722593
H	1.5884769677	1.4107277034	-0.2089660009
H	1.1735283912	0.6859319962	1.3411596356
C	0.3888310734	-0.3734250268	-0.3652251649
O	-2.0905385501	-0.4262184249	-0.5580815915
H	-0.9332543808	1.2728811773	-0.9987209188
C	1.4678239860	0.4598131759	0.3106149529
H	0.2418903726	-1.3117836763	0.1764594343
H	2.6858979732	-1.0067839537	0.7240838038

Energy of Optimized Geometry: -307.627355123406

OX4 3,4-epoxybutanol M06-2X STRUC14

C	-1.3513165322	-0.6732939427	0.4875572501
C	-0.9855442799	0.6735099778	0.0436647163
H	0.7591573228	1.7648528095	0.6018266633
H	-0.5685960791	-1.3999586473	0.6614841604
H	-2.2738653658	-0.8177863703	1.0367435191
O	1.7367196318	-0.6794810394	0.8464135275
H	1.0055868585	-0.7940135803	-1.0280464024
H	2.3396182328	0.3422728905	-0.8520793578
C	0.4345566117	1.1104036113	-0.2122731270
O	-1.5201128724	-0.2727269370	-0.8664199367
H	-1.6748507889	1.4779269633	0.2817641320
C	1.4281277868	-0.0289450336	-0.3768391594
H	0.4391834453	1.7173705303	-1.1216717931
H	2.1819972391	-0.0577473162	1.4267854325

Energy of Optimized Geometry: -307.628776326820

OX4 3,4-epoxybutanol M06-2X STRUC15

C	-1.8527828323	-0.2774358734	0.4638800539
C	-1.0649139601	0.6400248937	-0.3661335751
H	0.6334097027	1.7779420165	0.2379723679
H	-1.3606207161	-0.8799656036	1.2177766715
H	-2.9014398539	-0.0704645852	0.6393730742
O	2.5354208415	-0.1515487824	0.4903357824
H	0.8627451195	-0.2408124581	1.5904491541
H	0.8147040619	-1.2666173920	0.1538394309
C	0.4245753459	0.8175287924	-0.2363236415
O	-1.5188760272	-0.6044412530	-0.8752137141
H	-1.5914626641	1.4905313069	-0.7877781689
C	1.1269590299	-0.2897940421	0.5347116653
H	0.8435521240	0.8669587868	-1.2464938324
H	2.8324701833	-0.2803942105	-0.4135522818

Energy of Optimized Geometry: -307.627157286214

OX4 3,4-epoxybutanol M06-2X STRUC16

C	-2.1575230430	0.1607968839	-0.2407147198
C	-0.7205411703	-0.0240447886	-0.4439008659
H	-0.1592883027	1.4657264058	0.9640581059
H	-2.5014740945	1.0230112991	0.3196433200
H	-2.8618628988	-0.2543732394	-0.9511584865
O	2.1598896094	-0.3452760259	-0.5620553083
H	2.2106491410	0.9177218737	1.0835587954
H	1.2597453274	-0.5659589633	1.2982541458
C	0.2866129688	0.9366510320	0.1201899148
O	-1.4316410548	-0.7948160263	0.5165909686
H	-0.3950746463	-0.5942020675	-1.3067131744
C	1.5371379325	0.2153621736	0.5846563140
H	0.5615182526	1.6709411360	-0.6397919913
H	2.8814821679	-0.9138858844	-0.2871814543

Energy of Optimized Geometry: -307.629664283128

OX4 3,4-epoxybutanol M06-2X STRUC17

C	-1.7165764205	-0.1159869602	-0.8051847173
C	-0.6722295298	-0.3491266005	0.1955399307
H	0.6404399533	0.6368675038	1.5590154737
H	-1.7540162221	0.8426363411	-1.3116594441
H	-2.1501533572	-0.9517374836	-1.3404761261
O	2.1775872993	-0.6782790631	-0.0777543419
H	1.2971007747	0.6414794466	-1.4153584464
H	2.2768312042	1.3942174150	-0.1426525391
C	0.3467763687	0.7099482670	0.5101502552
O	-2.0079273287	-0.0714038289	0.5823599625
H	-0.3620644858	-1.3723935944	0.3729394441
C	1.5810034401	0.5790080327	-0.3591875238
H	-0.1072975186	1.6906377351	0.3575269247
H	2.9558920823	-0.7930501917	-0.6263440166

Energy of Optimized Geometry: -307.628575254741

OX4 3,4-epoxybutanol M06-2X STRUC18

C	-1.4028552119	-0.8927325454	0.2672144050
C	-0.7879717606	0.4311018295	0.2091314195
H	0.3461643425	1.7214893842	-1.0534339271
H	-0.9776039497	-1.6851807984	-0.3366864366
H	-1.9357441066	-1.2019432919	1.1578282975
O	1.8240505082	-0.8182963603	0.4830154661
H	2.5456160891	0.8322405644	-0.5477222282
H	1.7152022110	1.1944707976	0.9737420034
C	0.4058912721	0.7037560234	-0.6617732785
O	-2.0090245570	0.1668695375	-0.4599481918
H	-0.8929052946	1.0748950834	1.0788415084
C	1.7094427857	0.5412723458	0.0938571925
H	0.3886508136	0.0187249611	-1.5109819093
H	2.6451734838	-0.9438619440	0.9626297256

Energy of Optimized Geometry: -307.628550249918

OX4 3,4-epoxybutanol M06-2X STRUC19

C	-2.2430135380	0.2496934302	0.3121629936
C	-0.9526002373	0.4687556131	-0.3422095657
H	0.2074887509	-0.0569845182	1.3652189055
H	-2.2655489109	0.0968927542	1.3849854400
H	-3.1472581560	0.6483496172	-0.1311680146
O	2.6353494540	-0.0977466658	0.4067737547
H	1.1848532248	-1.2109881176	-0.5837037059
H	1.5816572988	0.3496113946	-1.3246144310
C	0.3370895747	0.4797176935	0.4243481577
O	-1.6157892404	-0.7849036383	-0.4281937959
H	-0.9553757477	1.0216498892	-1.2777983697
C	1.4518870770	-0.1722231959	-0.3697758657
H	0.6201706330	1.5074969115	0.6604626762
H	3.3414396901	-0.5634610620	-0.0454544473

Energy of Optimized Geometry: -307.628577166412

OX4 3,4-epoxybutanol M06-2X STRUC20

C	-1.8427261740	-0.2095453722	0.5476854165
C	-1.0641218358	0.6486854510	-0.3522392861
H	0.6650816559	1.7770756788	0.1445499451
H	-1.3386319968	-0.7889124123	1.3116106662
H	-2.8799534431	0.0301500261	0.7480819429
O	2.5446048110	-0.2531043435	0.3200422296
H	0.8918425147	-0.2216952345	1.5655087850
H	0.8188703997	-1.2713927266	0.1465507408
C	0.4331405350	0.7981792631	-0.2848918000
O	-1.5624230692	-0.6060050316	-0.7848052136
H	-1.5862751280	1.4907851799	-0.7958480398
C	1.1414325854	-0.2935848745	0.5024145895
H	0.8259220799	0.7945690140	-1.3042935926
H	2.8783757922	0.5872250718	0.6428151957

Energy of Optimized Geometry: -307.626647522180

OX4 3,4-epoxybutanol M06-2X STRUC21

C	-1.3581887652	-0.6837849042	0.4764589381
C	-0.9802166482	0.6704215490	0.0714247195
H	0.7861660475	1.6995730659	0.6787103336
H	-0.5802552374	-1.4206604902	0.6274466717
H	-2.2793104223	-0.8376102981	1.0255081745
O	1.7321407056	-0.6163013921	0.8568002132
H	0.9841191445	-0.7619558942	-1.0811913257
H	2.3298519944	0.3702900975	-0.8640375076
C	0.4425429436	1.1056993075	-0.1703547595
O	-1.5281685085	-0.2439336406	-0.8649945170
H	-1.6611021221	1.4737250120	0.3359438618
C	1.4232311714	-0.0260539204	-0.4002054636
H	0.4465319311	1.7588322072	-1.0462305226
H	2.3643517775	-1.3256769568	0.7239058422

Energy of Optimized Geometry: -307.628979595723

OX4 3,4-epoxybutanol M06-2X STRUC22

C	-1.9067256051	0.2356884437	0.6625454448
C	-0.9215032115	0.3363592130	-0.4179616951
H	0.7291725952	-0.6625554544	-1.3393045376
H	-1.6731001484	-0.3797261913	1.5243579836
H	-2.6156702806	1.0376699708	0.8276999462
O	2.6808595363	-0.2114387963	0.2833258989
H	1.5574145447	1.4409531864	-0.2761527648
H	1.1657998168	0.7664686243	1.3136256948
C	0.4004445779	-0.3720198696	-0.3399033529
O	-2.0733083804	-0.4758400770	-0.5526077801
H	-0.9472461999	1.2315867301	-1.0333311326
C	1.4608922700	0.5082473035	0.2908643099
H	0.2848264159	-1.2846917117	0.2462557936
H	3.3691425497	0.3245991449	0.6822305615

Energy of Optimized Geometry: -307.627416123839

OX4 3,4-epoxybutanol M06-2X STRUC23

C	-1.3640419864	-0.7114459180	0.4379807263
C	-0.9437873408	0.6655759005	0.1362182270
H	0.8617957941	1.7216477187	0.5632449619
H	-0.6456228952	-1.5239472334	0.4122625860
H	-2.2328454235	-0.8771087074	1.0639042725
O	1.8661277380	-0.6417644293	0.8129945056
H	0.9600148307	-0.8561481350	-1.0473678397
H	2.3226506858	0.2519001852	-0.9241925986
C	0.4654886759	1.0715619306	-0.2190216486
O	-1.6288929154	-0.1240026216	-0.8213442026
H	-1.5596837355	1.4556519137	0.5541798948
C	1.4235191219	-0.0906081251	-0.4159767288
H	0.4114282715	1.6632056669	-1.1359540941
H	1.1040098813	-0.8158050756	1.3703668940

Energy of Optimized Geometry: -307.627532654926

OX4 3,4-epoxybutanol M06-2X STRUC24

C	-1.8549977798	-0.2479775774	0.4907403015
C	-1.0547195371	0.6458097383	-0.3527109669
H	0.6599822595	1.7597797193	0.2391951837
H	-1.3698518149	-0.8534412257	1.2469931051
H	-2.8983896679	-0.0197190225	0.6718488742
O	2.5241027736	-0.1267978702	0.3600018161
H	0.8602811470	-0.2659144309	1.5854609278
H	0.8094063658	-1.2791482321	0.1275604461
C	0.4373303659	0.8028698311	-0.2360785492
O	-1.5367772410	-0.5940677153	-0.8472723690
H	-1.5683124558	1.5019697128	-0.7785705479
C	1.1274454145	-0.3107152925	0.5249828855
H	0.8614170515	0.8411655712	-1.2419815539
H	2.9898828931	-0.8409512932	0.7994047637

Energy of Optimized Geometry: -307.627218655403

OX4 3,4-epoxybutanol M06-2X STRUC25

C	-2.1400279256	0.1745862840	0.1335541435
C	-0.7848962679	0.2871879579	-0.4105587917
H	0.2307952637	0.6342462408	1.4194037465
H	-2.3334111769	0.5411574531	1.1358857908
H	-2.9918902624	0.1525345473	-0.5354109890
O	1.8713748225	-0.8213708075	-0.0087760753
H	1.7848777457	0.8775066948	-1.1942021738
H	2.4611890037	1.1207450332	0.4243977783
C	0.3221785458	0.9365959256	0.3746111506
O	-1.2823229927	-0.9501824947	0.0616682640
H	-0.6799915261	0.3294170893	-1.4919618642
C	1.7001716063	0.5765565284	-0.1433494144
H	0.2076130040	2.0222537426	0.3248862764
H	2.7189324288	-1.0771381434	-0.3777829666

Energy of Optimized Geometry: -307.625662035200

6.4 MN15

OX4 3,4-epoxybutanol MN15 STRUC1

C	-2.0413916563	-0.0015010038	0.3107660668
C	-0.8546768142	0.4730626624	-0.4006361404
H	0.2524150774	0.8375041952	1.3853845723
H	-2.0741294506	0.0954091963	1.3900636435
H	-2.9996694147	-0.0303081258	-0.1928003119
O	1.8052134380	-0.8596950142	0.1524879491
H	1.6675903598	0.6082689156	-1.3043043218
H	2.4796077116	1.0395221404	0.1992518743
C	0.3260208226	1.0606373819	0.3185196734
O	-1.0798459359	-0.9182856700	-0.1826921623
H	-0.9698801903	0.7661933307	-1.4403370073
C	1.6342669203	0.4895958355	-0.2132382633
H	0.3149657313	2.1457149809	0.1959324514
H	0.9591723400	-1.3176261495	0.0626717384

Energy of Optimized Geometry: -307.407357172723

OX4 3,4-epoxybutanol MN15 STRUC2

C	-1.7130660011	-0.0894052751	0.5872657835
C	-0.9564649120	0.5236708459	-0.5058960049
H	0.2479277423	2.1667156495	0.1014523877
H	-1.2968943621	-0.1025887530	1.5875556700
H	-2.7937720595	-0.1328606979	0.5290058772
O	1.7716430301	-0.8216609532	-0.0228394665
H	2.2098918127	0.9559269137	0.8139947777
H	0.8394068504	0.1457414423	1.5533774462
C	0.3946645664	1.1637286497	-0.3072758008
O	-1.0299783432	-0.8914715003	-0.3629218968
H	-1.5336420014	0.8980494860	-1.3452039721
C	1.3242800004	0.3616233788	0.5938428195
H	0.8855606075	1.2775543719	-1.2754657052
H	0.9892333911	-1.3527142264	-0.2233641186

Energy of Optimized Geometry: -307.408011490519

OX4 3,4-epoxybutanol MN15 STRUC3

C	-1.7190703512	-0.0745867896	-0.8471637315
C	-0.6844811021	-0.3559267936	0.1494431710
H	0.5674494085	0.6136319363	1.5847116563
H	-1.7516178069	0.9100186329	-1.3026110509
H	-2.1396652214	-0.8807834460	-1.4358601996
O	2.2448883565	-0.6634794036	-0.1014973996
H	1.3472729786	0.6125225641	-1.3671290543
H	2.2628243426	1.4117083279	-0.0911123530
C	0.3209188099	0.6959840886	0.5216379720
O	-2.0238114200	-0.1085727536	0.5354979765
H	-0.3634361907	-1.3848194867	0.2698302198
C	1.5913274627	0.5752319650	-0.3049274374
H	-0.1311144556	1.6791358341	0.3715130720
H	2.5495221499	-0.7184866536	0.8100575815

Energy of Optimized Geometry: -307.404020762335

OX4 3,4-epoxybutanol MN15 STRUC4

C	-1.3885356543	-0.9202154907	0.2319313273
C	-0.7834289503	0.4077436157	0.2877030223
H	0.2954825032	1.8289685035	-0.8850546925
H	-0.9908262034	-1.6417517570	-0.4741311511
H	-1.8719616430	-1.3348947220	1.1079023448
O	1.9098728110	-0.8340809208	0.3896220079
H	2.5191103196	0.9066914016	-0.5607229684
H	1.7572221311	1.0666169245	1.0170639138
C	0.3680390593	0.7760015108	-0.6028504066
O	-2.0315371366	0.2014150372	-0.3471968433
H	-0.8443879309	0.9519476274	1.2266719737
C	1.7091898720	0.5284012640	0.0696696692
H	0.2953938270	0.1903547069	-1.5246975606
H	1.8988695666	-1.3564515517	-0.4190554924

Energy of Optimized Geometry: -307.403991892736

OX4 3,4-epoxybutanol MN15 STRUC5

C	-1.3888866968	-0.9039595759	0.2519174603
C	-0.7790153169	0.4327207298	0.2447355215
H	0.3268159387	1.7876636911	-0.9746463268
H	-0.9846795496	-1.6593554657	-0.4141402160
H	-1.8898786919	-1.2704231203	1.1403825183
O	1.9158716162	-0.8380307716	0.4007122868
H	2.5399256101	0.8071384401	-0.5718609786
H	1.7601634133	1.1492855755	0.9687419130
C	0.3887284470	0.7478869029	-0.6462583136
O	-2.0172975611	0.1884362161	-0.3883714879
H	-0.8551319066	1.0264558616	1.1531202689
C	1.7102533482	0.5209470732	0.0712801493
H	0.3357661665	0.1110475449	-1.5313846376
H	1.1973436041	-1.1391041053	0.9665345524

Energy of Optimized Geometry: -307.404627504468

OX4 3,4-epoxybutanol MN15 STRUC6

C	-2.1455527852	0.2375124555	-0.0717268407
C	-0.7394974050	0.1405349368	-0.4621486511
H	-0.0781528627	1.4684438376	1.0635867109
H	-2.4277722897	0.9690556158	0.6775870747
H	-2.9240488922	-0.0503558206	-0.7681357338
O	2.1100810541	-0.4969433710	-0.5694185938
H	2.3115301977	0.7849114677	0.9779624618
H	1.2418466292	-0.5777003517	1.3117395585
C	0.3255683902	0.9913236726	0.1674864014
O	-1.3322076242	-0.8221528780	0.3989321540
H	-0.5200816657	-0.2373484410	-1.4565342879
C	1.5359852804	0.1505270927	0.5513451156
H	0.6297289309	1.7730965893	-0.5308222761
H	1.6081318470	-1.2922551601	-0.7718796961

Energy of Optimized Geometry: -307.404943687038

OX4 3,4-epoxybutanol MN15 STRUC7

C	-2.2495927369	0.2786144009	0.2465826559
C	-0.9451339901	0.4647901720	-0.3881789722
H	0.1478210346	0.0051581309	1.3668134556
H	-2.2953624678	0.1908911861	1.3267156080
H	-3.1452328809	0.6514339233	-0.2356491296
O	2.6748336949	-0.1337057246	0.3931711535
H	1.1734837550	-1.2034876866	-0.5672701644
H	1.6511611653	0.3382525937	-1.2714694354
C	0.3229081610	0.5102499348	0.4124030000
O	-1.6039489951	-0.7912956701	-0.4187296279
H	-0.9191129983	0.9682419791	-1.3510498877
C	1.4611023720	-0.1752258300	-0.3283880608
H	0.6016437273	1.5439759365	0.6257233231
H	2.5870374000	-0.6546881840	1.1974293577

Energy of Optimized Geometry: -307.404086479676

OX4 3,4-epoxybutanol MN15 STRUC8

C	-2.1688649364	0.1153060992	-0.2120708106
C	-0.7333105121	-0.0670091302	-0.4229521550
H	-0.1816167847	1.4767397226	0.9215759560
H	-2.5126781194	1.0093869758	0.2976579806
H	-2.8800063170	-0.3442265485	-0.8881411821
O	2.2081976875	-0.3875130532	-0.5413013879
H	2.2073622579	0.9731128252	1.0263882818
H	1.3070796892	-0.5266937119	1.2535727992
C	0.2628166233	0.9360615931	0.0826740118
O	-1.4235914821	-0.7844710009	0.5876120416
H	-0.4061307403	-0.6812320490	-1.2552867450
C	1.5465636619	0.2546555756	0.5329241338
H	0.4907035640	1.6655277492	-0.7008869604
H	2.5347413919	0.2748933878	-1.1585758067

Energy of Optimized Geometry: -307.404494474556

OX4 3,4-epoxybutanol MN15 STRUC9

C	-1.9085282920	0.1236971845	0.6650116736
C	-0.9223479572	0.3872500075	-0.3851100453
H	0.7293434553	-0.4784043745	-1.4236760825
H	-1.6699528048	-0.6094096033	1.4285387059
H	-2.6190226074	0.8902774320	0.9496575524
O	2.7069433321	-0.1894220851	0.3867468739
H	1.5467279940	1.4719383019	-0.0549620235
H	1.1550607365	0.5773702864	1.4083762020
C	0.3999808072	-0.3242209682	-0.3915799437
O	-2.0691929692	-0.3984362896	-0.6408930172
H	-0.9416961906	1.3647393941	-0.8597971064
C	1.4558612837	0.4640739267	0.3667657727
H	0.2760923486	-1.3080935394	0.0638856605
H	3.0114506885	-0.3276447908	-0.5158218388

Energy of Optimized Geometry: -307.403037504821

OX4 3,4-epoxybutanol MN15 STRUC10

C	-2.2484266960	0.2437733323	0.2854827385
C	-0.9557874363	0.4658560682	-0.3622225468
H	0.1783023026	-0.0137596918	1.3727795625
H	-2.2767447154	0.1213100622	1.3626808012
H	-3.1555822261	0.6196585633	-0.1723364942
O	2.6632111876	-0.1843493744	0.3835152821
H	1.1926506232	-1.2057213959	-0.5373235939
H	1.5857555471	0.3243051810	-1.3162482967
C	0.3230816785	0.5026432867	0.4222608438
O	-1.5988732998	-0.7960531388	-0.4221134031
H	-0.9501774375	0.9985658985	-1.3097590652
C	1.4534045515	-0.1662292271	-0.3446066487
H	0.5880710305	1.5419368265	0.6407911047
H	2.9456217646	0.7183436244	0.5611087242

Energy of Optimized Geometry: -307.403661557403

OX4 3,4-epoxybutanol MN15 STRUC12

C	-1.6586219575	-0.2411161706	-0.7978654700
C	-0.6956868401	-0.2746289489	0.3069159591
H	0.6620301376	0.9018172467	1.4678636809
H	-1.6008534228	0.5754155854	-1.5106981003
H	-2.1025998633	-1.1584549218	-1.1655568182
O	2.2276976617	-0.7352394911	0.0013547842
H	1.3007115176	0.3905746593	-1.4729575805
H	2.3261919994	1.2481553306	-0.3250935955
C	0.3628988184	0.7850102958	0.4250770728
O	-2.0344728349	0.1318398998	0.5142581162
H	-0.4629590277	-1.2408307655	0.7461017027
C	1.5860264319	0.4552333192	-0.4167932632
H	-0.0682690941	1.7312447998	0.0895812299
H	1.7059619812	-1.4980120118	-0.2653736026

Energy of Optimized Geometry: -307.403173016746

OX4 3,4-epoxybutanol MN15 STRUC13

C	-1.8821323413	0.2614150693	0.6761797615
C	-0.9209974473	0.3540119905	-0.4249968201
H	0.7198988834	-0.6473356973	-1.3724610615
H	-1.6384621824	-0.3664525987	1.5273797819
H	-2.5678225456	1.0768757920	0.8718744632
O	2.7311018778	-0.1564440496	0.2697535829
H	1.5713212171	1.4117080810	-0.2072497589
H	1.1602627879	0.6797579179	1.3394033207
C	0.3870295889	-0.3811441503	-0.3678733571
O	-2.0915210884	-0.4305894040	-0.5408329908
H	-0.9327753540	1.2576224399	-1.0285190242
C	1.4603734451	0.4570869335	0.3085405599
H	0.2311391472	-1.3115417850	0.1866403684
H	2.6855015217	-1.0160095192	0.7008320291

Energy of Optimized Geometry: -307.403078748085

OX4 3,4-epoxybutanol MN15 STRUC14

C	-1.3517612524	-0.6833861438	0.4592860666
C	-0.9922860231	0.6657538142	0.0206555382
H	0.7419377479	1.7668847447	0.6043849724
H	-0.5638929795	-1.4005538909	0.6513187078
H	-2.2801783234	-0.8376245969	0.9965598663
O	1.7328709734	-0.6464501656	0.8907122949
H	1.0000370949	-0.8126605431	-0.9815199709
H	2.3346877068	0.3259546936	-0.8365023620
C	0.4296639913	1.1080440631	-0.2116364592
O	-1.5007631474	-0.2864508322	-0.8951364666
H	-1.6902759130	1.4663594866	0.2474855460
C	1.4244903054	-0.0329352389	-0.3486701546
H	0.4495021579	1.7109958586	-1.1233820656
H	2.1575346442	-0.0047313880	1.4687625162

Energy of Optimized Geometry: -307.404961874954

OX4 3,4-epoxybutanol MN15 STRUC15

C	-1.8430750031	-0.2930348578	0.4630792492
C	-1.0699178821	0.6312573160	-0.3716725791
H	0.6145452615	1.7909700272	0.2383417653
H	-1.3406204697	-0.8785130226	1.2245625029
H	-2.8949044771	-0.1021914960	0.6397745511
O	2.5273606725	-0.1379729466	0.4829173329
H	0.8624036532	-0.2179337669	1.5981527403
H	0.8011208004	-1.2514106151	0.1692599225
C	0.4159918578	0.8274146767	-0.2337939141
O	-1.5022167062	-0.6222074801	-0.8708257165
H	-1.6059081678	1.4734212378	-0.7986675761
C	1.1221070594	-0.2735161935	0.5415452105
H	0.8427300028	0.8770654445	-1.2410259530
H	2.8214816576	-0.2491240044	-0.4267159310

Energy of Optimized Geometry: -307.403358731177

OX4 3,4-epoxybutanol MN15 STRUC16

C	-2.1515679812	0.1720462576	-0.2339337335
C	-0.7186431134	-0.0217144076	-0.4510401837
H	-0.1592052926	1.4783559197	0.9414211262
H	-2.4828799131	1.0443171155	0.3198317523
H	-2.8678126635	-0.2409813912	-0.9342044434
O	2.1490885156	-0.3682373471	-0.5531490552
H	2.2087460817	0.9068967482	1.0833263056
H	1.2353256478	-0.5605805691	1.3031965128
C	0.2912225639	0.9398265407	0.1053548309
O	-1.4236251039	-0.7795097286	0.5211164889
H	-0.3989984868	-0.5963050961	-1.3132856303
C	1.5302596166	0.2093128560	0.5836124744
H	0.5776193699	1.6655837763	-0.6584328763
H	2.8606099732	-0.9542890269	-0.2810212619

Energy of Optimized Geometry: -307.405503221901

OX4 3,4-epoxybutanol MN15 STRUC17

C	-1.6969338761	-0.1186281957	-0.8125147306
C	-0.6719697662	-0.3504214697	0.2068665403
H	0.6415885370	0.6522475935	1.5628119984
H	-1.7118616091	0.8353606586	-1.3305266797
H	-2.1278439732	-0.9539841588	-1.3511758664
O	2.1678483184	-0.6822707740	-0.0690743344
H	1.2851198968	0.6291002797	-1.4131360710
H	2.2734924560	1.3893983790	-0.1528327232
C	0.3452404749	0.7118020010	0.5141250110
O	-2.0101158176	-0.0585170867	0.5669077353
H	-0.3664155268	-1.3723636178	0.3996822426
C	1.5759854034	0.5722489353	-0.3578186040
H	-0.1146096993	1.6883817640	0.3496936160
H	2.9476231074	-0.8153804817	-0.6152582226

Energy of Optimized Geometry: -307.404378160990

OX4 3,4-epoxybutanol MN15 STRUC18

C	-1.3742465266	-0.8954303939	0.2811756865
C	-0.7897636046	0.4407668227	0.2151576202
H	0.3396558360	1.7369105432	-1.0527618339
H	-0.9269584044	-1.6794139506	-0.3189206065
H	-1.8937877712	-1.2168791015	1.1757307193
O	1.8096869598	-0.8166816265	0.4622007460
H	2.5381796454	0.8375989714	-0.5577682256
H	1.7142101283	1.1927734618	0.9679870618
C	0.3967906847	0.7185459877	-0.6631902925
O	-2.0055412102	0.1458306420	-0.4459230633
H	-0.8983931758	1.0882824647	1.0820028546
C	1.7030333701	0.5438719803	0.0843385140
H	0.3690059280	0.0331876421	-1.5124857357
H	2.6306895108	-0.9633613025	0.9404104379

Energy of Optimized Geometry: -307.404462845145

OX4 3,4-epoxybutanol MN15 STRUC19

C	-2.2371801239	0.2424124770	0.3180032299
C	-0.9532606098	0.4732366527	-0.3431292757
H	0.2005497965	-0.0510740070	1.3646379872
H	-2.2495097640	0.0920249269	1.3919489282
H	-3.1486013971	0.6366313472	-0.1153293654
O	2.6298928532	-0.1021969378	0.4003891233
H	1.1689358150	-1.2078991897	-0.5831473838
H	1.5707583294	0.3484934561	-1.3273048557
C	0.3352327866	0.4859667716	0.4242335829
O	-1.6067805732	-0.7833411990	-0.4268616094
H	-0.9596214811	1.0303337745	-1.2765013364
C	1.4457666643	-0.1712135687	-0.3698999082
H	0.6226880568	1.5121649540	0.6615487687
H	3.3392422013	-0.5753664614	-0.0434854102

Energy of Optimized Geometry: -307.404275572771

OX4 3,4-epoxybutanol MN15 STRUC20

C	-1.8440453344	-0.2241401239	0.5086598318
C	-1.0591811323	0.6507709563	-0.3679471871
H	0.6547244409	1.7830784899	0.1822138481
H	-1.3461238100	-0.8011745755	1.2793844231
H	-2.8869253819	0.0027665119	0.6961093834
O	2.5287057540	-0.2555666643	0.3614431091
H	0.8590057294	-0.2263200674	1.5847688861
H	0.7975865732	-1.2635067823	0.1577549352
C	0.4345318807	0.8088938473	-0.2642250167
O	-1.5350437635	-0.6036086515	-0.8194536354
H	-1.5784236189	1.4945930042	-0.8122571972
C	1.1256580712	-0.2914843535	0.5247250235
H	0.8532686685	0.8178950848	-1.2732554360
H	2.8680562248	0.5878776496	0.6770702717

Energy of Optimized Geometry: -307.402841565750

OX4 3,4-epoxybutanol MN15 STRUC21

C	-1.3624131107	-0.6758646656	0.4636322895
C	-0.9778147329	0.6765428340	0.0624894286
H	0.7991575807	1.6922660310	0.6706555431
H	-0.5867326171	-1.4150475776	0.6190547519
H	-2.2831647791	-0.8284234379	1.0143745563
O	1.7351489785	-0.6067301696	0.8770827666
H	0.9541056284	-0.7965777180	-1.0447828465
H	2.3173674568	0.3221233965	-0.8805058070
C	0.4493118582	1.0974869216	-0.1752503324
O	-1.5264215780	-0.2340619499	-0.8746840074
H	-1.6527578737	1.4847256836	0.3279717016
C	1.4146485082	-0.0499161628	-0.3891117338
H	0.4647523434	1.7450889759	-1.0548430404
H	2.3454522027	-1.3414946632	0.7653263640

Energy of Optimized Geometry: -307.405141392358

OX4 3,4-epoxybutanol MN15 STRUC22

C	-1.8929112725	0.2338913991	0.6648422862
C	-0.9216219203	0.3345875699	-0.4270123347
H	0.7356489657	-0.6636283958	-1.3456912929
H	-1.6431504468	-0.3775722672	1.5259102328
H	-2.5996817779	1.0356460063	0.8421699540
O	2.6752480736	-0.2041942117	0.2878533419
H	1.5447580485	1.4433740969	-0.2712632257
H	1.1476056490	0.7606295586	1.3119776137
C	0.3995491451	-0.3737795435	-0.3488406071
O	-2.0726606171	-0.4774523411	-0.5453907070
H	-0.9489432395	1.2298071425	-1.0428413291
C	1.4537388517	0.5064893778	0.2907921882
H	0.2777104069	-1.2865878434	0.2365484159
H	3.3679340436	0.3276221977	0.6898665969

Energy of Optimized Geometry: -307.403096180697

OX4 3,4-epoxybutanol MN15 STRUC23

C	-1.3629407495	-0.6866770694	0.4524079951
C	-0.9421919432	0.6777528593	0.1034982817
H	0.8719125616	1.7290996509	0.5224634208
H	-0.6395991912	-1.4960701923	0.4724447518
H	-2.2388655933	-0.8325454010	1.0738257889
O	1.8462641500	-0.6227859586	0.8468143233
H	0.9378325944	-0.8943900494	-1.0067195277
H	2.3177666674	0.1946098458	-0.9259679587
C	0.4727884370	1.0648754045	-0.2468684545
O	-1.6093962546	-0.1504206683	-0.8307708353
H	-1.5621520998	1.4849369961	0.4816469537
C	1.4155804360	-0.1147947798	-0.4024536890
H	0.4337753853	1.6354191333	-1.1776808587
H	1.0823996886	-0.7558979056	1.4176523538

Energy of Optimized Geometry: -307.403884186209

OX4 3,4-epoxybutanol MN15 STRUC24

C	-1.8560538406	-0.2718816032	0.4370876120
C	-1.0522704794	0.6324373023	-0.3903376119
H	0.6250216746	1.7786060584	0.2539826148
H	-1.3776021756	-0.8565591009	1.2148352891
H	-2.9087419311	-0.0657746100	0.5909337471
O	2.5044056719	-0.1039378913	0.4280654734
H	0.8126501606	-0.2339159448	1.6161138387
H	0.7945754695	-1.2580181832	0.1664418328
C	0.4313770221	0.8167798747	-0.2240548138
O	-1.4922714157	-0.6202064330	-0.8855975759
H	-1.5681776387	1.4766914143	-0.8372374863
C	1.1075380928	-0.2866693980	0.5626578824
H	0.8915224972	0.8569280033	-1.2137830514
H	2.9727676124	-0.8102712828	0.8817467340

Energy of Optimized Geometry: -307.403407544791

OX4 3,4-epoxybutanol MN15 STRUC25

C	-2.1342491417	0.1714355961	0.1378637080
C	-0.7824871526	0.2866155572	-0.4113020686
H	0.2251473503	0.6319456909	1.4196128632
H	-2.3231806907	0.5428408071	1.1399704143
H	-2.9908275345	0.1509091371	-0.5257572953
O	1.8623706571	-0.8200054544	-0.0149047495
H	1.7793013730	0.8841446642	-1.1921026675
H	2.4611173310	1.1184871341	0.4248074218
C	0.3209017636	0.9382035578	0.3760397361
O	-1.2758728100	-0.9493740124	0.0627858363
H	-0.6762700008	0.3327153902	-1.4928713300
C	1.6976226502	0.5763464506	-0.1422813657
H	0.2068258963	2.0240357640	0.3295845291
H	2.7045948140	-1.0911665385	-0.3906886832

Energy of Optimized Geometry: -307.401433629879

6.5 ωB97X-D

OX4 3,4-epoxybutanol ωB97X-D STRUC1

C	-2.0494376248	-0.0448042120	0.2823013247
C	-0.8706039610	0.4644295022	-0.4099419083
H	0.2211190583	0.8867812997	1.3840837888
H	-2.0939705273	0.0206164143	1.3641319784
H	-3.0050938311	-0.0769923830	-0.2282418095
O	1.8399334530	-0.8238272514	0.2137215338
H	1.6824150985	0.6036680955	-1.2800989885
H	2.4547772931	1.0908521721	0.2276476796
C	0.3013593700	1.0716844460	0.3112806457
O	-1.0711697861	-0.9397194292	-0.2275286788
H	-0.9912783898	0.7747152764	-1.4445896557
C	1.6300133330	0.5115019467	-0.1870244554
H	0.2803787695	2.1529012901	0.1559501881
H	1.0227262702	-1.3109121628	0.0805436213

Energy of Optimized Geometry: -307.660654428281

OX4 3,4-epoxybutanol ωB97X-D STRUC2

C	-1.6843007934	-0.1537865849	0.5593158523
C	-0.9563130984	0.5583176452	-0.4875221871
H	0.2843410434	2.1821091961	0.0678101039
H	-1.2356116034	-0.2940571770	1.5358044849
H	-2.7680587443	-0.1685368902	0.5391974725
O	1.7326727795	-0.8562111247	0.1012345214
H	2.2688871010	0.9718079080	0.7441774569
H	0.9044942475	0.2930877894	1.6140270418
C	0.4136256568	1.1637184213	-0.3084858950
O	-1.0575444138	-0.8666052412	-0.5012622096
H	-1.5586481143	1.0292399960	-1.2590830717
C	1.3515775294	0.3975866137	0.6173546572
H	0.8880332208	1.2435332219	-1.2888809793
H	0.9251510577	-1.3217117084	-0.1368051134

Energy of Optimized Geometry: -307.661065754099

OX4 3,4-epoxybutanol ωB97X-D STRUC3

C	-1.7508603072	-0.0737017517	-0.8223248490
C	-0.6966025121	-0.3421660897	0.1513489821
H	0.5780566354	0.6154152960	1.5673829369
H	-1.8018286492	0.9034586073	-1.2926860242
H	-2.1854784657	-0.8896513596	-1.3888920958
O	2.2636790698	-0.6803340166	-0.1263242397
H	1.3641422507	0.5952132522	-1.3859644802
H	2.2773834460	1.3943007536	-0.1087334498
C	0.3332217025	0.6949155940	0.5036960616
O	-2.0285688023	-0.0868769182	0.5699031657
H	-0.3836400021	-1.3733772805	0.2788637092
C	1.6047293829	0.5577074198	-0.3230011721
H	-0.0924201354	1.6891289906	0.3508721778
H	2.5790832883	-0.7213944612	0.7769293784

Energy of Optimized Geometry: -307.658096489931

OX4 3,4-epoxybutanol ωB97X-D STRUC4

C	-1.4448896313	-0.9170464703	0.2159759147
C	-0.7873545384	0.3815576302	0.2811048422
H	0.2898492666	1.7973574808	-0.8866647189
H	-1.0929767746	-1.6511187004	-0.5015775512
H	-1.9378381678	-1.3193137257	1.0937765160
O	1.9666020981	-0.8242676990	0.4090704199
H	2.5170729643	0.9205969704	-0.5675591041
H	1.7600719279	1.0757091609	1.0128842747
C	0.3719821920	0.7445617071	-0.6039270475
O	-2.0514209290	0.2367094756	-0.3477014422
H	-0.8273748789	0.9146118770	1.2283858449
C	1.7195787148	0.5270709481	0.0708615728
H	0.3149358823	0.1641674716	-1.5290435808
H	1.9974876895	-1.3421734409	-0.3957080205

Energy of Optimized Geometry: -307.657982094265

OX4 3,4-epoxybutanol ωB97X-D STRUC5

C	-1.4355963828	-0.8954186174	0.2404227180
C	-0.7901209846	0.4195201921	0.2411339443
H	0.3339322173	1.7761700630	-0.9495926575
H	-1.0579099029	-1.6653333060	-0.4246479014
H	-1.9524164509	-1.2477008667	1.1267166603
O	1.9680966874	-0.8437606975	0.3973447616
H	2.5397719597	0.8289223134	-0.5503068396
H	1.7572934636	1.1293378017	0.9972713690
C	0.3940036218	0.7324194470	-0.6314235885
O	-2.0338031156	0.2160225302	-0.4040602909
H	-0.8642248985	1.0105544109	1.1521488111
C	1.7175043003	0.5140540249	0.0898989802
H	0.3567256373	0.1109849224	-1.5274405490
H	1.2895089718	-1.1553801524	0.9964594890

Energy of Optimized Geometry: -307.658486898138

OX4 3,4-epoxybutanol ωB97X-D STRUC6

C	-2.1533498170	0.3190480069	-0.1509251355
C	-0.7395842903	0.1342165059	-0.4588823291
H	-0.0479057076	1.4282766077	1.0879044930
H	-2.4408949203	1.0738669146	0.5737376770
H	-2.9065276165	0.0659724927	-0.8889908910
O	2.1794316422	-0.5152976332	-0.5307177156
H	2.3066520385	0.7319688103	1.0451151205
H	1.2499007411	-0.6519121562	1.3193018494
C	0.3457525671	0.9448573245	0.1909237474
O	-1.4340065092	-0.7829299161	0.3808669092
H	-0.4959756127	-0.2744159273	-1.4362018278
C	1.5516174336	0.0974961469	0.5820160137
H	0.6647174676	1.7277075090	-0.5005638276
H	1.6936070674	-1.3057180577	-0.7644498589

Energy of Optimized Geometry: -307.658795986158

OX4 3,4-epoxybutanol ωB97X-D STRUC7

C	-2.2571830274	0.2845651706	0.2618629152
C	-0.9522333630	0.4439027114	-0.3704085391
H	0.1718842202	-0.0326870774	1.3698202358
H	-2.3129018572	0.1864035941	1.3411810451
H	-3.1437061351	0.6822727286	-0.2196128338
O	2.7016063197	-0.0953502477	0.3650329298
H	1.2222807857	-1.2050239035	-0.5804606071
H	1.6452898949	0.3546549007	-1.2808729772
C	0.3256654580	0.4822283193	0.4178442641
O	-1.6390496130	-0.8008850376	-0.4114758637
H	-0.9227618110	0.9517296208	-1.3316822323
C	1.4770498043	-0.1684856428	-0.3387396230
H	0.5858517926	1.5192944943	0.6408581818
H	2.6398664377	-0.6421625690	1.1484009906

Energy of Optimized Geometry: -307.658286487890

OX4 3,4-epoxybutanol ωB97X-D STRUC8

C	-2.1749919978	0.1429442801	-0.2441137262
C	-0.7410276227	-0.0651316375	-0.4129478508
H	-0.1530817524	1.4640938306	0.9443662200
H	-2.5247945024	1.0372215741	0.2623820363
H	-2.8730093390	-0.3005769823	-0.9457454365
O	2.2403657166	-0.3986118140	-0.5282375183
H	2.2195537158	0.9765128366	1.0254727581
H	1.3443735804	-0.5348370140	1.2649144321
C	0.2722088529	0.9196784355	0.0982826342
O	-1.4734694653	-0.7784449939	0.5758979339
H	-0.4107763917	-0.6853833057	-1.2404266608
C	1.5663892479	0.2458045421	0.5379901089
H	0.4901078726	1.6531908428	-0.6845926815
H	2.5651002402	0.2668159755	-1.1352469665

Energy of Optimized Geometry: -307.658399594313

OX4 3,4-epoxybutanol ωB97X-D STRUC9

C	-1.9207777311	0.1294196887	0.6831138572
C	-0.9301052911	0.3793784859	-0.3608919671
H	0.6959930636	-0.4930498169	-1.4214632404
H	-1.6997718933	-0.6021571247	1.4538581660
H	-2.6268721632	0.9049027342	0.9584788026
O	2.7252860619	-0.1869520061	0.3518356890
H	1.5629270619	1.4688761607	-0.1025471817
H	1.1988833118	0.6023555124	1.3859767756
C	0.3957134330	-0.3289631575	-0.3820251113
O	-2.0833894231	-0.4020285129	-0.6225629264
H	-0.9487111178	1.3546474847	-0.8416995301
C	1.4738715385	0.4690908498	0.3395060312
H	0.2920702918	-1.3094406840	0.0845572329
H	3.0222637518	-0.3052351433	-0.5508967475

Energy of Optimized Geometry: -307.657402545047

OX4 3,4-epoxybutanol ωB97X-D STRUC10

C	-2.2573660083	0.2464109948	0.2866056305
C	-0.9622282136	0.4383917197	-0.3573871368
H	0.1968116424	-0.0324456139	1.3719850219
H	-2.2985469983	0.1291583324	1.3645935132
H	-3.1558247648	0.6369868448	-0.1784285471
O	2.6864668063	-0.1446399508	0.3770255918
H	1.2467715186	-1.1994700225	-0.5438453711
H	1.5962015181	0.3432106620	-1.3195610938
C	0.3226610305	0.4848351283	0.4197794577
O	-1.6278846330	-0.8159762731	-0.4118236790
H	-0.9518776631	0.9622084988	-1.3106463372
C	1.4734946331	-0.1523307212	-0.3488287392
H	0.5617719897	1.5303785952	0.6396661895
H	2.9528645295	0.7627476629	0.5264927401

Energy of Optimized Geometry: -307.657882716188

OX4 3,4-epoxybutanol ωB97X-D STRUC12

C	-1.7048403132	-0.2352074356	-0.8033473773
C	-0.7139051421	-0.2708505883	0.2708321755
H	0.6313591221	0.8987435841	1.4418403663
H	-1.6775553282	0.5809474710	-1.5187115650
H	-2.1579036905	-1.1544488840	-1.1582063285
O	2.2770012213	-0.7235065093	0.0516039737
H	1.3548249909	0.3383533880	-1.4733741183
H	2.3280462456	1.2491284358	-0.3225025143
C	0.3590519736	0.7755334932	0.3920031718
O	-2.0487042017	0.1371466130	0.5212714792
H	-0.4771849824	-1.2414745450	0.6996767664
C	1.6083753258	0.4364811499	-0.4117120293
H	-0.0427672952	1.7286500064	0.0408470263
H	1.7917095285	-1.4996469142	-0.2262815254

Energy of Optimized Geometry: -307.657252326152

OX4 3,4-epoxybutanol ωB97X-D STRUC13

C	-1.9068082865	0.2509924370	0.6811983199
C	-0.9256461019	0.3563379212	-0.3960732291
H	0.6947479871	-0.6372278716	-1.3689502537
H	-1.6920116238	-0.3917380155	1.5293606871
H	-2.5925394305	1.0686007747	0.8737036140
O	2.7471149215	-0.1628794401	0.2500241690
H	1.5963316217	1.4047679637	-0.2321930202
H	1.1996073032	0.6985666505	1.3305748934
C	0.3859312266	-0.3768432544	-0.3548865208
O	-2.0987958640	-0.4242259086	-0.5515517826
H	-0.9302141309	1.2696414964	-0.9861587267
C	1.4793163070	0.4590097516	0.2980817018
H	0.2485075094	-1.3116721546	0.1951823009
H	2.7026431690	-0.9928718423	0.7253721845

Energy of Optimized Geometry: -307.657424638202

OX4 3,4-epoxybutanol ωB97X-D STRUC14

C	-1.3742296854	-0.6924232105	0.4720131326
C	-1.0048261246	0.6511634820	0.0364768224
H	0.6978793309	1.7584749729	0.6708842896
H	-0.5958716982	-1.4184223894	0.6710470650
H	-2.3105931404	-0.8413562399	0.9989787517
O	1.7587574074	-0.6815254916	0.8320054794
H	1.0892797878	-0.7168870426	-1.0648078144
H	2.3645030463	0.4636923373	-0.7839384484
C	0.4136201182	1.1204344655	-0.1717496327
O	-1.5092742411	-0.2985608321	-0.8887217153
H	-1.7069993350	1.4515596907	0.2557502665
C	1.4561077581	0.0268705128	-0.3580845364
H	0.4244679566	1.7615118284	-1.0577601196
H	2.1423356076	-0.0733832754	1.4641157314

Energy of Optimized Geometry: -307.657877715971

OX4 3,4-epoxybutanol ωB97X-D STRUC15

C	-1.8552820719	-0.2207337487	0.5442878336
C	-1.0675514542	0.6458806407	-0.3306383186
H	0.6691346517	1.7532957166	0.1965496079
H	-1.3626856078	-0.8285083431	1.2951464336
H	-2.8882483184	0.0319152994	0.7574905781
O	2.5686452883	-0.1760756758	0.3928511984
H	0.9335678199	-0.2719630095	1.5440332859
H	0.8412501290	-1.2935625258	0.1088281859
C	0.4304490828	0.7924761357	-0.2639338302
O	-1.5876571541	-0.5900407832	-0.7998438165
H	-1.5787046990	1.5086069897	-0.7495360253
C	1.1633278809	-0.3157978193	0.4797749279
H	0.8047701313	0.8383959166	-1.2917054733
H	2.8367711159	-0.3172341416	-0.5154095443

Energy of Optimized Geometry: -307.656744748562

OX4 3,4-epoxybutanol ωB97X-D STRUC16

C	-2.1610338031	0.1805087843	-0.2548269077
C	-0.7292720604	-0.0324544379	-0.4335107908
H	-0.1404104203	1.4535360730	0.9719805747
H	-2.5024851213	1.0517660459	0.2954411751
H	-2.8627985377	-0.2206600380	-0.9778447364
O	2.1878716741	-0.3433888012	-0.5611734257
H	2.2147187738	0.9219520948	1.0823363894
H	1.2889464539	-0.5753261956	1.2980127905
C	0.2918815369	0.9184504486	0.1244881137
O	-1.4697885326	-0.7876380379	0.5193189262
H	-0.4045361072	-0.6122274195	-1.2912850420
C	1.5525695218	0.2086445339	0.5808703929
H	0.5554373432	1.6542432162	-0.6387251564
H	2.9074410496	-0.9060516797	-0.2783185612

Energy of Optimized Geometry: -307.659150348622

OX4 3,4-epoxybutanol ωB97X-D STRUC17

C	-1.7252998195	-0.1160392692	-0.8224654987
C	-0.6867518802	-0.3468946095	0.1779297154
H	0.6117650144	0.6332580278	1.5568833056
H	-1.7638247626	0.8401452909	-1.3356857767
H	-2.1569155747	-0.9532735111	-1.3597993438
O	2.2062260156	-0.6726046251	-0.0400241558
H	1.3310883944	0.6228082421	-1.4048051049
H	2.2723927579	1.4000749364	-0.1195772047
C	0.3403703278	0.7031579465	0.5016631123
O	-2.0240725703	-0.0667822787	0.5639425979
H	-0.3807290697	-1.3717001148	0.3590301451
C	1.5935064054	0.5703634369	-0.3415415583
H	-0.0977007696	1.6898137043	0.3397501895
H	2.9865640540	-0.7780464025	-0.5829248440

Energy of Optimized Geometry: -307.658218426568

OX4 3,4-epoxybutanol ωB97X-D STRUC18

C	-1.4577242732	-0.8682942377	0.2304106581
C	-0.7904201402	0.4257772507	0.1892970569
H	0.3881000092	1.6855023042	-1.0568138574
H	-1.0597588925	-1.6802338958	-0.3680002331
H	-2.0162122966	-1.1611558973	1.1124700813
O	1.8446693273	-0.8352658955	0.5335392812
H	2.5646504943	0.8114897754	-0.5033259313
H	1.7087301889	1.1813624935	1.0012660694
C	0.4308102884	0.6711320746	-0.6531965654
O	-2.0104104319	0.2160265843	-0.5039218482
H	-0.8871251370	1.0692295010	1.0614209236
C	1.7205462633	0.5170486354	0.1284000355
H	0.4310345846	-0.0178483303	-1.4992935562
H	2.6534491407	-0.9390589170	1.0335008662

Energy of Optimized Geometry: -307.658220518571

OX4 3,4-epoxybutanol ωB97X-D STRUC19

C	-2.2444422394	0.2681879555	0.3100746490
C	-0.9524645429	0.4549082771	-0.3407013503
H	0.2112858426	-0.0814691283	1.3655356587
H	-2.2772157317	0.1187280709	1.3842886190
H	-3.1393071117	0.6909901250	-0.1331874185
O	2.6500019802	-0.0815345928	0.4031501242
H	1.2168143949	-1.2221280680	-0.5792983464
H	1.5827878254	0.3431091056	-1.3254422999
C	0.3386943119	0.4577216297	0.4257517559
O	-1.6438708099	-0.7850764570	-0.4274158914
H	-0.9423175557	1.0073872254	-1.2775819487
C	1.4650709584	-0.1774524342	-0.3669517334
H	0.6113766055	1.4871907372	0.6690129746
H	3.3564412426	-0.5369620840	-0.0536668142

Energy of Optimized Geometry: -307.658365150211

OX4 3,4-epoxybutanol ωB97X-D STRUC20

C	-1.8612226255	-0.2343175853	0.5372446110
C	-1.0770030105	0.6133070397	-0.3598875348
H	0.6149852127	1.7744902136	0.1646049240
H	-1.3693677090	-0.8034012794	1.3181584123
H	-2.9023059094	0.0054376215	0.7246002068
O	2.5681894456	-0.1707882082	0.3402502012
H	0.8928351109	-0.2523684363	1.5526458886
H	0.9009583841	-1.2657230480	0.1078420207
C	0.4169922974	0.7944472216	-0.2805165570
O	-1.5646464923	-0.6491775179	-0.7869127554
H	-1.5988191212	1.4487675190	-0.8191648164
C	1.1663787427	-0.2819997943	0.4923569599
H	0.8128970817	0.8246974316	-1.2982454417
H	2.8538835925	0.6673648538	0.7043099680

Energy of Optimized Geometry: -307.656299261472

OX4 3,4-epoxybutanol ωB97X-D STRUC21

C	-1.3860554248	-0.6733181117	0.4837020730
C	-0.9738598563	0.6787594887	0.1243998825
H	0.8067393300	1.6331589633	0.7933974667
H	-0.6310900167	-1.4395354674	0.6089066175
H	-2.3087989528	-0.8210890352	1.0345249557
O	1.7496152033	-0.7155614127	0.7554833046
H	1.0028071421	-0.6620334008	-1.1862735498
H	2.3428651875	0.4474775447	-0.8525327619
C	0.4554186392	1.1069062194	-0.0968393049
O	-1.5501109240	-0.1867455570	-0.8433462111
H	-1.6337282468	1.4901179510	0.4205034793
C	1.4370009129	0.0013180385	-0.4312386685
H	0.4602649458	1.8306637623	-0.9157113280
H	2.3610544770	-1.4187198471	0.5388013627

Energy of Optimized Geometry: -307.658044860174

OX4 3,4-epoxybutanol ωB97X-D STRUC22

C	-1.9317544586	0.2120431913	0.6222409760
C	-0.9177788635	0.3305233659	-0.4229857921
H	0.7524493635	-0.6468583366	-1.3238089734
H	-1.7262772290	-0.4120907672	1.4862597287
H	-2.6478520185	1.0112447111	0.7787109165
O	2.6843138846	-0.1898479067	0.3303123018
H	1.5539627366	1.4552834710	-0.2339694714
H	1.1474072610	0.7671765073	1.3459133245
C	0.4103165878	-0.3662782605	-0.3258942113
O	-2.0616850199	-0.4858947868	-0.6061365613
H	-0.9298677067	1.2345078319	-1.0276061979
C	1.4579393741	0.5164178178	0.3248946728
H	0.2963603608	-1.2857262291	0.2498606528
H	3.3561467378	0.3530566972	0.7415751742

Energy of Optimized Geometry: -307.657348715351

OX4 3,4-epoxybutanol ωB97X-D STRUC23

C	-1.3857842704	-0.7260113864	0.4422723746
C	-0.9752271043	0.6368408886	0.0947475434
H	0.7843794123	1.7501242057	0.5430112355
H	-0.6605359431	-1.5341215156	0.4682362354
H	-2.2649695322	-0.8793306885	1.0589746584
O	1.9127343555	-0.5600703989	0.8553447217
H	1.0442458905	-0.8542818016	-1.0121414877
H	2.3390031054	0.3284381682	-0.8869565275
C	0.4342216227	1.0663787426	-0.2331787424
O	-1.6310080046	-0.1978739736	-0.8477866764
H	-1.6103628626	1.4368362803	0.4656436556
C	1.4520480888	-0.0537867543	-0.3849583709
H	0.3876760475	1.6389645907	-1.1630581758
H	1.1620299021	-0.8525235108	1.3721872878

Energy of Optimized Geometry: -307.656714949743

OX4 3,4-epoxybutanol ωB97X-D STRUC24

C	-1.8752753645	-0.2445071700	0.4815615042
C	-1.0589369349	0.6192432923	-0.3695872648
H	0.6310140171	1.7520585540	0.2372458979
H	-1.4099740068	-0.8371130854	1.2616540997
H	-2.9193601700	-0.0011790429	0.6468605669
O	2.5399040344	-0.0715646759	0.3907942482
H	0.8539942287	-0.2899743539	1.5732952489
H	0.8754132904	-1.2816657519	0.1016702000
C	0.4314274320	0.7912565492	-0.2420566062
O	-1.5451532536	-0.6284503002	-0.8441661966
H	-1.5613329525	1.4700880192	-0.8218201710
C	1.1480980402	-0.3068988135	0.5179516846
H	0.8575595852	0.8477172398	-1.2462547242
H	3.0152932162	-0.7792693090	0.8243971883

Energy of Optimized Geometry: -307.656692774329

OX4 3,4-epoxybutanol ωB97X-D STRUC25

C	-2.1496188921	0.1939381665	0.1277602654
C	-0.7960676776	0.2884729510	-0.4111429519
H	0.2367927815	0.6236733161	1.4188492967
H	-2.3434904649	0.5512060518	1.1345789688
H	-3.0022471237	0.1992103244	-0.5428240572
O	1.9140433569	-0.8217315109	0.0187067069
H	1.7793581509	0.8474469518	-1.2045796069
H	2.4562428126	1.1428946953	0.4043789819
C	0.3242750087	0.9196129628	0.3719612774
O	-1.3152258447	-0.9482739328	0.0443915535
H	-0.6911151787	0.3427728906	-1.4928674312
C	1.7046789460	0.5662330626	-0.1462893365
H	0.2141874358	2.0064072886	0.3237499534
H	2.7619813116	-1.0580412735	-0.3555493281

Energy of Optimized Geometry: -307.655455928682

7 2,3-epoxybutanol (OX5)

7.1 BMK

OX5 2,3-epoxybutanol BMK STRUC1

C	-2.4007054539	0.0909144279	-0.4084967934
C	-0.9250318925	-0.2192445965	-0.3505166828
H	-0.3413141115	1.5774408361	0.7929745662
O	1.9622765118	-0.5449705442	-0.5057951958
H	1.6471345254	-1.2176542263	0.1053175596
H	-2.6453623370	0.5868791613	-1.3501945345
H	-2.9848023086	-0.8292765948	-0.3455162368
H	-2.6868810423	0.7408188593	0.4192879801
C	0.0392041526	0.6709358815	0.3267948287
O	-0.3972351448	-0.5368140599	0.9471812786
H	-0.5311697026	-0.8584419809	-1.1354345732
C	1.5166574033	0.6945693852	-0.0319151653
H	2.0857981253	1.0070288945	0.8537675554
H	1.6918624633	1.4310798055	-0.8189579621

Energy of Optimized Geometry: -307.572383356310

OX5 2,3-epoxybutanol BMK STRUC2

C	-2.5374708684	0.4013441914	-0.0495524732
C	-1.1970707146	-0.2185746919	-0.3624641647
H	0.0371070999	1.3583770991	0.5812433283
O	2.4461189613	0.0288021087	0.2103173774
H	2.2374272651	-0.6684422204	0.8364193756
H	-2.8950547796	0.9815326244	-0.9026755334
H	-3.2724959649	-0.3757154088	0.1685772877
H	-2.4580641780	1.0572260813	0.8178111068
C	0.0784260186	0.4233905256	0.0270887279
O	-0.4427655317	-0.6961773849	0.7499957114
H	-1.1711652212	-0.8899958057	-1.2179564230
C	1.3754005923	0.1434985958	-0.6955367080
H	1.6201021483	0.9705562173	-1.3643442023
H	1.2635533547	-0.7678654235	-1.2950334353

Energy of Optimized Geometry: -307.570936249354

OX5 2,3-epoxybutanol BMK STRUC3

C	-2.3248625588	0.1148550719	-0.6265904098
C	-1.0398720672	-0.4421513454	-0.0630290287
H	0.0446477883	1.4837408950	0.0536471120
O	2.2036826471	0.1302847265	-0.9640367669
H	1.7493554461	-0.3200633906	-1.6778785549
H	-2.4221770437	1.1706464386	-0.3717165135
H	-2.3423183602	0.0092994550	-1.7133143753
H	-3.1828915963	-0.4213106974	-0.2163627128
C	0.1302249633	0.4168764224	0.2494416122
O	-0.7226275637	-0.0703588789	1.2756006893
H	-0.8390661602	-1.4913394100	-0.2738163509
C	1.5422822402	-0.1342313946	0.2508146073
H	1.5078291397	-1.2059453536	0.4791255077
H	2.1282638500	0.3555601078	1.0302857509

Energy of Optimized Geometry: -307.568524959870

OX5 2,3-epoxybutanol BMK STRUC4

C	-2.3376130618	0.0955566506	-0.6139156871
C	-1.0455938319	-0.4558496211	-0.0627019913
H	0.0224151181	1.4726119906	0.0448320892
O	2.1882144518	0.1027001227	-0.9918109099
H	2.4185187210	1.0296758852	-1.0628214210
H	-2.3561311307	-0.0012956428	-1.7013007565
H	-3.1895725880	-0.4516394417	-0.2056601022
H	-2.4435281911	1.1483061937	-0.3492602928
C	0.1196713284	0.4040678678	0.2373083517
O	-0.7173548101	-0.0821862868	1.2777295277
H	-0.8382124533	-1.5010778449	-0.2806062363
C	1.5369687456	-0.1360944806	0.2348838588
H	1.5064014958	-1.2172863432	0.3761305411
H	2.0944344716	0.2943692015	1.0750092540

Energy of Optimized Geometry: -307.567551870210

OX5 2,3-epoxybutanol BMK STRUC5

C	-2.1842467831	0.4793336189	-0.9221740729
C	-1.0320845553	-0.3226272720	-0.3686243568
H	0.0643538016	1.3838663931	0.5018291263
O	2.3334009993	0.2025244862	-0.5448184799
H	2.6046886934	1.0839776145	-0.2863284316
H	-3.1140357254	-0.0876153764	-0.8447051484
H	-2.2993847503	1.4105250298	-0.3661647646
H	-2.0108230552	0.7127700121	-1.9745763332
C	0.0886322948	0.3024275694	0.3668807741
O	-0.9381856675	-0.3972975341	1.0561298361
H	-0.8244781121	-1.2687800995	-0.8632517404
C	1.4639363487	-0.3345157140	0.4254564724
H	1.3716891622	-1.4013009111	0.2174479967
H	1.8750475664	-0.2260606359	1.4359589345

Energy of Optimized Geometry: -307.567551852644

OX5 2,3-epoxybutanol BMK STRUC6

C	-2.3184695048	0.1071904181	-0.6405234724
C	-1.0393997565	-0.4488665589	-0.0646312700
H	0.0547220213	1.4703896852	0.0207760589
O	2.1234074090	0.1366363729	-0.9886138482
H	3.0561221139	-0.0775824696	-0.9639866428
H	-2.4153457776	1.1650971949	-0.3940158425
H	-2.3236677102	-0.0060202824	-1.7264335220
H	-3.1815666346	-0.4242234971	-0.2346833668
C	0.1291226181	0.4065596011	0.2348530527
O	-0.7223140959	-0.0574989729	1.2730678157
H	-0.8397564880	-1.4994914191	-0.2632746676
C	1.5350553836	-0.1476287141	0.2612574939
H	1.4890546734	-1.2251911064	0.4567169945
H	2.0905618460	0.3262874912	1.0794635286

Energy of Optimized Geometry: -307.567932584734

OX5 2,3-epoxybutanol BMK STRUC7

C	-2.5307145663	0.4536067876	-0.1085746766
C	-1.2103797498	-0.2356889165	-0.3577454871
H	0.0525824497	1.3061642994	0.5917330438
O	2.4985515130	0.0648809402	0.1851202405
H	2.6229041186	0.9444439614	0.5443986511
H	-2.4515082421	1.1249601876	0.7470998681
H	-2.8316945915	1.0295255105	-0.9861668650
H	-3.3085743484	-0.2833260420	0.1005473840
C	0.0832997806	0.3514512162	0.0659677610
O	-0.5145574771	-0.7177642512	0.7863887168
H	-1.1864614036	-0.9264810873	-1.1983435748
C	1.3737309678	0.0492080410	-0.6620372258
H	1.4928858392	0.7591028991	-1.4910619197
H	1.3183946837	-0.9579496931	-1.0770674446

Energy of Optimized Geometry: -307.566890477784

OX5 2,3-epoxybutanol BMK STRUC9

C	-2.5161965761	0.3608805058	-0.3207013898
C	-1.2003526832	-0.3763697732	-0.2441510533
H	0.0798552036	1.4114509498	0.0246224898
O	2.4382130642	0.2949620048	0.1623895908
H	3.2694996667	-0.0891001650	-0.1175409742
H	-2.7966693715	0.5327197343	-1.3620246414
H	-3.3060507102	-0.2238824300	0.1548012242
H	-2.4409349073	1.3213513573	0.1899967630
C	0.0900617604	0.3280048183	-0.0707471054
O	-0.5292407139	-0.3582482005	1.0111147974
H	-1.1731390360	-1.3488615483	-0.7324209868
C	1.3798884715	-0.2508837571	-0.5930397493
H	1.4829872256	0.0086316321	-1.6548318444
H	1.3375451638	-1.3417508891	-0.4977706297

Energy of Optimized Geometry: -307.567326707227

7.2 CAM-B3LYP

OX5 2,3-epoxybutanol CAM-B3LYP STRUC1

C	-2.3829863078	0.0654938428	-0.4090027002
C	-0.9175727278	-0.2269922602	-0.3401617960
H	-0.3592280684	1.5721533330	0.7731297898
O	1.9668231532	-0.5247807008	-0.5115754157
H	1.6707646803	-1.2013061413	0.1076810878
H	-2.6295192666	0.5467963006	-1.3558451445
H	-2.9602643466	-0.8562614187	-0.3407692694
H	-2.6862852079	0.7208842254	0.4055654246
C	0.0279093925	0.6647943984	0.3172007858
O	-0.3915823321	-0.5380486914	0.9592719651
H	-0.5219551467	-0.8692461575	-1.1192718126
C	1.4900654789	0.7120772022	-0.0377793695
H	2.0556039642	1.0364566972	0.8430825005
H	1.6555926946	1.4471566128	-0.8256913243

Energy of Optimized Geometry: -307.625801115841

OX5 2,3-epoxybutanol CAM-B3LYP STRUC2

C	-2.5132706455	0.4308668108	-0.0604572775
C	-1.1909849359	-0.2032281998	-0.3595398430
H	0.0490269684	1.3335951620	0.5924247053
O	2.4500039844	0.0354541200	0.2043407701
H	2.2637423179	-0.6660099332	0.8353118701
H	-2.8528273757	1.0220958488	-0.9112125843
H	-3.2647823981	-0.3318392293	0.1415940656
H	-2.4402978099	1.0795537953	0.8102348313
C	0.0763938063	0.4026414601	0.0340233707
O	-0.4580084174	-0.7124852911	0.7537666735
H	-1.1746335000	-0.8672229100	-1.2192240880
C	1.3572042739	0.1270980124	-0.6896578410
H	1.5878284449	0.9464423647	-1.3699540558
H	1.2538580167	-0.7896101931	-1.2776740781

Energy of Optimized Geometry: -307.624895448377

OX5 2,3-epoxybutanol CAM-B3LYP STRUC3

C	-2.3168808681	0.1265932631	-0.5753280258
C	-1.0361817830	-0.4283231940	-0.0349389690
H	0.0536342650	1.4706544637	0.0430678208
O	2.1763072259	0.1200105061	-1.0178919686
H	1.7288278798	-0.3585801556	-1.7198728509
H	-2.4108856949	1.1824887241	-0.3288981698
H	-2.3548020113	0.0155361559	-1.6593255260
H	-3.1707495165	-0.4015867869	-0.1518048421
C	0.1319404243	0.4055768545	0.2413294053
O	-0.6949461686	-0.0615260024	1.3004184131
H	-0.8525156067	-1.4790929059	-0.2459648259
C	1.5296082792	-0.1446628300	0.2142726369
H	1.5045055008	-1.2141100084	0.4410621996
H	2.1322769989	0.3424870029	0.9794055912

Energy of Optimized Geometry: -307.622435291170

OX5 2,3-epoxybutanol CAM-B3LYP STRUC4

C	-2.3191787494	0.0923032345	-0.5893516823
C	-1.0338584805	-0.4472841854	-0.0464429313
H	0.0202057246	1.4652135277	0.0477228410
O	2.1588201883	0.0985120529	-1.0292030880
H	2.4130285971	1.0212747038	-1.0973144582
H	-2.3437913333	-0.0078723728	-1.6746129476
H	-3.1675789425	-0.4552636965	-0.1795922745
H	-2.4341153621	1.1436833612	-0.3318377398
C	0.1182897829	0.3983983338	0.2385398709
O	-0.7033166874	-0.0817066509	1.2960684441
H	-0.8299063687	-1.4911085014	-0.2669898519
C	1.5246284582	-0.1309875264	0.2175033154
H	1.5067622572	-1.2086818592	0.3686305628
H	2.0975924639	0.3087236066	1.0389164578

Energy of Optimized Geometry: -307.621728719749

OX5 2,3-epoxybutanol CAM-B3LYP STRUC5

C	-2.1732494938	0.4589658291	-0.8951459334
C	-1.0235686552	-0.3233173684	-0.3437651203
H	0.0616658507	1.3802571093	0.4912204447
O	2.3089788800	0.2084585848	-0.5941168277
H	2.6046104850	1.0848120864	-0.3385074263
H	-3.0990980586	-0.1093313603	-0.8088612120
H	-2.2957586245	1.3959718724	-0.3547725115
H	-2.0098415060	0.6812646846	-1.9498562627
C	0.0875968452	0.2998778693	0.3637751692
O	-0.9246067336	-0.3959562085	1.0810553319
H	-0.8191137959	-1.2704529456	-0.8346943439
C	1.4565114994	-0.3188344217	0.4082256685
H	1.3753634775	-1.3883063911	0.2228609491
H	1.8897140987	-0.1879222646	1.4039628922

Energy of Optimized Geometry: -307.621728732918

OX5 2,3-epoxybutanol CAM-B3LYP STRUC6

C	-2.3012578712	0.1145151150	-0.6146947401
C	-1.0302276815	-0.4359593040	-0.0497423353
H	0.0568667494	1.4628873318	0.0264637710
O	2.0977910687	0.1254137357	-1.0243972705
H	3.0327776668	-0.0871064138	-1.0046012258
H	-2.4039512950	1.1703035561	-0.3709854052
H	-2.3132449378	0.0026492127	-1.6989794185
H	-3.1622882226	-0.4163947720	-0.2092909273
C	0.1290360518	0.3999210608	0.2363331127
O	-0.7087961307	-0.0570613523	1.2906762318
H	-0.8378656380	-1.4853766288	-0.2539687940
C	1.5230091354	-0.1488952185	0.2429943998
H	1.4858185014	-1.2228295042	0.4451910346
H	2.0943382582	0.3295244809	1.0441984687

Energy of Optimized Geometry: -307.622153760209

OX5 2,3-epoxybutanol CAM-B3LYP STRUC7

C	-2.5160109510	0.4178569003	-0.1057252087
C	-1.1979303397	-0.2503482179	-0.3455368253
H	0.0342137008	1.2975208168	0.5829480458
O	2.4920810685	0.1146995252	0.1688709933
H	2.6070997319	1.0065066197	0.5054231673
H	-2.4541874973	1.0955122229	0.7437161917
H	-2.8240238672	0.9841330683	-0.9851221315
H	-3.2861860427	-0.3240347149	0.1038938519
C	0.0724509244	0.3419749885	0.0628419333
O	-0.5010147770	-0.7306434324	0.8001409824
H	-1.1715021351	-0.9433905397	-1.1824194280
C	1.3508497105	0.0646983042	-0.6673400488
H	1.4486718150	0.7670196878	-1.5029280094
H	1.3205132336	-0.9444767868	-1.0736797051

Energy of Optimized Geometry: -307.621080846419

OX5 2,3-epoxybutanol CAM-B3LYP STRUC9

C	-2.5190610889	0.3144738000	-0.1298338681
C	-1.1993154574	-0.3924223966	-0.1402397502
H	0.0541339330	1.4012466901	0.0028422647
O	2.4310966469	0.3448186385	-0.0439816932
H	3.2399802044	-0.0401552033	-0.3868155430
H	-2.8788809652	0.4684515319	-1.1475892189
H	-3.2630606852	-0.2753583415	0.4050951342
H	-2.4318546948	1.2819409501	0.3608801483
C	0.0727579116	0.3186246149	-0.0794161193
O	-0.4400494325	-0.3580927524	1.0640639642
H	-1.1983814062	-1.3690874462	-0.6178085093
C	1.3146667498	-0.2357444281	-0.6966059666
H	1.3228277246	0.0081801852	-1.7649965615
H	1.3101164923	-1.3232681444	-0.5853712736

Energy of Optimized Geometry: -307.621651901982

7.3 M06-2X

OX5 2,3-epoxybutanol M06-2X STRUC1

C	-2.3800120841	0.0755072240	-0.4023703716
C	-0.9127796789	-0.2228651621	-0.3398923481
H	-0.3531374259	1.5803645245	0.7760748086
O	1.9445739640	-0.5435372452	-0.5199540863
H	1.6307794055	-1.2138531492	0.0976702585
H	-2.6287434529	0.5584471934	-1.3473831149
H	-2.9593378010	-0.8445787565	-0.3281976942
H	-2.6693363786	0.7322692922	0.4167624942
C	0.0348784968	0.6728246857	0.3217921203
O	-0.3897376857	-0.5268182867	0.9561070467
H	-0.5130930737	-0.8631772541	-1.1190262961
C	1.4999480746	0.7003131680	-0.0367525841
H	2.0729059514	1.0049777701	0.8463102135
H	1.6753730908	1.4377179489	-0.8201965457

Energy of Optimized Geometry: -307.637860966894

OX5 2,3-epoxybutanol M06-2X STRUC2

C	-2.5188511600	0.3968141851	-0.0393812426
C	-1.1885192301	-0.2175930050	-0.3556857331
H	0.0306169179	1.3602094096	0.5675047938
O	2.4336932237	0.0259192911	0.2047431310
H	2.2076858327	-0.6620971721	0.8381876654
H	-2.8825824006	0.9753252482	-0.8885159529
H	-3.2517658132	-0.3777736319	0.1849748165
H	-2.4342804346	1.0529605495	0.8253316019
C	0.0733407361	0.4230541428	0.0200526354
O	-0.4327565180	-0.6912976127	0.7521227556
H	-1.1642491231	-0.8896070536	-1.2089242683
C	1.3572852508	0.1449764577	-0.7027974006
H	1.6034784877	0.9691965902	-1.3716208395
H	1.2435845358	-0.7655760597	-1.2997740656

Energy of Optimized Geometry: -307.636293728553

OX5 2,3-epoxybutanol M06-2X STRUC3

C	-2.2979897566	0.1153453712	-0.6216734005
C	-1.0218318608	-0.4361320008	-0.0605279600
H	0.0346771778	1.4838357216	0.0685603765
O	2.1741807291	0.1224794867	-0.9836434917
H	1.6889528211	-0.3267204385	-1.6803863268
H	-2.4029012918	1.1651453715	-0.3523663365
H	-2.3085924683	0.0270625552	-1.7080681505
H	-3.1550578045	-0.4293905283	-0.2262966599
C	0.1278207060	0.4182380530	0.2589447329
O	-0.7182938777	-0.0791447022	1.2812393851
H	-0.8114718088	-1.4799124511	-0.2819625024
C	1.5305830468	-0.1238406748	0.2512301329
H	1.5012597896	-1.1904800603	0.4946008121
H	2.1293706277	0.3769176473	1.0111460011

Energy of Optimized Geometry: -307.633805071784

OX5 2,3-epoxybutanol M06-2X STRUC4

C	-2.3111538916	0.1059124489	-0.6116903634
C	-1.0309160411	-0.4455930889	-0.0615516544
H	0.0187752560	1.4728038600	0.0587640896
O	2.1632620481	0.0837363662	-1.0056216772
H	2.3733640627	1.0155241017	-1.0983339653
H	-2.3251504790	0.0208237656	-1.6981231396
H	-3.1650546389	-0.4422284086	-0.2140949765
H	-2.4177166338	1.1545414807	-0.3376221089
C	0.1187006971	0.4050302922	0.2458640502
O	-0.7157127119	-0.0869440478	1.2808136715
H	-0.8181893553	-1.4861501799	-0.2895337353
C	1.5244298955	-0.1314911202	0.2385867519
H	1.4939715519	-1.2084161852	0.3991347053
H	2.0953214768	0.3115449288	1.0602171417

Energy of Optimized Geometry: -307.632773219313

OX5 2,3-epoxybutanol M06-2X STRUC5

C	-2.1333334189	0.5048048023	-0.9597938008
C	-1.0165948897	-0.3041665612	-0.3731007574
H	0.0738392220	1.3891445813	0.4872617991
O	2.3231438136	0.1389775814	-0.5310884084
H	2.5895777327	1.0337870086	-0.3090283483
H	-3.0741185201	-0.0417570372	-0.8973507767
H	-2.2439563587	1.4428604621	-0.4176633030
H	-1.9332203848	0.7235906578	-2.0085299317
C	0.0850874656	0.3068595648	0.3702435039
O	-0.9623442911	-0.3647645824	1.0493268949
H	-0.8100437816	-1.2570648682	-0.8519848727
C	1.4375366087	-0.3464041003	0.4601419127
H	1.3265139547	-1.4168410771	0.2917912741
H	1.8505649405	-0.2084313271	1.4639735650

Energy of Optimized Geometry: -307.632773213515

OX5 2,3-epoxybutanol M06-2X STRUC6

C	-2.2906879871	0.1014181142	-0.6463460109
C	-1.0212501155	-0.4460266168	-0.0682068153
H	0.0377486345	1.4720379702	0.0400098719
O	2.1012804961	0.1361908186	-0.9956341943
H	3.0357703498	-0.0775540897	-0.9689562799
H	-2.3995764428	1.1528228091	-0.3846647669
H	-2.2841864005	0.0066515488	-1.7319993332
H	-3.1527382021	-0.4415928363	-0.2593547291
C	0.1235293251	0.4090090296	0.2455234119
O	-0.7255516098	-0.0714870902	1.2738301689
H	-0.8064176986	-1.4897235413	-0.2796518792
C	1.5227004168	-0.1315859120	0.2690122732
H	1.4831730950	-1.2046105340	0.4794489578
H	2.0857149850	0.3550384197	1.0715659918

Energy of Optimized Geometry: -307.633181037912

OX5 2,3-epoxybutanol M06-2X STRUC7

C	-2.5157745472	0.4258280392	-0.1054073341
C	-1.1984056907	-0.2473286703	-0.3501998973
H	0.0337364590	1.3136388432	0.5675403057
O	2.4855269323	0.0846777697	0.1807700685
H	2.5833130097	0.9645702589	0.5524597556
H	-2.4399744691	1.1030949770	0.7438733893
H	-2.8256585515	0.9922115757	-0.9836676547
H	-3.2854641873	-0.3143960812	0.1120425380
C	0.0749305842	0.3531366376	0.0560675501
O	-0.4994449222	-0.7117119549	0.7949307562
H	-1.1677889060	-0.9444540990	-1.1835284471
C	1.3551123975	0.0583079487	-0.6684855734
H	1.4720654489	0.7625785993	-1.4997173750
H	1.3060394326	-0.9501181353	-1.0772889088

Energy of Optimized Geometry: -307.632223948870

OX5 2,3-epoxybutanol M06-2X STRUC9

C	-2.4959081515	0.3674529775	-0.3245894440
C	-1.1913432650	-0.3679377896	-0.2462292624
H	0.0751286644	1.4096493396	0.0138947806
O	2.4279069750	0.2792809175	0.1748736298
H	3.2541132279	-0.1189818993	-0.1057674442
H	-2.7739180264	0.5449244677	-1.3635845827
H	-3.2886198836	-0.2135637500	0.1463178574
H	-2.4183533665	1.3244327562	0.1889341341
C	0.0857571945	0.3272662870	-0.0762540004
O	-0.5272867520	-0.3522684913	1.0083214710
H	-1.1634415424	-1.3387241745	-0.7347977436
C	1.3641399942	-0.2528141114	-0.5931968864
H	1.4778242298	0.0119619844	-1.6502444896
H	1.3128380076	-1.3420496874	-0.5052398170

Energy of Optimized Geometry: -307.632632772168

7.4 MN15

OX5 2,3-epoxybutanol MN15 STRUC1

C	-2.3667484870	0.0859488493	-0.4356343048
C	-0.9011917089	-0.2148598237	-0.3620944533
H	-0.3688065154	1.5672967660	0.8000237970
O	1.9380022171	-0.5409904419	-0.4891351965
H	1.5794966746	-1.2182575282	0.1011093259
H	-2.6052951072	0.6012928994	-1.3660268813
H	-2.9505609487	-0.8332890248	-0.3982473406
H	-2.6620647617	0.7166287337	0.4022087054
C	0.0301273860	0.6695843876	0.3353380970
O	-0.3977919149	-0.5449880699	0.9334451096
H	-0.4865870214	-0.8329401780	-1.1516449728
C	1.4993999398	0.7013958827	-0.0039094690
H	2.0617945306	0.9975649958	0.8892153516
H	1.6867521769	1.4478640756	-0.7760029703

Energy of Optimized Geometry: -307.414511534715

OX5 2,3-epoxybutanol MN15 STRUC2

C	-2.5140879805	0.3904757463	-0.0183574752
C	-1.1849253823	-0.2185734530	-0.3464786982
H	0.0263727699	1.3821199996	0.5490592226
O	2.4185809792	-0.0010918560	0.1908412252
H	2.1702675912	-0.6777467821	0.8321856609
H	-2.8909679561	0.9677120524	-0.8625485730
H	-3.2434970830	-0.3836410063	0.2176328979
H	-2.4178140367	1.0484579067	0.8441212138
C	0.0722650531	0.4385655612	0.0127432393
O	-0.4147894462	-0.6734986573	0.7572490147
H	-1.1611029539	-0.8942664593	-1.1972651692
C	1.3501149005	0.1493485569	-0.7167657287
H	1.6102663833	0.9713876950	-1.3827696702
H	1.2123335210	-0.7547862633	-1.3205787175

Energy of Optimized Geometry: -307.412392180265

OX5 2,3-epoxybutanol MN15 STRUC3

C	-2.2851065814	0.1035071260	-0.6391734919
C	-1.0115394016	-0.4402894148	-0.0665557555
H	0.0211992323	1.4923604385	0.0713238231
O	2.1682597344	0.1185758665	-0.9733160466
H	1.6838585739	-0.3343727182	-1.6712887584
H	-2.3979876077	1.1503069176	-0.3602051845
H	-2.2840575673	0.0273229958	-1.7265270798
H	-3.1443629322	-0.4487122678	-0.2598084371
C	0.1230486936	0.4279103595	0.2649035726
O	-0.7259696136	-0.0811534335	1.2760472266
H	-0.7843748804	-1.4804111771	-0.2914078762
C	1.5273429294	-0.1098214425	0.2637140021
H	1.4907330359	-1.1736420924	0.5224281014
H	2.1264699198	0.3958055108	1.0197602432

Energy of Optimized Geometry: -307.409712830939

OX5 2,3-epoxybutanol MN15 STRUC4

C	-2.3009102857	0.0881059873	-0.6306479052
C	-1.0226367690	-0.4554609104	-0.0698398531
H	0.0016848659	1.4769028223	0.0585723991
O	2.1653185697	0.0982617133	-0.9875216339
H	2.3826166994	1.0300129310	-1.0859871872
H	-2.3032352610	0.0167986529	-1.7181273755
H	-3.1567010169	-0.4686719158	-0.2500137223
H	-2.4160295886	1.1332151181	-0.3452476035
C	0.1114040527	0.4103565938	0.2494449890
O	-0.7250570769	-0.0943608209	1.2733065574
H	-0.7928165527	-1.4919227106	-0.3017220634
C	1.5203450050	-0.1176505651	0.2506503298
H	1.4860587003	-1.1943241484	0.4155270570
H	2.0843760092	0.3249108557	1.0774228179

Energy of Optimized Geometry: -307.408758849121

OX5 2,3-epoxybutanol MN15 STRUC5

C	-2.1077657260	0.4973216076	-1.0044914483
C	-1.0060011996	-0.3087658606	-0.3879918467
H	0.0631828112	1.3990702489	0.4720592084
O	2.3269105446	0.1260607049	-0.4909708951
H	2.6077741037	1.0211241032	-0.2788157900
H	-3.0515930255	-0.0457720221	-0.9652207904
H	-2.2281184820	1.4355111022	-0.4638387535
H	-1.8837210934	0.7176674142	-2.0480335182
C	0.0764919785	0.3147449610	0.3717010401
O	-0.9819871233	-0.3516947138	1.0334959479
H	-0.7843177066	-1.2646640593	-0.8548549445
C	1.4251944051	-0.3413771984	0.4909998617
H	1.3035558307	-1.4132308115	0.3363915193
H	1.8230937185	-0.1967260043	1.5000749754

Energy of Optimized Geometry: -307.408758866518

OX5 2,3-epoxybutanol MN15 STRUC6

C	-2.2852974288	0.1024978616	-0.6473671749
C	-1.0178982850	-0.4459735603	-0.0673468028
H	0.0336568011	1.4771289840	0.0350334763
O	2.0987285686	0.1251148348	-0.9939697393
H	3.0355791030	-0.0886845396	-0.9758608448
H	-2.3914627118	1.1525708338	-0.3777108150
H	-2.2770705081	0.0169974731	-1.7337515471
H	-3.1505973062	-0.4401693275	-0.2676688952
C	0.1214798193	0.4151150093	0.2463323896
O	-0.7256055017	-0.0677696596	1.2718020152
H	-0.7965603473	-1.4882821766	-0.2811970866
C	1.5190312355	-0.1298189711	0.2694431899
H	1.4660995462	-1.2013993032	0.4891521502
H	2.0851972903	0.3544637623	1.0713291526

Energy of Optimized Geometry: -307.409181892015

OX5 2,3-epoxybutanol MN15 STRUC7

C	-2.5033586822	0.4628725405	-0.1164235043
C	-1.1978224522	-0.2330657062	-0.3569121889
H	0.0455500666	1.3109566039	0.5745786581
O	2.4852642785	0.0568270311	0.1866793611
H	2.5915838477	0.9324226425	0.5716950916
H	-2.4157801321	1.1282489283	0.7415385676
H	-2.7963673922	1.0474151752	-0.9885475620
H	-3.2917366838	-0.2607635588	0.0884577551
C	0.0783644927	0.3496467033	0.0630058515
O	-0.5153081562	-0.7095947916	0.7903349992
H	-1.1695461615	-0.9240413845	-1.1959711287
C	1.3557290563	0.0387400195	-0.6593495739
H	1.4779431956	0.7354524288	-1.4968478867
H	1.2875435270	-0.9705175513	-1.0643408443

Energy of Optimized Geometry: -307.408254625971

OX5 2,3-epoxybutanol MN15 STRUC9

C	-2.5045309431	0.3622566271	-0.2083930794
C	-1.1984880381	-0.3731335830	-0.1860245639
H	0.0681223207	1.4132551108	0.0071148062
O	2.4340770202	0.2819348880	0.0489158444
H	3.2509847294	-0.1124572476	-0.2691306855
H	-2.8275929610	0.5452080418	-1.2333337494
H	-3.2793899596	-0.2155601763	0.2946703996
H	-2.3994114592	1.3175267840	0.3041327202
C	0.0797989906	0.3300500257	-0.0752697108
O	-0.4802311956	-0.3483566114	1.0354131426
H	-1.1872242675	-1.3442183991	-0.6757891621
C	1.3308200540	-0.2525279263	-0.6528854625
H	1.3852469078	-0.0017384258	-1.7185625751
H	1.2755269014	-1.3413870418	-0.5519074446

Energy of Optimized Geometry: -307.408700599932

7.5 ωB97X-D

OX5 2,3-epoxybutanol ωB97X-D STRUC1

C	-2.3873870385	0.0553171583	-0.4006729190
C	-0.9196084304	-0.2381599414	-0.3284164865
H	-0.3622730259	1.5734892780	0.7679883155
O	1.9722862512	-0.5117597042	-0.5320563585
H	1.6964290410	-1.1903590967	0.0898034406
H	-2.6289354059	0.5322170874	-1.3514068678
H	-2.9651720962	-0.8663377714	-0.3288773041
H	-2.6914633462	0.7164927169	0.4100067204
C	0.0261706708	0.6620128572	0.3192902047
O	-0.3903117432	-0.5337305798	0.9688489487
H	-0.5250032542	-0.8860583801	-1.1049015713
C	1.4893805174	0.7147971234	-0.0454863243
H	2.0573454295	1.0388938839	0.8352530667
H	1.6424093515	1.4579889070	-0.8290744292

Energy of Optimized Geometry: -307.667166460843

OX5 2,3-epoxybutanol ωB97X-D STRUC2

C	-2.5158554997	0.4314365291	-0.0654564672
C	-1.1912870646	-0.2075379935	-0.3577221163
H	0.0458694193	1.3309871430	0.5985353662
O	2.4551656971	0.0418094914	0.2002123765
H	2.2710270099	-0.6596860493	0.8275086466
H	-2.8459620393	1.0238198153	-0.9195853954
H	-3.2708804826	-0.3298090057	0.1313818380
H	-2.4458743971	1.0807420104	0.8060255004
C	0.0755986067	0.3988549194	0.0403432558
O	-0.4623064226	-0.7138276710	0.7547525211
H	-1.1727171539	-0.8706208837	-1.2193526358
C	1.3599885060	0.1273422764	-0.6866913389
H	1.5805218132	0.9487009072	-1.3690042292
H	1.2569145695	-0.7899791267	-1.2768242247

Energy of Optimized Geometry: -307.666149920187

OX5 2,3-epoxybutanol ωB97X-D STRUC3

C	-2.3031867420	0.1176970025	-0.6094625181
C	-1.0265918766	-0.4320221732	-0.0472655846
H	0.0416810889	1.4786000501	0.0686129941
O	2.1708619774	0.1249450447	-1.0013907475
H	1.6958740375	-0.3388687409	-1.6917857708
H	-2.4131995259	1.1705000241	-0.3531447153
H	-2.3131462641	0.0188593740	-1.6956549991
H	-3.1607927886	-0.4250279003	-0.2115068595
C	0.1286026422	0.4116629063	0.2585351954
O	-0.7104811992	-0.0762170770	1.2932988422
H	-0.8279263437	-1.4789173230	-0.2689870427
C	1.5340302104	-0.1286365192	0.2345511512
H	1.5146802184	-1.1969703254	0.4750711068
H	2.1359458834	0.3711458869	0.9930563477

Energy of Optimized Geometry: -307.663930311503

OX5 2,3-epoxybutanol ωB97X-D STRUC4

C	-2.3167379909	0.1028787391	-0.5971308120
C	-1.0349945687	-0.4440678464	-0.0455785429
H	0.0245723691	1.4665965965	0.0540862728
O	2.1576422431	0.0886256248	-1.0269493466
H	2.3726167914	1.0176195336	-1.1089138837
H	-2.3314259330	0.0040284670	-1.6830731009
H	-3.1701750489	-0.4427211816	-0.1940867385
H	-2.4288348378	1.1552496434	-0.3388483441
C	0.1195922639	0.3981801356	0.2448608855
O	-0.7063107812	-0.0815340157	1.2940991950
H	-0.8339087852	-1.4887194208	-0.2693769679
C	1.5288866008	-0.1342725631	0.2200960391
H	1.5098410561	-1.2122784417	0.3763509360
H	2.1039808341	0.3078538152	1.0404676013

Energy of Optimized Geometry: -307.663002919605

OX5 2,3-epoxybutanol ωB97X-D STRUC5

C	-2.1435403053	0.5099033697	-0.9379024989
C	-1.0254150020	-0.3015979687	-0.3570283429
H	0.0853259006	1.3806779444	0.4915103555
O	2.3202848107	0.1480165972	-0.5573070274
H	2.5916241319	1.0346994845	-0.3201265979
H	-3.0857358673	-0.0345416734	-0.8714524122
H	-2.2534384025	1.4528228580	-0.4034696845
H	-1.9470030137	0.7229076106	-1.9891889355
C	0.0878940986	0.2980096884	0.3694319602
O	-0.9528759793	-0.3688892920	1.0657619603
H	-0.8346160281	-1.2553900791	-0.8425852485
C	1.4459788227	-0.3511543367	0.4357933167
H	1.3435951543	-1.4211994885	0.2581574695
H	1.8671235192	-0.2220392077	1.4384465589

Energy of Optimized Geometry: -307.663002906317

OX5 2,3-epoxybutanol ωB97X-D STRUC6

C	-2.2968788259	0.1105755090	-0.6313614391
C	-1.0280158453	-0.4381077787	-0.0527264120
H	0.0510858316	1.4664335333	0.0347347456
O	2.0978171411	0.1270529653	-1.0150862348
H	3.0294675363	-0.0871138159	-0.9887991882
H	-2.4049954204	1.1668021498	-0.3883208581
H	-2.2937058973	-0.0008360986	-1.7160712952
H	-3.1608209675	-0.4240600869	-0.2360334865
C	0.1267873989	0.4023982979	0.2433225165
O	-0.7158484622	-0.0608247926	1.2861581251
H	-0.8301670650	-1.4868052207	-0.2604102594
C	1.5260035087	-0.1428078993	0.2509855301
H	1.4910616618	-1.2172385887	0.4597324104
H	2.0946815703	0.3407011858	1.0527359403

Energy of Optimized Geometry: -307.663223071573

OX5 2,3-epoxybutanol ωB97X-D STRUC7

C	-2.5117971943	0.4568798831	-0.1042102611
C	-1.2020064909	-0.2333889289	-0.3438482411
H	0.0516973195	1.2932846385	0.5943309226
O	2.4961541426	0.0769555418	0.1642846794
H	2.6037796976	0.9582532635	0.5229287546
H	-2.4419952903	1.1283909885	0.7505152141
H	-2.8031420271	1.0342253751	-0.9824840614
H	-3.2954427537	-0.2741028301	0.0959444588
C	0.0772827281	0.3387855964	0.0691334985
O	-0.5126425702	-0.7275250872	0.7961732968
H	-1.1848419283	-0.9206485956	-1.1868620181
C	1.3543714072	0.0506388184	-0.6670978182
H	1.4549728306	0.7615786252	-1.4966027967
H	1.3083678214	-0.9538489695	-1.0861210024

Energy of Optimized Geometry: -307.662470966649

OX5 2,3-epoxybutanol ωB97X-D STRUC9

C	-2.5114857442	0.3573882230	-0.1984317043
C	-1.2009264541	-0.3716309398	-0.1724515096
H	0.0731441487	1.4042174208	0.0192571347
O	2.4398912252	0.2954734857	0.0244227149
H	3.2431837848	-0.0909518746	-0.3224215576
H	-2.8335598286	0.5230567001	-1.2273590944
H	-3.2812741364	-0.2251564858	0.3082077950
H	-2.4245160119	1.3211134539	0.3014249090
C	0.0798940413	0.3209667861	-0.0695469802
O	-0.4758871253	-0.3558990800	1.0490356606
H	-1.2005897897	-1.3446159673	-0.6595753173
C	1.3303530481	-0.2482691011	-0.6641678988
H	1.3656984928	0.0144407508	-1.7287593946
H	1.2992285868	-1.3391192586	-0.5750776267

Energy of Optimized Geometry: -307.662779531630

7.6 EOM-CCSD

OX5 2,3-epoxybutanol EOM-CCSD STRUC1

C	-2.3824340317	0.0912620295	-0.4323681180
C	-0.9121156214	-0.2128083431	-0.3623390554
H	-0.3646613914	1.5660988603	0.8059181611
O	1.9609645525	-0.5364087010	-0.5081806112
H	1.6259038914	-1.2064920545	0.1017318840
H	-2.6188724543	0.5997285087	-1.3705728962
H	-2.9653013066	-0.8311859855	-0.3879116605
H	-2.6796361360	0.7298761522	0.4011480652
C	0.0298961638	0.6694421740	0.3323504235
O	-0.4027901906	-0.5598752221	0.9516231179
H	-0.5053042087	-0.8409799207	-1.1491807707
C	1.5030854538	0.7060294876	-0.0044016108
H	2.0633038175	0.9947706453	0.8941082723
H	1.6898987407	1.4567309048	-0.7753835980

Energy of Optimized Geometry: -307.172365559194

OX5 2,3-epoxybutanol EOM-CCSD STRUC2

C	-2.5259081327	0.4006716932	-0.0693766838
C	-1.1884713337	-0.2148730229	-0.3748502039
H	0.0239214750	1.3568122259	0.5800843361
O	2.4456100593	0.0361196227	0.2297164035
H	2.2120631198	-0.6685643926	0.8448463251
H	-2.8800854558	0.9825585323	-0.9240246680
H	-3.2624259568	-0.3767174327	0.1445988646
H	-2.4507080509	1.0561399789	0.7995270434
C	0.0720469204	0.4240226825	0.0235018795
O	-0.4452151702	-0.7106169178	0.7569695367
H	-1.1546256909	-0.8847690907	-1.2313400618
C	1.3653179200	0.1483955665	-0.6902733078
H	1.6159448784	0.9759420193	-1.3567698554
H	1.2602202022	-0.7645992602	-1.2878772590

Energy of Optimized Geometry: -307.171131642521

OX5 2,3-epoxybutanol EOM-CCSD STRUC3

C	-2.3129466334	0.1380237154	-0.5993275460
C	-1.0319083248	-0.4278916238	-0.0519896836
H	0.0513184467	1.4842281401	0.0590819986
O	2.1815358070	0.1060597861	-1.0117576662
H	1.6988005503	-0.3710453088	-1.6943747809
H	-2.4009258458	1.1934203546	-0.3368336294
H	-2.3391949785	0.0403854993	-1.6876709943
H	-3.1722826698	-0.3948356563	-0.1866712772
C	0.1339702095	0.4164663309	0.2483026127
O	-0.7108998606	-0.0683466839	1.3065333723
H	-0.8385427650	-1.4775208022	-0.2682562458
C	1.5331576300	-0.1440661353	0.2330285034
H	1.4945809280	-1.2135890629	0.4683637439
H	2.1425960313	0.3484076033	0.9920523479

Energy of Optimized Geometry: -307.168755272386

OX5 2,3-epoxybutanol EOM-CCSD STRUC4

C	-2.3206390083	0.1087899602	-0.6013072909
C	-1.0330898389	-0.4450113895	-0.0584757213
H	0.0211017602	1.4773534093	0.0579533086
O	2.1643089335	0.0788407089	-1.0300871837
H	2.4013073941	1.0088453970	-1.0934784144
H	-2.3412235067	0.0200192719	-1.6903230584
H	-3.1735938974	-0.4394946378	-0.1956318094
H	-2.4242092239	1.1605349582	-0.3291172552
C	0.1210202443	0.4079248538	0.2411829408
O	-0.7132208147	-0.0850682439	1.3044624428
H	-0.8241948382	-1.4886026304	-0.2830651359
C	1.5283098463	-0.1337734690	0.2292084731
H	1.4977571855	-1.2123643869	0.3883040342
H	2.1072176557	0.3116015043	1.0458771759

Energy of Optimized Geometry: -307.167935540050

OX5 2,3-epoxybutanol EOM-CCSD STRUC5

C	-2.1639541324	0.4880143326	-0.9152976422
C	-1.0194563650	-0.3137480331	-0.3617207019
H	0.0622395346	1.3871430104	0.5084871087
O	2.3188441993	0.1834769840	-0.5835119352
H	2.5961699249	1.0660421305	-0.3203325344
H	-3.0960968031	-0.0764610725	-0.8431841330
H	-2.2787604392	1.4184303304	-0.3566160042
H	-1.9859172670	0.7249442256	-1.9671636698
C	0.0886669553	0.3066057941	0.3707366469
O	-0.9448229089	-0.4000998760	1.0792659248
H	-0.8096054354	-1.2596856197	-0.8560115374
C	1.4538276225	-0.3314884911	0.4274747290
H	1.3562130140	-1.4019981876	0.2428665130
H	1.8871472297	-0.1991372839	1.4250512248

Energy of Optimized Geometry: -307.167935539777

OX5 2,3-epoxybutanol EOM-CCSD STRUC6

C	-2.2966716371	0.1025864940	-0.6467039059
C	-1.0220316933	-0.4462910810	-0.0698070056
H	0.0397947298	1.4763664510	0.0394499916
O	2.1047170262	0.1329750693	-1.0100217617
H	3.0400484388	-0.0847713299	-0.9662936967
H	-2.4031527598	1.1579184673	-0.3904457123
H	-2.2934481271	0.0005363203	-1.7347082963
H	-3.1593822378	-0.4389552733	-0.2525942610
C	0.1257729770	0.4114925177	0.2413527212
O	-0.7283336004	-0.0692355820	1.2936616174
H	-0.8097380743	-1.4929741524	-0.2764242874
C	1.5266854267	-0.1332814744	0.2679599043
H	1.4872860539	-1.2074654055	0.4796043820
H	2.0940810789	0.3575734099	1.0669160330

Energy of Optimized Geometry: -307.168263529051

OX5 2,3-epoxybutanol EOM-CCSD STRUC7

C	-2.5232178806	0.4295049919	-0.1121385037
C	-1.2014758169	-0.2446194734	-0.3584330960
H	0.0311741375	1.3144416894	0.5750115060
O	2.4960286321	0.0851911012	0.1865308114
H	2.6053527576	0.9821375851	0.5174405183
H	-2.4478123899	1.1091707987	0.7381566310
H	-2.8332724914	0.9961375809	-0.9938851357
H	-3.2937826717	-0.3131466444	0.1056422005
C	0.0735438487	0.3566302154	0.0564363509
O	-0.5031308181	-0.7263844170	0.8048281935
H	-1.1695777665	-0.9424222991	-1.1930032358
C	1.3575954844	0.0632896793	-0.6695267482
H	1.4764665020	0.7738809237	-1.4979104592
H	1.3116762981	-0.9453108509	-1.0820610953

Energy of Optimized Geometry: -307.167132255293

OX5 2,3-epoxybutanol EOM-CCSD STRUC9

C	-2.5087459956	0.3902692785	-0.2458297596
C	-1.2048605244	-0.3587709946	-0.2108901039
H	0.0861451602	1.4128303526	0.0051377376
O	2.4415082474	0.2582816250	0.0982594392
H	3.2477856095	-0.1491947446	-0.2309335583
H	-2.8230352132	0.5619270824	-1.2785676287
H	-3.2899070877	-0.1821871978	0.2590528840
H	-2.4040928582	1.3525275064	0.2576558256
C	0.0859298416	0.3292604258	-0.0855815551
O	-0.4961069282	-0.3498390391	1.0419194765
H	-1.2026768099	-1.3340985925	-0.6942959118
C	1.3430604090	-0.2650010211	-0.6458264873
H	1.4279961288	0.0067698867	-1.7060622696
H	1.2853889198	-1.3556620829	-0.5606184478

Energy of Optimized Geometry: -307.167581916110

8 2,3-epoxypentane (OX6)

8.1 BMK

OX6 2,3-epoxypentane BMK STRUC1

C	-2.3840608445	-0.8406176185	-0.0147298130
C	-1.5366609036	0.4317822123	-0.1758839003
H	2.2782663811	-0.7021961666	1.2288328165
H	2.0998283026	-1.9567971314	-0.0163289801
H	3.1232013414	-0.5447215794	-0.3193915750
H	-1.9233054655	-1.6829019455	-0.5350431561
H	-2.4859814714	-1.1113530194	1.0386380192
H	-3.3856106501	-0.6967018808	-0.4216868265
C	-0.1534651360	0.2530688911	0.4091083494
H	-1.4372200248	0.6987545371	-1.2305814176
H	-2.0249006157	1.2732337581	0.3238369020
C	1.0135413683	-0.1434418943	-0.4136625515
O	0.8369046879	1.2021903152	0.0258395185
H	-0.1207073825	-0.0067913347	1.4668251946
C	2.2002657378	-0.8830820631	0.1561128224
H	0.8385394807	-0.2728278362	-1.4801705842

Energy of Optimized Geometry: -271.653636663750

OX6 2,3-epoxypentane BMK STRUC2

C	-2.5809473874	0.2231581937	0.2888405453
C	-1.4357324063	-0.3688069440	-0.5491694503
H	2.4034379236	-0.7064780196	1.1810885921
H	2.7893144547	-1.4525248377	-0.3845754658
H	3.2709565248	0.2184973535	-0.0551173890
H	-2.7228228871	-0.3457982400	1.2105395374
H	-2.3554220427	1.2561562441	0.5584346860
H	-3.5205944975	0.2087103787	-0.2651076465
C	-0.1251085409	-0.3298104205	0.2036361302
H	-1.6589692586	-1.4060027272	-0.8160794099
H	-1.3215988544	0.1940767222	-1.4786713135
C	1.1724784474	-0.0359969850	-0.4457282505
O	0.4902141017	0.9531907994	0.3227839521
H	-0.0961590521	-0.9070030080	1.1274301300
C	2.4889143617	-0.5256980467	0.1089320964
H	1.1517913796	0.1617538436	-1.5155520130

Energy of Optimized Geometry: -271.653982747337

OX6 2,3-epoxypentane BMK STRUC3

C	-2.1534923537	-0.4206882515	-0.6059514040
C	-1.5513614638	0.1824785154	0.6702903246
H	2.4204965936	-1.6621959411	0.5733655581
H	2.9529627796	-0.6249837279	-0.7579687041
H	2.6678311121	0.0761933786	0.8431727534
H	-1.8078400909	0.1250150089	-1.4853642639
H	-1.8772726507	-1.4703740382	-0.7189490981
H	-3.2422282507	-0.3648959910	-0.5750889584
C	-0.0420958893	0.3425914666	0.6479720881
H	-1.9810533851	1.1738508006	0.8449318671
H	-1.8133715434	-0.4275421230	1.5397286075
C	0.8773554547	-0.4054449553	-0.2422030515
O	0.5313036721	0.9532501456	-0.5057729248
H	0.3854264065	0.7106819675	1.5793842718
C	2.3175225477	-0.6698725918	0.1288911972
H	0.4362974012	-1.1037544685	-0.9484089983

Energy of Optimized Geometry: -271.653082076641

OX6 2,3-epoxypentane BMK STRUC4

C	-2.5047133966	-0.3150438998	0.1327481633
C	-1.4202219061	0.7236092073	-0.1958398854
H	1.6468905893	-2.3448214571	0.1745471026
H	2.9257347101	-1.2223080789	-0.3130471572
H	2.1347688237	-1.0248264790	1.2589860940
H	-2.2519906803	-1.2885955283	-0.2924475561
H	-2.6134955345	-0.4380833633	1.2127166956
H	-3.4718923986	-0.0098703122	-0.2683493601
C	-0.0795254483	0.3241354135	0.3789042849
H	-1.3145753683	0.8432109392	-1.2766128663
H	-1.7009278779	1.6998599648	0.2094607274
C	0.9425296376	-0.3995365532	-0.4136247041
O	1.0680579850	0.9935520289	-0.1341450949
H	-0.0547192842	0.1836226757	1.4591716174
C	1.9744593044	-1.3038743999	0.2169009862
H	0.6976922574	-0.6084996270	-1.4534157689

Energy of Optimized Geometry: -271.653636697486

OX6 2,3-epoxypentane BMK STRUC5

C	-2.1062532924	-0.4344817537	-0.7457634649
C	-1.4895529243	-0.4867268252	0.6585640974
H	2.6991711595	-1.2321688897	-0.3852233152
H	2.9288739734	0.4250499849	-0.9619384128
H	2.6893164437	0.1205918250	0.7662140416
H	-3.1819074718	-0.6069444998	-0.6944882959
H	-1.9377950195	0.5432048719	-1.2000136186
H	-1.6778181234	-1.1977162280	-1.3975552714
C	-0.0344347508	-0.0604730633	0.7311279605
H	-2.0538938266	0.1669887755	1.3309918754
H	-1.5662911262	-1.4972942505	1.0704260031
C	0.9038515574	-0.0482652242	-0.4166395391
O	0.3212506136	1.1560999880	0.0787078957
H	0.4153758352	-0.1600657277	1.7177976901
C	2.3964842117	-0.1933921387	-0.2363694496
H	0.5146124731	-0.3437036759	-1.3872434026

Energy of Optimized Geometry: -271.653082045337

OX6 2,3-epoxypentane BMK STRUC6

C	-2.1352700032	-0.3285951282	-0.7167058182
C	-1.5689033027	0.0840199862	0.6485992798
H	2.9740083615	-0.5071176854	-0.7620849266
H	2.6438832796	-0.0468919742	0.9158320948
H	2.4118510592	-1.7275747962	0.3893588958
H	-3.2246726093	-0.2794600337	-0.7063309506
H	-1.7701961344	0.3391677628	-1.4985821046
H	-1.8509403640	-1.3504339178	-0.9733924295
C	-0.0601516641	0.2455695965	0.6895479358
H	-2.0070143158	1.0396092535	0.9533135252
H	-1.8510768468	-0.6452300005	1.4136230141
C	0.8846900250	-0.3665377047	-0.2751478089
O	0.5406564680	1.0158539891	-0.3487505737
H	0.3416531810	0.4751957421	1.6752131320
C	2.3158621239	-0.6812239582	0.0915787527
H	0.4646402946	-0.9559265226	-1.0856965151

Energy of Optimized Geometry: -271.653081968384

8.2 CAM-B3LYP

OX6 2,3-epoxypentane CAM-B3LYP STRUC1

C	-2.3787291613	-0.8285530856	-0.0050693953
C	-1.5229001578	0.4216502059	-0.1610788154
H	2.2763465036	-0.7278705484	1.1987907569
H	2.0807574781	-1.9515330911	-0.0664909006
H	3.1035959500	-0.5423347843	-0.3497148230
H	-1.9404214503	-1.6754467746	-0.5334121254
H	-2.4808928709	-1.1095126954	1.0436674057
H	-3.3799873728	-0.6697681546	-0.4022002555
C	-0.1449504606	0.2381551466	0.4031000215
H	-1.4354737630	0.6993846946	-1.2121339008
H	-1.9976669627	1.2641337353	0.3462305273
C	1.0047292908	-0.1356895380	-0.4156015010
O	0.8362993115	1.2059058200	0.0401713951
H	-0.1089843905	-0.0386636733	1.4547204673
C	2.1836462567	-0.8829851102	0.1253457870
H	0.8285406862	-0.2485543045	-1.4823259017

Energy of Optimized Geometry: -271.701884853504

OX6 2,3-epoxypentane CAM-B3LYP STRUC2

C	-2.5783559933	0.2037209889	0.2411722806
C	-1.4126492306	-0.3538927016	-0.5648875937
H	2.3723169556	-0.7209803376	1.2126606026
H	2.7518509021	-1.4776449645	-0.3430932582
H	3.2577240710	0.1811520279	-0.0199144960
H	-2.7348203718	-0.3701168550	1.1553340984
H	-2.3884577466	1.2381117124	0.5242333665
H	-3.5038225222	0.1740022838	-0.3321065685
C	-0.1240158303	-0.3117815903	0.2010828628
H	-1.6141507411	-1.3893938934	-0.8502069890
H	-1.2889954160	0.2146523507	-1.4875726955
C	1.1682609554	-0.0415858935	-0.4193616961
O	0.4962439404	0.9685617978	0.3317313846
H	-0.1159189131	-0.8847715099	1.1259613293
C	2.4614517765	-0.5452163001	0.1421304337
H	1.1670053861	0.1505626156	-1.4890044698

Energy of Optimized Geometry: -271.702252712570

OX6 2,3-epoxypentane CAM-B3LYP STRUC3

C	-2.1443823210	-0.4363044069	-0.6075841232
C	-1.5390214947	0.1503940141	0.6581128579
H	2.4123014387	-1.6255511173	0.6062266974
H	2.9482150025	-0.6089090809	-0.7319636860
H	2.6544946748	0.1112376093	0.8532627555
H	-1.8378673132	0.1330314010	-1.4835738657
H	-1.8460506368	-1.4738231090	-0.7542683781
H	-3.2316826958	-0.4136275395	-0.5554121321
C	-0.0453413494	0.3353205710	0.6355483321
H	-1.9846643069	1.1280298115	0.8572549623
H	-1.7848331813	-0.4744413627	1.5201338356
C	0.8796978568	-0.3896213779	-0.2311322258
O	0.5235762595	0.9639123086	-0.5118898772
H	0.3668758384	0.7104239535	1.5692870505
C	2.3062573575	-0.6415296451	0.1482403180
H	0.4565829451	-1.0949383458	-0.9389057727

Energy of Optimized Geometry: -271.700829064531

OX6 2,3-epoxypentane CAM-B3LYP STRUC4

C	-2.4919634806	-0.3467916421	0.1082795147
C	-1.4178023948	0.6834763787	-0.2144301183
H	1.6657036368	-2.3088335124	0.1939634051
H	2.9310076635	-1.1734456108	-0.2773181072
H	2.1266041025	-0.9908757614	1.2835720094
H	-2.2383901758	-1.3240411964	-0.3031712762
H	-2.6173016486	-0.4635805212	1.1852975557
H	-3.4549382952	-0.0508196994	-0.3046805375
C	-0.0856900117	0.3136000119	0.3681358035
H	-1.3067957323	0.7987649015	-1.2933266986
H	-1.7094626559	1.6609526311	0.1757094336
C	0.9479940311	-0.3810794095	-0.3940659859
O	1.0528204016	1.0159615380	-0.1238554572
H	-0.0745337020	0.1769438701	1.4475215442
C	1.9762713991	-1.2645034163	0.2407124055
H	0.7235642801	-0.5982682761	-1.4353494271

Energy of Optimized Geometry: -271.701884819138

OX6 2,3-epoxypentane CAM-B3LYP STRUC5

C	-2.3739558814	0.9751269826	-0.4090187573
C	-1.5451070774	-0.2200521407	0.0422584188
H	2.2969104563	-1.3953445543	1.6297639902
H	3.1404947580	-0.8664427498	0.1736587161
H	2.4326216450	0.3314003305	1.2602596265
H	-3.4001149104	0.6832370789	-0.6277603441
H	-2.4046984462	1.7461284894	0.3617845038
H	-1.9492303041	1.4186107404	-1.3085496623
C	-0.1231908024	0.1553589126	0.3353174973
H	-1.9809740709	-0.6609328747	0.9423400777
H	-1.5502143359	-0.9926209321	-0.7278897245
C	1.0094353585	-0.7216000673	0.0596311875
O	0.7199898686	0.3844173798	-0.7945412832
H	0.0075880391	0.8960880165	1.1215122522
C	2.2933898160	-0.6573797935	0.8268885846
H	0.7851049764	-1.6822081078	-0.3964272152

Energy of Optimized Geometry: -271.702252470724

OX6 2,3-epoxypentane CAM-B3LYP STRUC6

C	-2.1479224484	-0.3563047292	-0.6443030711
C	-1.5371821605	0.0942017160	0.6735267176
H	2.9420293607	-0.5358612350	-0.7999963414
H	2.6536583123	-0.0126469508	0.8617037272
H	2.4023014122	-1.7053516587	0.4053158701
H	-3.2349569443	-0.3270759825	-0.5899012699
H	-1.8345981791	0.2944490027	-1.4591082799
H	-1.8607820808	-1.3771653613	-0.8940302132
C	-0.0417792042	0.2660669182	0.6684510798
H	-1.9731068267	1.0513662752	0.9700419029
H	-1.7895070829	-0.6115437807	1.4686376429
C	0.8754023273	-0.3689835478	-0.2739605945
O	0.5313683628	1.0100631142	-0.4054510364
H	0.3749949393	0.5338809066	1.6364539095
C	2.3007043380	-0.6722491735	0.0708255032
H	0.4447747103	-0.9899037272	-1.0527134832

Energy of Optimized Geometry: -271.700831040801

8.3 M06-2X

OX6 2,3-epoxypentane M06-2X STRUC1

C	-2.3571647642	-0.8272763091	-0.0282744069
C	-1.5228721006	0.4414133122	-0.1679656645
H	2.2538846837	-0.7069275178	1.2254809072
H	2.0457869036	-1.9710352362	-0.0001332743
H	3.0903614590	-0.5842460221	-0.3263875459
H	-1.8889672361	-1.6587675790	-0.5561680559
H	-2.4606627896	-1.1138872321	1.0190185257
H	-3.3571552133	-0.6880921119	-0.4356499649
C	-0.1492266577	0.2593418522	0.4110823740
H	-1.4194886337	0.7235750912	-1.2169449906
H	-2.0145460164	1.2745167176	0.3386196999
C	0.9956066788	-0.1498336660	-0.4069857633
O	0.8438167008	1.1973556629	0.0234190899
H	-0.1144758421	-0.0006159115	1.4673234645
C	2.1655077896	-0.8989035754	0.1571229832
H	0.8101813620	-0.2838232040	-1.4698538919

Energy of Optimized Geometry: -271.719684866055

OX6 2,3-epoxypentane M06-2X STRUC2

C	-2.5636201098	0.2144463732	0.2492953566
C	-1.4150088346	-0.3666806584	-0.5687237241
H	2.3635607480	-0.6986481594	1.2147349164
H	2.7718551494	-1.4507176314	-0.3382387819
H	3.2522386633	0.2166954125	-0.0082158001
H	-2.7160889257	-0.3569325238	1.1658664181
H	-2.3439345802	1.2446658330	0.5276825015
H	-3.4962021411	0.2025531675	-0.3128914670
C	-0.1244453494	-0.3277398425	0.1978487330
H	-1.6307626777	-1.4007373475	-0.8468170943
H	-1.2860803003	0.2001673154	-1.4923466052
C	1.1677751509	-0.0348841932	-0.4253672661
O	0.4801068163	0.9540436134	0.3309588394
H	-0.1072501113	-0.9072135490	1.1189672570
C	2.4660101524	-0.5225370584	0.1449099862
H	1.1607642095	0.1608920623	-1.4943136342

Energy of Optimized Geometry: -271.720022506402

OX6 2,3-epoxypentane M06-2X STRUC3

C	-2.1252089195	-0.3881687439	-0.6254730479
C	-1.5429956424	0.2128636613	0.6467451175
H	2.3539270098	-1.7070960283	0.5950905983
H	2.9227461298	-0.6831005221	-0.7268984260
H	2.6367690149	0.0218329585	0.8683516923
H	-1.7415017148	0.1314440196	-1.5032741082
H	-1.8771353095	-1.4454154810	-0.7162711816
H	-3.2104932061	-0.3020291590	-0.6280140837
C	-0.0413233885	0.3406056489	0.6462381324
H	-1.9545278278	1.2130542390	0.8034494155
H	-1.8335397758	-0.3800262526	1.5167027590
C	0.8578239918	-0.4156815424	-0.2307274041
O	0.5515344632	0.9481471871	-0.4944713173
H	0.3855283214	0.6907606989	1.5830279055
C	2.2772489598	-0.7144294348	0.1508868011
H	0.4049124466	-1.0993761104	-0.9418271736

Energy of Optimized Geometry: -271.719446945374

OX6 2,3-epoxypentane M06-2X STRUC4

C	-2.4603176583	-0.4221163777	0.0600677167
C	-1.4313167106	0.6665105756	-0.2247721467
H	1.6925865490	-2.2706972832	0.2400062774
H	2.9454153509	-1.1207658108	-0.2380634661
H	2.1208841538	-0.9255848196	1.3127194444
H	-2.1433211117	-1.3752267756	-0.3646451731
H	-2.5928493647	-0.5637561321	1.1334054221
H	-3.4302458567	-0.1683758506	-0.3643940932
C	-0.0974304785	0.3292281290	0.3773443973
H	-1.2989474109	0.8030247029	-1.2992663131
H	-1.7718420785	1.6227736227	0.1778380617
C	0.9491971102	-0.3612743912	-0.3810941291
O	1.0311595033	1.0340895310	-0.1185709059
H	-0.0883641155	0.1968057370	1.4574982753
C	1.9880312466	-1.2218590470	0.2731788432
H	0.7254711714	-0.5898728106	-1.4202309534

Energy of Optimized Geometry: -271.719684260463

OX6 2,3-epoxypentane M06-2X STRUC5

C	-2.0977860234	-0.3763014091	-0.7191399090
C	-1.4850629611	-0.4302769606	0.6737992089
H	2.6269640492	-1.3194190507	-0.4145150175
H	2.9037925900	0.3237546004	-1.0000725605
H	2.6789459905	0.0346651519	0.7288488324
H	-3.1788056146	-0.4941893930	-0.6669807896
H	-1.8832497470	0.5811069002	-1.1934788258
H	-1.7089865436	-1.1701828501	-1.3563584750
C	-0.0259288159	-0.0570114957	0.7290105349
H	-2.0209506750	0.2517886630	1.3384725897
H	-1.5968617841	-1.4289110707	1.1015304333
C	0.8822571631	-0.0770019401	-0.4218420581
O	0.3545851544	1.1468041915	0.0752832715
H	0.4362921971	-0.1731366860	1.7063420925
C	2.3611567967	-0.2725481095	-0.2665025242
H	0.4656604776	-0.3614418624	-1.3829270126

Energy of Optimized Geometry: -271.719447055143

OX6 2,3-epoxypentane M06-2X STRUC6

C	-2.1243278572	-0.4216166684	-0.6080911653
C	-1.5322205766	0.0863948580	0.6996115037
H	2.9278873793	-0.4847886303	-0.8542002825
H	2.6565615816	0.0575383624	0.8058367479
H	2.4389507535	-1.6497487421	0.3800126431
H	-3.2118633771	-0.3816249955	-0.5774625693
H	-1.7856173682	0.1906212464	-1.4437058897
H	-1.8367431704	-1.4549667500	-0.8006098173
C	-0.0376015251	0.2789013077	0.6753726847
H	-1.9804252318	1.0491871897	0.9573113573
H	-1.7738696066	-0.5950681313	1.5181459501
C	0.8678300841	-0.3558142702	-0.2873797894
O	0.4981666744	1.0111360347	-0.4193671393
H	0.3998331325	0.5622817705	1.6296687812
C	2.3084908754	-0.6249374310	0.0317235585
H	0.4247204554	-0.9926528880	-1.0465449954

Energy of Optimized Geometry: -271.719447047460

8.4 MN15

OX6 2,3-epoxypentane MN15 STRUC1

C	-2.3492160864	-0.8203258703	-0.0243073319
C	-1.5195556828	0.4506329019	-0.1573957032
H	2.2448778790	-0.7124337480	1.2125629775
H	2.0249318492	-1.9822609714	-0.0047670947
H	3.0797483818	-0.6065566968	-0.3414723358
H	-1.8790027510	-1.6471842977	-0.5580322480
H	-2.4477295780	-1.1143275622	1.0216171326
H	-3.3515204865	-0.6851953555	-0.4272211492
C	-0.1452883085	0.2633915747	0.4170788237
H	-1.4122339689	0.7383643186	-1.2050490844
H	-2.0125971847	1.2811241269	0.3519225081
C	0.9871100895	-0.1519700225	-0.4135174444
O	0.8491374062	1.1927502604	0.0217965512
H	-0.1053752143	-0.0040136534	1.4719158132
C	2.1530156720	-0.9100635093	0.1451414134
H	0.7882604177	-0.2861645468	-1.4744753714

Energy of Optimized Geometry: -271.507920713056

OX6 2,3-epoxypentane MN15 STRUC2

C	-2.5543312971	0.2210810415	0.2437745544
C	-1.4109268646	-0.3705623452	-0.5713888049
H	2.3509704753	-0.6890553816	1.2182508631
H	2.7705749050	-1.4480210725	-0.3280842428
H	3.2500148071	0.2198760961	-0.0021108058
H	-2.7133609980	-0.3488912495	1.1603282756
H	-2.3222058738	1.2482700703	0.5235395621
H	-3.4875918950	0.2212438418	-0.3173515352
C	-0.1234095735	-0.3340988715	0.1993349281
H	-1.6331243936	-1.4027037192	-0.8517583021
H	-1.2710565953	0.1960510827	-1.4942850576
C	1.1647795069	-0.0317920013	-0.4255930536
O	0.4731084560	0.9486891407	0.3339782803
H	-0.1033366808	-0.9199079177	1.1169938685
C	2.4616079870	-0.5173182814	0.1481541784
H	1.1548333611	0.1628215055	-1.4951832232

Energy of Optimized Geometry: -271.508311779539

OX6 2,3-epoxypentane MN15 STRUC3

C	-2.1186246225	-0.3832251464	-0.6152835911
C	-1.5370376514	0.2141690132	0.6576470414
H	2.3513256474	-1.7022778025	0.5959934825
H	2.9160540643	-0.6940172302	-0.7386980474
H	2.6301486814	0.0301103233	0.8481305268
H	-1.7218521783	0.1303621619	-1.4909610164
H	-1.8810554045	-1.4432945770	-0.7032343825
H	-3.2028812692	-0.2862939829	-0.6278344795
C	-0.0359097944	0.3417131122	0.6472132197
H	-1.9490201849	1.2134129821	0.8190823028
H	-1.8250203858	-0.3804769409	1.5274265930
C	0.8509723608	-0.4179935340	-0.2376092331
O	0.5464069192	0.9444725373	-0.4985713817
H	0.4007878606	0.6879130815	1.5815437385
C	2.2711632366	-0.7154930807	0.1395108811
H	0.3898213497	-1.1044687013	-0.9413900884

Energy of Optimized Geometry: -271.508311779539

OX6 2,3-epoxypentane MN15 STRUC4

C	-2.4751218463	-0.2449531320	0.0858611579
C	-1.3777883676	0.7811327267	-0.1673775824
H	1.5071663762	-2.3965282904	0.1229227081
H	2.8397950795	-1.3367223131	-0.3451036328
H	2.0611366221	-1.1251992670	1.2271516380
H	-2.2305037254	-1.1977956879	-0.3851908640
H	-2.6009198491	-0.4261397425	1.1542558210
H	-3.4325647230	0.0905272557	-0.3090806638
C	-0.0647265764	0.3210210955	0.3962646355
H	-1.2449543053	0.9542283647	-1.2370782561
H	-1.6448146896	1.7409336606	0.2794735578
C	0.9090735964	-0.4105831560	-0.4168229091
O	1.1032615177	0.9581842285	-0.0921816619
H	-0.0496721208	0.1333941443	1.4688453795
C	1.8865249802	-1.3762256889	0.1815363290
H	0.6456872209	-0.5750921274	-1.4593418444

Energy of Optimized Geometry: -271.507920684318

OX6 2,3-epoxypentane MN15 STRUC5

C	-2.0806814619	-0.4772877733	-0.6773089944
C	-1.4315243845	-0.5534527597	0.6968564164
H	2.6898278403	-1.1300100881	-0.5288581742
H	2.8458991325	0.5459359363	-1.0618897900
H	2.6776114779	0.1849028795	0.6602081539
H	-3.1529141143	-0.6524929284	-0.6085813709
H	-1.9251138879	0.5082656441	-1.1159586485
H	-1.6674144274	-1.2232668763	-1.3559650614
C	0.0007307615	-0.0868889212	0.7276366685
H	-1.9913565304	0.0624503576	1.4050104216
H	-1.4684207515	-1.5745637865	1.0828197489
C	0.8674410695	-0.0037680185	-0.4508023157
O	0.2800798241	1.1606724370	0.1109271333
H	0.5028255398	-0.2133938829	1.6844262653
C	2.3588207666	-0.1086721518	-0.3394064776
H	0.4399709557	-0.2823128222	-1.4094178819

Energy of Optimized Geometry: -271.508172601521

OX6 2,3-epoxypentane MN15 STRUC6

C	-2.1156385749	-0.4293197509	-0.5938444037
C	-1.5330432981	0.1612182432	0.6818358541
H	2.9227793732	-0.5997582570	-0.7903238288
H	2.6397493084	0.0844704332	0.8146846394
H	2.4044651897	-1.6496208007	0.5311461525
H	-3.2022173675	-0.3617682895	-0.5903866280
H	-1.7448038215	0.1102196972	-1.4653273124
H	-1.8506933342	-1.4808507776	-0.7039306192
C	-0.0361137485	0.3294180937	0.6538272717
H	-1.9695396196	1.1459064711	0.8666292995
H	-1.7930804122	-0.4562899054	1.5444753079
C	0.8582879452	-0.3906901602	-0.2561871365
O	0.5140383068	0.9673161726	-0.4886184334
H	0.4038917398	0.6708153574	1.5883836048
C	2.2911323493	-0.6564416922	0.0959717500
H	0.4059102863	-1.0772000607	-0.9656727776

Energy of Optimized Geometry: -271.508172800899

8.5 ωB97X-D

OX6 2,3-epoxypentane ωB97X-D STRUC1

C	-2.3691673405	-0.8110696155	-0.0020328610
C	-1.5225041318	0.4488413493	-0.1478796528
H	2.2703128117	-0.7530553946	1.1921605138
H	2.0232896204	-1.9883030750	-0.0543027426
H	3.0840058846	-0.6121779611	-0.3704534267
H	-1.9243662833	-1.6463886263	-0.5446503832
H	-2.4607241939	-1.1055695712	1.0446962416
H	-3.3743801358	-0.6543683256	-0.3912849393
C	-0.1400649301	0.2573900448	0.4094848798
H	-1.4402579978	0.7364050753	-1.1974390883
H	-1.9996309022	1.2829855454	0.3714098881
C	0.9936188995	-0.1485940130	-0.4173887917
O	0.8546439560	1.1972001274	0.0253113097
H	-0.1001299153	-0.0107394933	1.4642863064
C	2.1609213163	-0.9204695232	0.1213067550
H	0.8026860458	-0.2717758627	-1.4814168272

Energy of Optimized Geometry: -271.759866882259

OX6 2,3-epoxypentane ωB97X-D STRUC2

C	-2.5810808685	0.2004766660	0.2485579497
C	-1.4151566958	-0.3575960462	-0.5606915728
H	2.3799035016	-0.7185394247	1.2066989966
H	2.7473407672	-1.4786869922	-0.3517801019
H	3.2622769775	0.1801096711	-0.0333330475
H	-2.7254701191	-0.3663864272	1.1698256235
H	-2.3955704531	1.2396084428	0.5195705440
H	-3.5099340048	0.1573470195	-0.3193488653
C	-0.1226691779	-0.3082946867	0.2038516835
H	-1.6131020786	-1.3959451561	-0.8389261414
H	-1.2973185084	0.2074231684	-1.4871517627
C	1.1677416532	-0.0374929807	-0.4225539419
O	0.4973674062	0.9685688594	0.3285751086
H	-0.1115973715	-0.8816262516	1.1297482803
C	2.4646709381	-0.5441295892	0.1347312501
H	1.1624921327	0.1516911378	-1.4936789522

Energy of Optimized Geometry: -271.760002788044

OX6 2,3-epoxypentane ωB97X-D STRUC3

C	-2.1536724191	-0.4501245989	-0.5609164159
C	-1.5277093729	0.1548896455	0.6883625400
H	2.4141212250	-1.6408128312	0.5328169706
H	2.9331514259	-0.6006987722	-0.7962143467
H	2.6714174476	0.0906093520	0.8092941072
H	-1.8555869553	0.1047822108	-1.4502005871
H	-1.8589416358	-1.4914493102	-0.6931199833
H	-3.2405060551	-0.4220256756	-0.4941259306
C	-0.0319506960	0.3387639531	0.6372673970
H	-1.9695909838	1.1359206727	0.8812953372
H	-1.7577718905	-0.4600245084	1.5620676236
C	0.8727425866	-0.3828444116	-0.2550775792
O	0.5156028905	0.9708924707	-0.5143833482
H	0.4000615219	0.7041776262	1.5669724158
C	2.3071906768	-0.6472188413	0.0953918933
H	0.4317470515	-1.0827524891	-0.9591113762

Energy of Optimized Geometry: -271.759166650018

OX6 2,3-epoxypentane ωB97X-D STRUC4

C	-2.4841663186	-0.3126213470	0.1094880377
C	-1.4069671880	0.7224756780	-0.1957856131
H	1.5946847137	-2.3457147821	0.1653981274
H	2.8943587678	-1.2465694746	-0.3056431129
H	2.1059618253	-1.0525960532	1.2642151760
H	-2.2337439584	-1.2790204465	-0.3300240440
H	-2.5993855482	-0.4563157899	1.1851039083
H	-3.4495351722	-0.0021118416	-0.2885017937
C	-0.0753181232	0.3237113186	0.3751591541
H	-1.2995447359	0.8585070004	-1.2733678881
H	-1.6913347155	1.6928146552	0.2176683520
C	0.9336332638	-0.3918108237	-0.4021020392
O	1.0740726101	0.9963230156	-0.1202246173
H	-0.0610534208	0.1758196208	1.4542343383
C	1.9409527997	-1.3127038815	0.2192762212
H	0.6939367303	-0.5962580934	-1.4435807790

Energy of Optimized Geometry: -271.759865483394

OX6 2,3-epoxypentane ωB97X-D STRUC5

C	-2.1488208981	-0.4864295025	-0.5485956607
C	-1.3974566183	-0.5273314841	0.7750247701
H	2.6473595703	-1.1780600558	-0.6402309585
H	2.8080316529	0.4830978507	-1.2168224370
H	2.7395896710	0.1587393659	0.5194282308
H	-3.2042442555	-0.7071715234	-0.3941538055
H	-2.0734172662	0.4999651838	-1.0058029734
H	-1.7587473355	-1.2205468492	-1.2540792521
C	0.0356435848	-0.0621602066	0.7187674356
H	-1.9137359193	0.0969898970	1.5089174994
H	-1.4022090550	-1.5419454790	1.1812362513
C	0.8568170456	-0.0160217885	-0.4891185452
O	0.3026248655	1.1729766769	0.0640145897
H	0.5712381947	-0.1671373121	1.6603785983
C	2.3508747122	-0.1452479784	-0.4518235911
H	0.3903390823	-0.3089013367	-1.4254856597

Energy of Optimized Geometry: -271.759166664933

OX6 2,3-epoxypentane ωB97X-D STRUC6

C	-2.1623898811	-0.3499825730	-0.5963071055
C	-1.5197029831	0.0673307155	0.7192912412
H	2.9206557548	-0.5030515309	-0.9010021017
H	2.6787843575	-0.0443471559	0.7887209803
H	2.4050752157	-1.7172415880	0.2725388065
H	-3.2483099621	-0.3274165141	-0.5138304755
H	-1.8706991802	0.3250608636	-1.4005565798
H	-1.8750222611	-1.3628973772	-0.8795221298
C	-0.0231299711	0.2472633093	0.6784039310
H	-1.9526235889	1.0132376551	1.0550510740
H	-1.7453076733	-0.6646642662	1.4989176665
C	0.8669008126	-0.3500375501	-0.3144914091
O	0.5190007655	1.0287442312	-0.3802317253
H	0.4202766014	0.4771719188	1.6453766864
C	2.3022156334	-0.6710877071	-0.0188745014
H	0.4135788935	-0.9430406121	-1.1038001529

Energy of Optimized Geometry: -271.759166289919

8.6 EOM-CCSD

OX6 2,3-epoxypentane EOM-CCSD STRUC1

C	-2.3577188621	-0.8658967247	-0.0462511619
C	-1.5316443788	0.4160301585	-0.1839405716
H	2.2559600371	-0.6863606163	1.2477772023
H	2.0866934121	-1.9458801936	0.0048710815
H	3.1111605780	-0.5328756470	-0.2962492076
H	-1.8794441497	-1.6964561267	-0.5718114599
H	-2.4662641384	-1.1518536749	1.0034106637
H	-3.3586347485	-0.7342853998	-0.4612356188
C	-0.1575776526	0.2494820059	0.4095033240
H	-1.4231016417	0.6960522794	-1.2353718552
H	-2.0373846060	1.2463063388	0.3186789717
C	1.0045591824	-0.1375539735	-0.4011964906
O	0.8325471082	1.2266621590	0.0350595864
H	-0.1278354630	-0.0092255300	1.4676164838
C	2.1849562436	-0.8703486554	0.1744061652
H	0.8349420992	-0.2665714967	-1.4687861773

Energy of Optimized Geometry: -271.285171995412

OX6 2,3-epoxypentane EOM-CCSD STRUC2

C	-2.5683376597	0.2424278799	0.2813669071
C	-1.4294648938	-0.3489666273	-0.5533108042
H	2.3745671596	-0.7410305550	1.1853007183
H	2.7546210205	-1.4948193881	-0.3792460491
H	3.2620009656	0.1693585384	-0.0486657810
H	-2.7156498458	-0.3304789075	1.2009694236
H	-2.3393348560	1.2736348973	0.5580213929
H	-3.5087786744	0.2360644886	-0.2731584668
C	-0.1260057787	-0.3283636299	0.2000093299
H	-1.6598234555	-1.3832601131	-0.8311095158
H	-1.3047458035	0.2200528725	-1.4789627301
C	1.1648186736	-0.0544840154	-0.4433513688
O	0.5012504843	0.9658636520	0.3307113745
H	-0.1050340253	-0.9050484486	1.1244182516
C	2.4668083102	-0.5619769328	0.1127308222
H	1.1472875766	0.1449725496	-1.5130419726

Energy of Optimized Geometry: -271.285539206821

OX6 2,3-epoxypentane EOM-CCSD STRUC3

C	-2.1431188190	-0.3945518183	-0.6088748046
C	-1.5414879711	0.1946111627	0.6669454019
H	2.3832688253	-1.6937421931	0.5834668888
H	2.9329917557	-0.6619029444	-0.7465205388
H	2.6502512150	0.0426615468	0.8544874541
H	-1.7989588593	0.1570710902	-1.4859252140
H	-1.8688619449	-1.4447434507	-0.7321693063
H	-3.2327176123	-0.3388048044	-0.5762057080
C	-0.0371037345	0.3368235414	0.6483875453
H	-1.9626014081	1.1895548801	0.8470630823
H	-1.8119062385	-0.4181497270	1.5336932388
C	0.8633717534	-0.4176885744	-0.2349057635
O	0.5435888899	0.9607463484	-0.5150846518
H	0.3962108251	0.6995827444	1.5793195727
C	2.2932895698	-0.6993476705	0.1381923055
H	0.4131173259	-1.1087507571	-0.9425677588

Energy of Optimized Geometry: -271.284529723774

OX6 2,3-epoxypentane EOM-CCSD STRUC4

C	-2.4787428344	-0.3847124112	0.1360669217
C	-1.4315582426	0.6819165267	-0.1961978596
H	1.6739147820	-2.3011102015	0.2099702810
H	2.9318267576	-1.1665901758	-0.3070405285
H	2.1464300250	-0.9519092864	1.2664903935
H	-2.1966598076	-1.3529428762	-0.2855391724
H	-2.5837450516	-0.5080461668	1.2173199849
H	-3.4567813475	-0.1129110456	-0.2650938673
C	-0.0849664353	0.3255793732	0.3762446915
H	-1.3277139142	0.7991942082	-1.2784017089
H	-1.7418763882	1.6523693675	0.2034069343
C	0.9369319623	-0.3820350976	-0.4064426911
O	1.0521951883	1.0335705171	-0.1538744322
H	-0.0488454247	0.2027222485	1.4583815830
C	1.9846005096	-1.2531441770	0.2301100102
H	0.6891283492	-0.6098670397	-1.4417442823

Energy of Optimized Geometry: -271.285171994946

OX6 2,3-epoxypentane EOM-CCSD STRUC5

C	-2.1306732329	-0.5092497228	-0.5659161394
C	-1.4084129489	-0.5145164459	0.7813390206
H	2.6815618851	-1.1745824579	-0.5646346780
H	2.8185234906	0.4743821459	-1.1954585522
H	2.7215399459	0.2067753451	0.5534219806
H	-3.1921155640	-0.7236889209	-0.4289718415
H	-2.0385362900	0.4664187765	-1.0470915624
H	-1.7242336594	-1.2664142189	-1.2403221066
C	0.0280599186	-0.0480534876	0.7294499501
H	-1.9388098352	0.1351587627	1.4858152331
H	-1.4213436464	-1.5196953780	1.2164380226
C	0.8613615110	-0.0396828072	-0.4812776515
O	0.2877421037	1.1832989201	0.0248007118
H	0.5597521729	-0.1088666379	1.6779199369
C	2.3610171848	-0.1399224942	-0.4165815949
H	0.4038626397	-0.3687973966	-1.4105044295

Energy of Optimized Geometry: -271.284529723401

OX6 2,3-epoxypentane EOM-CCSD STRUC6

C	-2.1251455577	-0.3923872966	-0.6701513428
C	-1.5486337447	0.0418355063	0.6773903642
H	2.9576957590	-0.4391659286	-0.8106132729
H	2.6456786851	0.0306615542	0.8691201126
H	2.4481024544	-1.6622219825	0.3642090396
H	-3.2161286699	-0.3844993488	-0.6371047049
H	-1.8024290200	0.2860116008	-1.4623491405
H	-1.8091514737	-1.4046022655	-0.9326133756
C	-0.0510874911	0.2442641919	0.6877241785
H	-2.0091887762	0.9859028329	0.9877350065
H	-1.7952482605	-0.6927746425	1.4515893636
C	0.8795203826	-0.3477868185	-0.2837576331
O	0.5054406540	1.0422393197	-0.3770386595
H	0.3665861274	0.4944577622	1.6618592904
C	2.3191442692	-0.6210222292	0.0568635506
H	0.4577719606	-0.9538723377	-1.0811739752

Energy of Optimized Geometry: -271.284529724560

9 2-propyloxirane (OX7)

9.1 BMK

OX7 2-propyloxirane BMK STRUC1

C	-1.4233036127	-0.9841689354	0.0735850157
C	-0.7370625586	0.2243851455	-0.4400519434
H	3.1313499162	-1.1518983159	-0.2689935255
H	2.0334143419	-1.1940172821	1.1142241841
H	1.4364439086	-1.5860540842	-0.5004688526
H	2.4997787282	1.1725905898	0.3498344345
O	-2.0462504982	0.2929682521	0.1088661033
H	-1.8653997620	-1.6942403037	-0.6178725543
C	0.3601391961	0.9243967611	0.3321538664
H	1.8495003849	0.7881518662	-1.2304288346
H	-1.1318065523	-1.3801496382	1.0419648668
C	1.7676795797	0.5470606193	-0.1659333965
H	0.2124088886	2.0037744888	0.2336256721
H	0.2532920181	0.6798414506	1.3931884194
C	2.1125641662	-0.9340680050	0.0555708803
H	-0.7099048883	0.3603946606	-1.5202305551

Energy of Optimized Geometry: -271.645822033251

OX7 2-propyloxirane BMK STRUC2

C	-1.8366306707	-0.8468211253	-0.2682054386
C	-0.9041245210	0.2939440263	-0.4268297525
H	3.1158670839	0.9732253806	0.5992430084
H	2.8206132765	-0.6075878710	1.3273251255
H	3.7281181625	-0.4873720637	-0.1835706469
H	1.6755032334	0.3321465254	-1.3670775585
O	-2.1196260152	0.4276783637	0.2964451528
H	-2.4819181674	-1.1405029725	-1.0894880712
C	0.3809777390	0.3872547675	0.3642311810
H	1.3793047410	-1.2398706624	-0.6464706240
H	-1.5966017731	-1.6240489886	0.4514066962
C	1.5770851095	-0.1901777685	-0.4106274892
H	0.5730840675	1.4368776732	0.6096960816
H	0.2490770591	-0.1447213466	1.3108915767
C	2.8894906007	-0.0722323339	0.3775990027
H	-0.9083305727	0.8107971975	-1.3846640811

Energy of Optimized Geometry: -271.646442985905

OX7 2-propyloxirane BMK STRUC3

C	-2.2094745011	-0.5101649671	-0.1843199539
C	-0.9259290021	0.1133541869	-0.5787751443
H	3.6021673312	0.5891075727	0.6003803608
H	2.7614412853	-0.8420067350	1.2033918928
H	3.2026447945	-0.7313366051	-0.5028417059
H	1.1551507625	1.0813447892	0.9119982293
O	-1.6540685924	0.5695245146	0.5565237300
H	-3.1169824712	-0.2753682666	-0.7308753510
C	0.4092303275	-0.5366009573	-0.2979901807
H	1.6008075036	1.2000308556	-0.7826535090
H	-2.1933447165	-1.4705002631	0.3220123687
C	1.4916468692	0.4991025590	0.0505662179
H	0.2899948618	-1.2405909308	0.5309084161
H	0.7165506987	-1.1129661836	-1.1772605461
C	2.8452073891	-0.1577466245	0.3558449824
H	-0.9452648774	0.8130254438	-1.4124035136

Energy of Optimized Geometry: -271.646846943549

OX7 2-propyloxirane BMK STRUC4

C	-2.1128263772	0.0711398623	-0.2425728699
C	-0.8005698192	0.3928981299	0.3628870525
H	1.9586460054	-1.2991179380	-0.9062995582
H	3.0026425827	-1.1223489820	0.5103163568
H	1.2758334946	-1.4571839329	0.7079510732
H	1.7891083257	0.9759991323	1.1567304737
O	-1.1840292775	-0.9523717366	0.0902800779
H	-3.0109671196	0.0946957429	0.3658237960
C	0.3429400206	0.9621905278	-0.4475244452
H	2.4926488149	1.1256082463	-0.4417120921
H	-2.2543484200	0.2337483153	-1.3068323419
C	1.7240091250	0.6000240730	0.1306250807
H	0.2563186160	0.5927003710	-1.4738490291
H	0.2324209083	2.0506673158	-0.4782584852
C	2.0084864572	-0.9099994063	0.1132316412
H	-0.7871034813	0.6290268449	1.4257957878

Energy of Optimized Geometry: -271.646073309720

OX7 2-propyloxirane BMK STRUC5

C	-1.6796122067	-0.7282788675	0.5471626557
C	-0.6922006723	-0.0883986283	-0.3543102058
H	3.3453439043	-0.9529147620	-0.3068783296
H	1.7992227022	-1.3094735329	-1.0715831009
H	2.6064136133	0.2284323346	-1.3920537952
H	1.4451120898	-0.6331758635	1.3238376276
O	-2.0387829276	0.3647301254	-0.2885521745
H	-2.1256150449	-1.6801783810	0.2785449518
C	0.3660755333	0.8518536585	0.1813732260
H	2.3356613201	0.8529432661	1.0492854567
H	-1.6440853323	-0.5013045159	1.6087490248
C	1.6697222476	0.1298208733	0.5725174726
H	0.5807812975	1.6137864834	-0.5752181795
H	-0.0534049622	1.3681665442	1.0485209072
C	2.3959082277	-0.5152063312	-0.6193618309
H	-0.4592596213	-0.6049312601	-1.2817454197

Energy of Optimized Geometry: -271.645305144894

OX7 2-propyloxirane BMK STRUC6

C	-2.1255896610	-0.2155132482	0.4604072223
C	-0.6753528144	-0.4490963783	0.2747068825
H	3.2259829771	-0.3717902061	-1.0628427494
H	2.7836593965	-0.8994538515	0.5635663728
H	1.7997027101	-1.3759120268	-0.8250437952
H	2.1706058249	1.5205437215	0.1426185013
O	-1.4969797035	0.0085878652	-0.7956777988
H	-2.8109564911	-1.0563170574	0.4888880055
C	0.3651403581	0.5292098934	0.7732493999
H	1.1285611454	1.0239405675	-1.1802360472
H	-2.4514287303	0.6819981962	0.9772564374
C	1.5339402080	0.7079297212	-0.2155697526
H	-0.1229259245	1.4925475475	0.9412857269
H	0.7454353694	0.1781244624	1.7386541870
C	2.3835604737	-0.5598577323	-0.3953414939
H	-0.3528130181	-1.4808633849	0.1580271450

Energy of Optimized Geometry: -271.645905183774

OX7 2-propyloxirane BMK STRUC7

C	-1.7528242853	-0.7269602315	-0.4191475744
C	-1.0627108083	0.5847515369	-0.3914747715
H	2.8820927932	0.6335889561	0.9982099408
H	3.3007014752	-1.0244244456	0.5570357038
H	3.1651874478	0.2151190630	-0.6937502146
H	1.1303269936	-1.2782039167	-0.6479274577
O	-1.6631066015	0.0397881592	0.7764283442
H	-2.7478723661	-0.7981611673	-0.8461367185
C	0.4448123079	0.7562189737	-0.3845483779
H	0.8335594763	-0.8259227485	1.0261982354
H	-1.1699776823	-1.6413361669	-0.4195639450
C	1.2449343206	-0.4695934469	0.0786110901
H	0.7583883833	1.0474801132	-1.3925025154
H	0.6754209611	1.6050927792	0.2690031474
C	2.7365854730	-0.1441004449	0.2450787798
H	-1.6126028488	1.4315334054	-0.7964742119

Energy of Optimized Geometry: -271.645778311250

OX7 2-propyloxirane BMK STRUC8

C	-1.7170625406	-0.2907438430	-0.6754201501
C	-0.9974527589	0.6233144877	0.2411884120
H	2.7303126089	-1.4541210033	0.0295025602
H	2.5361989266	-0.1732221257	1.2288128744
H	1.2298912486	-1.3379392343	0.9621297775
H	2.2161300828	0.7254579985	-1.0840071753
O	-1.1496388502	-0.7591884006	0.5419376041
H	-2.8019043177	-0.2754546507	-0.7024023847
C	0.3958026540	1.1701782944	-0.0314984117
H	0.9458281640	-0.4373462771	-1.3870391175
H	-1.2278566394	-0.6456995408	-1.5764576049
C	1.4125199328	0.1631361042	-0.6024840745
H	0.2750502687	2.0157620729	-0.7160355264
H	0.7932327824	1.5830816162	0.9013022443
C	2.0120825481	-0.7602325140	0.4703343877
H	-1.6189211149	1.2649024784	0.8621040909

Energy of Optimized Geometry: -271.643597543496

OX7 2-propyloxirane BMK STRUC10

C	-1.5387426720	-0.4966059361	-0.7565655450
C	-1.0114907098	0.6729120296	-0.0139913570
H	1.0088660998	-1.4355367238	0.9084746242
H	2.6798424683	-1.4844882203	0.3154819712
H	2.2000784064	-0.2897679203	1.5233877406
H	2.4041039791	0.7343916794	-0.7566890684
O	-1.2730618664	-0.5625118591	0.6398548118
H	-2.5892955372	-0.5275003379	-1.0270829391
C	0.4171912544	1.1812220568	-0.0992400294
H	1.2118226400	-0.3729744150	-1.3888741737
H	-0.8743844941	-1.0880733099	-1.3768720448
C	1.5095523064	0.1673077206	-0.4865273577
H	0.4081331736	2.0014002505	-0.8245908628
H	0.6717280600	1.6295896071	0.8676321878
C	1.8680871320	-0.8264488728	0.6310804279
H	-1.7356860997	1.4448564568	0.2395267804

Energy of Optimized Geometry: -271.643456714860

OX7 2-propyloxirane BMK STRUC11

C	-1.3162600640	-0.9734204040	-0.1074104068
C	-0.9457570421	0.3826657552	-0.5815939588
H	2.8020478302	-0.4787773203	-0.9015395868
H	2.7751871398	-1.3752136554	0.6195812515
H	1.4746319253	-1.5839065363	-0.5566488131
H	0.8149283642	-0.0462942872	1.4136344897
O	-1.7039136421	0.1921113677	0.6068111502
H	-2.1264222468	-1.5060180030	-0.5953914116
C	0.4243522451	1.0197644005	-0.4242963467
H	2.1011882006	1.0326568884	0.9288728187
H	-0.5793880763	-1.5910467300	0.3937080599
C	1.3816972587	0.3052353304	0.5472513170
H	0.8848264421	1.0892408185	-1.4152241165
H	0.2506955537	2.0468842633	-0.0890157249
C	2.1509818904	-0.8536669153	-0.1081676399
H	-1.5375938788	0.7854630548	-1.4007235386

Energy of Optimized Geometry: -271.644049789091

9.2 CAM-B3LYP

OX7 2-propyloxirane CAM-B3LYP STRUC1

C	-1.4539642049	-0.9363280462	0.0593086575
C	-0.7273859745	0.2259116526	-0.4416989540
H	3.1268491312	-1.1941959635	-0.2373041386
H	2.0362642091	-1.2229463651	1.1440294185
H	1.4386207535	-1.6324803833	-0.4596063054
H	2.5031664783	1.1225897491	0.3592480922
O	-2.0427179278	0.3578307827	0.0849617028
H	-1.9074768553	-1.6355714520	-0.6336614559
C	0.3762992072	0.8926927478	0.3272348725
H	1.8655981070	0.7263480274	-1.2144165198
H	-1.1945390904	-1.3415832086	1.0308775053
C	1.7707112619	0.4951818906	-0.1504615121
H	0.2520051458	1.9730948388	0.2273267237
H	0.2566431121	0.6597968556	1.3873702877
C	2.1107481026	-0.9704030196	0.0853773629
H	-0.6811541194	0.3539569564	-1.5205954874

Energy of Optimized Geometry: -271.693133428749

OX7 2-propyloxirane CAM-B3LYP STRUC2

C	-1.8573382707	-0.7878855603	-0.1991447607
C	-0.8993549619	0.2917118875	-0.4135027611
H	3.1398152357	0.8994579866	0.5003495779
H	2.8267593770	-0.6477183802	1.2775424203
H	3.6933955762	-0.5921094870	-0.2541006018
H	1.6479887140	0.2371051007	-1.4073328477
O	-2.0913929366	0.5117802061	0.3309094265
H	-2.5280351254	-1.0882843701	-0.9955079522
C	0.3973384172	0.3715734024	0.3357515647
H	1.3338543980	-1.2991080327	-0.6372742982
H	-1.6359734626	-1.5506210361	0.5392744174
C	1.5572455256	-0.2495505009	-0.4332590973
H	0.6174793501	1.4211357146	0.5474301958
H	0.2745458243	-0.1229056442	1.3014091324
C	2.8788226494	-0.1425805396	0.3124265263
H	-0.9049764964	0.7733560822	-1.3878773294

Energy of Optimized Geometry: -271.694166227347

OX7 2-propyloxirane CAM-B3LYP STRUC3

C	-2.1889472593	-0.5005313642	-0.2099610783
C	-0.9176705821	0.1155827723	-0.5707859017
H	3.5905558954	0.5746646517	0.5945422574
H	2.7511067403	-0.8436947983	1.2118784416
H	3.1880509020	-0.7520768511	-0.4906994564
H	1.1648021079	1.0804237885	0.9065053065
O	-1.6607270519	0.5770855747	0.5546935590
H	-3.0845387449	-0.2691319938	-0.7745210791
C	0.4039579425	-0.5298854152	-0.2819930166
H	1.6040443062	1.1792554265	-0.7829782817
H	-2.1872112954	-1.4622644516	0.2905886585
C	1.4910058395	0.4842932716	0.0527856899
H	0.2791385118	-1.2275341158	0.5488104042
H	0.7041419310	-1.1199120275	-1.1528627910
C	2.8300072085	-0.1686924399	0.3588164180
H	-0.9226005789	0.8164702820	-1.4016654829

Energy of Optimized Geometry: -271.694637208913

OX7 2-propyloxirane CAM-B3LYP STRUC4

C	-2.1032966660	0.0950751729	-0.2310686472
C	-0.8035343909	0.3874704096	0.3621221915
H	2.0128386548	-1.2704688921	-0.9384123058
H	3.0288130913	-1.0918161841	0.4902136180
H	1.3169666469	-1.4811129898	0.6580771764
H	1.7722126389	0.9486648010	1.1611377929
O	-1.2040217442	-0.9561096389	0.0983742705
H	-3.0000977140	0.1420407586	0.3753915608
C	0.3393420464	0.9410603698	-0.4374973213
H	2.4707848116	1.1442406991	-0.4256543615
H	-2.2480295969	0.2587959731	-1.2931380780
C	1.7123833867	0.5900178257	0.1301920075
H	0.2523442371	0.5775179015	-1.4636898595
H	0.2290987658	2.0280549986	-0.4724358395
C	2.0358936965	-0.8972585474	0.0859218999
H	-0.7856740110	0.6258671859	1.4227815183

Energy of Optimized Geometry: -271.693462604113

OX7 2-propyloxirane CAM-B3LYP STRUC5

C	-1.7071576427	-0.6732214196	0.5335569670
C	-0.6930858002	-0.0936742247	-0.3418553314
H	3.3411330683	-0.9923832208	-0.2579357985
H	1.8176643951	-1.3350994755	-1.0595180536
H	2.6389623661	0.1937739812	-1.3524990132
H	1.4297171384	-0.6683518853	1.3290597396
O	-2.0236833650	0.4131568133	-0.3284674605
H	-2.1748767903	-1.6165072708	0.2770934920
C	0.3775483882	0.8178376024	0.1842383526
H	2.3224954133	0.8090862478	1.0711251472
H	-1.6926098499	-0.4303500396	1.5904242396
C	1.6621207555	0.0926126822	0.5804584431
H	0.6018909452	1.5714223164	-0.5752530644
H	-0.0306839006	1.3516624426	1.0435315237
C	2.4030909283	-0.5469121512	-0.5870697478
H	-0.4562648201	-0.6376427610	-1.2505446144

Energy of Optimized Geometry: -271.692712558193

OX7 2-propyloxirane CAM-B3LYP STRUC6

C	-2.1069186159	-0.2207187791	0.4666086904
C	-0.6806911980	-0.4392990590	0.2553200007
H	3.2423914265	-0.3696658967	-1.0183988256
H	2.7830658886	-0.8927416857	0.5981472273
H	1.8281396791	-1.3838735579	-0.7978695529
H	2.1625782750	1.5088464772	0.1582048024
O	-1.5157124312	0.0230242550	-0.8044639254
H	-2.7862451392	-1.0642377682	0.5044101273
C	0.3581141912	0.5236031626	0.7504129480
H	1.1567260781	1.0063643294	-1.1788319517
H	-2.4326718250	0.6660894803	0.9985314300
C	1.5375094813	0.6918158327	-0.2056778384
H	-0.1220894046	1.4899464182	0.9111184048
H	0.7178987467	0.1777068188	1.7238787587
C	2.3920992356	-0.5585158554	-0.3642668043
H	-0.3559271359	-1.4667600721	0.1235494203

Energy of Optimized Geometry: -271.693384917458

OX7 2-propyloxirane CAM-B3LYP STRUC7

C	-1.7491790200	-0.6840831151	-0.4510009020
C	-1.0364496119	0.5894307176	-0.4119300964
H	2.8800215500	0.6023826555	1.0136373984
H	3.2614759879	-1.0707232577	0.6218383763
H	3.1675405909	0.1339536286	-0.6584158488
H	1.1106575539	-1.3073941233	-0.5933338270
O	-1.6786384576	0.0762087326	0.7504401333
H	-2.7314231693	-0.7344261191	-0.9063688468
C	0.4610216009	0.7313888704	-0.3763147498
H	0.8142195894	-0.8175283626	1.0617007797
H	-1.1963857600	-1.6148163643	-0.4350284533
C	1.2352997177	-0.4840851289	0.1124317724
H	0.7958383759	1.0065587928	-1.3803376803
H	0.6932284609	1.5876785782	0.2635015817
C	2.7185343316	-0.1897719913	0.2817078264
H	-1.5528896930	1.4468158705	-0.8346888344

Energy of Optimized Geometry: -271.692995081373

OX7 2-propyloxirane CAM-B3LYP STRUC8

C	-1.6977351913	-0.3159653081	-0.6532050746
C	-1.0036587188	0.6060495228	0.2394662283
H	2.7608009270	-1.3936058539	-0.0602745397
H	2.5304849194	-0.1831805528	1.1959362066
H	1.2643358518	-1.3673209764	0.8745016341
H	2.1944724381	0.8003579154	-1.0700155587
O	-1.1459447626	-0.7702955585	0.5773650526
H	-2.7807639119	-0.3051180541	-0.6963252048
C	0.3741600222	1.1628729232	-0.0163180591
H	0.9572866757	-0.3699219129	-1.4242184125
H	-1.2030442322	-0.6867887603	-1.5426477663
C	1.4038846473	0.2044519578	-0.6111746417
H	0.2435624495	2.0267187980	-0.6733410060
H	0.7629655762	1.5594033011	0.9250304391
C	2.0229080976	-0.7428709537	0.4087022805
H	-1.6353846578	1.2553566157	0.8395694557

Energy of Optimized Geometry: -271.690617116648

OX7 2-propyloxirane CAM-B3LYP STRUC10

C	-1.5789453155	-0.4052785228	-0.8070235712
C	-0.9915661948	0.6783905382	-0.0259049205
H	1.0262885951	-1.3313062718	1.1334403900
H	2.6595993473	-1.4944612758	0.4817725354
H	2.2710455306	-0.1619669183	1.5629832575
H	2.3861202489	0.5984023832	-0.8093745450
O	-1.2752957007	-0.5823503314	0.5724360085
H	-2.6359108187	-0.3793822047	-1.0459654540
C	0.4388457546	1.1399462862	-0.1346428607
H	1.1652309498	-0.5507594192	-1.2671878585
H	-0.9648439826	-0.9803247379	-1.4887686692
C	1.4967752594	0.0828769782	-0.4429450938
H	0.4537830608	1.9140060072	-0.9065944041
H	0.7033894969	1.6445043760	0.7985076800
C	1.8821468591	-0.7800479631	0.7521822312
H	-1.6757020364	1.4596627851	0.2948849217

Energy of Optimized Geometry: -271.690595903811

OX7 2-propyloxirane CAM-B3LYP STRUC11

C	-1.3156028412	-0.9628749070	-0.0870214610
C	-0.9394559885	0.3566347037	-0.5871860054
H	2.7878639932	-0.5108284672	-0.8954164825
H	2.7964202119	-1.3239583733	0.6654777814
H	1.4890583649	-1.6149328990	-0.4753010260
H	0.8310327889	-0.0008205312	1.4134730294
O	-1.7117783217	0.2164748657	0.6015210284
H	-2.1194220416	-1.5084136905	-0.5681298530
C	0.4209782759	0.9890669561	-0.4491844972
H	2.0977391935	1.0636368225	0.8786591189
H	-0.5901523829	-1.5725248359	0.4357755710
C	1.3830615077	0.3184114642	0.5278120379
H	0.8752723641	1.0339352964	-1.4424934098
H	0.2495670711	2.0252687609	-0.1488441181
C	2.1534491916	-0.8495487603	-0.0753066339
H	-1.5207594810	0.7389329371	-1.4216445142

Energy of Optimized Geometry: -271.690896964706

9.3 M06-2X

OX7 2-propyloxirane M06-2X STRUC1

C	-1.3598581931	-1.0097586431	0.0562191666
C	-0.7364714241	0.2225065745	-0.4318016932
H	3.1127322470	-1.0944427316	-0.2800518325
H	1.9845278744	-1.1830261962	1.0719862544
H	1.4293665619	-1.5276393412	-0.5634284345
H	2.4658147445	1.2002356377	0.3774078647
O	-2.0376575557	0.2334359986	0.1300359215
H	-1.7783427110	-1.7220811359	-0.6447916858
C	0.3386651765	0.9336925619	0.3405036368
H	1.8266058907	0.8549722253	-1.2117672347
H	-1.0361657336	-1.4139019906	1.0091933786
C	1.7405240719	0.5844880090	-0.1561270086
H	0.1709463383	2.0097097796	0.2572772189
H	0.2346545463	0.6739858065	1.3970084376
C	2.0876468531	-0.8891587897	0.0258437407
H	-0.7242016557	0.3814101719	-1.5076595217

Energy of Optimized Geometry: -271.712568589000

OX7 2-propyloxirane M06-2X STRUC2

C	-1.8327964848	-0.7999350685	-0.2168885747
C	-0.8988663942	0.3105047049	-0.4167953797
H	3.1320578420	0.9007645202	0.5043057428
H	2.8001584740	-0.6362105641	1.2967758206
H	3.6829102389	-0.6049703644	-0.2290413763
H	1.6428804348	0.2343548470	-1.4035649719
O	-2.0933064171	0.4849988417	0.3254921711
H	-2.4957127784	-1.1047370205	-1.0174056874
C	0.3948075955	0.3928691882	0.3406719124
H	1.3109731108	-1.2946996436	-0.6202299782
H	-1.5858954030	-1.5630747773	0.5131505707
C	1.5481512611	-0.2458741618	-0.4264355888
H	0.6245160945	1.4417260672	0.5470767809
H	0.2631316994	-0.0995002894	1.3072474528
C	2.8681026268	-0.1425151883	0.3265078670
H	-0.9117707495	0.8032864793	-1.3854448637

Energy of Optimized Geometry: -271.712704685416

OX7 2-propyloxirane M06-2X STRUC3

C	-2.1899777412	-0.5009588468	-0.1856824276
C	-0.9199116333	0.1108816717	-0.5789884509
H	3.5784201154	0.5804992581	0.6047966204
H	2.7316005930	-0.8420736692	1.2086563306
H	3.1795245834	-0.7410394916	-0.4918199385
H	1.1390318946	1.0765018957	0.9038387895
O	-1.6375150557	0.5761122446	0.5547753581
H	-3.0960051468	-0.2666557030	-0.7312601775
C	0.4047103720	-0.5363122180	-0.2977690026
H	1.5914374358	1.1877457845	-0.7847191640
H	-2.1745552678	-1.4597423340	0.3200670319
C	1.4794608671	0.4895932703	0.0487260675
H	0.2813962795	-1.2372195354	0.5316994109
H	0.7127871933	-1.1174699072	-1.1716703767
C	2.8199836627	-0.1620607079	0.3602832697
H	-0.9377319426	0.8066199950	-1.4139905066

Energy of Optimized Geometry: -271.713126903987

OX7 2-propyloxirane M06-2X STRUC4

C	-2.0944259169	-0.0058573014	-0.2390939342
C	-0.8020249505	0.3620279076	0.3419607083
H	1.9467448467	-1.2844500803	-0.7950501269
H	3.0196368638	-0.9750361479	0.5730182037
H	1.3113255020	-1.3330831566	0.8401971421
H	1.7523434056	1.1237400120	1.0907819677
O	-1.1396527691	-0.9974515103	0.1005212533
H	-2.9870043992	0.0036911386	0.3745228775
C	0.3060874570	0.9468479490	-0.4875677350
H	2.4392708795	1.1765699234	-0.5163548419
H	-2.2478567718	0.1368835818	-1.3029743828
C	1.6906469421	0.6721400486	0.0969971542
H	0.2328935562	0.5275205312	-1.4943426072
H	0.1455754737	2.0246222375	-0.5697211657
C	2.0133127177	-0.8152455886	0.1874291798
H	-0.7852126494	0.6189357010	1.3985305006

Energy of Optimized Geometry: -271.712715187953

OX7 2-propyloxirane M06-2X STRUC5

C	-1.6355918263	-0.7302702073	0.5008264112
C	-0.6744955845	-0.0713623834	-0.3875096197
H	3.3034081515	-0.9858515204	-0.1844228032
H	1.7611607070	-1.3815571660	-0.9280434795
H	2.5761856578	0.1246471731	-1.3444791861
H	1.3835820639	-0.5596425952	1.3950753125
O	-2.0243075782	0.3593915469	-0.3200087602
H	-2.0621270619	-1.6877788727	0.2279988709
C	0.3620377421	0.8707417081	0.1586173015
H	2.3068075979	0.8887036306	1.0577216358
H	-1.5970923356	-0.5122235792	1.5626940230
C	1.6355325327	0.1555603050	0.6081461234
H	0.6067603294	1.6147963211	-0.6042838583
H	-0.0873209039	1.4069608297	0.9964814721
C	2.3590846719	-0.5637156730	-0.5258306599
H	-0.4322629761	-0.5748932510	-1.3179595186

Energy of Optimized Geometry: -271.711752854334

OX7 2-propyloxirane M06-2X STRUC6

C	-2.1175772972	-0.2063772857	0.3638712335
C	-0.6755568113	-0.4429226390	0.2748435243
H	3.2265718559	-0.3801859662	-0.9282438929
H	2.6833644045	-0.9322656339	0.6550558881
H	1.7677180454	-1.3486938035	-0.7936196921
H	2.1613207076	1.5125168118	0.2337896217
O	-1.4234928509	-0.0303951655	-0.8607947000
H	-2.8035476506	-1.0445359111	0.3871311755
C	0.3286487530	0.5491680434	0.7895850407
H	1.1559650973	1.0462339711	-1.1229941683
H	-2.4701917912	0.7087773493	0.8259063766
C	1.5233784575	0.7148567567	-0.1499768088
H	-0.1733526698	1.5096937210	0.9197085869
H	0.6729832914	0.2223415889	1.7749402475
C	2.3453342640	-0.5588283141	-0.3134628962
H	-0.3488614245	-1.4770395748	0.2213385720

Energy of Optimized Geometry: -271.712340288130

OX7 2-propyloxirane M06-2X STRUC7

C	-1.7257587081	-0.7041357637	-0.4413752715
C	-1.0399198399	0.5906698812	-0.4181347996
H	2.8419718188	0.5791299168	1.0634946865
H	3.2518558314	-1.0726841832	0.6059508135
H	3.1563551309	0.1859028653	-0.6242241033
H	1.1141692608	-1.2831168799	-0.6423595318
O	-1.6688655380	0.0742528784	0.7443916877
H	-2.7072970176	-0.7809060083	-0.8931428459
C	0.4575918151	0.7472237587	-0.3721631401
H	0.7847996668	-0.8451680777	1.0254556861
H	-1.1460375831	-1.6179985714	-0.4128226090
C	1.2240051587	-0.4840150669	0.0935086733
H	0.8030833663	1.0476477759	-1.3651266123
H	0.6802639162	1.5846181399	0.2962643135
C	2.7033770636	-0.1839977513	0.2968508328
H	-1.5737312172	1.4319956848	-0.8501479173

Energy of Optimized Geometry: -271.712385130700

OX7 2-propyloxirane M06-2X STRUC8

C	-1.7122665142	-0.2224891889	-0.7107154979
C	-0.9909233812	0.5950408877	0.2669816685
H	2.7070922684	-1.4462403095	0.0744419439
H	2.5419865187	-0.1413408110	1.2470976024
H	1.2273288832	-1.3006563429	1.0258085020
H	2.1885704448	0.7160285286	-1.0632589587
O	-1.1258650631	-0.8078426487	0.4398647070
H	-2.7955810761	-0.2127203878	-0.7212411407
C	0.3872725695	1.1605105995	0.0087789054
H	0.9189438496	-0.4527131935	-1.3286212361
H	-1.2347892487	-0.4778637989	-1.6496417354
C	1.3942880818	0.1613896182	-0.5616484228
H	0.2659602590	2.0119546217	-0.6651172951
H	0.7817998044	1.5637800189	0.9447872378
C	2.0024841752	-0.7377940389	0.5094168206
H	-1.6025706312	1.1700592892	0.9556194830

Energy of Optimized Geometry: -271.710279332293

OX7 2-propyloxirane M06-2X STRUC10

C	-1.4263190818	-0.6947522362	-0.5536068789
C	-1.0511512333	0.5956742745	0.0314126445
H	1.0654566144	-1.4991243654	0.4762858694
H	2.7374499817	-1.2988760089	-0.0636596497
H	2.1205592605	-0.3708353109	1.3032989786
H	2.3054681722	0.9925107927	-0.8045241830
O	-1.1825593188	-0.5652901304	0.8377295945
H	-2.4582632927	-0.8816687313	-0.8252829137
C	0.3110491763	1.2284323049	-0.1018982331
H	1.1688862327	-0.0635434571	-1.5967173408
H	-0.6853104062	-1.2721451477	-1.0943168120
C	1.4445444164	0.3464837476	-0.6224182618
H	0.1942237566	2.0884519165	-0.7658791490
H	0.5854951703	1.6328748852	0.8772050661
C	1.8633409614	-0.7756535632	0.3233702715
H	-1.8571100820	1.3069427997	0.1898888261

Energy of Optimized Geometry: -271.710445644735

OX7 2-propyloxirane M06-2X STRUC11

C	-1.3057566680	-0.9390490020	-0.1031622776
C	-0.9199478038	0.3894717137	-0.5905525176
H	2.7881633032	-0.5316979223	-0.8645371325
H	2.6870110965	-1.4553826295	0.6329461948
H	1.4125044427	-1.5855984404	-0.5783182966
H	0.7852801423	-0.0627016378	1.4143299640
O	-1.6978699868	0.2352046648	0.5866295862
H	-2.1090865508	-1.4741889558	-0.5957626769
C	0.4459273777	1.0065793718	-0.4176887061
H	2.1020889417	0.9787262701	0.9475803753
H	-0.5831816954	-1.5530267211	0.4198409846
C	1.3686244190	0.2741660190	0.5544075642
H	0.9204674044	1.0714972261	-1.4004955098
H	0.2831995867	2.0339424738	-0.0836631117
C	2.1036606787	-0.8937370760	-0.0960663044
H	-1.4924207139	0.7812425407	-1.4262578023

Energy of Optimized Geometry: -271.711021754037

9.4 MN15

OX7 2-propyloxirane MN15 STRUC1

C	-1.3538362531	-0.9882230934	0.0781945930
C	-0.7289897104	0.2268746317	-0.4467631569
H	3.0794719786	-1.1590003086	-0.2050395748
H	1.8990444496	-1.1925494821	1.1055476561
H	1.3964508855	-1.5514101526	-0.5431683192
H	2.4808462152	1.1664581649	0.3717944020
O	-2.0280028939	0.2552937555	0.1143933642
H	-1.7713125137	-1.7245898178	-0.5986345377
C	0.3480424296	0.9455679016	0.3144278703
H	1.8516946579	0.8044920512	-1.2179491191
H	-1.0228220715	-1.3638727668	1.0413562321
C	1.7469140864	0.5576605898	-0.1579488729
H	0.1988467147	2.0217504906	0.2050801848
H	0.2278658525	0.7097654662	1.3757311295
C	2.0501368400	-0.9198142186	0.0589484720
H	-0.7092731016	0.3558910987	-1.5267989819

Energy of Optimized Geometry: -271.500821179315

OX7 2-propyloxirane MN15 STRUC2

C	-1.8178400400	-0.8038777657	-0.2219296954
C	-0.8990624975	0.3183882161	-0.4194873356
H	3.1205345034	0.9014851180	0.5158061576
H	2.7819491550	-0.6353401648	1.3043269113
H	3.6787375802	-0.6036615221	-0.2134043348
H	1.6419232435	0.2388296132	-1.4024608992
O	-2.0925323688	0.4733877780	0.3244035982
H	-2.4770879198	-1.1182948413	-1.0221901075
C	0.3928488820	0.3991971877	0.3397642047
H	1.3027569193	-1.2900718021	-0.6221747102
H	-1.5543860132	-1.5680098072	0.5023367381
C	1.5439188832	-0.2422790494	-0.4257703548
H	0.6284914109	1.4457240278	0.5517560725
H	0.2553064611	-0.0973809671	1.3041928893
C	2.8583450803	-0.1418525057	0.3343539846
H	-0.9131122402	0.8149697690	-1.3865016500

Energy of Optimized Geometry: -271.500678993889

OX7 2-propyloxirane MN15 STRUC3

C	-2.1871953899	-0.4879849653	-0.1665036587
C	-0.9211845869	0.1158165265	-0.5816772163
H	3.5793576405	0.5494165710	0.5978745074
H	2.7145956208	-0.8633035406	1.2001667650
H	3.1624510819	-0.7656562445	-0.5000490708
H	1.1394315106	1.0708341744	0.8967821935
O	-1.6228407049	0.5917624922	0.5551699783
H	-3.1004971345	-0.2602175819	-0.7033742769
C	0.3989159985	-0.5392091846	-0.3015056984
H	1.5920153486	1.1782344194	-0.7919741796
H	-2.1651073216	-1.4452133572	0.3434041294
C	1.4775035002	0.4812254607	0.0424644242
H	0.2684879345	-1.2348235264	0.5323100879
H	0.7063492073	-1.1280398536	-1.1707979518
C	2.8108298676	-0.1825468925	0.3529085047
H	-0.9407515706	0.8023714416	-1.4244921663

Energy of Optimized Geometry: -271.501108409721

OX7 2-propyloxirane MN15 STRUC4

C	-2.0897062202	0.0035320061	-0.2418891396
C	-0.7971260838	0.3555895179	0.3460919421
H	1.9183678423	-1.2919643005	-0.7807811919
H	3.0152519613	-0.9841237704	0.5694406393
H	1.3084222625	-1.3241030083	0.8635520339
H	1.7543981318	1.1277508723	1.0923300312
O	-1.1426078095	-0.9960095157	0.0840748089
H	-2.9854752061	0.0119515805	0.3677001358
C	0.3082582324	0.9439898018	-0.4836236419
H	2.4424045927	1.1708600420	-0.5148098005
H	-2.2396463074	0.1681383012	-1.3037789152
C	1.6918608933	0.6713215722	0.1003666101
H	0.2316980780	0.5172829694	-1.4879528709
H	0.1460426580	2.0210690081	-0.5723526625
C	2.0051483574	-0.8163321173	0.1973140856
H	-0.7744649785	0.6012245112	1.4055902592

Energy of Optimized Geometry: -271.500804881046

OX7 2-propyloxirane MN15 STRUC5

C	-1.6207306862	-0.7273318239	0.5028704151
C	-0.6729137716	-0.0704910010	-0.3994857070
H	3.2937873659	-0.9920387125	-0.1693985592
H	1.7543466068	-1.3746756409	-0.9259054149
H	2.5797030810	0.1291491599	-1.3275549602
H	1.3563489456	-0.5605472055	1.3969383508
O	-2.0222189454	0.3527031743	-0.3198573967
H	-2.0443546257	-1.6907374988	0.2455294487
C	0.3604449135	0.8741554184	0.1469420731
H	2.2927838429	0.8833389973	1.0762011635
H	-1.5655869043	-0.5029914854	1.5635090956
C	1.6236611260	0.1553532510	0.6150152522
H	0.6180049252	1.6135839098	-0.6163970825
H	-0.1011662651	1.4144807186	0.9762361385
C	2.3538750830	-0.5625967239	-0.5137805965
H	-0.4313512615	-0.5771184178	-1.3287709818

Energy of Optimized Geometry: -271.499900120627

OX7 2-propyloxirane MN15 STRUC6

C	-2.0942476422	-0.2508906220	0.4452068677
C	-0.6565429232	-0.4657344869	0.2799128966
H	3.1998498792	-0.3125785041	-1.0489238964
H	2.7404440106	-0.8614810565	0.5620608707
H	1.7773328311	-1.3214093873	-0.8409363424
H	2.1249134354	1.5587585043	0.1505361111
O	-1.4624679498	-0.0252432722	-0.8016531780
H	-2.7708046662	-1.0968537957	0.4758762953
C	0.3489308988	0.5323599521	0.7790036021
H	1.0806516669	1.0564436885	-1.1633071031
H	-2.4313616197	0.6425295510	0.9599417024
C	1.4982584211	0.7394814556	-0.2055605579
H	-0.1715900775	1.4774568243	0.9483615806
H	0.7409773553	0.1947098719	1.7430697801
C	2.3510624616	-0.5086266548	-0.3950812422
H	-0.3146578932	-1.4916815578	0.1785384507

Energy of Optimized Geometry: -271.500490190734

OX7 2-propyloxirane MN15 STRUC7

C	-1.7623476030	-0.6627771047	-0.3177641609
C	-1.0490140947	0.6158814495	-0.3608115097
H	2.8794430485	0.5220402720	0.9048462434
H	3.2454574603	-1.1398755510	0.4446520677
H	3.1175467191	0.1112719718	-0.7906875693
H	1.0482823834	-1.3189974949	-0.7153396988
O	-1.5996114451	0.1240774074	0.8484163379
H	-2.7753887108	-0.7243798561	-0.6978234179
C	0.4511342973	0.7276696307	-0.4275849230
H	0.7960605760	-0.8661779189	0.9616609157
H	-1.1997566072	-1.5889480781	-0.3277853581
C	1.2065463337	-0.5190161721	0.0111731587
H	0.7358623601	0.9968419523	-1.4485554348
H	0.7503793269	1.5696895607	0.2046837013
C	2.6973211495	-0.2455125083	0.1513100955
H	-1.5907501311	1.4665895680	-0.7644299983

Energy of Optimized Geometry: -271.500845890542

OX7 2-propyloxirane MN15 STRUC8

C	-1.7060464017	-0.1972151415	-0.7272854095
C	-0.9888212216	0.5953835660	0.2721713874
H	2.6986846274	-1.4608851816	0.0925187687
H	2.5474130093	-0.1389718943	1.2488833888
H	1.2261366185	-1.2944056024	1.0508943913
H	2.1781632601	0.6916152809	-1.0706534434
O	-1.1315573988	-0.8081882662	0.4126347233
H	-2.7894466412	-0.1805818029	-0.7492856227
C	0.3941072066	1.1536459035	0.0276705836
H	0.8958027454	-0.4713972941	-1.3055839815
H	-1.2222186695	-0.4299591913	-1.6697631820
C	1.3873697481	0.1474765677	-0.5520663734
H	0.2845386151	2.0169935584	-0.6331405369
H	0.7933827711	1.5378171634	0.9702409322
C	2.0006719466	-0.7424092694	0.5217253507
H	-1.5998911770	1.1628836426	0.9678379395

Energy of Optimized Geometry: -271.498838995264

OX7 2-propyloxirane MN15 STRUC10

C	-1.4075973231	-0.6930949660	-0.5836043652
C	-1.0496433549	0.5996778783	0.0046726617
H	1.0540365467	-1.5092759616	0.4932233805
H	2.7410235442	-1.3010739633	0.0083795706
H	2.0748130104	-0.3753241967	1.3539254350
H	2.3249776207	0.9810880952	-0.7575610839
O	-1.2103129844	-0.5546864550	0.8113251531
H	-2.4292136732	-0.8806611859	-0.8926682539
C	0.3186550932	1.2247964953	-0.0931073241
H	1.1998127024	-0.0717281607	-1.5702268474
H	-0.6491496230	-1.2724017398	-1.0990990442
C	1.4579626959	0.3387377280	-0.5907746379
H	0.2236704507	2.0896058060	-0.7546761863
H	0.5740010506	1.6229781443	0.8939874271
C	1.8523339342	-0.7811498785	0.3656965789
H	-1.8567889491	1.3166546310	0.1302161193

Energy of Optimized Geometry: -271.498778581316

OX7 2-propyloxirane MN15 STRUC11

C	-1.2971686172	-0.9407530779	-0.1313670093
C	-0.9060780222	0.3856765285	-0.6171352848
H	2.8118526914	-0.5115675073	-0.7816713205
H	2.6681603876	-1.4482781865	0.7048685508
H	1.4331757034	-1.5739385359	-0.5477721338
H	0.7234971586	-0.0673237470	1.4256554192
O	-1.7044844179	0.2335314488	0.5440286280
H	-2.0871523537	-1.4830511257	-0.6382594753
C	0.4557055128	1.0016495444	-0.4165106655
H	2.0539987529	0.9824222438	1.0195950554
H	-0.5808671380	-1.5516940569	0.4050931112
C	1.3416271666	0.2759019202	0.5923449196
H	0.9620138240	1.0481108833	-1.3850769722
H	0.2889895092	2.0354598651	-0.1052102215
C	2.1055449732	-0.8828543143	-0.0373554016
H	-1.4594792096	0.7731566359	-1.4679065208

Energy of Optimized Geometry: -271.499611651011

9.5 ωB97X-D

OX7 2-propyloxirane ωB97X-D STRUC1

C	-1.4027314177	-0.9705612899	0.0623555929
C	-0.7345493561	0.2263981631	-0.4404292923
H	3.1185057103	-1.1561367839	-0.2294974694
H	1.9991086233	-1.1929840530	1.1317374789
H	1.4327062128	-1.5935017380	-0.4870519871
H	2.4878094470	1.1599445345	0.3548588644
O	-2.0427541475	0.2948015376	0.1049182103
H	-1.8344780351	-1.6850942817	-0.6301873815
C	0.3594056174	0.9231740339	0.3201763048
H	1.8521091021	0.7650968833	-1.2228911684
H	-1.1069942954	-1.3708389150	1.0269283863
C	1.7572824582	0.5325426761	-0.1586214064
H	0.2216599212	2.0011221545	0.2086330231
H	0.2470264254	0.6973484076	1.3834970667
C	2.0957309291	-0.9355340517	0.0753028201
H	-0.7057778122	0.3632265251	-1.5197204563

Energy of Optimized Geometry: -271.752498375306

OX7 2-propyloxirane ωB97X-D STRUC2

C	-1.8457921290	-0.7914103345	-0.2066940170
C	-0.9006813800	0.3024204007	-0.4111444790
H	3.1436452098	0.8962225251	0.4928606131
H	2.8210508964	-0.6444422619	1.2829180131
H	3.6874205440	-0.6063562052	-0.2519555425
H	1.6429269572	0.2297116542	-1.4078641807
O	-2.0952668989	0.4995419038	0.3284815011
H	-2.5092116235	-1.0958688319	-1.0087555817
C	0.3976764448	0.3846084948	0.3401325520
H	1.3189503074	-1.3002486068	-0.6244345412
H	-1.6139703890	-1.5567311766	0.5274907272
C	1.5519407970	-0.2503807964	-0.4299207089
H	0.6243340368	1.4348198716	0.5435330633
H	0.2754310477	-0.1047679426	1.3094019372
C	2.8771571223	-0.1466222260	0.3135127386
H	-0.9069566976	0.7894673389	-1.3837874442

Energy of Optimized Geometry: -271.752707903099

OX7 2-propyloxirane ωB97X-D STRUC3

C	-2.1933324444	-0.4908780813	-0.1998007018
C	-0.9202571343	0.1197091434	-0.5673546978
H	3.5968918462	0.5598495434	0.5846913551
H	2.7529409235	-0.8602102956	1.1982583677
H	3.1834311764	-0.7609300386	-0.5074338325
H	1.1719419424	1.0715435825	0.9103381563
O	-1.6574034633	0.5836892193	0.5568023173
H	-3.0901138443	-0.2574400816	-0.7634981741
C	0.4019862002	-0.5330992008	-0.2841540363
H	1.6040765852	1.1780574691	-0.7826253243
H	-2.1922742377	-1.4532623005	0.3020465198
C	1.4932394752	0.4796026035	0.0511848278
H	0.2796422827	-1.2341813928	0.5452430121
H	0.6975613291	-1.1196222349	-1.1594150698
C	2.8329427858	-0.1805203463	0.3481240331
H	-0.9244164448	0.8184688766	-1.4012026986

Energy of Optimized Geometry: -271.752997627383

OX7 2-propyloxirane ωB97X-D STRUC4

C	-2.1044249368	0.0494825961	-0.2421986969
C	-0.8078054984	0.3769473804	0.3416746431
H	2.0066315473	-1.2892309483	-0.8461780659
H	3.0510392301	-1.0097854936	0.5485453081
H	1.3467125648	-1.4066127530	0.7768503296
H	1.7581015026	1.0500795072	1.1200352768
O	-1.1911568173	-0.9749142967	0.1157104253
H	-3.0020388396	0.1026680378	0.3642417194
C	0.3259517852	0.9282249639	-0.4776536377
H	2.4558628782	1.1633476475	-0.4778065495
H	-2.2508495508	0.1835184184	-1.3093530867
C	1.7051253831	0.6320376005	0.1107022564
H	0.2533804312	0.5206540616	-1.4892028464
H	0.1891993761	2.0102179922	-0.5558218576
C	2.0475044683	-0.8528112941	0.1529671414
H	-0.7910599889	0.6468111422	1.3957119920

Energy of Optimized Geometry: -271.752283136933

OX7 2-propyloxirane ωB97X-D STRUC5

C	-1.6740765030	-0.6971439098	0.5119051816
C	-0.6851431910	-0.0785323789	-0.3668071665
H	3.3232573035	-1.0011042416	-0.1960815506
H	1.7930306881	-1.3761704839	-0.9744438095
H	2.6171908495	0.1375551216	-1.3414982504
H	1.4003573548	-0.6059165090	1.3734936739
O	-2.0260500256	0.3875435336	-0.3326461805
H	-2.1163392847	-1.6516414820	0.2481903561
C	0.3720481901	0.8483390103	0.1673187882
H	2.3146756433	0.8524598383	1.0659333360
H	-1.6528655413	-0.4665202480	1.5726375504
C	1.6480436433	0.1249896717	0.5994325746
H	0.6119616933	1.5906534336	-0.5990410290
H	-0.0528561552	1.3924099473	1.0129256923
C	2.3847063199	-0.5686148486	-0.5421591989
H	-0.4398806521	-0.6053360838	-1.2846173155

Energy of Optimized Geometry: -271.751759605965

OX7 2-propyloxirane ωB97X-D STRUC6

C	-2.1170786725	-0.1925499740	0.4206562003
C	-0.6852074130	-0.4251077997	0.2625451706
H	3.2542095613	-0.4241950226	-0.9350338422
H	2.7285737386	-0.9194325758	0.6726721003
H	1.8075909665	-1.4029565201	-0.7511277555
H	2.1814148090	1.4967535067	0.1738428956
O	-1.4864175750	-0.0108089278	-0.8374778688
H	-2.8003205417	-1.0330478599	0.4782028376
C	0.3452921982	0.5558343419	0.7475659875
H	1.1916832739	0.9879592029	-1.1759358000
H	-2.4542391498	0.7194266671	0.9030296067
C	1.5474179218	0.6855681027	-0.1889899902
H	-0.1333656748	1.5300592800	0.8642887162
H	0.6824962931	0.2405339574	1.7397335760
C	2.3787136860	-0.5871217950	-0.3068842321
H	-0.3602888462	-1.4596174166	0.1900967765

Energy of Optimized Geometry: -271.752233244213

OX7 2-propyloxirane ωB97X-D STRUC7

C	-1.7535140186	-0.6904707263	-0.4144890611
C	-1.0454815979	0.5874228974	-0.3969830751
H	2.8875119044	0.5972465627	0.9846241429
H	3.2738323789	-1.0701736920	0.5645220425
H	3.1586108216	0.1536992452	-0.6989081885
H	1.1093121370	-1.3022943066	-0.6280420269
O	-1.6681359327	0.0800476819	0.7747652695
H	-2.7421494947	-0.7490247723	-0.8572146677
C	0.4544599010	0.7335496991	-0.3853303933
H	0.8277250496	-0.8292118897	1.0368720198
H	-1.1942303008	-1.6190880724	-0.3999205743
C	1.2382722534	-0.4855497988	0.0856459429
H	0.7723701896	1.0123060237	-1.3942472030
H	0.6954945110	1.5873617868	0.2554018112
C	2.7235838777	-0.1861844174	0.2428705288
H	-1.5711065247	1.4397107931	-0.8206410771

Energy of Optimized Geometry: -271.751960456310

OX7 2-propyloxirane ωB97X-D STRUC8

C	-1.5007468468	-0.6151407814	-0.5378461790
C	-1.0554035562	0.5300666607	0.2517567139
H	2.7660432110	-1.1509156787	-0.5805979261
H	2.3434066711	-0.4702700178	0.9899396539
H	1.1939565011	-1.4965939772	0.1524508671
H	2.1803052102	1.2252576250	-0.8481228988
O	-1.0948934649	-0.7752039949	0.8125224049
H	-2.5593304236	-0.7620074845	-0.7233026814
C	0.2744982838	1.2252467803	0.0982687491
H	1.0041202710	0.2443492654	-1.6764229873
H	-0.8348503231	-1.0553156970	-1.2719035685
C	1.3747630712	0.5067085268	-0.6825100521
H	0.0650706381	2.1827673096	-0.3864060128
H	0.6388407725	1.4679172591	1.1012728651
C	1.9496237957	-0.7279954959	0.0054251839
H	-1.8364727005	1.1768468490	0.6460551078

Energy of Optimized Geometry: -271.750098561081

OX7 2-propyloxirane ωB97X-D STRUC10

C	-1.4668357968	-0.6136764764	-0.6429397994
C	-1.0290688739	0.6326239828	-0.0191525279
H	1.0351020871	-1.5007822928	0.6401647992
H	2.7180923650	-1.3974866836	0.1057691635
H	2.1345605741	-0.3785008453	1.4209405682
H	2.3751872043	0.8876755284	-0.7310835447
O	-1.2628177749	-0.5333413074	0.7591559224
H	-2.4996571762	-0.7250313381	-0.9552809644
C	0.3673529777	1.1984620390	-0.0992264760
H	1.2411379555	-0.1811393346	-1.5071237565
H	-0.7519584422	-1.2318560187	-1.1751689301
C	1.4954142187	0.2682634073	-0.5441190245
H	0.3149991281	2.0461795485	-0.7878707766
H	0.6103469496	1.6208298506	0.8806783741
C	1.8641793713	-0.8194443711	0.4599021179
H	-1.7937811233	1.3919555844	0.1282685157

Energy of Optimized Geometry: -271.750104614411

OX7 2-propyloxirane ωB97X-D STRUC11

C	-1.3038310436	-0.9733534314	-0.0855523598
C	-0.9421048508	0.3544459213	-0.5783844239
H	2.7762810903	-0.4892024268	-0.9219879619
H	2.7972921072	-1.3246307577	0.6296568109
H	1.4748995168	-1.5932808690	-0.5031857541
H	0.8488010135	-0.0008010994	1.4180664092
O	-1.7142556247	0.1955845681	0.6040016652
H	-2.0988423801	-1.5266692352	-0.5745908057
C	0.4128336677	1.0037939701	-0.4344887922
H	2.1088434407	1.0700787458	0.8731729462
H	-0.5717378855	-1.5782413312	0.4368442008
C	1.3889559744	0.3270191355	0.5270156898
H	0.8622003729	1.0690940934	-1.4294779114
H	0.2298021929	2.0328225200	-0.1154255944
C	2.1494110906	-0.8375700862	-0.0991972394
H	-1.5253549916	0.7330268946	-1.4142129236

Energy of Optimized Geometry: -271.750491958464

9.6 EOM-CCSD

OX7 2-propyloxirane EOM-CCSD STRUC1

C	-1.3921371767	-0.9899517451	0.0713854142
C	-0.7314979945	0.2173828192	-0.4422153272
H	3.1252026332	-1.1402168723	-0.2527932142
H	2.0094586096	-1.1859755280	1.1167751716
H	1.4339150014	-1.5776315452	-0.5063383806
H	2.4895440460	1.1789551372	0.3557854272
O	-2.0553406503	0.2861435003	0.1135090849
H	-1.8260904032	-1.7073012010	-0.6174885016
C	0.3542662388	0.9270827986	0.3249404710
H	1.8475402816	0.7944996505	-1.2287785484
H	-1.0921082855	-1.3783191036	1.0399928213
C	1.7590394226	0.5524384665	-0.1637321668
H	0.2015656937	2.0060227176	0.2202950217
H	0.2435186379	0.6879476098	1.3878694930
C	2.1013574710	-0.9229708224	0.0589261608
H	-0.7103102778	0.3528092079	-1.5224859562

Energy of Optimized Geometry: -271.277433131303

OX7 2-propyloxirane EOM-CCSD STRUC2

C	-1.8470991600	-0.7868366573	-0.2286879595
C	-0.8954748049	0.3155192741	-0.4209663594
H	3.1477114021	0.8706874699	0.5304537923
H	2.8036523033	-0.6825152699	1.2971949105
H	3.6942493983	-0.6290066556	-0.2283938285
H	1.6601152709	0.2422083805	-1.4008568444
O	-2.1033814486	0.5145608008	0.3316111605
H	-2.5150669340	-1.0761078712	-1.0329350005
C	0.3991073028	0.3804673707	0.3450747030
H	1.3161543954	-1.3027482317	-0.6416589153
H	-1.6154439509	-1.5569259102	0.5011308312
C	1.5564529090	-0.2552865424	-0.4303280381
H	0.6330176651	1.4279576260	0.5656804314
H	0.2606654628	-0.1260511208	1.3058869983
C	2.8781849632	-0.1705030101	0.3340563315
H	-0.9020823859	0.8175870597	-1.3865441118

Energy of Optimized Geometry: -271.277860756000

OX7 2-propyloxirane EOM-CCSD STRUC3

C	-2.1961379671	-0.5061167350	-0.1903004391
C	-0.9223803552	0.1097084226	-0.5824362525
H	3.5918552133	0.5788079381	0.5975709793
H	2.7446529323	-0.8458777865	1.2087438488
H	3.1893203303	-0.7479842574	-0.4981433366
H	1.1494170542	1.0798115991	0.9036180563
O	-1.6464372926	0.5884532417	0.5665110905
H	-3.1029420352	-0.2712739355	-0.7375064766
C	0.4049720924	-0.5394653123	-0.2964456454
H	1.5977838632	1.1863344317	-0.7920252722
H	-2.1808643757	-1.4637740839	0.3207010676
C	1.4856221078	0.4894912195	0.0460247549
H	0.2796088036	-1.2388903651	0.5368994153
H	0.7114219385	-1.1243057963	-1.1721413001
C	2.8311871243	-0.1666426893	0.3562498452
H	-0.9398770034	0.8071570600	-1.4179029231

Energy of Optimized Geometry: -271.278330985390

OX7 2-propyloxirane EOM-CCSD STRUC4

C	-2.1033301000	0.0337608369	-0.2463592267
C	-0.8077186114	0.3851834756	0.3489280565
H	1.9683724931	-1.2859504214	-0.8542217279
H	3.0247452470	-1.0304533303	0.5419429926
H	1.3089780870	-1.3987144782	0.7742465142
H	1.7613251504	1.0563686958	1.1259835551
O	-1.1591541064	-0.9916904681	0.1103004068
H	-3.0024125892	0.0569679360	0.3601855089
C	0.3206251493	0.9520943863	-0.4731053044
H	2.4612349248	1.1638658583	-0.4782226752
H	-2.2437906216	0.1694135734	-1.3143556560
C	1.7030626357	0.6414558196	0.1131344868
H	0.2414271720	0.5499331033	-1.4888158028
H	0.1843679954	2.0376104665	-0.5381610367
C	2.0204455938	-0.8556182392	0.1494481799
H	-0.7990760318	0.6450783142	1.4062583259

Energy of Optimized Geometry: -271.277732470196

OX7 2-propyloxirane EOM-CCSD STRUC5

C	-1.6470722764	-0.7430093539	0.5418709665
C	-0.6845942354	-0.0939053655	-0.3588660430
H	3.3399450603	-0.9356741032	-0.2838449964
H	1.7941507672	-1.3194162162	-1.0372877440
H	2.5880303899	0.2191025322	-1.3896383516
H	1.4397952586	-0.6023981207	1.3419335764
O	-2.0487816224	0.3578805962	-0.2940099726
H	-2.0824613128	-1.7002087964	0.2755582497
C	0.3585891129	0.8550520290	0.1739242362
H	2.3239263512	0.8833355455	1.0412929483
H	-1.6062337393	-0.5151022424	1.6029267035
C	1.6601339376	0.1489719290	0.5758596750
H	0.5708859665	1.6163434680	-0.5851439267
H	-0.0728415517	1.3722923595	1.0360029891
C	2.3851032789	-0.5108895098	-0.6007247829
H	-0.4523980317	-0.6047664017	-1.2894776026

Energy of Optimized Geometry: -271.276809904415

OX7 2-propyloxirane EOM-CCSD STRUC6

C	-2.1129266218	-0.2323201684	0.4308611178
C	-0.6705205280	-0.4571938362	0.2724504060
H	3.2193842520	-0.3389950298	-1.0277691050
H	2.7388878744	-0.9252064064	0.5680688652
H	1.7770620731	-1.3350201178	-0.8578263932
H	2.1656390344	1.5184336491	0.2194096070
O	-1.4681148525	0.0069674968	-0.8339260468
H	-2.7948936674	-1.0760207342	0.4416716255
C	0.3496174974	0.5217375198	0.7933203182
H	1.1330719344	1.0751754819	-1.1309477294
H	-2.4515678332	0.6624928241	0.9437511380
C	1.5249862101	0.7227348299	-0.1721295317
H	-0.1518654549	1.4784483635	0.9673057374
H	0.7197037190	0.1627345761	1.7613348368
C	2.3606633852	-0.5419863254	-0.3844566919
H	-0.3419451797	-1.4870774536	0.1570508135

Energy of Optimized Geometry: -271.277503538798

OX7 2-propyloxirane EOM-CCSD STRUC7

C	-1.7523462196	-0.6905625494	-0.4141316923
C	-1.0479298738	0.5996440937	-0.3967627290
H	2.8860861567	0.5859908507	0.9765461938
H	3.2694151474	-1.0857903878	0.5520979471
H	3.1541673633	0.1430590286	-0.7123447020
H	1.0927064038	-1.3066969302	-0.6427551579
O	-1.6558500109	0.0798151351	0.7985796399
H	-2.7495113854	-0.7484384136	-0.8379359048
C	0.4561169711	0.7412556668	-0.3935821807
H	0.8129579605	-0.8325134404	1.0290649235
H	-1.1845386458	-1.6142194002	-0.4088508652
C	1.2279158608	-0.4923315155	0.0759965771
H	0.7743142727	1.0175792184	-1.4058444413
H	0.7067065712	1.5916647944	0.2519920462
C	2.7205654871	-0.1975241314	0.2322429579
H	-1.5851524472	1.4531798117	-0.8046672217

Energy of Optimized Geometry: -271.277111702689

OX7 2-propyloxirane EOM-CCSD STRUC8

C	-1.7068550817	-0.2729264927	-0.6865292863
C	-0.9980540644	0.6179893534	0.2417284726
H	2.7292210360	-1.4384244224	0.0348621539
H	2.5344638964	-0.1504054900	1.2276746945
H	1.2338087940	-1.3256635863	0.9765346459
H	2.1996938000	0.7280430166	-1.0861901071
O	-1.1377939347	-0.7844100247	0.5327752644
H	-2.7914947986	-0.2619497703	-0.7138416431
C	0.3883175951	1.1675924458	-0.0237770073
H	0.9309968776	-0.4415655389	-1.3750013510
H	-1.2141277416	-0.6083594147	-1.5930567627
C	1.4002363875	0.1653998440	-0.5950443527
H	0.2677421468	2.0176686848	-0.7048803145
H	0.7857418570	1.5769030840	0.9116991771
C	2.0084462197	-0.7451165327	0.4749762546
H	-1.6217715607	1.2445694668	0.8755755503

Energy of Optimized Geometry: -271.275083585480

OX7 2-propyloxirane EOM-CCSD STRUC10

C	-1.4490160740	-0.6488609617	-0.6490066700
C	-1.0486585191	0.6122017437	-0.0083156090
H	1.0627084851	-1.4729205524	0.6371752729
H	2.7458943307	-1.3301821744	0.0941220932
H	2.1415753690	-0.3186585921	1.4110438494
H	2.3460260813	0.9467537187	-0.7517976084
O	-1.2282077590	-0.5833704644	0.7719429164
H	-2.4829145363	-0.7952878936	-0.9437646086
C	0.3335260513	1.2192918944	-0.0974338233
H	1.2213302512	-0.1475077881	-1.5190868043
H	-0.7145281437	-1.2325268050	-1.1937676498
C	1.4782293380	0.3086311416	-0.5574022545
H	0.2538374241	2.0682653605	-0.7863745514
H	0.5774130941	1.6430323519	0.8842210807
C	1.8769417981	-0.7725394571	0.4517878163
H	-1.8400959229	1.3398171495	0.1612192883

Energy of Optimized Geometry: -271.275028808934

OX7 2-propyloxirane EOM-CCSD STRUC11

C	-1.2990196865	-0.9654989078	-0.0945599124
C	-0.9305491720	0.3665548834	-0.5980357341
H	2.7894272898	-0.5101998329	-0.8829006901
H	2.7482956221	-1.3846382202	0.6517203591
H	1.4497010461	-1.5975060213	-0.5270497068
H	0.8024411451	-0.0236535230	1.4187569855
O	-1.7131734470	0.2228480929	0.6011347726
H	-2.1007211129	-1.5144815413	-0.5781576546
C	0.4309772688	1.0062725512	-0.4388175575
H	2.1003773122	1.0346121987	0.9169191599
H	-0.5677640540	-1.5652201892	0.4350924669
C	1.3744994473	0.3060421583	0.5458853653
H	0.9018479229	1.0597775614	-1.4274495405
H	0.2550040384	2.0396911316	-0.1221416439
C	2.1310743077	-0.8662416101	-0.0855776415
H	-1.5140475960	0.7450654001	-1.4344968294

Energy of Optimized Geometry: -271.275454712782

10 (2-methyloxiran-2-yl)methanol (OX8)

10.1 BMK

OX8 (2-methyloxiran-2-yl)methanol BMK STRUC1

O	-1.9802080156	0.0853300505	-0.0380386484
H	1.7725145132	1.7220642265	-0.7779388030
H	1.6557027635	1.6019630557	0.9852742350
C	-0.8700497353	0.9351240583	-0.0634338931
H	2.5854763859	0.3968335769	0.0786893056
H	-1.8194305004	-0.5765691538	0.6414338621
C	0.4524014096	0.1726443478	-0.0789962742
H	-0.9472449089	1.5515206505	-0.9617513365
H	-0.8646837355	1.6075683108	0.8060501136
C	1.6946072971	1.0242525284	0.0589284322
C	0.5123946951	-1.1656297570	-0.7026562698
O	0.4188047874	-1.0111837300	0.7195707823
H	1.4674714441	-1.5425182843	-1.0536450953
H	-0.3747312106	-1.5735468050	-1.1738731386

Energy of Optimized Geometry: -307.572726014442

OX8 (2-methyloxiran-2-yl)methanol BMK STRUC2

O	-1.7754601428	0.8575352838	0.4660422425
H	2.2068093684	0.7993644817	0.6003427025
H	1.4358187967	1.8303476151	-0.6243511600
C	-1.0444941965	0.3385265721	-0.6178870615
H	0.7515279061	1.7337493621	1.0049857618
H	-1.7850017935	0.1976400990	1.1635517093
C	0.3800767304	0.0032767291	-0.2074200102
H	-1.5234878635	-0.5599345647	-1.0229482455
H	-1.0430399713	1.1098145468	-1.3904278291
C	1.2521493392	1.1633635502	0.2208317614
C	0.9802620166	-1.2674865004	-0.6732627481
O	0.4396995945	-1.1518211651	0.6393410259
H	2.0615211020	-1.3540216422	-0.7165175915
H	0.4255070728	-1.9164486265	-1.3440618098

Energy of Optimized Geometry: -307.570843717983

OX8 (2-methyloxiran-2-yl)methanol BMK STRUC3

O	-1.8140633699	0.9599221639	-0.5012220729
H	0.7647544865	1.7828456628	-1.0052073141
H	1.3539197728	1.7540601475	0.6630226070
C	-1.0878448683	0.2810661068	0.4962798240
H	2.2045491082	0.8241008679	-0.5835556550
H	-1.9241529878	0.3839688028	-1.2596658813
C	0.3365462985	-0.0334824669	0.0586098612
H	-1.0627666046	0.9482803645	1.3605834623
H	-1.5779585779	-0.6501662109	0.8008016029
C	1.2239601901	1.1541594927	-0.2409544339
C	0.6074129585	-1.3603198097	-0.5532888935
O	0.9400465383	-1.0853777402	0.8026712410
H	1.4560453916	-1.4744562484	-1.2202493371
H	-0.2033721881	-2.0707827587	-0.6900037234

Energy of Optimized Geometry: -307.568669728470

OX8 (2-methyloxiran-2-yl)methanol BMK STRUC5

O	-1.8190775352	0.9492015293	-0.5320923413
H	0.8446410867	1.6977533955	-1.1000973084
H	1.2660292947	1.7955347124	0.6229172737
C	-1.0998166808	0.2609767064	0.4643548894
H	2.2353427671	0.7979409946	-0.4730793296
H	-1.5537779880	1.8689254832	-0.5486435097
C	0.3303488194	-0.0545328497	0.0454257644
H	-1.0771664099	0.8281494215	1.4034703459
H	-1.6273827189	-0.6745006490	0.6520471828
C	1.2234611977	1.1327344045	-0.2456752378
C	0.6265640735	-1.3795712973	-0.5424778081
O	0.9199392698	-1.0908783227	0.8244297465
H	1.4965458172	-1.4964301560	-1.1807841791
H	-0.1802670506	-2.0879067175	-0.7009048570

Energy of Optimized Geometry: -307.567572777869

OX8 (2-methyloxiran-2-yl)methanol BMK STRUC6

O	-1.7336907642	0.9589385409	-0.5473569857
H	2.1992848642	0.8022540663	-0.5806008345
H	0.7612551258	1.7345807753	-1.0634792591
C	-1.0855782142	0.2723145255	0.5008972823
H	1.3202427564	1.7688193474	0.6174777922
H	-2.5947978605	1.2577954483	-0.2545417653
C	0.3277627327	-0.0475288470	0.0488616265
H	-1.0337673253	0.8910898031	1.4060505842
H	-1.5956282120	-0.6640112833	0.7510583653
C	1.2106278739	1.1383315761	-0.2684710308
C	0.5947563369	-1.3761771215	-0.5464506250
O	0.9302007779	-1.0886427844	0.8102600867
H	1.4438271117	-1.4977621280	-1.2111345509
H	-0.2216686950	-2.0787639638	-0.6800459259

Energy of Optimized Geometry: -307.568141972775

OX8 (2-methyloxiran-2-yl)methanol BMK STRUC8

O	-1.7929208594	0.9799176202	0.4025005625
H	2.2237452133	0.8592958459	0.3788045995
H	1.2078532298	1.8615835248	-0.6763701031
C	-1.0957396526	0.2097137558	-0.5469692076
H	0.8006587682	1.6680259943	1.0425383099
H	-1.4954023344	1.8895332922	0.3674300495
C	0.3530475216	-0.0539499876	-0.1636678835
H	-1.6172582758	-0.7458640888	-0.6103554931
H	-1.1219417399	0.6851862197	-1.5361222505
C	1.1990905130	1.1597573955	0.1615909832
C	0.9856563795	-1.3020707009	-0.6541199042
O	0.5554688461	-1.1719893185	0.6946858321
H	2.0614824306	-1.3298163665	-0.7976854509
H	0.4098251579	-1.9997795427	-1.2554923664

Energy of Optimized Geometry: -307.566711594163

OX8 (2-methyloxiran-2-yl)methanol BMK STRUC9

O	-1.6965387888	1.0245144603	0.3808019684
H	1.2066297926	1.8440209577	-0.7049041109
H	0.7096441501	1.7186140237	0.9884752125
C	-1.0898167529	0.1827982709	-0.5738514135
H	2.1820012336	0.8909356319	0.4360045373
H	-2.6266046921	1.1259333465	0.1782060907
C	0.3489222397	-0.0543507237	-0.1599640762
H	-1.5970743148	-0.7859863131	-0.6318450393
H	-1.0997942278	0.6497095682	-1.5678479683
C	1.1674935347	1.1777861315	0.1598538093
C	1.0025375907	-1.3027539331	-0.6182015011
O	0.5420859193	-1.1599951156	0.7191692353
H	2.0814669372	-1.3181294324	-0.7382611899
H	0.4492511188	-2.0163542022	-1.2222859997

Energy of Optimized Geometry: -307.567328207888

OX8 (2-methyloxiran-2-yl)methanol BMK STRUC10

O	-1.9539157008	0.3197587420	0.1193731580
H	2.6142538886	0.0852141499	-0.0918289917
H	1.9126848460	1.5758476759	-0.7525771050
C	-0.7502928524	1.0310093274	0.0909523855
H	1.9039780035	1.2901644997	0.9953657907
H	-1.8390339012	-0.4171760709	0.7268569403
C	0.4639285083	0.1253734597	-0.0970255168
H	-0.8076079876	1.7415460476	-0.7364952747
H	-0.6060692520	1.6049632904	1.0172127552
C	1.8077139189	0.8096967263	0.0189974319
C	0.3172179609	-1.1426599960	-0.8415668256
O	0.3396347739	-1.1198206225	0.5916047712
H	1.1930366389	-1.5905851854	-1.2995283071
H	-0.6429411431	-1.3967269015	-1.2762369034

Energy of Optimized Geometry: -307.572727564606

10.2 CAM-B3LYP

OX8 (2-methyloxiran-2-yl)methanol CAM-B3LYP STRUC1

O	-1.9782103038	0.0869892647	-0.0406299557
H	1.7685978451	1.7038114582	-0.7919343509
H	1.6547861477	1.5960001135	0.9661743941
C	-0.8549357055	0.9315862852	-0.0771459414
H	2.5757075923	0.3863485557	0.0684504479
H	-1.8171502567	-0.5683918905	0.6478639102
C	0.4515788560	0.1692849024	-0.0827437970
H	-0.9335873455	1.5368236662	-0.9803165733
H	-0.8515947607	1.6143374903	0.7808311810
C	1.6860802505	1.0114255474	0.0465092799
C	0.5102190422	-1.1562483834	-0.6860338779
O	0.4133059083	-1.0036088445	0.7351388421
H	1.4625941260	-1.5369105361	-1.0353961224
H	-0.3714434801	-1.5681048874	-1.1595433552

Energy of Optimized Geometry: -307.625651733673

OX8 (2-methyloxiran-2-yl)methanol CAM-B3LYP STRUC2

O	-1.7719898288	0.8735737967	0.4623503475
H	2.2024995032	0.7796017509	0.5982502598
H	1.4379310372	1.8121341275	-0.6199768322
C	-1.0299748868	0.3360847498	-0.6150924341
H	0.7587159408	1.7191876528	1.0046775284
H	-1.8063929183	0.2120424289	1.1597986355
C	0.3793232856	-0.0021023981	-0.2039821425
H	-1.5159388073	-0.5580970845	-1.0140934864
H	-1.0244391970	1.0990814409	-1.3930225089
C	1.2497434887	1.1444429580	0.2212665762
C	0.9690051262	-1.2510359714	-0.6763837995
O	0.4369919481	-1.1595934733	0.6414216188
H	2.0480008462	-1.3389291014	-0.7292056814
H	0.4159462451	-1.8931672822	-1.3525065360

Energy of Optimized Geometry: -307.624058991225

OX8 (2-methyloxiran-2-yl)methanol CAM-B3LYP STRUC3

O	-1.8119651728	0.9501545587	-0.5305739612
H	0.7642969655	1.7654616519	-1.0141060487
H	1.3279201383	1.7569710504	0.6564497557
C	-1.0819472749	0.2729940023	0.4771137887
H	2.1982642658	0.8226940991	-0.5644651218
H	-1.9538542289	0.3553993328	-1.2710558441
C	0.3352134688	-0.0350951899	0.0568151176
H	-1.0697053757	0.9422924183	1.3369737595
H	-1.5740322297	-0.6539345732	0.7809828523
C	1.2119727039	1.1456674957	-0.2390303784
C	0.6184871551	-1.3447528152	-0.5305073580
O	0.9370663765	-1.0717554308	0.8296094450
H	1.4722807775	-1.4606764366	-1.1878298325
H	-0.1836319695	-2.0604590704	-0.6775056778

Energy of Optimized Geometry: -307.621935748056

OX8 (2-methyloxiran-2-yl)methanol CAM-B3LYP STRUC5

O	-1.8126580470	0.9296700578	-0.5727061693
H	0.8570549781	1.6960451309	-1.0779797927
H	1.2775965459	1.7775257062	0.6397041004
C	-1.0895009764	0.2679970469	0.4505930240
H	2.2351179374	0.7830475263	-0.4597464610
H	-1.5842532683	1.8610885021	-0.5811220030
C	0.3312871302	-0.0513444601	0.0508565071
H	-1.0811364011	0.8598360747	1.3711429045
H	-1.6158398389	-0.6607060113	0.6597620298
C	1.2270968179	1.1201189997	-0.2296904453
C	0.6194926486	-1.3602093439	-0.5250150335
O	0.9085646892	-1.0866039838	0.8447725379
H	1.4892891296	-1.4847499240	-1.1593777933
H	-0.1883134650	-2.0623623545	-0.6947985058

Energy of Optimized Geometry: -307.621030067619

OX8 (2-methyloxiran-2-yl)methanol CAM-B3LYP STRUC6

O	-1.7292781833	0.9669610806	-0.5503893957
H	2.1990410064	0.7859706170	-0.5657773977
H	0.7754519310	1.7282714483	-1.0475480482
C	-1.0723602191	0.2720694688	0.4977983350
H	1.3244633857	1.7482532807	0.6302156479
H	-2.5912015060	1.2594679341	-0.2481726337
C	0.3272536207	-0.0501674112	0.0471693364
H	-1.0213787607	0.8882117521	1.4015392157
H	-1.5872972845	-0.6587652732	0.7461560062
C	1.2122198550	1.1222958326	-0.2558779536
C	0.5833233506	-1.3562461334	-0.5509740875
O	0.9227688998	-1.0978834743	0.8098505500
H	1.4284192708	-1.4786556615	-1.2180047513
H	-0.2349791705	-2.0514643350	-0.6980325197

Energy of Optimized Geometry: -307.621696326653

OX8 (2-methyloxiran-2-yl)methanol CAM-B3LYP STRUC8

O	-1.7843510860	1.0048949212	0.3607134898
H	2.2192814396	0.8330493821	0.3934392898
H	1.2459291653	1.8147891652	-0.7093080584
C	-1.0722917097	0.2060741650	-0.5653545129
H	0.8006912780	1.6811426582	0.9992623945
H	-1.5010202016	1.9184591055	0.2875033847
C	0.3554058628	-0.0624890464	-0.1598137555
H	-1.6043578778	-0.7413541684	-0.6191579870
H	-1.0843325339	0.6621253374	-1.5612032661
C	1.2054413006	1.1380099178	0.1456106105
C	0.9711124381	-1.3078438917	-0.6114390929
O	0.5303261904	-1.1620864511	0.7322205973
H	2.0460350496	-1.3510871324	-0.7456667406
H	0.4000432741	-2.0098400744	-1.2095318280

Energy of Optimized Geometry: -307.620071521664

OX8 (2-methyloxiran-2-yl)methanol CAM-B3LYP STRUC9

O	-1.6966244724	1.0289856640	0.3652595234
H	1.2165248042	1.8231948910	-0.7157695679
H	0.7067767413	1.7200243028	0.9685122075
C	-1.0734686642	0.1764582969	-0.5801174368
H	2.1737023807	0.8815253124	0.4398544039
H	-2.6290387350	1.1098970543	0.1566766356
C	0.3501343192	-0.0574450980	-0.1592798769
H	-1.5827206566	-0.7882794478	-0.6358981577
H	-1.0802434544	0.6361743837	-1.5749627913
C	1.1639451888	1.1652590181	0.1522176602
C	0.9961544102	-1.2895611374	-0.6027970421
O	0.5350001812	-1.1555163086	0.7350416867
H	2.0734090445	-1.3069419855	-0.7230268664
H	0.4500926082	-2.0044870923	-1.2091158837

Energy of Optimized Geometry: -307.620835731757

OX8 (2-methyloxiran-2-yl)methanol CAM-B3LYP STRUC10

O	-1.9738210894	0.1378484374	0.0882082626
H	2.5851525498	0.3164837208	-0.0611799992
H	1.7680156776	1.7109162535	-0.7787552883
C	-0.8307064127	0.9551367809	0.0459992988
H	1.7549442612	1.4831283181	0.9715149849
H	-1.7933448991	-0.5701901678	0.7173698027
C	0.4505222522	0.1638674515	-0.0978855544
H	-0.9462005335	1.6295716941	-0.8026878205
H	-0.7556166735	1.5697944758	0.9511327941
C	1.7146289475	0.9638657857	0.0137831328
C	0.4333465179	-1.1138723713	-0.7989309808
O	0.4285088079	-1.0652940154	0.6331244765
H	1.3509004296	-1.4909401132	-1.2347241978
H	-0.4874117150	-1.4672188021	-1.2443226293

Energy of Optimized Geometry: -307.625651898715

10.3 M06-2X

OX8 (2-methyloxiran-2-yl)methanol M06-2X STRUC1

O	-1.9742140305	0.0913891088	-0.0252927596
H	1.7631277304	1.7049917982	-0.7970010365
H	1.6764135698	1.5773577325	0.9637810783
C	-0.8533591625	0.9375428138	-0.0572824359
H	2.5785156605	0.3711474356	0.0368623154
H	-1.8061055283	-0.5708619351	0.6549703784
C	0.4513660966	0.1695758220	-0.0829919243
H	-0.9336358356	1.5553180871	-0.9522736793
H	-0.8397968806	1.6064944098	0.8120965424
C	1.6937170405	1.0044300992	0.0359042279
C	0.4916374898	-1.1575173758	-0.6970375969
O	0.4173862399	-1.0054523507	0.7210362218
H	1.4380251613	-1.5398897189	-1.0604182899
H	-0.4027365154	-1.5571987973	-1.1577464047

Energy of Optimized Geometry: -307.638738700448

OX8 (2-methyloxiran-2-yl)methanol M06-2X STRUC2

O	-1.7615223087	0.8682196955	0.4595169782
H	2.2001245884	0.7839573747	0.5914175846
H	1.4465867675	1.8001070207	-0.6517374516
C	-1.0303930080	0.3347676406	-0.6253843546
H	0.7535148833	1.7360740426	0.9716088371
H	-1.7563808953	0.2122871246	1.1637367860
C	0.3792941679	-0.0028923744	-0.2099282855
H	-1.5160574373	-0.5619242558	-1.0212966711
H	-1.0245054615	1.0991663610	-1.4024013448
C	1.2522067494	1.1491117757	0.2010493682
C	0.9661970771	-1.2696012134	-0.6524654739
O	0.4244354392	-1.1394002707	0.6544027803
H	2.0453741093	-1.3619862022	-0.6919108006
H	0.4102503956	-1.9207837917	-1.3172731195

Energy of Optimized Geometry: -307.636842222599

OX8 (2-methyloxiran-2-yl)methanol M06-2X STRUC3

O	-1.8059470719	0.9378729658	-0.5302944768
H	0.7524374789	1.7765530114	-0.9962774620
H	1.3391700155	1.7521184473	0.6690494991
C	-1.0814057400	0.2779033077	0.4896015885
H	2.1928877791	0.8250054761	-0.5730589623
H	-1.8803858598	0.3488129380	-1.2856815196
C	0.3350816266	-0.0310931228	0.0658273436
H	-1.0694884433	0.9594486496	1.3408127000
H	-1.5687967625	-0.6495536168	0.8032655216
C	1.2121540928	1.1510042962	-0.2323432822
C	0.6056120469	-1.3414936709	-0.5398352882
O	0.9382204723	-1.0753250570	0.8144163545
H	1.4525278616	-1.4516506397	-1.2068221176
H	-0.2043778931	-2.0498720711	-0.6797072294

Energy of Optimized Geometry: -307.634629920232

OX8 (2-methyloxiran-2-yl)methanol M06-2X STRUC5

O	-1.7994563231	0.9528169794	-0.5592468351
H	0.8505299090	1.6799743507	-1.0924284009
H	1.2370707552	1.7920792417	0.6347171690
C	-1.0959977262	0.2552368758	0.4504016308
H	2.2298817614	0.7922133506	-0.4332223612
H	-1.5185505196	1.8701813005	-0.5666649049
C	0.3271999327	-0.0577495434	0.0517037494
H	-1.0875346917	0.8198035300	1.3890641317
H	-1.6296756230	-0.6771852724	0.6266319852
C	1.2138486221	1.1233597593	-0.2280019714
C	0.6259079675	-1.3665220633	-0.5318333274
O	0.9076970784	-1.0903804207	0.8350397585
H	1.4991160408	-1.4790569833	-1.1637332843
H	-0.1796941981	-2.0709338159	-0.7026521806

Energy of Optimized Geometry: -307.633545614441

OX8 (2-methyloxiran-2-yl)methanol M06-2X STRUC6

O	-1.7309399366	0.9351511595	-0.5719862766
H	2.1869436660	0.8163860968	-0.5565477220
H	0.7459822480	1.7262014585	-1.0614458114
C	-1.0823020655	0.2597116411	0.4910745261
H	1.2834824187	1.7787416235	0.6222900395
H	-2.5988478511	1.2227336581	-0.2824944843
C	0.3267120790	-0.0451555526	0.0542520893
H	-1.0462507798	0.8854249991	1.3897539668
H	-1.5853849941	-0.6779053159	0.7421058319
C	1.1920169635	1.1413867100	-0.2586435980
C	0.6061374927	-1.3566369923	-0.5336661937
O	0.9336871172	-1.0745165623	0.8212984252
H	1.4565955949	-1.4677405135	-1.1957619979
H	-0.2031760403	-2.0637462829	-0.6738781734

Energy of Optimized Geometry: -307.634053043890

OX8 (2-methyloxiran-2-yl)methanol M06-2X STRUC8

O	-1.7716555875	1.0162550073	0.3621543818
H	2.2208747010	0.8366475897	0.3661149642
H	1.2112951317	1.8276880067	-0.7005565946
C	-1.0771289460	0.2039996498	-0.5630753170
H	0.8052764509	1.6606193003	1.0181062306
H	-1.4424870710	1.9159995441	0.3060042806
C	0.3517711289	-0.0663599729	-0.1598222864
H	-1.6137241227	-0.7426208625	-0.6039382185
H	-1.0891476843	0.6524572293	-1.5627997924
C	1.1991089296	1.1382927805	0.1454726302
C	0.9694083728	-1.3160940131	-0.6120823157
O	0.5295292018	-1.1611578019	0.7273892007
H	2.0444746305	-1.3556393306	-0.7451880374
H	0.3934323393	-2.0172422108	-1.2063306426

Energy of Optimized Geometry: -307.632808176996

OX8 (2-methyloxiran-2-yl)methanol M06-2X STRUC9

O	-1.6800523792	1.0398113946	0.3741127246
H	1.2165102845	1.8187009912	-0.7131255292
H	0.7145711893	1.7076143613	0.9753957809
C	-1.0746050947	0.1867740140	-0.5800205975
H	2.1793716609	0.8666833821	0.4342487060
H	-2.6099821926	1.1420099373	0.1637806018
C	0.3483408072	-0.0607802983	-0.1600506049
H	-1.5915682502	-0.7747399253	-0.6382996381
H	-1.0766404166	0.6528390231	-1.5719631322
C	1.1695138627	1.1584627995	0.1533419556
C	0.9864076045	-1.3021649396	-0.6054477175
O	0.5238454954	-1.1574147381	0.7271311831
H	2.0639026637	-1.3251863655	-0.7217895814
H	0.4309942287	-2.0106198261	-1.2107403015

Energy of Optimized Geometry: -307.633299603279

OX8 (2-methyloxiran-2-yl)methanol M06-2X STRUC10

O	-1.9687452471	0.1603240303	0.0913755305
H	2.5888067592	0.2780211054	-0.0664809650
H	1.7858607118	1.7082546077	-0.7365682302
C	-0.8182661289	0.9662637075	0.0811925006
H	1.7822869910	1.4276854893	1.0086099043
H	-1.7957396649	-0.5689980477	0.6979154237
C	0.4524558683	0.1611895179	-0.0907454314
H	-0.9194769903	1.6723978509	-0.7437393778
H	-0.7340542685	1.5446622084	1.0095702314
C	1.7306207571	0.9392566692	0.0347798941
C	0.4095878062	-1.1008135559	-0.8290136628
O	0.4166566357	-1.0841043463	0.5989261751
H	1.3200754294	-1.4769991649	-1.2800506645
H	-0.5224429905	-1.4247222936	-1.2742948004

Energy of Optimized Geometry: -307.638739231344

10.4 MN15

OX8 (2-methyloxiran-2-yl)methanol MN15 STRUC1

O	-1.9638605340	0.0986949917	-0.0096517537
H	1.7945856583	1.6632825318	-0.8288325310
H	1.6851617364	1.5899603063	0.9331985196
C	-0.8472802430	0.9452198739	-0.0592189487
H	2.5758900556	0.3399562972	0.0529740814
H	-1.7749559469	-0.5784172876	0.6550620012
C	0.4511058417	0.1692057987	-0.0861088935
H	-0.9293322289	1.5587849994	-0.9570866409
H	-0.8214870484	1.6223284515	0.8044545457
C	1.7026593316	0.9890155391	0.0232545342
C	0.4755974516	-1.1593377059	-0.6946933875
O	0.4048614432	-1.0020813922	0.7205333036
H	1.4174078486	-1.5516425918	-1.0609149541
H	-0.4237727895	-1.5481336633	-1.1559599754

Energy of Optimized Geometry: -307.415373474992

OX8 (2-methyloxiran-2-yl)methanol MN15 STRUC2

O	-1.7631049153	0.8090608977	0.5030999675
H	2.1944283431	0.7998083965	0.5867898141
H	1.4326490301	1.8315723105	-0.6385006621
C	-1.0400957791	0.3369033441	-0.6116236472
H	0.7427549430	1.7379437481	0.9847216121
H	-1.7128561891	0.1361826816	1.1930943660
C	0.3789479453	0.0143956182	-0.2197203686
H	-1.5051681019	-0.5600167068	-1.0339052806
H	-1.0631889299	1.1258134335	-1.3633682366
C	1.2433292295	1.1659978436	0.2041130626
C	0.9712255525	-1.2412544730	-0.6813067816
O	0.4365852393	-1.1335440638	0.6272336594
H	2.0512761825	-1.3244818920	-0.7308165995
H	0.4167842034	-1.8838274208	-1.3566953008

Energy of Optimized Geometry: -307.412999014137

OX8 (2-methyloxiran-2-yl)methanol MN15 STRUC3

O	-1.8039276864	0.9334273011	-0.5265027695
H	0.7530115685	1.7731123159	-1.0023434273
H	1.3507175169	1.7544569962	0.6584667172
C	-1.0783057009	0.2806818208	0.4941247968
H	2.1917291415	0.8162413562	-0.5851367869
H	-1.8763080975	0.3479586115	-1.2877796804
C	0.3366535290	-0.0281893616	0.0682145373
H	-1.0673919264	0.9610180039	1.3461612841
H	-1.5571642459	-0.6513895976	0.8106901118
C	1.2149412015	1.1488918516	-0.2384527544
C	0.5964044618	-1.3386628648	-0.5388373472
O	0.9377317683	-1.0755695962	0.8107382363
H	1.4368891394	-1.4525089173	-1.2141086079
H	-0.2209824398	-2.0398009085	-0.6775907696

Energy of Optimized Geometry: -307.410591322939

OX8 (2-methyloxiran-2-yl)methanol MN15 STRUC5

O	-1.7960134752	0.9301407519	-0.5989266352
H	0.8498848675	1.6419385554	-1.1476655574
H	1.2355061052	1.8150743984	0.5741473432
C	-1.0985616463	0.2672419495	0.4347407012
H	2.2279244798	0.7758331742	-0.4575282659
H	-1.5093179749	1.8468930049	-0.6503218487
C	0.3272816514	-0.0546637354	0.0563680207
H	-1.0977406738	0.8594589130	1.3566568340
H	-1.6250472756	-0.6641955319	0.6386708566
C	1.2121553734	1.1155908193	-0.2637209017
C	0.6295374206	-1.3822595125	-0.4779650571
O	0.9041753074	-1.0577205448	0.8765023386
H	1.5042637080	-1.5152108445	-1.1046939692
H	-0.1765761423	-2.0909617084	-0.6310130034

Energy of Optimized Geometry: -307.409660208319

OX8 (2-methyloxiran-2-yl)methanol MN15 STRUC6

O	-1.7313797339	0.9405059739	-0.5590709349
H	2.1860891621	0.8121317697	-0.5597233729
H	0.7468143197	1.7274986148	-1.0597374370
C	-1.0809667248	0.2548242644	0.4922482942
H	1.2881627923	1.7776204547	0.6220706345
H	-2.5978687086	1.2402814770	-0.2706584000
C	0.3279035262	-0.0446225171	0.0534492879
H	-1.0452603486	0.8670251294	1.4004888094
H	-1.5758768460	-0.6908884115	0.7332953774
C	1.1928473961	1.1404979425	-0.2585156006
C	0.6042296653	-1.3532729395	-0.5397693788
O	0.9326401958	-1.0781918664	0.8132178216
H	1.4524389382	-1.4620835434	-1.2059882502
H	-0.2088174178	-2.0557712188	-0.6857280166

Energy of Optimized Geometry: -307.410110705165

OX8 (2-methyloxiran-2-yl)methanol MN15 STRUC8

O	-1.7666289166	1.0313051785	0.3487256648
H	2.2204158207	0.8253026715	0.3695775735
H	1.2159002203	1.8221043181	-0.6971861878
C	-1.0770380085	0.2018102769	-0.5605795997
H	0.8086331570	1.6579278305	1.0207586831
H	-1.4238071654	1.9288710719	0.2964624656
C	0.3501693725	-0.0689857831	-0.1546928876
H	-1.6112228074	-0.7472003483	-0.5883047898
H	-1.0872302748	0.6315148482	-1.5691729234
C	1.1999730160	1.1325481657	0.1487884538
C	0.9636884388	-1.3185361732	-0.6083690302
O	0.5282743654	-1.1643393561	0.7293921311
H	2.0385001375	-1.3586003316	-0.7481051940
H	0.3847503429	-2.0155622727	-1.2057672624

Energy of Optimized Geometry: -307.408987835659

OX8 (2-methyloxiran-2-yl)methanol MN15 STRUC9

O	-1.6822560118	1.0417856383	0.3712489861
H	1.2090482086	1.8292262440	-0.6914873673
H	0.7220141263	1.6944294237	0.9991149117
C	-1.0757296066	0.1846862187	-0.5736033576
H	2.1813002587	0.8585761913	0.4330289347
H	-2.6100502767	1.1682296430	0.1547447542
C	0.3477756628	-0.0592943400	-0.1556987718
H	-1.5838195313	-0.7830762018	-0.6248144003
H	-1.0799248199	0.6390319792	-1.5715452750
C	1.1688328329	1.1560150640	0.1655702189
C	0.9847080921	-1.2948472955	-0.6144600188
O	0.5291010451	-1.1638577212	0.7186504084
H	2.0618015172	-1.3136425404	-0.7401086545
H	0.4268167018	-1.9953702197	-1.2278977557

Energy of Optimized Geometry: -307.409439099203

OX8 (2-methyloxiran-2-yl)methanol MN15 STRUC10

O	-1.9596513037	0.0621707075	0.1372685701
H	2.5710621908	0.3776895231	-0.0738821320
H	1.7162275852	1.8120582104	-0.6662349942
C	-0.8573173465	0.9282579552	0.1375897908
H	1.7356110083	1.4462649070	1.0622380997
H	-1.7140376427	-0.7055263462	0.6726321962
C	0.4429249321	0.1889096691	-0.0894006811
H	-1.0062499379	1.6640126509	-0.6535477161
H	-0.7781229270	1.4730938076	1.0873446774
C	1.6922836875	1.0044335900	0.0661234676
C	0.4380278853	-1.0309327231	-0.8944399827
O	0.4583891911	-1.0921667229	0.5298351395
H	1.3569269852	-1.3462723183	-1.3752114151
H	-0.4864535498	-1.3609390966	-1.3519153247

Energy of Optimized Geometry: -307.415376136601

10.5 ωB97X-D

OX8 (2-methyloxiran-2-yl)methanol ωB97X-D STRUC1

O	-1.9836572987	0.1003642433	-0.0485921571
H	1.7620499832	1.7044477792	-0.7884099793
H	1.6592413926	1.5909026226	0.9716487352
C	-0.8546984569	0.9331891859	-0.0700557464
H	2.5809934089	0.3865944570	0.0635479150
H	-1.8365326089	-0.5522711453	0.6416770222
C	0.4524316112	0.1641222578	-0.0774602906
H	-0.9208422649	1.5473030870	-0.9692338765
H	-0.8482552644	1.6100507908	0.7944096291
C	1.6885650234	1.0092998165	0.0492453334
C	0.5092341020	-1.1600200764	-0.6864902447
O	0.4170939144	-1.0108313658	0.7311730540
H	1.4615085339	-1.5385918182	-1.0419097646
H	-0.3747606967	-1.5695608897	-1.1607696833

Energy of Optimized Geometry: -307.667510104561

OX8 (2-methyloxiran-2-yl)methanol ωB97X-D STRUC2

O	-1.7762916535	0.8960049315	0.4253252324
H	2.2037149155	0.7925285084	0.5942391407
H	1.4339818078	1.7985794985	-0.6451245675
C	-1.0286643214	0.3239074884	-0.6264218998
H	0.7545714042	1.7349745724	0.9825089468
H	-1.8093665787	0.2575388638	1.1402098382
C	0.3799958411	-0.0111125257	-0.1961807165
H	-1.5159742465	-0.5794047022	-1.0071242322
H	-1.0079970144	1.0638242700	-1.4271702906
C	1.2490691513	1.1471018890	0.2100098935
C	0.9728972220	-1.2689055732	-0.6434529569
O	0.4361984886	-1.1520811792	0.6661087401
H	2.0534503173	-1.3549635611	-0.6914234517
H	0.4228819230	-1.9230620016	-1.3124438327

Energy of Optimized Geometry: -307.666001035859

OX8 (2-methyloxiran-2-yl)methanol ωB97X-D STRUC3

O	-1.8125597449	0.9549303731	-0.5043177407
H	0.7651838992	1.7838464538	-0.9813621166
H	1.3526538876	1.7396060322	0.6821338562
C	-1.0775310827	0.2794052161	0.4962951468
H	2.2014145784	0.8243941846	-0.5710050176
H	-1.9146135775	0.3697194574	-1.2557865650
C	0.3377247552	-0.0351244911	0.0607101088
H	-1.0544778807	0.9497251676	1.3562307163
H	-1.5687978711	-0.6478188207	0.8070122681
C	1.2213377879	1.1468534044	-0.2243171829
C	0.5995083322	-1.3346722844	-0.5613408472
O	0.9378612177	-1.0942424706	0.7963284392
H	1.4429426890	-1.4426562032	-1.2349295771
H	-0.2139609564	-2.0381137052	-0.7141858405

Energy of Optimized Geometry: -307.664045798500

OX8 (2-methyloxiran-2-yl)methanol ωB97X-D STRUC5

O	-1.8112788659	0.9359006873	-0.5658503883
H	0.8556543571	1.6941258628	-1.0769867821
H	1.2656460385	1.7831960734	0.6447372241
C	-1.0932638611	0.2677300454	0.4521844314
H	2.2375245435	0.7918006857	-0.4487278781
H	-1.5448589360	1.8544583094	-0.5885934252
C	0.3316064587	-0.0543021736	0.0536039026
H	-1.0820571186	0.8536223311	1.3783928545
H	-1.6238345041	-0.6609897497	0.6560315992
C	1.2255755595	1.1232868881	-0.2240811473
C	0.6206349456	-1.3599974338	-0.5315834349
O	0.9087591238	-1.0933716502	0.8359163050
H	1.4903546302	-1.4786133778	-1.1690905234
H	-0.1883737532	-2.0611988712	-0.7066714696

Energy of Optimized Geometry: -307.663083166474

OX8 (2-methyloxiran-2-yl)methanol ωB97X-D STRUC6

O	-1.7305964447	0.9660437374	-0.5431226287
H	2.2053915065	0.7847726629	-0.5652292506
H	0.7813915922	1.7331291477	-1.0381501633
C	-1.0711791281	0.2797594139	0.5049562517
H	1.3350251320	1.7431056636	0.6400019169
H	-2.5780616625	1.2765955964	-0.2270453205
C	0.3292237348	-0.0505417360	0.0497703879
H	-1.0114888913	0.9022623088	1.4055081157
H	-1.5868784166	-0.6493127904	0.7654633980
C	1.2197784381	1.1223490696	-0.2500822742
C	0.5748511273	-1.3541046477	-0.5609482208
O	0.9212234369	-1.1073727698	0.7962417865
H	1.4158231131	-1.4744030338	-1.2353268912
H	-0.2498764188	-2.0429369088	-0.7106404265

Energy of Optimized Geometry: -307.663290914294

OX8 (2-methyloxiran-2-yl)methanol ωB97X-D STRUC8

O	-1.7913551406	0.9923175455	0.3744706626
H	2.2217000713	0.8531645875	0.3729883796
H	1.2107447404	1.8349473334	-0.6982622434
C	-1.0791243625	0.2058272175	-0.5573735382
H	0.8017370253	1.6725673562	1.0187167063
H	-1.4887352442	1.8984861859	0.3203256175
C	0.3548457498	-0.0615773087	-0.1584415983
H	-1.6085139180	-0.7437054009	-0.6209875394
H	-1.0902552034	0.6694130790	-1.5513440754
C	1.1981405819	1.1468220497	0.1491584000
C	0.9774363195	-1.2991030763	-0.6253050396
O	0.5446082901	-1.1660841426	0.7185170913
H	2.0527879982	-1.3317140510	-0.7678444494
H	0.4069516957	-1.9997319513	-1.2276165453

Energy of Optimized Geometry: -307.662300163867

OX8 (2-methyloxiran-2-yl)methanol ωB97X-D STRUC9

O	-1.6971353557	1.0318097916	0.3660999027
H	1.2172776626	1.8217898516	-0.7088975974
H	0.7238923073	1.7127457570	0.9816843784
C	-1.0729564573	0.1875617998	-0.5813985850
H	2.1854121567	0.8735477417	0.4345563967
H	-2.6138881239	1.1502880240	0.1215217209
C	0.3504327434	-0.0596891089	-0.1547364417
H	-1.5879354348	-0.7749543335	-0.6544090377
H	-1.0649804274	0.6566995030	-1.5732518562
C	1.1726887235	1.1607556346	0.1578362674
C	0.9907987969	-1.2943789563	-0.6033356909
O	0.5297729542	-1.1611592771	0.7307131067
H	2.0690682969	-1.3151588177	-0.7241583737
H	0.4407328250	-2.0036157492	-1.2147450613

Energy of Optimized Geometry: -307.662492812337

OX8 (2-methyloxiran-2-yl)methanol ωB97X-D STRUC10

O	-1.9826873829	0.1239007206	0.0783802669
H	2.5859606508	0.3497531933	-0.0183225497
H	1.7474013626	1.7798728794	-0.6388325851
C	-0.8415918460	0.9389698181	0.1222711619
H	1.7327985735	1.4277587778	1.0925469093
H	-1.8129954554	-0.6271822864	0.6536743908
C	0.4500144778	0.1694422947	-0.0769246776
H	-0.9457715069	1.6860975597	-0.6656774537
H	-0.7759498998	1.4732531579	1.0792301150
C	1.7048956018	0.9776387050	0.0992821704
C	0.4512680764	-1.0485008116	-0.8792830798
O	0.4418964677	-1.1126017544	0.5481276140
H	1.3759975789	-1.3762389516	-1.3416939180
H	-0.4645982460	-1.3758416167	-1.3566552431

Energy of Optimized Geometry: -307.667509479195

10.6 EOM-CCSD

OX8 (2-methyloxiran-2-yl)methanol EOM-CCSD STRUC1

O	-1.9839821376	0.0767701702	-0.0501936441
H	1.7730456592	1.7155268041	-0.7888794886
H	1.6615326948	1.5970720526	0.9758207481
C	-0.8601575715	0.9364990091	-0.0708339743
H	2.5817245887	0.3856579115	0.0662425129
H	-1.7994186721	-0.5777054312	0.6362950664
C	0.4523489081	0.1750843473	-0.0831090540
H	-0.9401232615	1.5519746448	-0.9697762243
H	-0.8608876987	1.6059806936	0.8002414063
C	1.6935399611	1.0181039776	0.0494247059
C	0.5098621445	-1.1560464874	-0.6958938094
O	0.4160795042	-1.0073874625	0.7443196830
H	1.4654900232	-1.5349595772	-1.0427292848
H	-0.3774342452	-1.5669500978	-1.1631285578

Energy of Optimized Geometry: -307.173344320079

OX8 (2-methyloxiran-2-yl)methanol EOM-CCSD STRUC2

O	-1.7779647583	0.8513705107	0.4856159096
H	2.2113037125	0.7983596403	0.5675654686
H	1.4150692275	1.8393875448	-0.6333977914
C	-1.0415571862	0.3298077230	-0.6150380900
H	0.7597003488	1.7235000781	1.0075754467
H	-1.7680693645	0.1707840194	1.1687146497
C	0.3792927470	0.0063170495	-0.2150245931
H	-1.5192723689	-0.5719891831	-1.0136824685
H	-1.0545765678	1.1034260066	-1.3856320682
C	1.2479551171	1.1625865132	0.2087129174
C	0.9787142625	-1.2482987697	-0.6887034499
O	0.4451788391	-1.1550104843	0.6474921024
H	2.0598614376	-1.3286757675	-0.7391171391
H	0.4211616737	-1.8974245407	-1.3566933194

Energy of Optimized Geometry: -307.171718277838

OX8 (2-methyloxiran-2-yl)methanol EOM-CCSD STRUC3

O	-1.8130105006	0.9504017523	-0.5520034647
H	0.7770082694	1.7615580865	-1.0295942276
H	1.3247217627	1.7691987082	0.6538902205
C	-1.0893636743	0.2752000533	0.4744215577
H	2.2086171750	0.8154460947	-0.5520135042
H	-1.9138286337	0.3386666002	-1.2885360007
C	0.3337343319	-0.0320074652	0.0595425403
H	-1.0847434825	0.9527687392	1.3307693579
H	-1.5815003691	-0.6554721169	0.7760570589
C	1.2182811189	1.1497408830	-0.2404904963
C	0.6170226618	-1.3520847335	-0.5265954414
O	0.9388697512	-1.0720409205	0.8499826044
H	1.4765139407	-1.4707189891	-1.1783446731
H	-0.1890391913	-2.0673241732	-0.6656316077

Energy of Optimized Geometry: -307.169567615919

OX8 (2-methyloxiran-2-yl)methanol EOM-CCSD STRUC5

O	-1.8144137794	0.9397131637	-0.5831763928
H	0.8663868205	1.6906353954	-1.0935321338
H	1.2719528805	1.7874463001	0.6340821402
C	-1.0961442030	0.2670760048	0.4492714896
H	2.2423795580	0.7771374944	-0.4507549772
H	-1.5440227569	1.8624335161	-0.5855792601
C	0.3291673990	-0.0513700341	0.0499345708
H	-1.0898247065	0.8565231607	1.3741671798
H	-1.6264545058	-0.6649108834	0.6466002030
C	1.2310862423	1.1226652773	-0.2337483654
C	0.6201603613	-1.3692846074	-0.5257360711
O	0.9110541566	-1.0893246693	0.8614544672
H	1.4962041111	-1.4938991553	-1.1539071626
H	-0.1898633870	-2.0728425888	-0.6884609385

Energy of Optimized Geometry: -307.168617103616

OX8 (2-methyloxiran-2-yl)methanol EOM-CCSD STRUC6

O	-1.7247128068	0.9655485654	-0.5829232274
H	2.2089840225	0.7740613114	-0.5569718818
H	0.7883630974	1.7150719024	-1.0755922977
C	-1.0812036781	0.2779097875	0.4906464357
H	1.3190390715	1.7641550906	0.6148851246
H	-2.5916292090	1.2485072934	-0.2784224336
C	0.3250497856	-0.0479422036	0.0496569566
H	-1.0377756456	0.9067742186	1.3886394646
H	-1.5987526230	-0.6537477739	0.7412179180
C	1.2177583808	1.1233429447	-0.2652860281
C	0.5838996159	-1.3696006457	-0.5340949707
O	0.9221578990	-1.0911863890	0.8420000392
H	1.4372083639	-1.5003113401	-1.1913309788
H	-0.2369465478	-2.0665836344	-0.6693692489

Energy of Optimized Geometry: -307.169170669916

OX8 (2-methyloxiran-2-yl)methanol EOM-CCSD STRUC8

O	-1.7964446200	0.9763107258	0.3980261734
H	2.2249698983	0.8529792896	0.3640355256
H	1.2169364608	1.8466915850	-0.7082908843
C	-1.0826537053	0.2035185597	-0.5619952772
H	0.8039718655	1.6799763828	1.0124837500
H	-1.4962697011	1.8878215802	0.3350460649
C	0.3555046898	-0.0535786032	-0.1703497120
H	-1.6036913524	-0.7521220264	-0.6288638218
H	-1.1083469049	0.6850413538	-1.5482187745
C	1.2017977429	1.1559631228	0.1401385894
C	0.9830157788	-1.2976901084	-0.6388999161
O	0.5449717188	-1.1632536136	0.7260661312
H	2.0595513233	-1.3293117461	-0.7742914641
H	0.4084999684	-2.0019475652	-1.2334890067

Energy of Optimized Geometry: -307.167635199650

OX8 (2-methyloxiran-2-yl)methanol EOM-CCSD STRUC9

O	-1.6837331998	1.0517749797	0.3632809326
H	1.2393322729	1.8122773779	-0.7312042740
H	0.7388535137	1.7167730466	0.9641200086
C	-1.0728992963	0.1884812316	-0.5938178090
H	2.1951882799	0.8538589892	0.4226603179
H	-2.6111990329	1.1474028312	0.1293393051
C	0.3501953469	-0.0605068020	-0.1641937740
H	-1.5931061420	-0.7733195128	-0.6499107516
H	-1.0711062587	0.6536788957	-1.5885541605
C	1.1856283625	1.1564844777	0.1420878641
C	0.9835953515	-1.3145401109	-0.5947182138
O	0.5112047982	-1.1573402806	0.7559411295
H	2.0631831684	-1.3485676328	-0.7019771346
H	0.4242677883	-2.0285343235	-1.1924335470

Energy of Optimized Geometry: -307.168262911418

OX8 (2-methyloxiran-2-yl)methanol EOM-CCSD STRUC10

O	-1.9841441970	0.0609300800	0.0646128138
H	2.5777571782	0.4062079028	-0.0924409017
H	1.7090756256	1.7129553328	-0.9240375208
C	-0.8708643879	0.9284603314	-0.0380470713
H	1.7018649626	1.6283123644	0.8461101220
H	-1.7543165633	-0.5785950731	0.7515246469
C	0.4451929746	0.1768039344	-0.1127631231
H	-1.0084886267	1.5257761864	-0.9422454534
H	-0.8264730949	1.6146825082	0.8187532778
C	1.6846886447	1.0315377952	-0.0693159920
C	0.4782654073	-1.1655503375	-0.7024326960
O	0.4675844700	-0.9895433453	0.7377108128
H	1.4152116886	-1.5439677727	-1.0974354911
H	-0.4312545167	-1.5921329312	-1.1089363006

Energy of Optimized Geometry: -307.173344320505

11 3,4-epoxy-1-butene (OX9)

11.1 BMK

OX9 3,4-epoxy-1-butene BMK STRUC1

C	-2.2316492048	-0.0243627301	0.1140402118
C	1.4649966817	-0.6881746761	-0.1010817238
C	-1.0074606671	0.0019673019	-0.4001009789
O	1.3292617086	0.7190767803	-0.0384204333
H	-3.1105763508	0.0053935069	-0.5161313539
H	-2.3911966711	-0.0791723498	1.1851598887
C	0.2238637436	-0.0572766236	0.4222950433
H	0.0820266335	-0.0361572366	1.4995040626
H	-0.8520683925	0.0697588405	-1.4727156934
H	1.4590530452	-1.1096669445	-1.1016325321
H	2.1749683768	-1.1198066946	0.5970203675

Energy of Optimized Geometry: -231.119280027690

OX9 3,4-epoxy-1-butene BMK STRUC2

C	-1.8004767237	0.2682513943	-0.5212655276
C	1.3468280148	-0.6712047905	-0.2683554550
C	-1.1162394078	-0.2842776629	0.4730930991
O	1.0302364401	0.7102272164	-0.2687324156
H	-2.8624126573	0.0952775670	-0.6343686847
H	-1.3071591029	0.9109636282	-1.2402653257
C	0.3469608691	-0.0918814914	0.6752683054
H	0.6689112158	0.0587567790	1.7028761951
H	-1.6107711165	-0.9149460292	1.2051363565
H	0.9793551029	-1.2268375648	-1.1251939240
H	2.3426618596	-0.9182760386	0.0853330896

Energy of Optimized Geometry: -231.118480185742

OX9 3,4-epoxy-1-butene BMK STRUC3

C	-1.9599831364	-0.3734390444	-0.3123195340
C	1.1347716549	-0.8506326623	-0.0582860098
C	-1.0729115288	0.5216194688	0.1025731091
O	1.3827011806	0.5340081279	-0.2662196772
H	-2.9460567185	-0.0764942650	-0.6462472212
H	-1.7295944706	-1.4324338921	-0.3279084537
C	0.2968562599	0.1818903796	0.5906009063
H	0.4916638458	0.4060463434	1.6372141233
H	-1.3280265587	1.5771308584	0.1195767438
H	0.7444417300	-1.3801877718	-0.9209874359
H	1.8891099076	-1.3709169166	0.5226841803

Energy of Optimized Geometry: -231.115047879342

11.2 CAM-B3LYP

OX9 3,4-epoxy-1-butene CAM-B3LYP STRUC1

C	-2.2183973725	-0.0095547285	0.1191820651
C	1.4490514757	-0.6875983483	-0.0991402945
C	-1.0015759713	-0.0073115005	-0.3965039415
O	1.3255993754	0.7227837091	-0.0400775046
H	-3.0982290254	0.0241798188	-0.5064884829
H	-2.3771544003	-0.0475226898	1.1892620785
C	0.2256981567	-0.0689999210	0.4130059498
H	0.0819482852	-0.0497460070	1.4884670912
H	-0.8540145505	0.0438486402	-1.4692716902
H	1.4508426797	-1.1098845804	-1.0976742913
H	2.1570969694	-1.1224678825	0.5966438627

Energy of Optimized Geometry: -231.159437577553

OX9 3,4-epoxy-1-butene CAM-B3LYP STRUC2

C	-1.8047250020	0.2769287778	-0.5053278236
C	1.3398322674	-0.6750823169	-0.2467460311
C	-1.1064634332	-0.2881160430	0.4630587408
O	1.0331666479	0.7107327857	-0.2833402712
H	-2.8649903248	0.0985949990	-0.6087790553
H	-1.3299722888	0.9375700141	-1.2172640530
C	0.3460630166	-0.0936566458	0.6608464150
H	0.6568670301	0.0714990022	1.6884244490
H	-1.5898378648	-0.9362127080	1.1854902937
H	0.9908840859	-1.2447076023	-1.0997649128
H	2.3293005475	-0.9201407986	0.1213773962

Energy of Optimized Geometry: -231.158172489148

OX9 3,4-epoxy-1-butene CAM-B3LYP STRUC3

C	-1.9474442498	-0.4404123438	-0.2781437302
C	1.1542917291	-0.8032349952	-0.0667570086
C	-1.0808121753	0.4751247321	0.1147749814
O	1.3596954013	0.5872001996	-0.2878094078
H	-2.9465850752	-0.1727083342	-0.5911085512
H	-1.6915201748	-1.4914709656	-0.2986099855
C	0.2970009483	0.1853169471	0.5760661673
H	0.4919796609	0.4169744076	1.6197318297
H	-1.3670995330	1.5209330808	0.1338543818
H	0.7810836185	-1.3520505784	-0.9226061741
H	1.9294259612	-1.2968410192	0.5074294632

Energy of Optimized Geometry: -231.155294882249

11.3 M06-2X

OX9 3,4-epoxy-1-butene M06-2X STRUC1

C	-2.2202310947	-0.0196390749	0.1138205569
C	1.4469831397	-0.6899020563	-0.1010973447
C	-0.9994135557	0.0042126371	-0.3983242169
O	1.3275002905	0.7165997235	-0.0366524057
H	-3.0990917288	0.0117146570	-0.5140415412
H	-2.3776256566	-0.0740536221	1.1840931450
C	0.2256730401	-0.0590892896	0.4187794466
H	0.0825547734	-0.0419154895	1.4943098029
H	-0.8427012408	0.0714127333	-1.4698134815
H	1.4347294402	-1.1091789293	-1.1006802269
H	2.1534681551	-1.1291453903	0.5927825560

Energy of Optimized Geometry: -231.176737082077

OX9 3,4-epoxy-1-butene M06-2X STRUC2

C	-1.7872531207	0.2678005062	-0.5170984761
C	1.3294502771	-0.6678184693	-0.2656689527
C	-1.1057263070	-0.2881510217	0.4722730811
O	1.0236898932	0.7140127688	-0.2704093800
H	-2.8468463226	0.0921994312	-0.6357376851
H	-1.2938458213	0.9171064504	-1.2283628838
C	0.3491009859	-0.0950075278	0.6714812454
H	0.6738893333	0.0526104641	1.6967230284
H	-1.5986959302	-0.9266750701	1.1968153728
H	0.9545197080	-1.2223195116	-1.1178001438
H	2.3242656599	-0.9196739867	0.0817462569

Energy of Optimized Geometry: -231.176230116696

OX9 3,4-epoxy-1-butene M06-2X STRUC3

C	-1.9340286893	-0.4486411539	-0.2896496960
C	1.1487148445	-0.8010123860	-0.0753579927
C	-1.0825995911	0.4780429333	0.1189994769
O	1.3538689247	0.5875745508	-0.2795568918
H	-2.9342229769	-0.1940008706	-0.6118559223
H	-1.6575708521	-1.4954790805	-0.3115218914
C	0.2975055883	0.1895818413	0.5846808519
H	0.4968309236	0.4107987694	1.6293764286
H	-1.3792880838	1.5212699528	0.1425635588
H	0.7607081753	-1.3356741856	-0.9338947514
H	1.9252377061	-1.3020323002	0.4895862133

Energy of Optimized Geometry: -231.172788763950

11.4 MN15

OX9 3,4-epoxy-1-butene MN15 STRUC1

C	-2.2200025160	-0.0076912768	0.1232599144
C	1.4453802036	-0.6914224543	-0.1077327718
C	-0.9989494193	0.0017900902	-0.3952801134
O	1.3269084541	0.7119693855	-0.0418138903
H	-3.1029900097	0.0251980349	-0.4985660660
H	-2.3700024204	-0.0510363145	1.1953840644
C	0.2276233232	-0.0615410285	0.4188442557
H	0.0846680879	-0.0472363672	1.4951879242
H	-0.8434226664	0.0578460660	-1.4677833151
H	1.4253462121	-1.1131199702	-1.1068256668
H	2.1547435696	-1.1354506477	0.5807666109

Energy of Optimized Geometry: -230.999470929990

OX9 3,4-epoxy-1-butene MN15 STRUC2

C	-1.7796425759	0.2922847984	-0.5058591782
C	1.3169434480	-0.6673225247	-0.2949086920
C	-1.1042968340	-0.2925050833	0.4744664580
O	1.0235638964	0.7138945343	-0.2536233877
H	-2.8408395190	0.1322191373	-0.6313754813
H	-1.2757546500	0.9506427957	-1.2013232544
C	0.3533309386	-0.1160659186	0.6707182550
H	0.6883791470	-0.0043775004	1.6975390215
H	-1.6040012562	-0.9407151223	1.1856292769
H	0.9247876386	-1.1956548877	-1.1566674106
H	2.3136283336	-0.9418438608	0.0304727188

Energy of Optimized Geometry: -230.998911305924

OX9 3,4-epoxy-1-butene MN15 STRUC3

C	-1.9231542547	-0.4449171006	-0.2889212225
C	1.1348063107	-0.8084628747	-0.0623698249
C	-1.0803415802	0.4885045172	0.1306514302
O	1.3496769861	0.5695193751	-0.3035702016
H	-2.9261913431	-0.2001825652	-0.6098152295
H	-1.6342694470	-1.4882929164	-0.3199882686
C	0.3040498542	0.2057604765	0.5863112577
H	0.5194324588	0.4496631556	1.6229813207
H	-1.3886991010	1.5278125446	0.1666181520
H	0.7262275527	-1.3633886457	-0.8990594319
H	1.9130431756	-1.3070110450	0.5031532740

Energy of Optimized Geometry: -230.995661404356

11.5 ωB97X-D

OX9 3,4-epoxy-1-butene ωB97X-D STRUC1

C	-2.2212590157	-0.0206995562	0.1159554663
C	1.4504009491	-0.6835479663	-0.1006969253
C	-1.0017130194	-0.0035545544	-0.3989408248
O	1.3265324507	0.7229401911	-0.0344729697
H	-3.1005094570	0.0099385239	-0.5118491870
H	-2.3805198335	-0.0683623533	1.1864232471
C	0.2266951321	-0.0642876182	0.4139119507
H	0.0817458402	-0.0511881728	1.4902954653
H	-0.8532802478	0.0567167862	-1.4718031879
H	1.4501093342	-1.1034970534	-1.1014213815
H	2.1559018798	-1.1240534591	0.5955266239

Energy of Optimized Geometry: -231.197085787415

OX9 3,4-epoxy-1-butene ωB97X-D STRUC2

C	-1.8032916031	0.2783118169	-0.5121844118
C	1.3324253212	-0.6758703535	-0.2579432857
C	-1.1087917163	-0.2820405385	0.4650276222
O	1.0399091988	0.7094151404	-0.2702152312
H	-2.8648821228	0.1035960759	-0.6154151221
H	-1.3234742078	0.9301586195	-1.2303595919
C	0.3474037674	-0.0972927419	0.6631227710
H	0.6617951292	0.0483460016	1.6931705536
H	-1.5972812911	-0.9217768026	1.1919654146
H	0.9711443189	-1.2303538915	-1.1173490189
H	2.3208245665	-0.9385853201	0.1035846727

Energy of Optimized Geometry: -231.196085575041

OX9 3,4-epoxy-1-butene ωB97X-D STRUC3

C	-1.9440881280	-0.4495982056	-0.2834596011
C	1.1573271311	-0.7966301482	-0.0736877915
C	-1.0840653538	0.4716481417	0.1184923627
O	1.3562014240	0.5928575298	-0.2820160823
H	-2.9454400250	-0.1870503141	-0.5959768803
H	-1.6778188085	-1.4988962384	-0.3108627382
C	0.2978716207	0.1856050392	0.5786131359
H	0.4910521697	0.4088576076	1.6252183918
H	-1.3781272316	1.5154898533	0.1453806837
H	0.7815249952	-1.3408305835	-0.9330345802
H	1.9338106281	-1.2938230656	0.4972520757

Energy of Optimized Geometry: -231.193280042599

11.6 EOM-CCSD

OX9 3,4-epoxy-1-butene EOM-CCSD STRUC1

C	-2.2338898584	-0.0210884006	0.1164112135
C	1.4638125155	-0.6817325410	-0.1014934129
C	-1.0010609109	-0.0193620917	-0.4072184748
O	1.3256462299	0.7437549249	-0.0293809418
H	-3.1147280343	0.0086390773	-0.5118387009
H	-2.3867322569	-0.0553756556	1.1897003580
C	0.2250172643	-0.0770451006	0.4166705901
H	0.0767131460	-0.0672367196	1.4931888647
H	-0.8507004714	0.0277104947	-1.4818075284
H	1.4687011805	-1.0903840446	-1.1071769105
H	2.1771765165	-1.1110110683	0.5940623708

Energy of Optimized Geometry: -230.805889626885

OX9 3,4-epoxy-1-butene EOM-CCSD STRUC2

C	-1.8005600500	0.2723765398	-0.5206896088
C	1.3382991303	-0.6782228277	-0.2561859447
C	-1.1090699720	-0.2931418011	0.4765372217
O	1.0287031119	0.7244998044	-0.2820459203
H	-2.8623332186	0.0952046656	-0.6339229757
H	-1.3080825873	0.9240295936	-1.2322506522
C	0.3508705110	-0.0979192299	0.6746018433
H	0.6736411039	0.0645040337	1.6998892885
H	-1.6042622483	-0.9346575947	1.1996138512
H	0.9709675653	-1.2388958394	-1.1093950218
H	2.3356171557	-0.9198835864	0.0960474269

Energy of Optimized Geometry: -230.805081820477

OX9 3,4-epoxy-1-butene EOM-CCSD STRUC3

C	-1.9432923477	-0.4496739360	-0.2922372260
C	1.1443093628	-0.8140120033	-0.0604475405
C	-1.0837826386	0.4865037292	0.1285731151
O	1.3641104357	0.5867387356	-0.3034781045
H	-2.9452185449	-0.1903754841	-0.6110275248
H	-1.6650833720	-1.4970982367	-0.3239134528
C	0.3003025493	0.1957485577	0.5903015754
H	0.5146084603	0.4392691829	1.6281733021
H	-1.3877900177	1.5286744334	0.1623550232
H	0.7502771299	-1.3638356526	-0.9082561934
H	1.9259005417	-1.3055728728	0.5089259026

Energy of Optimized Geometry: -230.802363018793

12 methyl oxirane (OX10)

12.1 BMK

OX10 methyl oxirane BMK STRUC1

C	-1.5623968876	-0.0641061226	-0.1750516397
C	-0.2089194704	-0.0313532057	0.4932957342
C	0.9779473092	-0.6809837808	-0.1085648730
O	0.8006346882	0.7294547334	-0.1580147601
H	-0.2126719737	0.0918745318	1.5734861082
H	-2.1626918745	-0.8862815714	0.2202511762
H	-2.0986462294	0.8696189864	0.0055149074
H	-1.4474311304	-0.1948758349	-1.2518006152
H	0.8711605957	-1.1906044417	-1.0613853790
H	1.7901364501	-1.0217325592	0.5251281444

Energy of Optimized Geometry: -193.05453709706

OX10 methyl oxirane BMK STRUC2

C	-1.5686348327	0.0241865672	-0.1237462980
C	-0.1910990032	0.1213934667	0.4867798433
C	0.9173871894	-0.7650543974	0.0652873326
O	0.8479519880	0.5879590556	-0.3658857585
H	-0.1421859861	0.5335293596	1.4915672286
H	-1.5075060593	-0.4050171030	-1.1246206578
H	-2.2150822159	-0.6040023200	0.4927525511
H	-2.0225079623	1.0143388858	-0.1981509032
H	0.7338870177	-1.5044902438	-0.7085391066
H	1.7254590259	-0.9897203427	0.7539200765

Energy of Optimized Geometry: -193.054536691664

12.2 CAM-B3LYP

OX10 methyl oxirane CAM-B3LYP STRUC1

C	-1.5502229048	-0.0777714582	-0.1752720620
C	-0.2074015145	-0.0381424139	0.4846915045
C	0.9686196279	-0.6714270119	-0.1014538133
O	0.7967402708	0.7401336682	-0.1573377391
H	-0.2178907405	0.0833357362	1.5638083884
H	-2.1456403813	-0.9015481737	0.2194581046
H	-2.0937352572	0.8491288368	0.0066662779
H	-1.4438685214	-0.2071589549	-1.2509468398
H	0.8738003072	-1.1830486778	-1.0527054790
H	1.7770331981	-1.0124341607	0.5345725252

Energy of Optimized Geometry: -193.088696071331

OX10 methyl oxirane CAM-B3LYP STRUC2

C	-1.5570149285	0.0391027071	-0.1185844994
C	-0.1871996674	0.1209049647	0.4790914282
C	0.8942255520	-0.7707445680	0.0756007321
O	0.8555754798	0.5789990950	-0.3743843130
H	-0.1361612963	0.5395948970	1.4798925899
H	-1.5141954827	-0.3964950925	-1.1152855132
H	-2.2091966655	-0.5741808112	0.5038735331
H	-2.0004317203	1.0315039442	-0.1966488494
H	0.7069129853	-1.5148476068	-0.6904150544
H	1.6951423694	-1.0027715445	0.7676748198

Energy of Optimized Geometry: -193.088695980715

12.3 M06-2X

OX10 methyl oxirane M06-2X STRUC1

C	-1.5500154176	-0.0669004166	-0.1729579516
C	-0.2058468185	-0.0329917243	0.4905225945
C	0.9659386951	-0.6771124877	-0.1062400616
O	0.7972666022	0.7303666473	-0.1595021873
H	-0.2082952751	0.0888073638	1.5693012431
H	-2.1506406442	-0.8867075687	0.2212532685
H	-2.0863959270	0.8652672556	0.0035546023
H	-1.4336922821	-0.1995810351	-1.2477132268
H	0.8543184211	-1.1879390087	-1.0560440750
H	1.7769904454	-1.0196347147	0.5248580617

Energy of Optimized Geometry: -193.098520304914

OX10 methyl oxirane M06-2X STRUC2

C	-1.5509787338	0.1747701530	-0.0274255517
C	-0.1446750511	0.1459011232	0.4917416624
C	0.8284607271	-0.8460009453	0.0299155930
O	0.8738074520	0.4947583835	-0.4315153833
H	0.0079669213	0.5662823284	1.4810154880
H	-1.5938615345	-0.2760719076	-1.0179853756
H	-2.2164996599	-0.3738146196	0.6393286444
H	-1.9101976082	1.2012619927	-0.0995441292
H	0.5199784695	-1.5751389134	-0.7108508135
H	1.6501742183	-1.1396830916	0.6717662361

Energy of Optimized Geometry: -193.098520278422

12.4 MN15

OX10 methyl oxirane MN15 STRUC1

C	-1.5464936231	-0.0664978555	-0.1733695885
C	-0.2048385620	-0.0294243321	0.4938942359
C	0.9616504085	-0.6771068718	-0.1072342184
O	0.7972467517	0.7281048449	-0.1601795937
H	-0.2075630946	0.0917363508	1.5729049661
H	-2.1460786057	-0.8918587516	0.2108037900
H	-2.0909163351	0.8609437364	0.0023546256
H	-1.4200205314	-0.1938091799	-1.2480153972
H	0.8403166853	-1.1917186953	-1.0545405800
H	1.7739830132	-1.0265211434	0.5190463884

Energy of Optimized Geometry: -192.953947907688

OX10 methyl oxirane MN15 STRUC2

C	-1.5534067196	0.0149207952	-0.1119373868
C	-0.1838408759	0.1331675358	0.4851900506
C	0.9037498725	-0.7563202006	0.0757676218
O	0.8401409282	0.5803635763	-0.3851915560
H	-0.1285511978	0.5637926230	1.4803563664
H	-1.4855399932	-0.4321473470	-1.1032043695
H	-2.1945346110	-0.6079349249	0.5120658438
H	-2.0190125143	0.9955208192	-0.2069675419
H	0.7022838927	-1.5136443456	-0.6745291348
H	1.7160228136	-0.9742652686	0.7591407397

Energy of Optimized Geometry: -192.953947925108

12.5 ωB97X-D

OX10 methyl oxirane ωB97X-D STRUC1

C	-1.5520347601	-0.0860596776	-0.1762297592
C	-0.2065264208	-0.0374476090	0.4843428755
C	0.9733393403	-0.6647709685	-0.1033286851
O	0.7934742599	0.7422023513	-0.1547268100
H	-0.2188523590	0.0803169686	1.5646337901
H	-2.1399976297	-0.9142103171	0.2215515974
H	-2.1008601532	0.8381729131	0.0060906130
H	-1.4461436994	-0.2168427228	-1.2526051732
H	0.8796325472	-1.1762050446	-1.0561161327
H	1.7827109145	-1.0046275763	0.5337389231

Energy of Optimized Geometry: -193.121194608896

OX10 methyl oxirane ωB97X-D STRUC2

C	-1.5394054885	0.1999245814	-0.1936739475
C	-0.2031638316	0.0916888842	0.4784735043
C	0.8165135991	-0.8555182907	0.0379984588
O	0.9254736654	0.5267345637	-0.2652257790
H	-0.1765732794	0.3994508553	1.5204100256
H	-1.4773311690	-0.1388844603	-1.2272514120
H	-2.2780277033	-0.4076369750	0.3308142328
H	-1.8861874270	1.2335643327	-0.1902571510
H	0.6079210642	-1.4984188356	-0.8113227481
H	1.5486466682	-1.2334165385	0.7434319638

Energy of Optimized Geometry: -193.121194616611

12.6 EOM-CCSD

OX10 methyl oxirane EOM-CCSD STRUC1

C	-1.5548572387	-0.0680877012	-0.1800980813
C	-0.2102170968	-0.0395875442	0.4925905022
C	0.9659947390	-0.6857374615	-0.1034429388
O	0.8052940872	0.7440454451	-0.1565983241
H	-0.2171180376	0.0807188702	1.5729752653
H	-2.1589141859	-0.8915463829	0.2092723374
H	-2.0920757094	0.8656588550	-0.0006532712
H	-1.4336880422	-0.1965388692	-1.2570635176
H	0.8596139954	-1.1899879436	-1.0588755726
H	1.7760842889	-1.0298165066	0.5305574331

Energy of Optimized Geometry: -192.797161765688

OX10 methyl oxirane EOM-CCSD STRUC2

C	-1.5597313889	0.0482186166	-0.1399645294
C	-0.1947134336	0.1122793005	0.4877264795
C	0.8904665129	-0.7849235764	0.0710213900
O	0.8673113386	0.5900918360	-0.3552497678
H	-0.1499657165	0.5104547306	1.4982725549
H	-1.4915117915	-0.3686511004	-1.1462461377
H	-2.2259602663	-0.5781399939	0.4586952601
H	-1.9968879900	1.0467393278	-0.2073234842
H	0.7004566568	-1.5072957358	-0.7167046583
H	1.6862504907	-1.0333635325	0.7649965928

Energy of Optimized Geometry: -192.797161765770
